# Genome-Wide Identification and Expression Profiling of Candidate Sex Pheromone Biosynthesis Genes in the Fall Armyworm (*Spodoptera frugiperda*)

**DOI:** 10.3390/insects13121078

**Published:** 2022-11-23

**Authors:** Cheng Qu, Zhiwei Kang, Biyun Zhang, Yong Fang, Ran Wang, Fengqi Li, Haipeng Zhao, Chen Luo

**Affiliations:** 1 Institute of Plant Protection, Beijing Academy of Agriculture and Forestry Sciences, Beijing 100097, China; 2 School of Life Science, Institute of Life Science and Green Development, Hebei University, Baoding 071002, China; 3 Hunan Agricultural Biotechnology Research Institute, Hunan Academy of Agricultural Sciences, Changsha 410125, China; 4 College of Plant Protection, Shandong Agricultural University, Tai'an 271018, China

**Keywords:** *Spodoptera frugiperda*, genome, sex pheromone gland, biosynthesis pathway

## Abstract

**Simple Summary:**

The fall armyworm (FAW), *Spodoptera frugiperda,* is a serious worldwide agricultural pest, threatening food security and crop production. Sex pheromone lures are commonly used in population monitoring and biological control of FAWs. Although the sex pheromone components of the FAW have been successfully identified, there are no reports on the molecular mechanism of FAW sex pheromone biosynthesis. In this study, we identified a total of 99 genes related to the biosynthesis of sex pheromones from the *S. frugiperda* genome, which belonged to 11 families of genes. Based on gene expression patterns and phylogenetic analysis, several genes had PG-biased expression, indicating that they may play an important role in sex pheromone biosynthesis. These results could lay a solid foundation for understanding the molecular mechanisms of *S. frugiperda* sex pheromone biosynthesis and provide new targets for developing novel pest control methods based on disrupting sexual communication.

**Abstract:**

*Spodoptera frugiperda* is an agricultural pest causing substantial damage and losses to commercial crops. Sex pheromones are critical for successful mating in Lepidoptera and have been used for monitoring and control of many pest species. The sex pheromone of *S. frugiperda* is known, but the genes involved in its biosynthesis have not been identified. We systematically studied 99 candidate sex pheromone genes in the genome of *S. frugiperda* including 1 acetyl-CoA carboxylase (ACC), 11 fatty acid synthases (FASs), 17 desaturases (DESs), 4 fatty acid transport proteins (FATPs), 29 fatty acyl-CoA reductases (FARs), 17 acetyl-CoA acetyltransferases (ACTs), 5 acyl-CoA dehydrogenase (ACDs), 3 enoyl-CoA hydratases (ECHs), 3 hydroxyacyl-CoA dehydrogenases (HCDs), 6 ethyl-CoA thiolases (KCTs), and 3 acyl-CoA-binding proteins (ACBPs). Based on the comparative transcriptome results, we found 22 candidate sex pheromone biosynthesis genes predominately expressed in pheromone glands (PGs) than abdomens without PGs including *SfruFAS4*, *SfruFATP3*, *SfruACD5*, *SfruKCT3*, *SfruDES2*, *SfruDES5*, *SfruDES11*, *SfruDES13*, *SfruFAR1*, *SfruFAR2*, *SfruFAR3*, *SfruFAR6*, *SfruFAR7*, *SfruFAR8*, *SfruFAR9*, *SfruFAR10*, *SfruFAR11*, *SfruFAR14*, *SfruFAR16*, *SfruFAR29*, *SfruACT6*, and *SfruACT10*. A combination of phylogenetic and tissue-specific transcriptomic analyses indicated that *SfruDES5*, *SfruDES11*, *SfruFAR2*, *SfruFAR3*, and *SfruFAR9* may be key genes involved in the sex pheromone synthesis of *S. frugiperda*. Our results could provide a theoretical basis for understanding the molecular mechanisms of sex pheromone biosynthesis in *S. frugiperda*, and also provide new targets for developing novel pest control methods based on disrupting sexual communication.

## 1. Introduction

Female Lepidoptera (moths) release sex pheromones to attract males for mating [1,2]. Most moth sex pheromones consist of two or more compounds combined in precise ratios with species specificity [3,4]. Based on the difference of their chemical structures and biosynthetic features, sex pheromones are classified into type I and type II [5]. Most known sex pheromones are type I, which are usually synthesized in female sex pheromone glands (PGs) situated in the intersegmental membrane between the eighth and ninth abdominal segments [5,6]. These pheromone components are mainly C10–C18 straight-chain compounds, containing 0–4 double bonds in different positions. The carbon chain ends have alcohol, ester, or aldehyde functional groups [7,8]. During type I sex pheromone biosynthesis, fatty acid intermediates such as palmitic acid or stearic acid are used as precursors. The double bond is generated by the desaturation system, and a short-chain reaction is carried out by a special β-oxidase system [9,10]. Oxidase, fatty acyl-CoA reductase, and acyl transferase catalyze the formation of functional groups such as esters, aldehydes, and alcohols to form a mixture of sex pheromones with specific component ratios and amounts. Acetyl-CoA carboxylase (ACC), fatty acid synthase (FAS), fatty acid transport protein (FATP), acyl-CoA dehydrogenase (ACD), 3-ketoacyl-CoA thiolase (KCT), hydroxyacyl-CoA dehydrogenase (HCD), enoyl-CoA hydratase (ECH), desaturase (DES), fatty acyl-CoA reductase (FAR), acetyl-CoA acetyltransferase (ACT), acyl-CoA-binding protein (ACBP) are the key enzymes in sex pheromone biosynthesis of moths [8,11,12].

The fall armyworm (FAW), *Spodoptera frugiperda*, native to tropical and subtropical America, is an agricultural pest. It has a wide plant host range, strong migratory ability, high reproductive capacity, and invaded sub-Saharan Africa, Asia, and other regions [13,14,15]. In China, the FAW was first found in Yunnan in January 2019. It then spread rapidly to many other food-producing areas in China and threatened food security and crop production [16,17]. Sex pheromone lures are usually used for population monitoring and biological control of FAW [18,19,20,21,22]. The sex pheromone components of FAW usually contain several acetates, including (Z)-9-Tetradeceny1 acetate (Z9-14:OAc) as a major component, (Z)-7-Dodecenyl acetate (Z7-12:OAc), (Z)-9-dodecenyl acetate (Z9-12:OAc), (Z)-11-hexadecell acetate (Z11-16:OAc), and trans-7-Dodecen acetate-1-yl acetate (E7-12:OAc); these acetate esters are all type-I sex pheromones [23,24,25,26,27,28,29]. Although the sex pheromone components of FAW have been successfully identified, there are no reports on the molecular mechanism of FAW sex pheromone biosynthesis.

In this study, we identified candidate FAW sex pheromone biosynthesis genes using previously published genome data. Then, we sequenced the transcriptome of PGs and ABs (abdomen without PGs) to analyze the expression of these candidate sex pheromone biosynthesis genes. Quantitative RT-PCR (qRT-PCR) was conducted to validate the transcriptome results. Based on the gene identifications, phylogenetic, and tissue-specific expression analyses, several genes were identified as potentially involved in FAW sex pheromone biosynthesis. Our results could provide potential targets for developing environmentally friendly control methods.

## 2. Materials and Methods

### 2.1. Insect Rearing and Tissue Collection

The original population of *S. frugiperda* was collected from a maize field of Dehong Dai and Jingpo Autonomous Prefecture, Yunnan Province, China. Larvae were reared with maize leaves in a growth chamber at (25 ± 1) °C, 55% relative humidity, with a 16:8 h (L:D) photoperiod. Adults were fed with 10% honey water. For the transcriptome sequencing and tissue expression study, 20–25 PGs (with the ovipositor) and 10–15 abdomens (without the PGs) were collected from 3 d old virgin female adults at 6–7 h into the scotophase [24,25]. The FAW shows particularly high mating activity at this time. Three biological replicates were conducted.

### 2.2. RNA Isolation, cDNA Library Construction, Illumina Sequencing

Total RNA was extracted using TRIzol reagent (Invitrogen, Carlsbad, CA, USA) according to manufacturer instructions. The quality and concentration of RNA samples were checked using a NanoDrop 2000 UV-Vis spectrophotometer (Thermo Fisher Scientific Inc., Waltham, MA, USA), and the RNA integrity was confirmed using an Agilent 2100 Bioanalyzer (Agilent Technologies, Davis, CA, USA).

Oligo (dT) magnetic beads were used to purify mRNA from total RNA, and the mRNA was randomly interrupted in the NEB fragmentation buffer. Fragmented mRNA were used as templates to synthesize the first strand of cDNA using a random hexamer, followed by synthesis of the second strand, cDNA, by adding RNaseH, dNTPs, and DNA polymerase I. After end-repair, poly-A tailing, and ligation of adapters, the cDNA was purified by an AMPure XP system (Beckman Coulter, Beverly, MA, USA), and PCR amplification was performed. The constructed library was quality-tested with an Agilent 2100 Bioanalyzer (Agilent Technologies, Davis, CA, USA).

An Illumina NovaSeq 6000 (Illumina, San Diego, CA, USA) was used for sequencing. The raw reads were processed to remove low-quality reads, poly-N reads, and adapter reads to obtain the clean reads. Q20, Q30, and GC content were used to assess the sequencing quality. HISAT2 v.2.0.4 software was used to assemble and compare clean data and obtain annotations in the reference genome of *S. frugiperda* (https://ftp.ncbi.nlm.nih.gov/genomes/all/GCF/011/064/685/GCF_011064685.1_ZJU_Sfru_1.0/, (accessed on 24 July 2020)). The RNA sequence data was uploaded to the NCBI platform (BioProject Accession: PRJNA885519). Fragments per kilobase of exon per million mapped reads (FPKM) were used to evaluate the expression level.

### 2.3. Identification and Analysis of Sex Pheromone Biosynthesis-Related Genes

The *S. frugiperda* genome database was obtained from NCBI (https://ftp.ncbi.nlm.nih.gov/genomes/all/GCF/011/064/685/GCF_011064685.1_ZJU_Sfru_1.0/, (accessed on 24 July 2020)). The amino acid sequences of sex pheromone biosynthesis genes (ACCs, FASs, FATPs, ACDs, ECHs, HCDs, KCTs, DESs, FARs, ACTs, ACBPs) in *Spodoptera exigua*, *Spodoptera litura*, *Helicoverpa armigera*, *Helicoverpa assulta*, *Helicoverpa zea*, *Agrotis segetum*, *Agrotis ipsilon*, *Heliothis virescens* were used as query sequences (Table S1) for local BLASTP search (E-value cutoff of <1 × 10^−5^ and identity >30%) against the *S. frugiperda* genome database [8,30,31,32,33,34,35,36,37,38]. The incomplete sequences and duplicated genes were removed to obtain initial genes. All of these potential sex pheromone biosynthesis genes were then verified by BLASTP in NCBI with the E-value < 1 × 10^−5^ and identity >30%. The location of these genes was generated based on the genome annotation of *S. frugiperda* (BioProject Accession: PRJNA590312) using TBtools software (v1.0987671).

### 2.4. cDNA Synthesis and Full-Length cDNA Cloning 

The cDNA was synthesized using the PrimeScriptTMRT reagent Kit with gDNA Eraser (Perfect Real Time) (TAKARA, Toyoko, Japan). Four differentially expressed genes (*SfurDES2*, *SfurDES5*, *SfurFAR2*, *SfurFAR3*) were randomly selected to amplify the full-length ORF sequence of these genes by using TransStart FastPfu Fly PCR Supermix (TransGen Biotech, Beijing, China). PCR conditions were: 5 min at 94 °C, followed by 40 cycles of 94 °C for 20 s, 20 s at 52 °C, and 45 s at 72 °C, followed by incubation at 72 °C for 10 min, carried out in a Bio-Rad thermocycler (Bio-Rad DNA Engine Peltier Thermal Cycler, Bio-Rad, Hercules, CA, USA). The primers were listed in Table S2, designed by Primer 5.0 software. The products were gel-purified and ligated into a pEASY-blunt vector (TransGen Biotech, Beijing, China). The ligation products were transformed into Trans T1 competent cells (TransGen Biotech, Beijing, China). All sequencing was performed by Tsingke Biotechnology Co., Ltd. (Beijing, China).

### 2.5. Phylogenetic Analysis

Phylogenetic trees were performed for SfruDESs and SfruFARs with their corresponding homologous genes from *S. exigua*, *S. litura*, *Sesamia inferens*, *A. pernyi*, *Ostrinia nubilalis*, and *H. assulta,* as reported previously [8,30,39]. The DES dataset included 17 sequences from *S. frugiperda*, and 41 from the other six species (12 from *S. litura*, 10 from *S. exigua*, 6 from *S. inferens*, 6 from *A. pernyi*, 3 from *O. nubilalis*, and 4 from *H. assulta*). The FAR dataset included 29 sequences from *S. frugiperda*, and 48 from six other insects (13 from *S. litura*, 13 from *S. exigua*, 3 from *S. inferens*, 11 from *A. pernyi*, 7 from *O. nubilalis*, and 1 from *H. assulta*) (Table S3). Amino acid sequences were aligned by ClatalW, and phylogenetic trees were constructed by MEGA X using the neighbor-joining method with position correction of distances and 1000 bootstrap replications. The final phylogenetic tree is displayed in the form of a circular tree diagram, and the color of each branch is labeled using FigTree v1.3.1.

### 2.6. Quantitative RT-PCR and Data Analysis

Four differentially expressed genes (*SfurDES2*, *SfurDES5*, *SfurFAR2*, *SfurFAR3*) were verified by qRT-PCR. Primers for qRT-PCR were designed by NCBI Primer-BLAST (https://www.ncbi.nlm.nih.gov/tools/primer-blast/ (accessed on 24 July 2020)). The 10-fold dilution series of cDNA from the PGs of *S. frugiperda* was used for a standard curve. The corresponding qRT-PCR efficiencies (E) were counted using the equation: E = (10 [−1/slope] − 1) × 100 [40] (Table S4). EF1α and RPS18 were selected as internal reference genes [41]. The qRT-PCR was performed on the ABI PRISM 7500 qRT-PCR System (Applied Biosystems, Foster City, CA, USA) using SYBR Premix Ex TaqTM II (TaKaRa, Toyoko, Japan) under the following conditions: 95 °C for 30 s, followed by 40 cycles of 95 °C for 5 s, and 60 °C for 34 s. Relative quantification was performed using the 2^−ΔΔCT^ method [42].

## 3. Results

### 3.1. Identification and Localization of the Candidate Sex Pheromone Biosynthesis Genes in the S. frugiperda Genome

**Figure 1:**
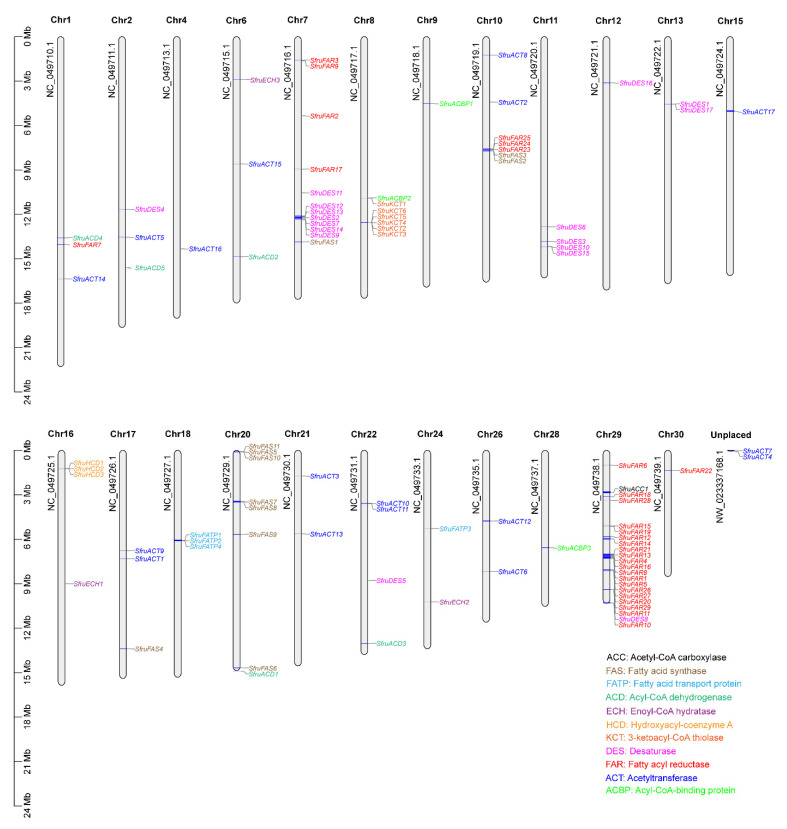
Localization of the candidate sex pheromone biosynthesis genes in the *S. frugiperda* genome. According to the annotation file of *S. frugiperda*, we determined the sex pheromone biosynthesis gene locations in the genome.

We identified 99 genes encoding pheromone biosynthesis from 11 gene families, including 1 ACC, 11 FASs, 4 FATPs, 5 ACDs, 3 ECHs, 3 HCDs, 6 KCTs, 17 DESs, 29 FARs, 17 ACTs, and 3 ACBPs (Table 1). To clarify the position of sex pheromone biosynthesis genes in chromosomes, chromosome location analysis of these genes was carried out. The results showed that except for two ACTs that were unplaced, the rest of the candidate sex pheromone biosynthesis genes were distributed across 23 chromosomes (Figure 1). ACC was located on chromosome 29. All of the KCT and HCD genes were distributed on only one chromosome (KCTs: chromosome 8 and HCDs: chromosome 16) (Figure 1). Four FATPs were found on chromosomes 18 (*SfruFATP1*, *SfruFATP2*, and *SfruFATP4*) and 24 (*SfruFATP3*) (Figure 1). Both of ECHs and ACBPs were located on three chromosomes (ECHs: chromosomes 6, 16, and 24; ACBPs: chromosomes 8, 9, and 28) (Figure 1). Seven FASs were clustered together on chromosome 20, followed by chromosomes 10 (*SfruFAS2* and *SfruFAS3*), 7 (*SfruFAS1*), and 17 (*SfruFAS4*) (Figure 1). Five ACDs were independently distributed on five chromosomes (1: *SfruACD4*, 2: *SfruACD5*, 6: *SfruACD2*, 20: *SfruACD1*, and 22: *SfruACD22*) (Figure 1). Thirty FARs were also located on five chromosomes (1, 7, 10, 29, and 30) (Figure 1). DESs and ACTs were widely distributed on more than five chromosomes (DESs: chromosomes 2, 7, 11, 12, 13, 22, and 29; ACTs: chromosomes 1, 2, 4, 6, 10, 15, 17, 21, 22 and 26) (Figure 1).

### 3.2. Phylogenetic Analyses of DESs and FARs

**Figure 2:**
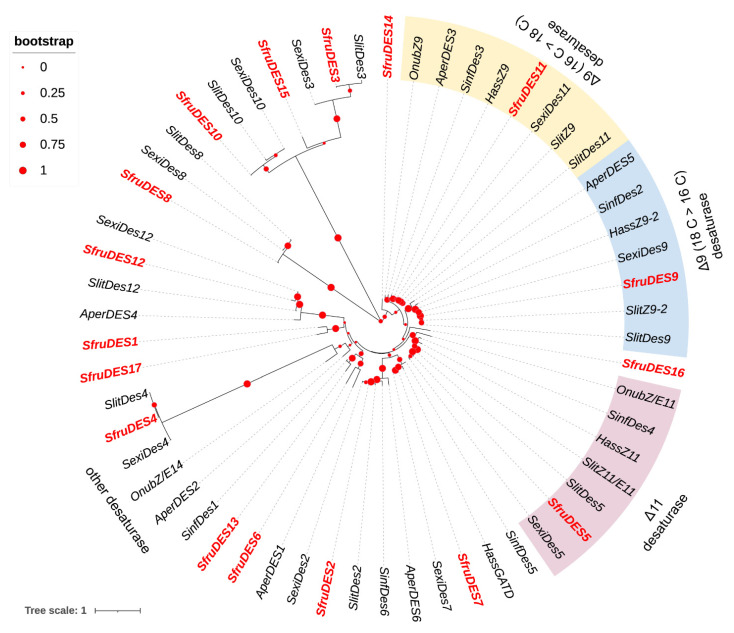
Phylogenetic tree of insect desaturase (DES). The *S. frugiperda* translated genes are shown in red. Sfru: *S. frugiperda*, Slit: *S. litura*, Sexi: *S. exigua*, Sinf: *S. inferens*, Aper: *A. pernyi*, Onub: *O. nubilalis*, Hass: *H. assulta*.

**Figure 3:**
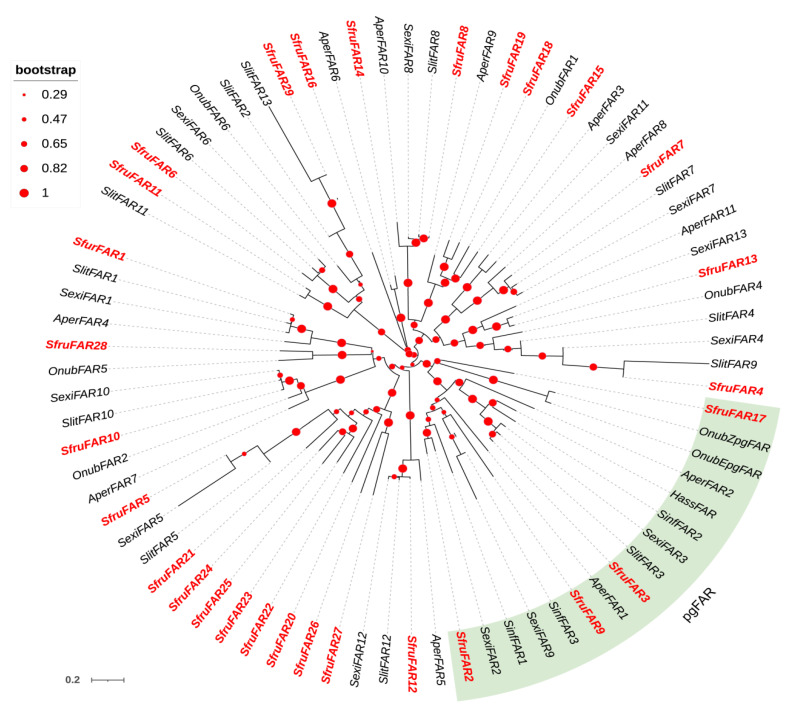
Phylogenetic tree of insect fatty acid reductase (FAR). The *S. frugiperda* translated genes are shown in red. Sfru: *S. frugiperda*, Slit: *S. litura*, Sexi: *S. exigua*, Sinf: *S. inferens*, Aper: *A. pernyi*, Onub: *O. nubilalis*, Hass: *H. assulta*.

To assign putative functions, two phylogenetic trees of DESs and FARs were constructed using protein sequences from *S. frugiperda*, *S. exigua*, *S. litura*, *S. inferens*, *A. pernyi*, *O. nubilalis*, and *H. assulta*. The DESs phylogenetic trees showed that all three identified SfruDESs from the *S. frugiperda* genome were clustered in three different clades of Lepidoptera desaturases: Δ11 desaturase (*SfruDES5*), Δ9 desaturase (18 C > 16 C) (*SfruDES9*), and Δ9 desaturase (16 C > 18 C) (*SfruDES11*) (Figure 2). In the FARs phylogenetic tree, four SfruFARs (*SfruFAR2*, *SfruFAR3*, *SfruFAR9*, and *SfruFAR17*) were clustered within the Lepidoptera pgFAR group, which is a clade with functionally investigated FARs (Figure 3).

### 3.3. Expression Profile of Sex Pheromone Biosynthesis Genes

**Figure 4:**
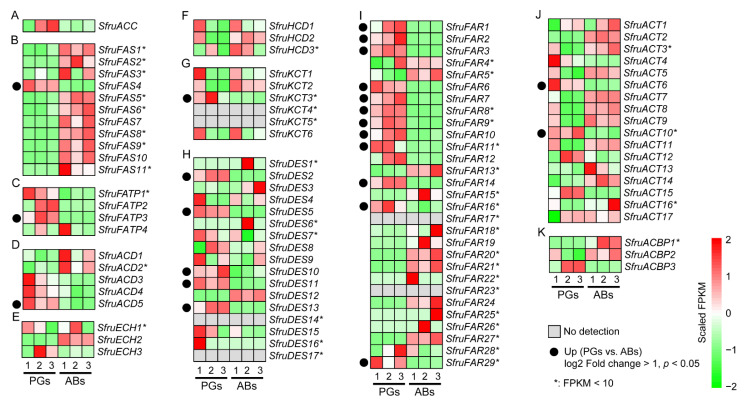
Expression profiles of the candidate sex pheromone biosynthesis genes in *S. frugiperda*. (**A**) ACC; (**B**): FASs; (**C**): FATPs; (**D**): ACDs; (**E**): ECHs; (**F**): HCDs; (**G**): KCTs; (**H**): DESs; (**I**); FARs; (**J**): ACTs; (**K**): ACBPs.

**Figure 5:**
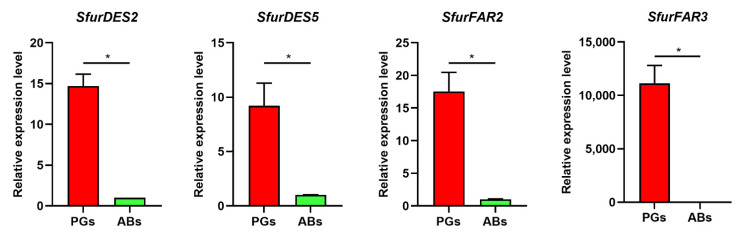
Relative expression levels of four sex pheromone biosynthesis genes. PGs: female pheromone glands, ABs: female abdomens without pheromone glands. The relative expression level (PGs VS ABs) is indicated as mean ± SE (*n* = 3). The asterisk indicates a significant difference between PGs and ABs (*p* < 0.05, Mann-Whitney U test).

To further screen the candidate sex pheromone biosynthesis genes in *S. frugiperda*, we performed a transcriptome analysis for the genes expressed in female PGs and ABs (Table S5). Based on the transcriptome results (Table S6), we analyzed the expression patterns of all of the candidate sex pheromone biosynthesis genes (Figure 4). None of the genes in ACC, ECH, HCD, and ACBP were significantly higher expressed in the PGs (Figure 4). Only one gene in FAS (*SfruFAS4*), FATP (*SfruFATP3*), ACD (*SfruACD5*), and KCT (*SfruKCT3*) had significantly higher expression levels in pheromone glands (PGs) than abdomens (ABs) (Figure 4). Five DESs (*SfruDES2*, *SfruDES5*, *SfruDES11*, and *SfruDES13*), twelve FARs (*SfruFAR1*, *SfruFAR2*, *SfruFAR3*, *SfruFAR6*, *SfruFAR7*, *SfruFAR8*, *SfruFAR9*, *SfruFAR10*, *SfruFAR11*, *SfruFAR14*, *SfruFAR16*, and *SfruFAR29*) and two ACTs (*SfruACT6* and *SfruACT10*) were predominately expressed in the PGs of *S. frugiperda* (Figure 4). *SfruFAR3* was specifically highly expressed in PGs (Figure 4I). Among the predominately expressed genes in the PGs, the FPKM of *SfruKCT3*, *SfruFAR8*, *SfruFAR9*, *SfruFAR11*, *SfruFAR16*, *SfruFAR29*, and *SfruACT10* were below 10, while the FPKM of *SfruFAS4*, *SfruACD5*, *SfruDES5*, *SfruDES11*, *SfruFAR3*, *SfruFAR10*, *SfruFAR14*, and *SfruACT6* exceeded 100 (Figure 4). The qRT-PCR validation experiments of *SfruDES2*, *SfruDES5*, *SfruFAR2*, and *SfruFAR3* confirmed that all of these four genes were predominately expressed in the PGs (Figure 5).

## 4. Discussion

In moths, the PGs are the most important organ for synthesizing and releasing sex pheromones [35]. Thus, the sex pheromone synthesis genes are usually highly expressed in PGs. In the present study, 99 candidate sex pheromone biosynthesis genes were identified from the genome of *S. frugiperda*. Among them, 22 genes were expressed at significantly higher levels in PGs than in the abdomen, and most of them were key genes involved in the sex pheromone biosynthesis pathway of moths such as DESs and FARs. Consistent with this study, DESs and FARs also showed a trend of high PG expression in *S. litura* and *S. exigua* [8,30].

Sex pheromones released by female moths are composed of a mixture of sex pheromone components in specific ratios that show high species specificity [43,44]. Synthesis of a specific sex pheromone mixture requires the coordination of multiple enzymes, such as ACC and FAS. These two enzyme families mainly work upstream of sex pheromone synthesis and are responsible for the synthesis of fatty acid precursors. Initially, ACC carboxylates acetyl-CoA to malonyl-CoA [45], after which FAS synthesizes malonyl-CoA and NAPDH into fatty acids [46,47]. In this study, one ACC gene and 11 FAS genes were identified from the genome of *S. frugiperda*. Among the 11 FAS genes, *SfurFAS4* had the highest expression in PG, suggesting its important role in fatty acid synthesis. As an evolutionarily conserved membrane-bound protein, FATPs can bind fatty acids and transport them to PG cells via the hemolymph for pheromone biosynthesis [12,48]. Four FATP genes were identified from the genome of *S. frugiperda*, consistent with the number of FATP genes in *S. litura* and *S. exigua* [8,30], and there was a high degree of sequence similarity among these three species. However, only *SfurFATP3* had abundant expression levels in the PGs based on the FPKM values.

There are several sex pheromone components of *S. frugiperda*. Except for Z11-16:OAc, containing 16 carbons, Z9-14:OAc, Z7-12:OAc, Z9-12:OAc, and E7-12:OAc are less than 16-carbon chain length unsaturated fatty acid ester derivatives [24,28]. Therefore, the carbon chain shortening reaction plays an important role in this process, and the pathway to generate sex pheromone precursors with different carbon chain lengths is similar to the local β-oxidation pathway of vertebrate peroxisomes, among which ACD, ECH, HCD, and KCT are four key enzymes in the β-oxidation pathway [9]. A total of five ACD genes, three ECH genes, three HCD genes, and six KCT genes were screened in the FAW genome. *SfurACD5* and *SfurKCT3* had higher expression abundance in PGs than in the abdomen.

DES is a key enzyme in sex pheromone biosynthesis. It removes hydrogen atoms at specific positions and introduces double bonds to form cis-trans isomers [31,49]. DES is classified into ∆5, ∆9 (18 C > 16 C) ∆9 (16 C > 18 C), ∆10, ∆11, ∆12, and ∆14 desaturases according to the position where the double bond is introduced into the catalytic substrate [50,51,52]. Because several sex pheromone components of *S. frugiperda* have different positions, numbers, and configurations of double bonds, DES is crucial for sex pheromone formation. A total of 17 DESs were identified from the FAW genome. Phylogenetic tree analysis showed that the *SfruDES5* was clustered with the DES5 of *S. litura* and *S. exigua*, and both were clustered in the ∆11 DES branch. Both transcriptome FPKM values and qRT-PCR showed that *SfruDES5* was significantly overexpressed in the PGs of *S. frugiperda*. *SfruDES9* and *SfruDES11* are clustered with the corresponding DES9 and DES11 of *S. litura* and *S. exigua* and belong to the Δ9 (18 C > 16 C) and Δ9 (16 C > 18 C) desaturase groups, respectively, in which *SfruDES11* was specifically expressed in PGs. The Δ9 and Δ11 desaturases are important desaturases in *Spodoptera*. The Δ9 desaturases and Δ11 desaturases can introduce Δ9-double bonds and Δ11-double bonds in precursors [53]. The Z9-14:OAc and Z11-16:OAc are the key components of *S. frugiperda* sex pheromone, and Δ9 and Δ11 desaturases are key enzymes for introducing the Δ9 and Δ11 double bonds into the pheromone. Therefore, *SfurDES5* and *SfurDES11* may participate in the desaturation step from saturated to unsaturated acids during sex pheromone synthesis in *S. frugiperda*.

The precursor substance forms an intermediate product with a specific length and double bond position after the desaturation reaction and chain shortening reaction. It then needs to be catalyzed by reductases to form alcohols. During this process, FAR is responsible for converting unsaturated fatty acids into the corresponding alcohols [11,54]. In our study, a total of 29 FAR genes were identified from the genome; among the 29 FARs of *S. frugiperda*, 12 FARs were specifically highly expressed in the PGs. The phylogenetic tree showed that *SfruFAR3* was clustered with FAR3 of *S. litura* and *S. exigua*, belonging to the PgFAR subfamily of *Spodoptera*, and had a high expression abundance. This indicated that this gene may play an important role in the synthesis of precursor alcohols. ACT can catalyze the formation of esters from alcohols [55,56]. Since there are only esters in the *S. frugiperda* sex pheromone, ACT genes play a key role in sex pheromone biosynthesis. A total of 17 ACT genes were identified in the FAW genome, among which the *SfurACT6* and *SfurACT10* were highly expressed in the PGs. These two genes may play a role in the process of converting alcohol to esters. Furthermore, two ACTs were unplaced in the chromosome, which might be caused by the genome quality. In *Bombyx mori*, ACBP functions as acyl-CoA or cell deposition [12]. We identified three ACBPs from the *S. frugiperda* genome.

According to the sex pheromone components of *S. frugiperda,* we speculated the *S. frugiperda* sex pheromone biosynthesis pathway. Firstly, ACC catalyzes acetyl-CoA to malonyl-CoA, which is followed by FAS to produce the most saturated palmitic acid (16: CoA). The 16: CoA was desaturated by Δ11 desaturase (Z11/E11) to produce Z11-16: CoA/E11-16: CoA. After the desaturase-induced formation of a double bond, specific β-oxidation enzymes shorten the chains to Z9-14: CoA, Z7-12: CoA, or E7-12: CoA. In addition, the 16: CoA was shortened by β-oxidation enzymes to 12: CoA. Then, 12: CoA generated Z9-12: CoA under Δ9 desaturase (Z9). These acyl-CoA precursors were further reduced by FARs to form corresponding fatty alcohols. Finally, pheromone components were produced after the oxidation by ACTs (Figure S1).

## 5. Conclusions

In conclusion, we identified a total of 99 genes belonging to gene families involved in the biosynthesis of sex pheromones from the *S. frugiperda* genome. Based on gene expression patterns and phylogenetic analysis, several genes highly expressed in the PGs might play an important role in sex pheromone synthesis. The specific functions of these genes in the process of sex pheromone biosynthesis in *S. frugiperda* require further study.

## Figures and Tables

**Table 1:** Genes related to sex pheromone biosynthesis in *Spodoptera frugiperda*.

Gene Name	Gene ID	ORF (aa)	Gene_chr	Gene Start	Gene End	Best BlastX Match				
						Gene Name	Acc.no.	Species	E Value	Identity (%)
Acetyl-CoA carboxylase (ACC)										
*SfruACC1*	LOC118280410	2385	NC_049738.1	2,772,652	2,888,149	acetyl-CoA carboxylase	XP_022824105.1	*Spodoptera litura*	0.0	99.20
Fatty acid synthase (FAS)										
*SfruFAS1*	LOC118265122	1503	NC_049716.1	13,860,534	13,873,617	fatty acid synthase-like	XP_035448777.1	*Spodoptera frugiperda*	0.0	100
*SfruFAS2*	LOC118267601	2337	NC_049719.1	7,708,838	7,745,487	fatty acid synthase-like	XP_035437568.1	*Spodoptera frugiperda*	0.0	100
*SfruFAS3*	LOC118267741	2328	NC_049719.1	7,644,074	7,683,559	fatty acid synthase-like	XP_035437791.1	*Spodoptera frugiperda*	0.0	100
*SfruFAS4*	LOC118272899	1286	NC_049726.1	13,387,368	13,397,181	fatty acid synthase	AGR49310.1	*Agrotis ipsilon*	0.0	85.25
*SfruFAS5*	LOC118274970	2384	NC_049729.1	88,602	113,004	fatty acid synthase-like	XP_035448683.1	*Spodoptera frugiperda*	0.0	100
*SfruFAS6*	LOC118275042	1580	NC_049729.1	14,669,977	14,692,392	fatty acid synthase-like	XP_035448779.1	*Spodoptera frugiperda*	0.0	100
*SfruFAS7*	LOC118274634	2422	NC_049729.1	3,425,920	3,481,831	fatty acid synthase-like	XP_035448162.1	*Spodoptera frugiperda*	0.0	100
*SfruFAS8*	LOC118274778	2348	NC_049729.1	3,486,227	3,504,960	fatty acid synthase-like	XP_035448370.1	*Spodoptera frugiperda*	0.0	100
*SfruFAS9*	LOC118274999	2276	NC_049729.1	5,659,108	5,676,788	fatty acid synthase-like	XP_035448720.1	*Spodoptera frugiperda*	0.0	100
*SfruFAS10*	LOC118274601	2321	NC_049729.1	117,349	133,649	fatty acid synthase-like	XP_047033130.1	*Helicoverpa zea*	0.0	68.91
*SfruFAS11*	LOC118274969	294	NC_049729.1	84,898	87,981	fatty acid synthase-like	XP_035448682.1	*Spodoptera frugiperda*	0.0	100
Fatty acid transport protein (FATP)										
*SfruFATP1*	LOC118273519	700	NC_049727.1	6,041,998	6,085,508	fatty acid transport protein	ARD71229.1	*Spodoptera exigua*	0.0	96.43
*SfruFATP2*	LOC118273025	651	NC_049727.1	6,086,086	6,101,822	fatty acid transport protein	ARD71230.1	*Spodoptera exigua*	0.0	98.00
*SfruFATP3*	LOC118277430	661	NC_049733.1	5,268,282	5,285,638	fatty acid transport protein	ARD71231.1	*Spodoptera exigua*	0.0	96.37
*SfruFATP4*	LOC118273026	643	NC_049727.1	6,103,562	6,131,706	fatty acid transport protein	ARD71232.1	*Spodoptera exigua*	0.0	98.29
Acyl-CoA dehydrogenase (ACD) (β-oxidation enzyme)										
*SfruACD1*	LOC118274515	408	NC_049729.1	14,890,759	14,893,193	short-chain specific acyl-CoA dehydrogenase, mitochondrial	XP_022831790.1	*Spodoptera litura*	0.0	99.02
*SfruACD2*	LOC118264809	410	NC_049715.1	14,866,408	14,875,265	acyl-CoA dehydrogenase	QZC92122.1	*Dioryctria abietella*	0.0	72.68
*SfruACD3*	LOC118276297	418	NC_049731.1	13,012,363	13,027,495	short/branched-chain specific acyl-CoA dehydrogenase, mitochondrial	XP_022827910.1	*Spodoptera litura*	0.0	99.28
*SfruACD4*	LOC118265626	422	NC_049710.1	13,581,976	13,599,130	putative medium-chain specific acyl-CoA dehydrogenase	AID66670.1	*Agrotis segetum*	0.0	96.45
*SfruACD5*	LOC118278275	626	NC_049711.1	15,605,655	15,609,993	very long-chain specific acyl-CoA dehydrogenase, mitochondrial	XP_047020249.1	*Helicoverpa zea*	0.0	89.97
Enoyl-CoA hydratase (ECH) (β-oxidation enzyme)										
*SfruECH1*	LOC118271967	278	NC_049725.1	8,995,073	8,998,854	enoyl-CoA hydratase domain-containing protein 3, mitochondrial-like	XP_035444142.1	*Spodoptera frugiperda*	0.0	100
*SfruECH2*	LOC118277211	343	NC_049733.1	10,231,633	10,237,455	putative enoyl-CoA hydratase	AID66686.1	*Agrotis segetum*	0.0	77.42
*SfruECH3*	LOC118264501	298	NC_049715.1	2,819,343	2,822,884	enoyl-CoA hydratase domain-containing protein 2, mitochondrial-like	XP_047024252.1	*Helicoverpa zea*	5 × 10^−180^	89.23
Hydroxyacyl-coenzyme A (HCD) (β-oxidation enzyme)										
*SfruHCD1*	LOC118272082	313	NC_049725.1	1,232,063	1,234,251	hydroxyacyl-coenzyme A dehydrogenase, mitochondrial-like	XP_035451823.1	*Spodoptera frugiperda*	0.0	100
*SfruHCD2*	LOC118272083	307	NC_049725.1	1,235,137	1,239,192	putative 3-hydroxyacyl-CoA dehydrogenase	AID66695.1	*Agrotis segetum*	0.0	94.14
*SfruHCD3*	LOC118272085	80	NC_049725.1	1,234,695	1,242,746	hydroxyacyl-coenzyme A dehydrogenase, mitochondrial-like	XP_022823313.1	*Spodoptera litura*	3 × 10^−49^	100
3-ketoacyl-CoA thiolase (KCT) (β-oxidation enzyme)										
*SfruKCT1*	LOC118266029	396	NC_049717.1	10,904,839	10,911,237	3-ketoacyl-CoA thiolase, mitochondrial-like	XP_035434809.1	*Spodoptera frugiperda*	0.0	100
*SfruKCT2*	LOC118266244	400	NC_049717.1	12,544,565	12,548,651	3-ketoacyl-CoA thiolase, mitochondrial-like	XP_035435538.1	*Spodoptera frugiperda*	0.0	100
*SfruKCT3*	LOC118266444	398	NC_049717.1	12,548,954	12,551,900	3-ketoacyl-CoA thiolase, mitochondrial-like	XP_035435800.1	*Spodoptera frugiperda*	0.0	100
*SfruKCT4*	LOC118266245	394	NC_049717.1	12,538,512	12,544,434	3-ketoacyl-CoA thiolase, mitochondrial-like	XP_035435539.1	*Spodoptera frugiperda*	0.0	100
*SfruKCT5*	LOC118266304	396	NC_049717.1	12,535,796	12,538,103	3-ketoacyl-CoA thiolase, mitochondrial-like	XP_035435600.1	*Spodoptera frugiperda*	0.0	100
*SfruKCT6*	LOC118265763	396	NC_049717.1	12,527,729	12,534,164	3-ketoacyl-CoA thiolase, mitochondrial-like	XP_035434809.1	*Spodoptera frugiperda*	0.0	100
Desaturase (DES)										
*SfruDES1*	LOC118269923	379	NC_049722.1	4,551,252	4,554,527	acyl-CoA desaturase 1-like	XP_035441201.1	*Spodoptera frugiperda*	0.0	100
*SfruDES2*	LOC118264925	375	NC_049716.1	12,167,231	12,217,589	desaturase	AAQ74260.1	*Spodoptera littoralis*	0.0	96.54
*SfruDES3*	LOC118268438	444	NC_049720.1	13,813,813	13,831,130	desaturase	ARD71179.1	*Spodoptera exigua*	0.0	91.22
*SfruDES4*	LOC118274250	336	NC_049711.1	11,647,117	11,651,688	desaturase	ARD71180.1	*Spodoptera exigua*	0.0	98.21
*SfruDES5*	LOC118276125	339	NC_049731.1	8,780,609	8,784,814	delta 11 desaturase	AGH12217.1	*Spodoptera litura*	0.0	91.15
*SfruDES6*	LOC118268131	322	NC_049720.1	12,847,464	12,851,906	acyl-CoA Delta(11) desaturase-like	XP_035438325.1	*Spodoptera frugiperda*	0.0	100
*SfruDES7*	LOC118264926	371	NC_049716.1	12,252,078	12,286,714	stearoyl-CoA desaturase 5-like	XP_035433487.1	*Spodoptera frugiperda*	0.0	100
*SfruDES8*	LOC118280447	321	NC_049738.1	9,365,125	9,401,887	putative desaturase des8	ALJ30231.1	*Spodoptera litura*	0.0	97.51
*SfruDES9*	LOC118264929	354	NC_049716.1	12,362,286	12,378,777	desaturase	ARD71183.1	*Spodoptera exigua*	0.0	98.02
*SfruDES10*	LOC118268107	390	NC_049720.1	14,173,733	14,179,282	desaturase	ARD71184.1	*Spodoptera exigua*	2 × 10^−151^	95.43
*SfruDES11*	LOC118264952	377	NC_049716.1	10,539,212	10,545,018	acyl-CoA Delta(11) desaturase-like	XP_035433515.1	*Spodoptera frugiperda*	0.0	100
*SfruDES12*	LOC118264931	358	NC_049716.1	12,104,709	12,122,940	desaturase	ARD71185.1	*Spodoptera exigua*	0.0	92.12
*SfruDES13*	LOC118264927	369	NC_049716.1	12,146,558	12,164,738	acyl-CoA Delta(11) desaturase-like	XP_035433488.1	*Spodoptera frugiperda*	0.0	100
*SfruDES14*	LOC118264933	340	NC_049716.1	12,346,231	12,349,171	acyl-CoA Delta(11) desaturase-like	XP_035433497.1	*Spodoptera frugiperda*	0.0	100
*SfruDES15*	LOC118268104	453	NC_049720.1	14,186,781	14,189,958	Desaturase	KOB71313.1	*Operophtera brumata*	0.0	64.52
*SfruDES16*	LOC118269215	360	NC_049721.1	3,116,248	3,129,873	acyl-CoA Delta(11) desaturase-like	XP_035440106.1	*Spodoptera frugiperda*	0.0	100
*SfruDES17*	LOC118269922	396	NC_049722.1	4,554,971	4,557,910	acyl-CoA Delta(11) desaturase-like	XP_035441200.1	*Spodoptera frugiperda*	0.0	100
Fatty acyl reductase (FAR)										
*SfruFAR1*	LOC118280370	535	NC_049738.1	7,174,208	7,214,859	fatty acyl reductase	ARD71186.1	*Spodoptera exigua*	0.0	96.07
*SfruFAR2*	LOC118265382	462	NC_049716.1	5,347,090	5,355,248	fatty acyl reductase	ARD71187.1	*Spodoptera exigua*	0.0	82.93
*SfruFAR3*	LOC118265592	454	NC_049716.1	1,589,115	1,598,988	putative fatty acyl reductase FAR3	ALJ30237.1	*Spodoptera litura*	0.0	93.83
*SfruFAR4*	LOC118280550	498	NC_049738.1	7,051,936	7,079,859	fatty acyl-CoA reductase 13	AKD01774.1	*Helicoverpa armigera*	0.0	88.76
*SfruFAR5*	LOC118280458	525	NC_049738.1	7,234,148	7,242,711	putative fatty acyl reductase FAR5	ALJ30239.1	*Spodoptera litura*	0.0	94.26
*SfruFAR6*	LOC118280491	520	NC_049738.1	1,004,955	1,024,481	fatty acyl reductase	ARD71191.1	*Spodoptera exigua*	0.0	85.38
*SfruFAR7*	LOC118263342	520	NC_049710.1	14,027,024	14,057,897	fatty acyl reductase	ARD71192.1	*Spodoptera exigua*	0.0	89.62
*SfruFAR8*	LOC118280282	526	NC_049738.1	7,122,361	7,154,556	fatty acyl-CoA reductase 1-like	XP_035456141.1	*Spodoptera frugiperda*	0.0	100
*SfruFAR9*	LOC118265401	490	NC_049716.1	1,634,217	1,641,393	fatty acyl-CoA reductase 1-like	XP_035434130.1	*Spodoptera frugiperda*	0.0	100
*SfruFAR10*	LOC118280227	624	NC_049738.1	10,266,635	10,318,251	putative fatty acyl reductase FAR10	ALJ30244.1	*Spodoptera litura*	0.0	95.83
*SfruFAR11*	LOC118280439	510	NC_049738.1	8,122,362	8,131,120	fatty acyl-CoA reductase 2	AKD01763.1	*Helicoverpa armigera*	0.0	56.24
*SfruFAR12*	LOC118280219	523	NC_049738.1	5,814,548	5,844,700	fatty acyl-CoA reductase 1-like	XP_047038603.1	*Helicoverpa zea*	0.0	68.84
*SfruFAR13*	LOC118280549	520	NC_049738.1	7,017,686	7,037,406	putative fatty acyl-CoA reductase CG5065	XP_035456531.1	*Spodoptera frugiperda*	0.0	100
*SfruFAR14*	LOC118280222	528	NC_049738.1	5,956,001	6,001,728	fatty acyl-CoA reductase 8	QLI61998.1	*Streltzoviella insularis*	0.0	86.39
*SfruFAR15*	LOC118280297	512	NC_049738.1	5,092,043	5,105,984	fatty acyl reductase 15	ATJ44470.1	*Helicoverpa armigera*	0.0	78.52
*SfruFAR16*	LOC118280293	531	NC_049738.1	7,103,707	7,121,330	fatty acyl reductase 16	ATJ44527.1	*Helicoverpa assulta*	0.0	89.29
*SfruFAR17*	LOC118265535	450	NC_049716.1	8,947,008	8,959,114	putative fatty acyl-CoA reductase CG5065	XP_035434352.1	*Spodoptera frugiperda*	0.0	100
*SfruFAR18*	LOC118280263	511	NC_049738.1	3,127,028	3,152,947	fatty acyl reductase 12	ATJ44526.1	*Helicoverpa assulta*	0.0	75.15
*SfruFAR19*	LOC118280403	513	NC_049738.1	5,124,444	5,137,543	fatty acyl-CoA reductase wat-like	XP_035456297.1	*Spodoptera frugiperda*	0.0	100
*SfruFAR20*	LOC118280192	523	NC_049738.1	7,287,590	7,295,954	fatty acyl-CoA reductase wat-like	XP_035455970.1	*Spodoptera frugiperda*	0.0	100
*SfruFAR21*	LOC118280547	542	NC_049738.1	6,979,095	6,990,649	fatty acyl reductase 13	ATJ44468.1	*Helicoverpa armigera*	0.0	66.73
*SfruFAR22*	LOC118280723	537	NC_049739.1	1,366,062	1,372,472	fatty acyl-CoA reductase wat-like	XP_035456945.1	*Spodoptera frugiperda*	0.0	100
*SfruFAR23*	LOC118267621	535	NC_049719.1	7,599,750	7,608,971	fatty acyl-CoA reductase wat-like	XP_035437588.1	*Spodoptera frugiperda*	0.0	100
*SfruFAR24*	LOC118267780	538	NC_049719.1	7,588,680	7,597,048	fatty acyl-CoA reductase 1-like	XP_035438040.1	*Spodoptera frugiperda*	0.0	69.60
*SfruFAR25*	LOC118267895	548	NC_049719.1	7,574,852	7,582,604	fatty acyl-CoA reductase 1-like	XP_035438040.1	*Spodoptera frugiperda*	0.0	100
*SfruFAR26*	LOC118280456	538	NC_049738.1	7,251,597	7,260,794	fatty acyl-CoA reductase wat-like	XP_035456388.1	*Spodoptera frugiperda*	0.0	100
*SfruFAR27*	LOC118280298	530	NC_049738.1	7,271,670	7,283,596	fatty acyl-CoA reductase wat-like	XP_035456154.1	*Spodoptera frugiperda*	0.0	100
*SfruFAR28*	LOC118280321	539	NC_049738.1	3,362,799	3,380,634	fatty acyl reductase 5	ATJ44463.1	*Helicoverpa armigera*	0.0	88.27
*SfruFAR29*	LOC118280434	538	NC_049738.1	8,055,508	8,085,920	fatty acyl reductase	AID66647.1	*Agrotis segetum*	0.0	80.76
Acetyltransferase (ACT)										
*SfruACT1*	LOC118272936	252	NC_049726.1	7,310,246	7,316,764	N-alpha-acetyltransferase 60-like	XP_035445570.1	*Spodoptera frugiperda*	0.0	100
*SfruACT2*	LOC118267998	176	NC_049719.1	4,426,976	4,431,010	probable N-acetyltransferase san	XP_022826510.1	*Spodoptera litura*	1 × 10^−127^	100
*SfruACT3*	LOC118275415	199	NC_049730.1	1,741,133	1,742,303	N-alpha-acetyltransferase 80-like	XP_035449252.1	*Spodoptera frugiperda*	8 × 10^−143^	100
*SfruACT4*	LOC118281885	107	NW_023337168.1	95,711	96,749	N-alpha-acetyltransferase 38-B	XP_022826548.1	*Spodoptera litura*	8 × 10^−66^	91.35
*SfruACT5*	LOC118272777	710	NC_049711.1	13,544,018	13,554,256	N-alpha-acetyltransferase 35, NatC auxiliary subunit	XP_028155763.1	*Ostrinia furnacalis*	0.0	91.85
*SfruACT6*	LOC118278521	469	NC_049735.1	8,174,301	8,181,092	putative acetyltransferase ACT9	ALJ30256.1	*Spodoptera litura*	6 × 10^−178^	99.25
*SfruACT7*	LOC118281877	294	NW_023337168.1	41,788	43,450	N-alpha-acetyltransferase 30-like	XP_035458539.1	*Spodoptera frugiperda*	0.0	100
*SfruACT8*	LOC118267522	173	NC_049719.1	1,242,030	1,243,082	N-alpha-acetyltransferase 20	XP_022826734.1	*Spodoptera litura*	9 × 10^−127^	100
*SfruACT9*	LOC118272523	180	NC_049726.1	6,776,065	6,778,854	N-alpha-acetyltransferase 10	XP_022830428.1	*Spodoptera litura*	6 × 10^−132^	99.44
*SfruACT10*	LOC118276179	396	NC_049731.1	3,555,474	3,572,627	acetyltransferase	ARD71213.1	*Spodoptera exigua*	0.0	98.49
*SfruACT11*	LOC118276445	512	NC_049731.1	3,602,442	3,605,363	fatty alcohol acetyltransferase	AIN34682.1	*Agrotis segetum*	0.0	88.85
*SfruACT12*	LOC118278776	479	NC_049735.1	4,751,837	4,791,014	acetyltransferase	ARD71206.1	*Spodoptera exigua*	0.0	99.79
*SfruACT13*	LOC118275943	245	NC_049730.1	5,635,234	5,651,704	N-alpha-acetyltransferase 40-like	XP_035449986.1	*Spodoptera frugiperda*	0.0	100
*SfruACT14*	LOC118267183	480	NC_049710.1	16,346,539	16,357,150	acetyltransferase 18	ATJ44585.1	*Helicoverpa assulta*	0.0	77.90
*SfruACT15*	LOC118264603	505	NC_049715.1	8,586,407	8,600,294	acetyltransferase	ARD71205.1	Spodoptera exigua	0.0	90.69
*SfruACT16*	LOC118262813	195	NC_049713.1	14,325,493	14,326,654	N-acetyltransferase 9-like	XP_035430320.1	*Spodoptera frugiperda*	1 × 10^−194^	100
*SfruACT17*	LOC118271269	869	NC_049724.1	4,989,091	5,073,434	N-alpha-acetyltransferase 15	XP_035442996.1	*Spodoptera frugiperda*	0.0	99.88
Acyl-CoA-binding protein (ACBP)										
*SfruACBP1*	LOC118267206	85	NC_049718.1	4,519,313	4,523,840	putative acyl-CoA-binding protein	XP_021185372.1	*Helicoverpa armigera*	2 × 10^−52^	94.12
*SfruACBP2*	LOC118266031	470	NC_049717.1	10,902,558	10,904,528	acyl-CoA-binding domain-containing protein 6-like	XP_035434810.1	*Spodoptera frugiperda*	0.0	100
*SfruACBP3*	LOC118279722	265	NC_049737.1	6,551,474	6,584,040	acyl-CoA-binding domain-containing protein 5-like isoform X4	XP_035455327.1	*Spodoptera frugiperda*	0.0	100

## Data Availability

The transcriptome data that support the findings of this study are openly available in SRA the Genbank SRA database (BioProject ID: PRJNA885519). Other data presented in this study are available in the article and Supplementary Materials.

## Supplementary Materials

The following supporting information can be downloaded at: https://www.mdpi.com/article/10.3390/insects13121078/s1. Figure S1: Hypothetical sex pheromone biosynthesis pathways of *S. frugiperda*; Table S1: Query gene sequences; Table S2: Primers used for RT-PCR and qPCR; Table S3: Sequences for phylogenetic tree; Table S4: Primer amplicon characteristics of 4 genes for qRT-PCR; Table S5: Summary of the transcriptome sequencing data of of *Spodoptera frugiperda*; Table S6: Fragments per kilobase million (FPKM) for the different samples.

## References

[1] Jurenka R. (2017). Regulation of pheromone biosynthesis in moths. Curr. Opin. Insect Sci..

[2] Xing Y., Thanasirungkul W., Aslam A., Niu F., Guo H.-R., Chi D.-F. (2021). Genes involved in the Type I pheromone biosynthesis pathway and chemoreception from the sex pheromone gland transcriptome of *Dioryctria abietella*. Comp. Biochem. Physiol. Part D: Genom. Proteom..

[3] Zhang Y.-N., Xia Y.-H., Zhu J.-Y., Li S.-Y., Dong S.-L. (2014). Putative Pathway of Sex Pheromone Biosynthesis and Degradation by Expression Patterns of Genes Identified from Female Pheromone Gland and Adult Antenna of *Sesamia inferens* (Walker). J. Chem. Ecol..

[4] Zhang Z.-B., Yin N.-N., Long J.-M., Zhang Y.-K., Liu N.-Y., Zhu J.-Y. (2021). Transcriptome analysis of the pheromone glands in *Noorda blitealis* reveals a novel AOX group of the superfamily *Pyraloidea*. J. Asia-Pac. Èntomol..

[5] Ando T., Yamakawa R. (2011). Analyses of lepidopteran sex pheromones by mass spectrometry. Trends Anal. Chem..

[6] Ando T., Inomata S., Yamamoto M. (2004). Lepidopteran Sex Pheromones. Topics in Current Chemistry.

[7] Jurenka R. (2004). Insect Pheromone Biosynthesis. Topics in Current Chemistry.

[8] Zhang Y.-N., Zhu X.-Y., Fang L.-P., He P., Wang Z.-Q., Chen G., Sun L., Ye Z.-F., Deng D.-G., Li J.-B. (2015). Identification and Expression Profiles of Sex Pheromone Biosynthesis and Transport Related Genes in *Spodoptera litura*. PLoS ONE.

[9] Lin X., Wang B., Du Y. (2018). Key genes of the sex pheromone biosynthesis pathway in female moths are required for pheromone quality and possibly mediate olfactory plasticity in conspecific male moths in *Spodoptera litura*. Insect Mol. Biol..

[10] Yang Y.C., Tao J., Zong S.X. (2020). Identification of putative Type-I sex pheromone biosynthesis-related genes expressed in the female pheromone gland of *Streltzoviella insularis*. PLoS ONE.

[11] Moto K., Yoshiga T., Yamamoto M., Takahashi S., Okano K., Ando T., Nakata T., Matsumoto S. (2003). Pheromone gland-specific fatty-acyl reductase of the silkmoth, *Bombyx mori*. Proc. Natl. Acad. Sci. USA.

[12] Ohnishi A., Hashimoto K., Imai K., Matsumoto S. (2009). Functional Characterization of the *Bombyx mori* Fatty Acid Transport Protein (BmFATP) within the Silkmoth Pheromone Gland. J. Biol. Chem..

[13] Jing W., Huang C., Li C.Y., Zhou H.X., Ren Y.L., Li Z.Y., Xing L., Zhang B., Qiao X., Liu B. (2021). Biology, invasion and management of the agricultural invader: Fall armyworm, *Spodoptera frugiperda* (Lepidoptera: Noctuidae). J. Integr. Agric..

[14] Suby S.B., Soujanya P.L., Yadava P., Patil J., Subaharan K., Prasad G.S., Babu K.S., Jat S.L., Yathish K.R., Vadassery J. (2020). Invasion of Fall Armyworm (*Spodoptera frugiperda*) in India: Nature, Distribution, Management and Potential Impact. Curr. Sci..

[15] Tepa-Yotto G., Chinwada P., Rwomushana I., Goergen G., Subramanian S. (2022). Integrated management of *Spodoptera frugiperda* 6 d years post detection in Africa: A review. Curr. Opin. Insect Sci..

[16] Jia H.-R., Guo J.-L., Wu Q.-L., Hu C.-X., Li X.-K., Zhou X.-Y., Wu K.-M. (2021). Migration of invasive *Spodoptera frugiperda* (Lepidoptera: Noctuidae) across the Bohai Sea in northern China. J. Integr. Agric..

[17] Wu F.F., Zhang L., Liu Y.Q., Cheng Y.X., Su J.Y., Sappington T.W., Jiang X.F. (2022). Population Development, Fecundity, and Flight of *Spodoptera frugiperda* (Lepidoptera: Noctuidae) Reared on Three Green Manure Crops: Implications for an Ecologically Based Pest Management Approach in China. J. Econ. Èntomol..

[18] Cruz I., de Lourdes M., Figueiredo C., da Silva R.B., da Silva I.F., Paula C.D., Foster J.E. (2012). Using sex pheromone traps in the decision-making process for pesticide application against fall armyworm (*Spodoptera frugiperda* [Smith] [Lepidoptera: Noctuidae]) larvae in maize. Int. J. Pest Manag..

[19] Meagher R.L., Nagoshi R.N., Armstrong J.S., Niogret J., Epsky N.D., Flanders K.L. (2013). Captures and Host Strains of Fall Armyworm (Lepidoptera: Noctuidae) Males in Traps Baited with Different Commercial Pheromone Blends. Fla. Èntomol..

[20] Mitchell E.R., Tumlinson J.H., McNeil J.N. (1985). Field Evaluation of Commercial Pheromone Formulations and Traps Using a More Effective Sex Pheromone Blend for the Fall Armyworm (Lepidoptera: Noctuidae)1. J. Econ. Èntomol..

[21] Rojas J.C., Virgen A., Malo E.A. (2004). Seasonal and nocturnal flight activity of *Spodoptera frugiperda* males (Lepidoptera: Noctuidae) monitored by pheromone traps in the Coast of Chiapas, Mexico. Fla. Èntomol..

[22] Cruz-Esteban S. (2020). Antennal sensitivity to female sex pheromone compounds of *Spodoptera frugiperda* males (Lepidoptera: Noctuidae) and associated field behaviour. Physiol. Èntomol..

[23] Sekul A.A., Sparks A.N. (1967). Sex Pheromone of the Fall Armyworm Moth: Isolation, Identification, and Synthesis. J. Econ. Èntomol..

[24] Tumlinson J.H., Mitchell E.R., Teal P.E.A., Heath R.R., Mengelkoch L.J. (1986). Sex pheromone of fall armyworm, *Spodoptera frugiperda* (J.E. Smith). J. Chem. Ecol..

[25] Batista-Pereira L.G., Stein K., De Paula A.F., Moreira J.A., Cruz I., Figueiredo M.D.L.C., Perri J., Corrêa A.G. (2006). Isolation, Identification, Synthesis, and Field Evaluation of the Sex Pheromone of the Brazilian Population of *Spodoptera frugiperda*. J. Chem. Ecol..

[26] Groot A.T., Marr M., Schöfl G., Lorenz S., Svatos A., Heckel D.G. (2008). Host strain specific sex pheromone variation in *Spodoptera frugiperda*. Front. Zool..

[27] Meagher R.L., Nagoshi R.N. (2013). Attraction of Fall Armyworm Males (Lepidoptera: Noctuidae) to Host Strain Females. Environ. Èntomol..

[28] Jiang N.J., Mo B.T., Guo H., Yang J., Tang R., Wang C.Z. (2021). Revisiting the sex pheromone of the fall armyworm *Spodoptera frugiperda*, a new invasive pest in South China. Insect Sci..

[29] Unbehend M., Hänniger S., Meagher R.L., Heckel D.G., Groot A.T. (2013). Pheromonal Divergence Between Two Strains of *Spodoptera frugiperda*. J. Chem. Ecol..

[30] Zhang Y.-N., Zhang L.-W., Chen D.-S., Sun L., Li Z.-Q., Ye Z.-F., Zheng M.-Y., Li J.-B., Zhu X.-Y. (2017). Molecular identification of differential expression genes associated with sex pheromone biosynthesis in *Spodoptera exigua*. Mol. Genet. Genom..

[31] Li R.-T., Ning C., Huang L.-Q., Dong J.-F., Li X., Wang C.-Z. (2017). Expressional divergences of two desaturase genes determine the opposite ratios of two sex pheromone components in *Helicoverpa armigera* and *Helicoverpa assulta*. Insect Biochem. Mol. Biol..

[32] Ding B.-J., Löfstedt C. (2015). Analysis of the *agrotis segetum* pheromone gland transcriptome in the light of Sex pheromone biosynthesis. BMC Genom..

[33] Gu S.-H., Wu K.-M., Guo Y.-Y., Pickett J.A., Field L.M., Zhou J.-J., Zhang Y.-J. (2013). Identification of genes expressed in the sex pheromone gland of the black cutworm *Agrotis ipsilon* with putative roles in sex pheromone biosynthesis and transport. BMC Genom..

[34] Vogel H., Heidel A.J., Heckel D.G., Groot A.T. (2010). Transcriptome analysis of the sex pheromone gland of the noctuid moth *Heliothis virescens*. BMC Genom..

[35] Dou X.Y., Liu S.J., Ahn S.-J., Choi M.-Y., Jurenka R. (2019). Transcriptional comparison between pheromone gland-ovipositor and tarsi in the corn earworm moth *Helicoverpa zea*. Comp. Biochem. Physiol. Part D: Genom. Proteom..

[36] Liu J.Q., Li S.L., Li W.S., Peng L., Chen Z.W., Xiao Y.D., Gu H.Z., Zhang J.W., Cheng T.C., Goldsmith M.R. (2019). Genome-wide annotation and comparative analysis of cuticular protein genes in the noctuid pest *Spodoptera litura*. Insect Biochem. Mol. Biol..

[37] Zhang J.P., Zhang F., Tay W.T., Robin C., Shi Y., Guan F., Yang Y.H., Wu Y.D. (2022). Population genomics provides insights into lineage divergence and local adaptation within the cotton bollworm. Mol. Ecol. Resour..

[38] Li Z.-Q., Zhang S., Luo J.-Y., Wang C.-Y., Lv L.-M., Dong S.-L., Cui J.-J. (2015). Transcriptome comparison of the sex pheromone glands from two sibling *Helicoverpa* species with opposite sex pheromone components. Sci. Rep..

[39] Wang Q.-H., Gong Q., Fang S.-M., Liu Y.-Q., Zhang Z., Yu Q.-Y. (2020). Identification of genes involved in sex pheromone biosynthesis and metabolic pathway in the Chinese oak silkworm, *Antheraea pernyi*. Int. J. Biol. Macromol..

[40] Radonić A., Thulke S., Mackay I.M., Landt O., Siegert W., Nitsche A. (2004). Guideline to reference gene selection for quantitative real-time PCR. Biochem. Biophys. Res. Commun..

[41] Zhou L., Meng J.Y., Ruan H.Y., Yang C.L., Zhang C.Y. (2021). Expression stability of candidate RT-qPCR housekeeping genes in *Spodoptera frugiperda* (Lepidoptera: Noctuidae). Arch. Insect Biochem. Physiol..

[42] Schmittgen T.D., Livak K.J. (2008). Analyzing real-time PCR data by the comparative *C-*_T_ method. Nat. Protoc..

[43] Tillman J.A., Seybold S.J., Jurenka R.A., Blomquist G.J. (1999). Insect pheromones—An overview of biosynthesis and endocrine regulation. Insect Biochem. Mol. Biol..

[44] Groot A.T., Dekker T., Heckel D.G. (2016). The Genetic Basis of Pheromone Evolution in Moths. Annu. Rev. Èntomol..

[45] Alabaster A., Isoe J., Zhou G.L., Lee A., Murphy A., Day W.A., Miesfeld R.L. (2011). Deficiencies in acetyl-CoA carboxylase and fatty acid synthase 1 differentially affect eggshell formation and blood meal digestion in *Aedes aegypti*. Insect Biochem. Mol. Biol..

[46] Choi M.-Y., Jurenka R.A. (2006). C75, a Fatty Acid Synthase Inhibitor, Inhibits Feeding Activity and Pheromone Production in a Moth, *Helicoverpa zea*. J. Asia-Pac. Èntomol..

[47] Wang J., Song Y., Hwarari D.T., Liang X.-H., Ding J.-H., Yan M.W., Wu F.A., Wang J., Sheng S. (2022). Fatty acid synthases and desaturases are essential for the biosynthesis of alpha-linolenic acid and metamorphosis in a major mulberry pest, *Glyphodes pyloalis* walker (Lepidoptera: Pyralidae). Pest Manag. Sci..

[48] Qian S.G., Fujii T., Ito K., Nakano R., Ishikawa Y. (2011). Cloning and functional characterization of a fatty acid transport protein (FATP) from the pheromone gland of a lichen moth, *Eilema japonica*, which secretes an alkenyl sex pheromone. Insect Biochem. Mol. Biol..

[49] Yu H.-Y., Zhou Z.-F., Jia J.-Q., Gui Z.-Z. (2016). Cloning, expression and functional analysis of a delta 6-desaturase gene from the silkworm, *Bombyx mori* L.. J. Asia-Pacific Èntomol..

[50] Hagström K., Albre J., Tooman L.K., Thirmawithana A.H., Corcoran J., Löfstedt C., Newcomb R.D. (2014). A Novel Fatty Acyl Desaturase from the Pheromone Glands of *Ctenopseustis obliquana* and *C. herana* with Specific Z5-Desaturase Activity on Myristic Acid. J. Chem. Ecol..

[51] Xia Y.-H., Zhang Y.-N., Ding B.-J., Wang H.-L., Löfstedt C. (2019). Multi-Functional Desaturases in Two *Spodoptera* Moths with ∆11 and ∆12 Desaturation Activities. J. Chem. Ecol..

[52] Zhang Y.-N., Zhang X.-Q., Zhu G.H., Zheng M.-Y., Yan Q., Zhu X.-Y., Xu J.-W., Zhang Y.-Y., He P., Sun L. (2019). A Δ9 desaturase (SlitDes11) is associated with the biosynthesis of ester sex pheromone components in *Spodoptera litura*. Pestic. Biochem. Physiol..

[53] Fujii T., Ito K., Tatematsu M., Shimada T., Katsuma S., Ishikawa Y. (2011). Sex pheromone desaturase functioning in a primitive *Ostrinia* moth is cryptically conserved in congeners’ genomes. Proc. Natl. Acad. Sci. USA.

[54] Hagström A.K., Liénard M.A., Groot A.T., Hedenström E., Löfstedt C. (2012). Semi–Selective Fatty Acyl Reductases from Four Heliothine Moths Influence the Specific Pheromone Composition. PLoS ONE.

[55] Jurenka R.A., Roelofs W.L. (1989). Characterization of the acetyltransferase used in pheromone biosynthesis in moths: Specificity for the Z isomer in tortricidae. Insect Biochem..

[56] Fujii T., Ito K., Katsuma S., Nakano R., Shimada T., Ishikawa Y. (2010). Molecular and functional characterization of an acetyl-CoA acetyltransferase from the adzuki bean borer moth *Ostrinia scapulalis* (Lepidoptera: Crambidae). Insect Biochem. Mol. Biol..

