# Supplementary material for: Genome-Wide Identification and Expression Profiling of Candidate Sex Pheromone Biosynthesis Genes in the Fall Armyworm (Spodoptera frugiperda)
Source: Insects. 2022 Nov 23;13(12):1078. doi: 10.3390/insects13121078 (PMC9783692; doi:10.3390/insects13121078)
Supplement: Supplementary file 1 [file insects-13-01078-s001.zip › Table S1-Query gene sequences.pdf]

ACC
>ARD71228.1 acetyl-CoA carboxylase [*Spodoptera exigua*]
MFTVIIVGLIFVYVFLKYFSGIEYSNTEMADQTDQARCSSSESDNFEKIDSDEAGENGGAN
FVVGEEVEQEPVDDGPAHEGRDSFPNAPRPNRPRPLTVAQQLALAEKRSTLRPSMSQGTVI
HSQRFQEKDFTVATPEEFVRRFQGTKPINKVLIANNGIGAVKCMRSVRRWSYEMFKNERA
VRFVVMVTPEDLKANAEYIKMADHYVPVPGGSNNNNYANVELIVDIAIRTQVQAVWAG
WGHASENPKLPELLHRAGVVFIGPPEKAMWALGDKIASSIVAQTAEIPTLPWSGSELKAEY
NSKKIKISSELFARGCVTTPEEGLQAAQKIGFPVMIKASEGGGGKGIRKVENPDDFNSAFR
QVQAEVPGSPIFVMKLAKSARHLEVQLLADQYGNAISLFGRDCSIQRRHQKIIEEAPAAIA
KPDVFIEMEKAAVRLAKMVGYVSAGTVEYLYEPATGAYYFLELNPRLQVEHPCTEMVAD
VNLPAAQLQIAMGLPLYHIKDIRLLYGESPWGLSQIEFDEPKQRPSPWGHVIAARITSENPD
EGFKPSSGTVQELNFRSSKNVWGYFSVAASGGLHEFADSQFGHCFSWGETREQARENLVI
ALKELSIRGDFRTTVEYLITLLETAAFQNNDIDTAWLDALIAERMQSEKPNIMLGVICGSILI
ADAYITANFQEFKSALEKGQIQGSSALSNCVEVELIHSGSKYKVSATKSGPTSYFLAMNGS
FKEMEVHKLTDGGMLLSIDGASFTTYLRDEVDKYRIVIGNQTVVFDKEKDPSKLRAPSAG
KLINTLVEDGGHVDKGQPYAEIEVMKMVMTLAAPESGKVTWILRSGAVLDMGALIGTLE
LDDPSLVTTATPYKGQFPIEDNNQLSEKLNHAHNKYKSVLENTLQGYCLPEPYNTPRLREV
VEKFMQSLRDPSLPLLELQEVLSSTSGRIPIAVEKKVRKLMALYERNITSVLAQFPSQQIAS
VIDHHAASLAKRADRDVFFMSTQALVVLVQRYRNGIRGRMKAAVHDLLKQYYQVESNF
QLGSYDKCVATLRERYKDDMQAVADIIFSHNQVAKKNMLVTLLIDHLWSNEPGLTDELAT
TLNELTSLHRAEHSRVALRARQVLIAAHQPAYELRHNQMESIFLSAVDMYGHDFHPENLQ
KLILSETSIFDILHDFFYHTNAAVCNAALEVYVRRAYTSYDITCLQHLALSGELGVVHFQFI
LPTGHPNRIPISQSEIELASAQDQEGIPAELCTAAMRKCHHRTGALAAFESFDQFVQYSDEL
LDLVHDFASSATVRREDLAALQEGSESRDSTSINVGLDYKPNDPDNEAPLEPIHILMIGVRD
SGENDDSAVSRRFGNFCRAHRHELHQKRIRRITFMLLIKRQFPKFFTFRARNDFTEDTIYRH
LEPASAFQLELYRMRSYELEALPTSNQKMHLYLGKAKVKKGQEVTDFRFFIRSIIRHQDLIT
KEASFEYLQNEGERVLLEAMDELEVAFSHPLAKRTDCNHIFLNFGPTVIMDPAKIEESVLG
MVMRYGPRLWKLRVLQAEIRFTLRIGPGAPTKNVRLCLSNGSGYSLDIYTYEEVSDPKIGV
IMFQSFGPRQGPMHGLPISTPYVTKDYLQQKRFLATSQGTTYVYDIPDMFRQMIERRWRE
CIEEGSVDGPPPDNVMNAVELVIEPDGERRVVEVTRLPGQNNVGMVAWRLTLYTPECPDG
RDIILIANDLTYYMGSFGPQEDWVYYKASAYARELKIPRVYVSVNSGARIGVAEEVKSEFN
VAWIDSERPERGFKYLYLTPESYSKLGPLGSVKTTLIEDEGESRYKITDIIGKEDGLGVECLR
DAGLIAGETAQAYEDIVTISIVTCRAIGIGSYVVRLGHRVIQVESSYIILTGYVALNKVLGRS
VYASNNQLGGQQIMHHNGVSSAVAPTDLEAVRTALRWLSFVPKDKMSLVPIMRPADPVDR
PVEWVPPRAAHDPRLMLAGDAARPGFFDAGSWDEVMQPWAQTVITGRARLGGIPVGVV
AVETRTVEITLPADPANLDSEAKTLQQAGQVWFPDSAYKTAQAINDFSREGLPIMIFANWR
GFSGGQKDMYEQILKFGAEIVRALRGASAPVLVYIPPGAELRGGAWAVVDPSVNNLRME
MYADPEARGGVLEAEAIVEVKFKQRDILKTMHRLDPELQRVGARISELKEQIKEISKGLDR
RGSVDESLIRTDAGRAAETRVRELETELLAAEKTAKAREKELSPIYHQIAVQFAELHDTAER
MLEKGCIFDIIPWRDSRRLFYWRLKRLLRQNEQERRVQEAVKPADRMEQGPAAATLRRWF
TEDRGETQSHQWEHDNEAVCKWLEAQAGDDNSVLERNLKAIHQDALMQAVNNLVLKLT
PSQRGEFIRKLSALEMEQ
>AID66639.1 acetyl-CoA carboxylase [*Agrotis segetum*]
MNSFAEFVNKYFKMMKRRTSKRFVLGETNEQQSFDDGDPTEVLPNLTQKFQMTLSMEDE

ERQHEAEQRQQDGRLLRTPNSGTLQPSMSQGTVIHSQRFQEKDFTVATPEEFVRRFQGTKP
INKVLIANNGIGAVKCMRSVRRWSYEMFKNERAVRFVVMVTPEDLKANAEYIKMADHY
VPVPGGSNNNNYANVELIVDIAIRTQVQAVWAGWGHASENPKLPELLHRAGVVFIGPPEK
AMWALGDKIASSIVAQTAEIPTLPWSGSELKAEYNSKKIKISSELFAKGCVTSPEQGLQAAQ
KIGFPVMIKASEGGGGKGIRKVDNPDEFNSSFRQVQAEVPGSPIFVMKLAKSARHLEVQL
LADQYGNAISLFGRDCSIQRRHQKIIEEAPAAIAKPEVFIEMEKAAVRLAKMVGYVSAGTV
EYLYEPATGAYYFLELNPRLQVEHPCTEMVADVNLPAAQLQIAMGLPLYHIKDIRLLYGES
PWGLSQIEFDEPKQRPSPWGHVIAARITSENPDEGFKPSSGTVQELNFRSSKNVWGYFSVA
ASGGLHEFADSQFGHCFSWGETREQARENLVIALKELSIRGDFRTTVEYLITLLETGAFQN
NDIDTAWLDALIAERMQSEKPDIMLGVICGSILIADAIITANFQEFKSALEKGQIQGSSALSN
CVEVELIHTGSKYKVYATKSGPTSYFLAMNGSFKEMEVHKLTDGGMLLSIDGASFTTYLR
DEVDKYRIVIGNQTVVFEKEKDPSKLRAPSAGKLINTLVDDGGHVDKGQPYAEIEVMKM
VMTLAAPESGKVTWILRSGAVLDMGALIGTLELDDPSLVTTATPYKGQFPIEENPNLSEKL
NHSHQKLRAVLENTLQGYCLPEPYNTPRLREVVEKFMQSLRDPSLPLLELQEVLSSTSGRI
PIAVEKKVRKLMALYERNITSVLAQFPSQQIASVIDHHAASLPKRADRDVFFMSTQALVVL
VQRYRNGIRGRMKAAVHDLLKQYYQVESHFQLGSYDKCVVALRDRYKDDMQMVSNIIF
SHNQVAKKNLLVTLLIDHLWSNEPGLTDELATTLNELTSLHRAEHSRVALRARQVLIAAHQ
PAYELRHNQMESIFLSAVDMYGHDFHPENLQKLILSETSIFDILHDFFYHTNAAVCNAALE
VYVRRAYTSYDITCLQHLALSGELGVVHFQFILPTGHPNRIPISQAEIEMESGTDAEGIPAEL
CTAAMRKCHHRTGALAAFESFDQFVQYSDELLDLVHDFASSATVRREDLAALQEGSESRD
STSINVGMDFKPSDTDIEATLEPIHILMIGVRDSGESDDSAVSRRFGNFCRKHRHELHQKRI
RRITFMLLIKRQFPKFFTYRARNDFTEDTIYRHLEPASAFQLELYRMRSYELEALPTSNQK
MHLYLGKAKVKKGQEVTDYRFFIRSIIRHQDLITKEASFEYLQNEGERVLLEAMDELEVAF
SHPLAKRTDCNHIFLNFGPTVIMDPAKIEESVLGMVMRYGPRLWKLRVLQAEIRFTLRIGP
GPGAPTKNVRLCLSNGSGYSLDVYTYEEISDPKIGVIMFQSFGPRQGPMHGLPISTPYVTK
DYLQQKRFLATSQGTTYVYDIPDMFRQMIERRWRECIEEGSVEGPIPDNVMTSVELVVEP
DGERRIVEVTRLPGQNNVGMVAWRLTLFTPECPDGRDIILIANDLTYFMGSFGPNEDWVY
YKASVYARELKIPRVYVSVNSGARIGVAEEVKSEFNVAWLDSERPSRGFKYLYLTPESYSK
LGPLGSVRTELIEDEGESRYKITDIIGKEDGLGVECLRDAGLIAGETAQAYEDIVTISIVTCR
AIGIGSYVVRLGHRVIQVESSYIILTGYAALNKVLGRAVYASNNQLGGQQVMHHNGVSHA
VAPTDLEAVRTALRWLAFVPKDKMSMVPIMRPWDPIDRPVEWVPPRAAHDPRLMLSGDA
ARAGFFDIGSWDEIMQPWAQTVITGRARLGGIPVGVVAVETRTVELTLPADPANLDSEAKT
LQQAGQVWFPDSAYKTAQAINDFSREGLPIMIFANWRGFSGGQKDMYEQILKFGAEIVRA
LRGATAPVIVYIPPGGELRGGAWAVVDPSVNNLRMEMYADPEARGGVLEAEAIVEVKFKQ
RDILKTMHRLDPELQRVGARIAELKEQIKEISKGLDRRGSVDESLIRTDAGKAAETRVREL
ETELLAAEKTSKAREKELGPIYHQIAVQFAELHDTAERMLEKGCIFDIVPWRDSRRQFYWR
LKRLLRQNEQERRVQEAVRPADKMEQGPAAATLRRWFTEDRGETQSHQWEHDNEAVCK
WLEAQAGDDNSVLERNLRSIHQDALLQAVNNLVVELTPSQRAEFIRKLSALEMEQ
>ALJ30271.1 putative acetyl-CoA carboxylase ACC [*Spodoptera litura*]
MFTVIIIFGFVFVYVFLKFFSGIEHLNTKMADQPDQARCSSSESDNFEKIESEEARESGGAN
FVVGEEVEQEPVDDGPAHEGRDSFPNAPRPNRPRPLTVAQQLALAEKRSTLRPSMSQGTVI
HSQRFQEKDFTVATPEEFVRRFQGTKPINKVLIANNGIGAVKCMRSVRRWSYEMFKNERA
VRFVVMVTPEDLKANAEYIKMADHYVPVPGGSNNNNYANVELIVDIAIRTQVQAVWAG
WGHASENPKLPELLHRAGVVFIGPPEKAMWALGDKIASSIVAQTAEIPTLPWSGSELKAEY

NSKKIKISSELFARGCVTTPEEGLQAAQKIGFPVMIKASEGGGGKGIRKVENPDDFNSAFR
QVQAEVPGSPIFVMKLAKSARHLEVQLLADQYGNAISLFGRDCSIQRRHQKIIEEAPAAIA
KPDVFIEMEKAAVRLAKMVGYVSAGTVEYLYEPATGAYYFLELNPRLQVEHPCTEMVAD
VNLPAAQLQIAMGLPLYHIKDIRLLYGESPWGLSQIEFDEPKQRPSPWGHVIAARITSENPD
EGFKPSSGTVQELNFRSSKNVWGYFSVAASGGLHEFADSQFGHCFSWGETREQARENLVI
ALKELSIRGDFRTTVEYLITLLETGAFQNNDIDTAWLDALIAERMQSEKPDIMLGVICGSILI
ADAYITANFQEFKSALEKGQIQGSSALSNCVEVELIHSGSKYKVSATKSGPTSYFLAMNGS
FKEMEVHKLTDGGMLLSIDGASFTTYLRDEVDKYRIVIGNQTVVFDKEKDPSKLRAPSAG
KLINTLVEDGGHVDKGQPYAEIEVMKMVMTLAAPESGKVTWILRPGAVLDMGAMIGTLE
LDDPSLVTTATPYKGQFPIEDNNQLSEKLNHAHNKYKAVLENTLQGYCLPEPYNTPRLRE
VVEKFMQSLRDPSLPLLELQEVLSSTSGRIPIAVEKKVRKLMALYERNITSVLAQFPSQQIA
SVIDHHAASLAKRADRDVFFMSTQALVVLVQRYRNGIRGRMKAAVHDLLKQYYQVESNF
QLGSYDKCVAALRERHKDDMQAVSNIIFSHNQVAKKNMLVTLLIDHLWSNEPGLTDELAT
TLNELTSLHRAEHSRVALRARQVLIAAHQPAYELRHNQMESIFLSAVDMYGHDFHPENLQ
KLILSETSIFDILHDFFYHTNAAVCNAALEVYVRRAYTSYDITCLQHLALSGELGVVHFQFI
LPTGHPNRIPISQSEIELASAQDQEGIPAELCTAAMRKCHHRTGALAAFESFDQFVQYSDEL
LDLVHDFASSATVRREDLAALQEGSESRDSTSINVGLDYKPNDPDNEAPLEPIHILMIGVRD
SGENDDSAVSRRFGNFCRAHRHELHQKRIRRITFMLLIKRQFPKFFTFRARNDFTEDTIYRH
LEPASAFQLELYRMRSYELEALPTSNQKMHLYLGKAKVKKGQEVTDFRFFIRSIIRHQDLIT
KEASFEYLQNEGERVLLEAMDELEVAFSHPLAKRTDCNHIFLNFGPTVIMDPAKIEESVLG
MVMRYGPRLWKLRVLQAEIRFTLRIGPGAPTKNVRLCLSNGSGYSLDIYTYEEVSDPKIGV
IMFQSFGPRQGPMHGLPISTPYVTKDYLQQKRFLATSQGTTYVYDIPDMFRQMIERRWRE
CIEEGSVDGPPPDNVMNSVELVIEPDGERRVVEVTRLPGQNNVGMVAWRLTLYTPECPDG
RDIILIANDLTYYMGSFGPQEDWVYYKASAYARELKIPRVYVSVNSGARIGVAEEVKSEFN
VAWIDSERPDRGFKYLYLTPESYSKLGPLGSVKTTLIEDEGESRYKITDIIGKEDGLGVECLR
DAGLIAGETAQAYEDIVTISIVTCRAIGIGSYVVRLGHRVIQVESSYIILTGYVALNKVLGRP
VYASNNQLGGQQVMHHNGVSHAVAPTDLEAVRTALRWLSFVPKDKMSLVPIMRPADPID
RPVEWVPPRAAHDPRLMLAGDAARGGFFDAGSWDEVMQPWAQTVITGRARLGGIPVGV
VAVETRTVELTLPADPANLDSEAKTLQQAGQVWFPDSAYKTAQAINDFSREGLPIIIFANWR
GFSGGQKDMYEQILKFGAEIVRALRGATAPVLVYIPPGAELRGGAWAVVDPSVNNLRME
MYADPEARGGVLEAEAIVEVKFKQRDILKTMHRLDPELQRVGARISELKEQIKEISKGLDR
RGSVDESLIRTDAGRAAESRVRELETELLAAEKTAKAREKELSPIYHQIAVQFAELHDTAER
MLEKGCIFDIIPWRESRRLLYWRLKRLLRQNEQERRVQHAVQPADCMQQGPAAATLRRWF
TEDRGETQSHQWEHDNEAVCKWLEAQAGDDNSVLERNLRAIHQDALMQAVNNLVLKLT
PSQRGEFIRKLSALEMEH
>ACX53705.1 acetyl-CoA carboxylase, partial [*Heliothis virescens*]
MNFLESVCGFVNIFLKMLKRRTSKRFVLGENAEPQSFDDEEPTEVLPNLAQKFQMTLSVD
PEEREGHDEGQRQPDGRLLRPPNSGTLQPSMSQGTVIHSQRFQEKDFTVATPEEFVRRFQG
TKPINKVLIANNGIGAVKCMRSIRRWSYEMFKNERAVRFVVMVTPEDLKANAEYIKMAD
HYVPVPGGSNNNNYANVELIVDIAIRTQVQAVWAGWGHASENPKLPELLHRAGVVFIGPP
EKAMWALGDKIASSIVAQTAEIPTLPWSGSELKAEYNSKKIKISSELFAKGCVTTPEQGLQA
AQKIGFPVMIKASEGGGGKGIRKVDNPDDFNSMFRQVQAEVPGSPIFVMKLAKSARHLE
VQLLADQYGNAISLFGRDCSIQRRH
>AGR49308.1 acetyl-coA carboxylase [*Agrotis ipsilon*]

MVGYVSAGTVEYLYEPATGAYYFLELNPRLQVEHPCTEMVADVNLPAAQLQIAMGLPLY
HIKDIRLLYGESPWGLSQIEFDEPKQRPSPWGHVIAARITSENPDEGFKPSSGTVQELNFRSS
KNVWGYFSVAASGGLHEFADSQFGHCFSWGETREQARENLVIALKELSIRGDFRTTVEYLI
TLLETGAFQNNDIDTAWLDALIAERMQSEKPDIMLGVICGSILIADAIITANFQEFKSALEK
GQIQGSSALSNCVEVELIHTGSKYKVYATKSGPTSYFLAMNGSFKEMEVHKLTDGGMLLS
IDGASFTTYLRDEVDKYRIVIGNQTVVFEKEKDPSKLRAPSAGKLINTLVDDGGHVDKGQ
PYAEIEVMKMVMTLAAPESGKVTWILRSGAVLDMGALIGTLELDDPSLVTTATPYKGQFPI
EENPNLSEKLNHSHQKLRAVLENTLQGYCLPEPYNTPRLREVVEKFMQSLRDPSLPLLELQ
EVLSSTSGRIPIAVEKKVRKLMALYERNITSVLAQFPSQQIASVIDHHAASLQKRADRDVFF
MSTQALVVLVQRYRNGIRGRMKAAVHDLLKQYYQVESHFQLGSYDKCVVTLRDRYKDD
MQMVSNIIFSHNQVAKKNLLVTLLIDHLWSNEPGLTDELATTLNELTSLHRAEHSRVALRA
RQVLIAAHQPAYELRHNQMESIFLSAVDMYGHDFHPENLQKLILSETSIFDILHDFFYHTNA
AVCNAALEVYVRRAYTSYDITCLQHLALSGELGVVHFQFILPTGHPNRIPISQAEIEMESGT
DAEGIPAELCTAAMRKCHHRTGALAAFESFDQFVQYSDELLDLVHDFASSASVRKEDLAA
LQEGSESRDSTSINVGMDFKPSDTDNEAPLEPIHILMIGVRDSGESDDSAVSRRFGNFCRKH
RHELHQKRIRRITFMLLIKRQFPKFFTYRARNDFSEDTIYRHLEPASAFQLELYRMRSYELE
ALPTSNQKMHLYLGKAKVKKGQEVTDYRFFIRSIIRHQDLITKEASFEYLQNEGERVLLEA
MDELEVAFSHPLAKRTDCNHIFLNFGPTVIMDPAKIEESVLGMVMRYGPRLWKLRVLQAE
IRFTLRIGPGPGAPTKNVRLCLSNGSGYSLDVYTYEEISDPKIGVIMFQSFGPRQGPMHGLP
ISTPYVTKDYLQQKRFLATSQGTTYVYDIPDMFRQMIERKWRECIEEGSVEGPIPDNVMTS
VELVVEPDGERRIVEVTRLPGQNNVGMVAWRLTLFTPECPDGRDIILIANDLTYFMGSFGP
NEDWVYYKASVYARELKIPRVYISVNSGARIGVAEEVKSEFNVAWLDSERPSRGFKYLYLT
PESYSKLGALGSVKTELIEDEGESRYKITDIIGKEDGLGVECLRDAGLIAGETAQAYEDIVTI
SIVTCRAIGIGSYVVRLGHRVIQVESSYIILTGYAALNKVLGRAVYASNNQLGGQQVMHHN
GVSHAVAPTDLDAVRTALRWLAFVPKDKMSMVPIMRPWDPIDRPVEWVPPRAAHDPRLM
LSGDAARAGFFDVGSWDEIMQPWAQTVITGRARLGGIPVGVVAVETRTVELTLPADPANL
DSEAKTLQQAGQVWFPDSAYKTAQAINDFSREGLPIMIFANWRGFSGGQKDMYEQILKFG
AEIVRALRGATAPVIVYIPPGGELRGGAWAVVDPSVNSLRMEMYADPEARGGVLEAEAIV
EVKFKQRDILKTMHRLDPELQRVGARIAEIKEQIKEISKGLDRRGSVDESLIRTDAGKAAE
TRVRELETELLAAEKTAKAREKELSPIYHQIAVQFAELHDTAERMLEKGCIFDIVPWRDSR
RQFYWRLKRLLRQNEQERRVQEAVRPADKMEQGPAAATLRRWFTEDRGETQSHQWEHD
NEAVCKWLEAQAGDDNSVLERNLRSIHQDALLQAVNNLVVELTPSQRAEFIRKLSALEME
Q
>AGR49309.1 acetyl-coA carboxylase, partial [*Agrotis ipsilon*]
CDYFSDILFYVDGYIYYRICLVFIVIKLLEWAEGFKEVKMAEQQDQARCSSSESDNFEKIDS
EEAKENGAAANFVIGEEVEQEPVDEPAHEGRDSFPGAPIPRPNRPRPMTVAQQLALAEKRS
TLRPSMSQGTVIHSQRFQEKDFTVATPEEFVRRFQGTKPINKVLIANNGIGAVKCMRSVRR
WSYEMFKNERAVRFVVMVTPEI
>ALS92678.1 acetyl-CoA carboxylase [*Helicoverpa armigera*]
MNIFESVCGIANVFFKMLKRRTSKRFVLGENAEPQSFDDEEPTEVLPNLAQKFQMTLSVD
PEEREGHEEGQRQQDGRLLRPPNSGTLQPSMSQGTVIHSQRFQEKDFTVATPEEFVRRFQG
TKPINKVLIANNGIGAVKCMRSIRRWSYEMFKNERAVRFVVMVTPEDLKANAEYIKMAD
HYVPVPGGSNNNNYANVELIVDIAIRTQVQAVWAGWGHASENPKLPELLHRAGVVFIGPP
EKAMWALGDKIASSIVAQTAEIPTLPWSGSELKAEYNSKKIKISSELFARGCVTTPEQGLQA

AQKIGFPVMIKASEGGGGKGIRKVDNPDDFNSMFRQVQAEVPGSPIFVMKLAKSARHLE
VQLLADQYGNAISLFGRDCSIQRRHQKIIEEAPAAIAKPDVFIEMEKAAVRLAKMVGYVSA
GTVEYLYEPATGAYYFLELNPRLQVEHPCTEMVADVNLPAAQLQIAMGLPLYHIKDIRLLY
GESPWGLSQIEFDEPKQRPSPWGHVIAARITSENPDEGFKPSSGTVQELNFRSSKNVWGYF
SVAASGGLHEFADSQFGHCFSWGETREQARENLVIALKELSIRGDFRTTVEYLITLLETGAF
QNNDIDTAWLDALIAERMQSEKPDIMLGVICGSILIADAYITANFQEFKSALEKGQIQGSSA
LSNCVEVELIHSGSKYKVSATKSGPTSYFLAMNGSFKEMEVHKLTDGGMLLSIDGASYTT
YLRDEVDKYRIVIGNQTVVFEKEKDPSKLRAPSAGKLINTLVEDGGHVDKGQPYAEIEVM
KMVMTLAAPESGKVTWILRSGAVLDMGAMIGTLELDDPSLVTTAVPYKGQFPIEDNQNLS
EKLNHAHNKYRAVLENTLQGYCLPEPYNTPRLREVVEKFMQSLRDPSLPLLELQEVLSST
SGRIPIAVEKKVRKLMALYERNITSVLAQFPSQQIASVIDHHAASLAKRADRDVFFMSTQA
LVVLVQRYRNGIRGRMKAAVHDLLKQYYQVESNFQLGSYDKCVVALRDRHKDDMQAVS
NIIFSHNQVAKKNLLVTLLIDHLWSNEPGLTDELATTLNELTSLHRAEHSRVALRARQVLIA
AHQPAYELRHNQMESIFLSAVDMYGHDFHPENLQKLILSETSIFDILHDFFYHTNAAVCNA
ALEVYYVRRAYTSYDITCLQHLALSGELGVVHFQFILPTGHPNRIPISQSEIELASASDQEGIP
AELCTAAMRKCHHRTGALAAFESFDQFVQYSDELLDLVHDFASSATVRREDLAALQEGSE
SRDSTSINVGSDFKPADADNEAPLEPIHILMIGVRDSGESDDSAVSRRFGNFCRAHRHELH
QKRVRRITFMLLIKRQFPKFFTFRARNDFTEDTIYRHLEPASAFQLELYRMRSYELEALPTS
NQKMHLYLGKAKVKKGQEVTDYRFFIRSIIRHQDLITKEASFEYLQNEGERVLLEAMDEL
EVAFSHPLAKRTDCNHIFLNFGPTVIMDPAKIEESVLGMVMRYGPRLWKLRVLQAEIRFTL
RIGPGAPTKNVRLCLSNGSGYSLDIYTYEEVSDPKIGVIMFQSFGPRQGPMHGLPISTPYVT
KDYLQQKRFLATSQGTTYVVYDIPDMFRQMVERRWRECIEEGSVDGPPPDNVMTSVELVV
EADGERRVVEVTRLPGQNNVGMVAWRLTLFTPECPDGRDIILIANDLTYYMGSFGPQEDW
VYYKASVYARELKIPRVYISVNSGARIGVAEEVKSEFNVAWLDSERPDRGFKYLYLTPESY
SKLGPLGSVKTTLIEDEGESRYKITDIIGKEDGLGVECLRDAGLIAGETAQAYEDIVTISIVT
CRAIGIGSYVVRLGHRVIQVESSYIILTGYAALNKVLGRAVYASNNQLGGQQVMHHNGVS
HAVAPTDLEAVRTALRWLSFVPKDKLSMVPIMRPSDPIDRPVEWAPPRAAHDPRLMLAGD
AARAGFFDVGSWDEIMQPWAQTVITGRARLGGIPVGVVAVETRTVELTLPADPANLDSEA
KTLQQAGQVWFPDSAYKTAQAINDFSRENLPIIIFANWRGFSGGQKDMYEQILKFGAEIVR
ALRGATAPVLVYIPPGAELRGGAWAVVDPSVNSLRMEMYADPDARGGVLEAEAIVEVKF
KQRDILKTMHRLDPELQRIGARISELKEQEQIKDKEISKSLDRRGSIDESLIRTDTGRAAETR
VRELETELLAAEKTSKAREKELSPIYHQIAVQFAELHDTAERMLEKGCIFDIVPWRSSRKQL
YWRLRRLLRQNEQERRVQAAARPGPAMQQGPAAATLRRWFTEDRGETQSHQWEHDNEA
VCRWLEAQAGDDNSVLERNLRAIHQDALLQAVNDLVLELTPSQRSEFIRKLSALEMEQ
>AOD74995.1 acetyl-CoA carboxylase 2 [*Helicoverpa armigera*]
MSEQQDQARCSSSESDNFEKIDSEEARENGGAANFVVGEEEQEHPDDAPAHEGRDSFPNA
PRPNRPRPLTVAQQLALAEKRSTLRPSMSQGTVIHSQRFQEKDFTVATPEEFVRRFQGTKPI
NKVLIANNGIGAVKCMRSIRRWSYEMFKNERAVRFVVMVTPEDLKANAEYIKMADHYVP
VPGGSNNNNYANVELIVDIAIRTQVQAVWAGWGHASENPKLPELLHRAGVVFIGPPEKAM
WALGDKIASSIVAQTAEIPTLPWSGSELKAEYNSKKIKISSELFARGCVTTPEQGLQAAQKI
GFPVMIKASEGGGGKGIRKVDNPDDFNSMFRQVQAEVPGSPIFVMKLAKSARHLEVQLL
ADQYGNAISLFGRDCSIQRRHQKIIEEAPAAIAKPDVFIEMEKAAVRLAKMVGYVSAGTVE
YLYEPATGAYYFLELNPRLQVEHPCTEMVADVNLPAAQLQIAMGLPLYHIKDIRLLYGESP
WGLSQIEFDEPKQRPSPWGHVIAARITSENPDEGFKPSSGTVQELNFRSSKNVWGYFSVAA

SGGLHEFADSQFGHCFSWGETREQARENLVIALKELSIRGDFRTTVEYLITLLETGAFQNN
DIDTAWLDALIAERMQSEKPDIMLGVICGSILIADAYITANFQEFKSALEKGQIQGSSALSN
CVEVELIHSGSKYKVSATKSGPTSYFLAMNGSFKEMEVHKLTDGGMLLSIDGASYTTYLR
DEVDKYRIVIGNQTVVFEKEKDPSKLRAPSAGKLINTLVEDGGHVDKGQPYAEIEVMKM
VMTLAAPESGKVTWILRSGAVLDMGAMIGTLELDDPSLVTTAVPYKGQFPIEDNQNLSEK
LNHAHNKYRAVLENTLQGYCLPEPYNTPRLREVVEKFMQSLRDPSLPLLELQEVLSSTSG
RIPIAVEKKVRKLMALYERNITSVLAQFPSQQIASVIDHHAASLAKRADRDVFFMSTQALV
VLVQRYRNGIRGRMKAAVHDLLKQYYQVESNFQLGSYDKCVVALRDRHKDDMQAVSNII
FSHNQVAKKNLLVTLLIDHLWSNEPGLTDELATTLNELTSLHRAEHSRVALRARQVLIAAH
QPAYELRHNQMESIFLSAVDMYGHDFHPENLQKLILSETSIFDILHDFFYHTNAAVCNAAL
EVYVRRAYTSYDITCLQHLALSGELGVVHFQFILPTGHPNRIPISQSEIELASASDQEGIPAE
LCTAAMRKCHHRTGALAAFESFDQFVQYSDELLDLVHDFASSATVRREDLAALQEGSESR
DSTSINVGSDFKPADADNEAPLEPIHILMIGVRDSGESDDSAVSRRFGNFCRAHRHELHQK
RVRRITFMLLIKRQFPKFFTFRARNDFTEDTIYRHLEPASAFQLELYRMRSYELEALPTSNQ
KMHLYLGKAKVKKGQEVTDYRFFIRSIIRHQDLITKEASFEYLQNEGERVLLEAMDELEV
AFSHPLAKRTDCNHIFLNFGPTVIMDPAKIEESVLGMVMRYGPRLWKLRVLQAEIRFTLRI
GPGAPTKNVRLCLSNGSGYSLDIYTYEEVSDPKIGVIMFQSFGPRQGPMHGLPISTPYVTK
DYLQQKRFLATSQGTTYVYDIPDMFRQMVERRWRECIEEGSVDGPPPDNVMTSVELVVE
ADGERRVVEVTRLPGQNNVGMVAWRLTLFTPECPDGRDIILIANDLTYYMGSFGPQEDWV
YYKASVYARELKIPRVYISVNSGARIGVAEEVKSEFNVAWLDSERPDRGFKYLYLTPESYS
KLGPLGSVKTTLIEDEGESRYKITDIIGKEDGLGVECLRDAGLIAGETAQAYEDIVTISIVTC
RAIGIGSYVVRLGHRVIQVESSYIILTGYAALNKVLGRAVYASNNQLGGQQVMHHNGVSH
AVAPTDLEAVRTALRWLSFVPKDKLSMVPIMRPSDPIDRPVEWAPPRAAHDPRLMLAGDA
ARAGFFDVGSWDEIMQPWAQTVITGRARLGGIPVGVVAVETRTVELTLPADPANLDSEAK
TLQQAGQVWFPDSAYKTAQAINDFSRENLPIIIFANWRGFSGGQKDMYEQILKFGAEIVRA
LRGATAPVLVYIPPGAELRGGAWAVVDPSVNSLRMEMYADPDARGGVLEAEAIVEVKFK
QRDILKTMHRLDPELQRIGARISELKEQIKEISKSLDRRGSIDESLIRTDTGRAAETRVRELE
TELLAAEKTSKAREKELSPIYHQIAVQFAELHDTAERMLEKGCIFDIVPWRSSRKQLYWRL
RRLLRQNEQERRVQAAARPGPAMQQGPAAATLRRWFTEDRGETQSHQWEHDNEAVCRW
LEAQAGDDNSVLERNLRAIHQDALLQAVNDLVLELTPSQRSEFIRKLSALEMEQ

> acetyl-CoA carboxylase [*Helicoverpa zea*]

QSFDDEEPTEVLPNLAQKFQMTLSVDPEEREGHEEGQRQQDGRLLRPPNSGTLQPSMSQG
TVIHSQRFQEKDFTVATPEEFVRRFQGTKPINKVLIANNGIGAVKCMRSIRRWSYEMFKNE
RAVRFVVMVTPEDLKANAEYIKMADHYVPVPGGSNNNNYANVELIVDIAIRTQVQAVWA
GWGHASENPKLPELLHRAGVVFIGPPEKAMWALGDKIASSIVAQTAEIPTLPWSGSELKAE
YNSKKIKISSELFARGCVTTPEQGLQAAQKIGFPVMIKASEGGGGKGIRKVDNPDDFNSMF
RQVQAEVPGSPIFVMKLAKSARHLEVQLLADQYGNAISLFGRDCSIQRRHQKIIEEAPAAI
AKPDVFIEMEKAAVRLAKMVGYVSAGTVEYLYEPATGAYYFLELNPRLQVEHPCTEMVA
DVNLPAAQLQIAMGLPLYHIKDIRLLYGESPWGLSQIEFDEPKQRPSPWGHVIAARITSENP
DEGFKPSSGTVQELNFRSSKNVWGYFSVAASGGLHEFADSQFGHCFSWGETREQARENLV
IALKELSIRGDFRTTVEYLITLLETGAFQNNDIDTAWLDALIAERMQSEKPDIMLGVICGSIL
IADAYITANFQEFKSALEKGQIQGSSALSNCVEVELIHSGSKYKVSATKSGPTSYFLAMNGS
FKEMEVHKLTDGGMLLSIDGASYTTYLRDEVDKYRIVIGNQTVVFEKEKDPSKLRAPSAG
KLINTLVEDGGHVDKGQPYAEIEVMKMVMTLAAPESGKVTWILRSGAVLDMGAMIGTLE

LDDPSLVTTAVPYKGQFPIEDNQNLSEKLNHAHNKYRAVLENTLQGYCLPEPYNTPRLRE
VVEKFMQSLRDPSLPLLELQEVLSSTSGRIPIAVEKKVRKLMALYERNITSVLAQFPSQQIA
SVIDHHAASLAKRADRDVFFMSTQALVVLVQRYRNGIRGRMKAAVHDLLKQYYQVESNF
QLGSYDKCVVALRDRHKDDMQAVSNIIFSHNQVAKKNLLVTLLIDHLWSNEPGLTDELAT
TLNELTSLHRAEHSRVALRARQVLIAAHQPAYELRHNQMESIFLSAVDMYGHDFHPENLQ
KLILSETSIFDILHDFFYHTNAAVCNAALEVYVRRAYTSYDITCLQHLALSGELGVVHFQFI
LPTGHPNRIPISQSEIELASASDQEGIPAELCTAAMRKCHHRTGALAAFESFDQFVQYSDEL
LDLVHDFASSATVRREDLAALQEGSESRDSTSINVGSDFKPADADNEAPLEPIHILMIGVRD
SGESDDSAVSRRFGNFCRAHRHELHQKRVRRITFMLLIKRQFPKFFTFRARNDFTEDTIYR
HLEPASAFQLELYRMRSYELEALPTSNQKMHLYLGKAKVKKGQEVTDYRFFIRSIIRHQDL
ITKEASFEYLQNEGERVLLEAMDELEVAFSHPLAKRTDCNHIFLNFGPTVIMDPAKIEESVL
GMVMRYGPRLWKLRVLQAEIRFTLRIGPGAPTKNVRLCLSNGSGYSLDIYTYEEVSDPKIG
VIMFQSFGPRQGPMHGLPISTPYVTKDYLQQKRFLATSQGTTYVYDIPDMFRQMVERRW
RECIEEGSVDGPPPDNVMTSVELVVEADGERRVVEVTRLPGQNNVGMVAWRLTLFTPECP
DGRDIILIANDLTYYMGSFGPQEDWVYYKASVYARELKIPRIYISVNSGARIGVAEEVKSEF
NVAWLDSERPDRGFKYLYLTPESYSKLGPLGSVKTTLIEDEGESRYKITDIIGKEDGLGVEC
LRDAGLIAGETAQAYEDIVTISIVTCRAIGIGSYVVRLGHRVIQVESSYIILTGYAALNKVLG
RAVYASNNQLGGQQVMHHNGVSHAVAPTDLEAVRTALRWLSFVPKDKLSMVPIMRPSDPI
DRPVEWAPPRAAHDPRLMLAGDAARAGFFDVGSWDEIMQPWAQTVITGRARLGGIPVG
VVAVETRTVELTLPADPANLDSEAKTLQQAGQVWFPDSAYKTAQAINDFSRENLPIIIFANW
RGFSGGQKDMYEQILKFGAEIVRALRGATAPVLVYIPPGAELRGGAWAVVDPSVNSLRME
MYADPDARGGVLEAEAIVEVKFKQRDILKTMHRLDPELQRIGARISELKEQIKEISKSLDR
RGSIDESLIRTDTGRAAETRVRELETELLAAEKTSKAREKELSPIYHQIAVQFAELHDTAER
MLEKGCIFDIVPWRSSRKQLYWRLRRLLRQNEQERRVQAAARPGPAMQQGPAAATLRRW
FTEDRGETQSHQWEHDNEAVCRWLEAQAGDDNSVLERNLRAIHQDALLQAVNDLVLELT
PSQRSEFIRKLSALEMEQ

FAS

>AGR49310.1 fatty acid synthase [*Agrotis ipsilon*]
MPSAVTNGARGSEDDIVLTGLSGRLPESDTIEEFAQQLFDGIDLVTADDRRWTPGLHGLPE
RNGKLKDLAHFDATFFGVHAKQAHLMDPQLRLLLELTHETIIDAGINPSELRGSRTGVYV
GVSNSETEEMWTVDPDKINGYALTGCCRAMFPNRISYTFDLKGPSFAVDTACSSSMFALA
QAATAIRSGHCDAAIVAGCNLCLKPANSLNFHRLSMLSPEGRCAAFDASGRGYVRSEAAV
AVLLQRRGAARRVYATLRGLRVNTDGAKDQGITFPSGDMQRRLAEETFAEAKLRPADVAY
VEAHGTGTKVGDPQEVNAIAELFCKGRKGPLLLGSVKSNMGHSEPASGLCSVAKVVVAM
ERGIIPSNLHYKNANPDIPALSDGRIKVVDRNTPWDGGLVAINSFGFGGANAHVIFESEAG
GGAARTPARYAAPRLVLASGRTEEAVQELTQLAAQHREDAGLHALLDAVHRHNIPGHSYR
GFAVLSDPPVQECIEIESGDPRPVWFVFSGMGSQWPGMAKTLMQLPAFAASINRSAAALRP
HKLDLINIITDAPAAAFDDVINSFVSIAAVQVALVDVLRALEIRPDGIVGHSVGEIGCAYADE
TLTAEQAVLAAYWRGRSIVDAKLAPGAMAAVGLSWEQCEARCPPDVVPACHNANDSVTI
SGPVDSLEKFVAELSAEGTFARRVNSSGVAFHSKYIAAAAPLLRRSLEKVITAPKPRTSRWV
SSSLPRDQWNSDLAKLSDANYHVNNLLSPVRFADALREVPARSIVVEVAPHALLQAVLKR
ALPAPAAAHVPLVRRDAACACAHLLAAAGRLYAAGAQPAVGRLYPAVVWPVPRGTPGLA
SRVRWDHRLEWQVAHFGNASRSGENVIEYDVSRNDDSFITGHNIDGRVLFPATGYLTLVW
RTMAKLNNRKPEETPIVMENIQFRRATIVSRDTPVRFLINVLDGTGEFDVCEGGAVVVTGT

VRLADDPAGERLKDLDCTPPRREDGLLPLVTDDIYKELRLRGYNYGGIFRGIRASDPRGTC
GELAWDDNWISFMDTMLQFGIIGVDTRELYLPTRLQRALIDPAAQLAAVAALGEGGTLPV
RMHRDIDVISAGGIEFRGVKTSLAPRRANPQNAPKLEKYVFLPYDNAAVATEDTSRSKRD
ALTVSLQLVLENAGALKLKLAEAALDRPAEALLTPQALAVLESEPQVRVDATLAAGPTPAP
YAAAVKDLGVKVLPKDGKSAPIESDCHLVMAADVLSRHGAATLEHLAAAMADSGMLLL
EEPHKALDDRAAQEMLQRAGLSPVSRQVAASCEYVLLRRAPALPAQHVVVDVADDTSYA
WVDALREALARAEGEDMRVYCVARTPNSGVLGLCTCLRGEAGGRALRCYFLPGAREPFR
PDAAPYAAQVRRDLAVNVLRAGVWGCYRHLPLGDAEAQLQVEHAYVNTLTRGDLSSLR
WIESPLRYARDVPQPARTDLCRVYCAPLNFRDIMLATGKLPPDALPGNLAGQECILGLEFS
GRSSDGKRVMGMVAACGLASTVLADKGFLWEVPAKWSLEEASTVPVAYATAYYALAVRG
RMQRGDSVLVHAGTGGVGQAAVAIALHAGCTVFTTVGTPDKRAFLRERFPTLPPENIGNS
RDTSFEQLIKRRTRGRGVDLVLNSLAADKLQASVRCLAEGGRFLEIGKLDLSNDTALGMS
VFLKNTTFHGILLDALFDADSENSDKAAVVRCVTDGIAAGAVRPLPATVFSDHQLEQAFRY
MATGKHIGKVVLRVRDEEAAGARPASKLVSAIPRTYMHPAKSYVLVGGMGGFGLELAQW
MVKRGCTRLVLNSRSGVRTGYQAWCVRRWREAGVRVLVSTADACSAAGARALLREAAA
LGAVGGVFNLAAVLRDAFLDKQTPADFQAVAKPKIDATKILDAATRELAPELEYFVVFSSV
SCGRGNPGQSNYGLANSAMERIMEQRQADGLPGLAVQWGAIGEVGLIVETMGGDETVV
GGTVPQRIASCMEALGALLALPHAVAASMVLADKRRSAAAPQQDLLHAVANILGIKDPTK
VSDSANLAELGMDSLMGAEIKQTLERGYDVVLGVQEIRGLTFSKLRGMAGGEDAAAGD
AAPAATESADQVQFAALGELMPKQVLVKLPSAAPAGSELRPVFMVHPIEGVVELLRGVAA
AVRAPVYGLQCTQAAPLEDMAALARHYVTHVRAMQPAAPYTILGYSFGAGVAFEMALQ
LEQAGCETRLVLVDGSPAYVATHTTRGKKKRTTRSAETDEADALAYFVQLFKDVDAAKVS
SELERLPSWEARLAHTTALVGPAAGPHGAEALAAAANSFYRKLVIADTYKPAGRLRAPVT
LFTARDNYVTLGEDYGLREVCAGELHTQQLAGTHRTILAGDAAAAIAQHLSQMLAH
>XP_022831505.1 fatty acid synthase [*Spodoptera litura*]
MAPTTIAEDRLQSGHRLSHPPPGDEVLITGISGYFPDSDSVIHLQENLFNKVDLISGDSRRW
KLGHPEIPQRTGKINNVNKFDASFFGVHYKQAHTMDPMCRILLEKAYEAVIDAGVNPKEL
RDTKTGVFVGACFSESEKTWFYEKMQVNGFGITGCSRAMLANRISYWLGVTGPSYTVDS
ACSSSLYALEHAFRAIRDGHCDAAIVGGSNLCLHPYVSLQFSRLGVLSPDGRCKCFDNSAN
GYARSEAIAVVFLQKAKDSRRVYAQLLHAKTNCDGYKEQGITYPAGHIQKLLLREFYEEC
SIPPSILEFVEAHGTGTRVGDPEELLAIDEIFCTGRKAPLLIGSVKSNLGHSEPASGLCSIAKL
CIAYSTGYIPPNLNYNVPREGVAALAEKRLSVVTEKTPWGRGMSGVNSFGFGGANAHVL
LKNVARAKVNNGIPSDDLPRLACVSGRTESAVARLLDDLESRTVDAELIRLLHSIHDDDIA
GHVVRGFSLLGSTPTKSVSLAREIQYFSGVRRPVWFVYSGMGSQWAGMAKELMRIPVFS
AAIEKCHKALEPKGINLTKIITENDPKIYDNILNSFIGIAAVQIGLTDVLKTIGIEPDYIIGHSV
GELGCAYADGCFTAEEMILSAYSRGLASIETPFIKGSMAAVGLSYTQVKSMCPPEIEVACHN
GPDSSTISGPADIMKNFVAKLSAQGIFAKEVPCSNIAYHSRYIAEAGPTLLKYLKQVIKNPK
ARSEKWVSTSVPQALWKDPKAELSSAEYHTNNLLSPVLFEEAARLIHANAITIEIAPHGLL
QAILRRSLKKDVINVALTQRDHPDNVQVLFTAIGKLYESGLNPHLANIYPHIPFPVSQGTPM
LAHLVEWEHSEDWYVTSYKAQEKMKSGERTVRMSIVDEDSEYMAGHVVDGRNLYPATG
YLVLVWETLGMMMGELYTEVSVVFENVRFQRATNIPKEGSLEFIIMIQKGSGNFEIVESGA
SIVTGRIYAKKNVGQDFRVLPHLPEVTGPSIKHLLTKDFYKELRLRGYQYSGLFRGVLGCN
VEGTRGRLSWVNNWVTFMDCMLQMKIIGQDTRGLFVPTRIEKLSIDANMHYNAISKMNP
DSNKNSFEIRVYPDVDVIRAGGVEVRGLHATPITKRLPLGVPVLEKNEFVSNYGKSKMKM

EDILRANIQLILENIQTYKVKSIELYDDEYKKNDLKPILESVGDMLGDLPLVQAELLLISEEP
VEMPSNITVENRKLAGESNTILFIGANLLGRPELLQNAVGTLRDKAFVISREKERPNPKDYS
DKYDIVTIQDTGFEYIVLLRKRVGARPAKFVRILASDDTFAWIDKVKEEIKEGQKVVLYTQ
DEHINGLLGLVNCLRKEPGGEIVYGLLISDPSAPPFNPDLEFYEEQLDKDLALNVYQDGQ
WGTYRHLLLGDLETVRANHAYVNTLTIGDLSSLRWVEGAIRENHVFKDKEKVLVHVYCA
ALNFRDVMTAIGRVTVDAVARGRLAQECVQGFEIVGKTVNGSRVMGMIRNRGMANLAE
GDRFLLWAIPDEWSFEEAATVPVAYGTVYYAMVMVGQIQRGESILIHAGSGGVGQAAINV
ALHYGCEVFTTVGTAEKRAFIKKLFPQLKDSHIGNSRDTSFEDMIRRETNGKGVDMVLNS
LSDDKLQASVRCLAYRGRFLEIGKFDISNNTPIGMYFFLKETSFHGIMLDYIFDQNQLFRKR
LQDLLLSGIENGAVRPLTYCTFEANDVEHAFRYMAAGKHIGKVIVKIREEERSSRAVMPAP
RPIDAVPRYICHEDHVYVVVGGLGGFGLELADWLIMRGGRKILLTSRRGITNGYQSSRLRA
WASYGADVQISTHDVTTESGCEEMLKMALSMGPVDAIFNLAVILKDSIFQNQTPETFKTSF
APKALATMHLDKLSRKLCPGLKDFVIFSSVSCGRGNAGQTNYGLSNSVMERICEWRKKL
GLPALAVQWGAIGDVGLVADMQDDDVQLEIGGTLQQRISSCLTALDKFMKQDAPIVSSIV
VAEKKAGGSGCGNIVDAVAQIMGIKDLKTVSQQVSLAELGMDSMMAVEIKQTLEREFEIF
LTAQDIRTLTFARLVELTAQREAAASTSASRPANIEGAVGLRVLMRNFGEEITASEPLVYMPS
MVSDGVEGEEAIHVMERVMFMLPGLEGCAAVLEPLCKRLKIKVCVLQLGVEHKNENMD
QMVNRLHQTVISRLAPGAPFWLLGYSYGSLLILELASRLEKEGFKGTVFCLDGAPDFLYAL
LTMTISFKNDFQLQNNLLCHTVDIVAPNNDVTKGLMEKLNEIESYDERVALTIKTSPVQSK
YSDKFIANIASASFDRLKTILDFDPKAFRKLQSPVILLRPKENPSFVAVEENYGLDKYTENN
VTVHFLEGNHVSIIENKDCANIINRVLAENERQDGKTADNVVTSIVENAREVAV
>AID66640.1 fatty acid synthase, partial [*Agrotis segetum*]
MPSAVTNGARGSEDDIVLTGLSGRLPESDTIEEFAQQLFDGVDLVTADDRRWTPGLHGLPE
RNGKLKDLAHFDATFFGVHAKQAHLMDPQLRLLLELTHEAIIDAGINPGELRGSRTGVYV
GVSNSETEEMWTVDPDKINGYALTGCCRAMFPNRISYTFDLKGPSFAVDTACSSSMFALA
QAATAIRSG
>AID66641.1 fatty acid synthase, partial [*Agrotis segetum*]
FPSGDMQRRLAEETFAEAKLRPADVAYVEAHGTGTKVGDPQEVNAIAELFCKGRKGPLL
GSVKSNMGHSEPASGLCSVAKVVVAMERGIIPANLHYKNANPDIPALSDGRIKVVDRNTP
NELMATRPPSHG
>AID66642.1 fatty acid synthase, partial [*Agrotis segetum*]
GGGAARTPARYAAPRLVLASGRTEEAVQELTQLAAQHRDDAGLHALLDAVHRHNIPGHSY
RGFAVLTDPPVQECIEIESGDPRPVWFVFSGMGSQWPGMAKTLMQLPAFAASINRSAAAL
RPHKLDLINIITEAPAAAFDDVINSFVSIAAVQVALVDVLRALEIRPDGIVGHSVGEIGCAYA
DETLTAEQAVLAAYWRGRSIVDAKLAPGAMAAVGLSWEQCEARCPPDLVPACHNANDSV
TISGPVESLEKFVAELSAEGTFARRVNSSGVAFHSKYIAAAAPLLRRSLEKVITSPKPRSSRW
VSSSLPRDQWDSDLAKLSDANYHVNNLLSPVRFADALREVPARSIVVEVAPHALLQAVLK
RALPAPAAA
>AID66643.1 fatty acid synthase, partial [*Agrotis segetum*]
WQVAHFGNASRSGENIIEYDVSRNDDSFITGHNIDGRVLFPATGYLTLVWRTMAKLNNRK
PEETPIVMENIQFRRATIVSRDTPVRFLINVLDGTGEFDVCEGGAVVVTGTVRLAEDPAGE
RLRDLDCTPPRREDGLLPLVTDDIYKELRLRGYNYGGIFRGIRASDPRGTCGELAWDDNW
ISFMDTMLQFGIIGVDTRELYLPTRLQRALIDPAAQLAAVAALGEGGTLPVRMHRDIDVISA
GGIEFRGVKTSLAPRRANPQNAPKLEKYVFLPYDNAAVATEDTSRSKRDALTVSLQLVLEN

AGALKLKLAEAALDRPAEALLTPQALAVLEAEPQVRVDATLAAGPAPAPYAAAVKDLGVK
VLPKDGRSAPIESDCHLVMAADV
>AID66644.1 fatty acid synthase, partial [*Agrotis segetum*]
RAPALPAQHVVVDVADDTSFAWVEALRDALARAEGEDMRVYCVARTPDSGVLGLCTCLG
EAGGRALRCYFLPGAREPFKPDAAPYAAQVRRDLAVNVLRAGVWGCYRHMPLGDAEAQ
LQVEHAYVNTLTRGDLSSLRWIESPLRYAGDVPQPARTDLCRVYCAPLNFRDIMLATGKLP
PDALPGNLAGQECILGLE
>AID66645.1 fatty acid synthase, partial [*Agrotis segetum*]
SGRSSDGKRVMGMVAACGLASTVLADKGFLWEVPAKWSLEEASTVPVAYATAYYALAVR
GRMRRGESVLVHAGTGGVGQAAVAIALHAGCTVFTTVGTPDKRAFLRERFPTLPPENIGN
SRDTSFEQLIKRRTRGRGVDLVLNSLAADKLQASVRCLAEGGRFLEIGKLDLSNDTALGM
SVFLKNTTFHGILLDALFDADSENSDKAAVVRCVTDGIAAGAVRPLPATVFSDHQLEQAFR
YMATGKHIGKVVLRVRDEEAAGARPASKLVSAIPRTYMHPAKSYVLVGGMGGFGLELAQ
WMVKRGCTRLVLNSRSGVRTGYQAWCVRRWREAGVRVVVSTADACSAGGARQLLREA
AALGAVGGVFNLAAVLRDAFLDKQTPADFQAVAKPKIDATKILDAATRELAPELEYFVVFS
SVSCGRGNPGQSNYGLANSAMERIMEQRQADGLPGLAVQWGAIGEVGLIVETMGGDET
VVGGTVPQRIASCMEALGALLALPHAVAASMVLADKRRSAAAPQQDLLHAVANILGIKDP
TKVSDSANLAELGMDSLMGAEIKQTLERGYDVVLGVQEIRALTFSKLRGMAGGEDAAA
GDADAAAPGAESADQVQFAALGELMPKQVLVKLPSAAPAGSELRPVFMVHPIEGVVELL
RGVAAAVRAPVYGLQCTQAAPLDDMAALARHYVTHVRAMQPAAPYTILGYSFGAGVAF
EMALQLEQAGCETRLVLVDGSPAYVATHTTRGKKKRTTRSAETDEADALAYFVQLFKDV
DAAKVSSELERLPSWEARLAHTTALVGPAAGPHGAEALAAAANSFYRKLVIADTYKPAG
RLRAPVTLFTARDNYVTLGEDYGLRDVCAGELHTQQLAGTHRTILAGDAAAAIAQHLSQ
MLAH
>ACX53771.1 fatty acid synthase, partial [*Heliothis virescens*]
GPQAKLCSAEYHTNNLLSPVLFEETSRLIPNNAVLVEVAPHGLLQAILKRSLPSCKNIALTR
RKHADNAFLVLEAIGKLYMEGYNPKVHVLYPEVQLPVSTGTPFLSHLSEMGRMMRNGP
>ACX53772.1 fatty acid synthase, partial [*Heliothis virescens*]
DGCFTAEEMILSAYSRGLASIETPFIKGSMAAVGLGYNQIKSMCPPEIEVACHNGPDSSTISG
PADIMKVFVAKLSSQGIFAKEVPCSNIAYHSRYISQAGPTLLKYLKQVIKDPKPRSEKWVST
SLPQAQWKDAKAALSSAEYHTNNLLSPVLFEETARLIHPNAITIEIAPHGLLQAILRRSLKK
DVINIALTQRNHKDNVQVLFTAFGKLYESGLNPHLANIYPHVPFPVSQGTPMISHLVEWEH
SEDWYVTSYKAQEKMKSGERT
>ACX53774.1 fatty acid synthase, partial [*Heliothis virescens*]
PKKRSEKWISTSVPQNQWNNDEAQYSSAEYHTNNLLNPVLFEESSRLIPENAIVIEVAPHG
LLQAILTRSLAACVHIPLTRRGHEHPVKFLLEAVGKLYLAGLTPKVKSLYPKVEYPVSTETP
LLSHLVEWEHSEEWLKTRYSTKTRVVTAGRDFILSTQDDDYKYFEYYKRDGVCVFPEAA
LLTLVWETYAMYRQSDYRTMSVEFTNVYFYEEVEINDLLKLGV
>AKD01757.1 fatty acid synthase 1, partial [*Helicoverpa armigera*]
MPSAVTNGSRGSDDDIVLTGLSGRLPESDSIEEFAQQLFDGVDLVTADDRRWTPGLHGLPE
RNGKLKDLAHFDATFFGVHAKQAHLMDPQLRLLLELTHETIIDAGINPGELRGSRTGVYV
GVSNSETEEMWTVDPDKINGYALTGCCRAMFPNRISYTFDLKGPSFAVDTACSSSMFALA
QAATAIRAGHCDAAIVAGTNLCLKPANSLNFHRLSMLSPEGRCAAFDASGRGYVRSEAAV
AVLLQRRSAARRVYCTLRGLRVNTDGAKDQGITFPSGDMQRRLAEETFAEAKLRPSDVV

YVEAHGTGTKVGDPQEVNAIAELFCKGRKGPLLLGSVKSNMGHSEPASGLCSVAKVVVA
MERGVIPGNLHYKNANPDIPALSDGRIKVVDRNTEWDGGLVAINSFGFGGANAHVIFESEP
GGGAARTPARYAVPRVVLASGRTEEAVRELTGLAAQHARDAGLHALLDAVHRHNIPGHSH
RGFAVLTDPPIEECAEVESGEPRPVWFVFSGMGSQWPGMAKSLMQLPVFAASVNRSAAAL
RPHNIDLVKIITEAPAAAFDDVINSFVSIAAVQVALVDVLRALEIRPDGIVGHSVGEIGCAYA
DETLTAEQAVLAAYWRGRSIVDAKLPPGAMAAVGLSWEQCEARCPPDVVPACHNANDSV
TISGPVESLEKFVATLSAEGTFARRVNSSGVAFHSKYIAAAAPLLRRSLEKVIPEPKPRSARW
VSSSLPRDKWNSDLAKLSDANYHVNNLLSPVRFADAVREVPERALLVEVAPHALLQAVLK
RARPAPAAHVPLVRRDAPDALAHLLAAAGRLYAAGAQPHVARLYPAVPFPVPRGTPGLAS
RVRWDHALEWSVAHFGSASRSGENVIEYDVSRADDGFITGHNIDGRVLFPATGYLTLVWR
TMAKLHNRKPEETPIVMENIQFRRATIVSRDTPVRFLINVLDGTGEFDVCEGGAVVVTGTV
RLADDPAAERLRDLDTAPPRQEDGLLPLVTDDIYKELRLRGYNYGGIFRGIRSSDPRGTCG
ELAWDDNWISFMDTMLQFGIIGVDTRELYLPTRLQRALIDPAAQLAAVAASGGGTLPVRM
HRDIDVISAGGIEFRGVKTSLAPRRANPQAAPKLEKYVFLPYDNTAVATEDTSRSKRDALT
VSLQLVLENAGALRLKLGEAALERPPEALLTPLAMQLLEAEPQVRVDATLAAGPAPAAYA
AALKDLGVKVLPKDGKAAPVESDCHLVLAADVLSRHGAGVLAQLAAALGEGGMLLLEE
PHKALDAAGARDMLA
>AKD01758.1 fatty acid synthase 2, partial [*Helicoverpa armigera*]
MSIFLKNTTFHGILLDALFDADSADSDKAAVVRCVTDGIASGAVRPLPATVFSDHQLEQAF
RYMATGKHIGKVVLRVREEEASGARPASKLVSAIPRTYMHPARSYVLVGGMGGFGLELAQ
WMVRRGATRLVLNSRSGVRTGYQAWCIRRWREAGVQVCVSTADACSPAGARALLREAA
ALGPVGGVFNLAAVLRDAFLDKQTPADFQAVAKPKIDATKILDAATRELAPELEYFVVFSS
VSCGRGNPGQSNYGLANSAMERIVEQRQADGLPGLAVQWGAIGEVGLIVETMGGDETV
VGGTVPQRIASCMEALGALLALPHAVAASMVLADKRRAAAAPQQDLLHAVANILGIKDPS
KVSDSANLAELGMDSLMGAEIKQTLERGYDVVLGVQEIRALTFAKLRGMAGGDDAAAG
DAAPPAAGPDDQVQFAALGELMPKQALVKLPSAAAAPEQRAVFMVHPIEGVVELLRGVA
AAVRAPVFGLQCTRAAPLDDMAALARHYVAHVRAQQPSPPYTILGYSFGAGVAFEMALQ
LEQAGCETRLVLVDGSPAYVATHTTRGKQKRTTRSAETDEADALAYFVQLFKDVDAAKVS
AELERLPSWEARLARTTQLVGAAAGPHDADALAAAAGSFYRKLVIADTYKPAGRLRAPV
TLFTARDNYVTLGEDYGLREVCAGPLQTQQLAGTHRTILAGDAAAAIAAHLSAMLAQ
>AKD01759.1 fatty acid synthase 3, partial [*Helicoverpa armigera*]
MLAAAGLAPVSRQQAASCEYVLLRRAAAPPAAHVVLEVPDDGSYAWVEALRDALARAE
AEDMRVYCVSRAPASGVLGLCTCLRGEAGGRRLRCYYLPGARDAFRPDAAPYAAQVRR
DLAVNVLRAGVWGSYRHVALGDAAEAQLQVEHAYVNTLTRGDLSSLRWIESPLRHARH
VPQSPRTDLCRVYCAPLNFRDIMLATGKLPPDALPGNLAGQECILGLEFSGRSSDGKRVMG
MVAACGLASTVLADKGFLWEVPAKWSLEEAATVPVAYATAYYALVVRGRMRRGEAVLVH
AGTGGVGQAAVAIALHAGCTVYTTVGTPDKRAFLRERFPTLPPENIGNSRDTSF
>AKD01760.1 fatty acid synthase 1, partial [*Helicoverpa assulta*]
LRGIQHVLLASTVPEQQTSLIQKIVALAGTPIKQSLDKNTTLEELGVFDDKIQEISQYLKLTY
NIVFDENKIPFLTVDTIQQIENSITKPAFKDEKGLSTFFTFVDADELVATTDFVCLPSLVNNSS
MREDEFDATQTYLCIVPGMEGHHERFRLLCERLKLPAIVLQPGLDHLRETMQETAKRFVD
VLLKKTQLQNNFYLLGYETGIAIALEMVALLEDRGLTGTLYCIGFAPDELKVELDEQLSEF
ASEEELQNAVARHMFTLMAGGDARGLGGLQAASTWAQKVELCVRTLLGRVPHSAQ
>AKD01761.1 fatty acid synthase 2, partial [*Helicoverpa assulta*]

MPSAVTNGSRGSDDDIVLTGLSGRLPESDSIEEFAQQLFDGVDLVTADDRRWTPGLHGLPE
RNGKLKDLAHFDATFFGVHAKQAHLMDPQLRLLLELTHETIIDAGINPGELRGSRTGVYV
GVSNSETEEMWTVDPDKINGYALTGCCRAMFPNRISYTFDLKGPSFAVDTACSSSMFALA
QAATAIRAGHCDAAIVAGTNLCLKPANSLNFHRLSMLSPEGRCAAFDASGRGYVRSEAAV
AVLLQRRSAARRVYCTLRGLRVNTDGAKDQGITFPSGDMQRRLAEETFAEAKLRPSDVV
YVEAHGTGTKVGDPQEVNAIAELFCKGRKGPLLLGSVKSNMGHSEPASGLCSVAKVVVA
MERGVIPGNLHYKNANPDIPALSDGRIKVVDRNTEWDGGLVAINSFGFGGANAHVIFESEP
GGGAARTPARYAVPRVVLASGRTEDAVRELTGLAAQHARDAGLHALLDAVHRHNIPGHS
HRGFAVLTDPPIEECAEVESGEPRPVWFVFSGMGSQWPGMAKSLMQLPVFAASVNRSAA
ALRPHNIDLIKIITEAPAAAFDDVINSFVSIAAVQVALVDVLRALEIRPDGIVGHSVGEIGCA
YADETLTAEQAVLAAYWRGRSIVDAKLPPGAMAAVGLSWEQCEARCPPDVVPACHNAND
SVTISGPVESLEKFVATLSAEGTFARRVNSSGVAFHSKYIAAAAPLLRRSLEKVIPDPKPRSA
RWVSSSLPRDKWNSDLAKLSDANYHVNNLLSPVRFADAVREVPERALLVEVAPHALLQA
VLKRARPAPAAHVPLVRRDAPDALVHLLAAAGRLYASGAQPHVARLYPAVAFPVPRGTPG
LASRVRWDHALEWSVAHFGSASRSGENVIEYDVSRADDGFIAGHNIDGRVLFPATGYLTLV
WRTMAKLHNRKPEETPIVMENIQFRRATIVSRDTPVRFLINVLDGTGEFDVCEGGAVVVT
GTVRLADDPAAERLRDLDTAPPRQEDGLLPLVTDDIYKELRLRGYNYGGIFRGIRSSDPRG
TCGELAWDDNWISFMDTMLQFGIIGVDTRELYLPTRLQRALIDPAAQLAAVAASGGGTVP
VRMHRDIDVISAGGIEFRGVKTSLAPRRANPQAAPKLEKYVFLPYDNTAVATEDTSRSKRD
ALTVSLQLVLENAGALRLKLGEAALERPAEALLTPLAMQLLEAEPQVRVDATLAAGPAPA
AYAAALKDLGVKVLPKDGKAVPVESDCHLVLAADVLSRHGAGVLAQLAAALGEGGMLL
LEEPHKALDAAGARDMLAQAGLAPVSRQQAASCEYVLLRRAAAPPAQHVVVEVPDDGS
YAWVEALRDALARAEAEDMRVYCVSRAPASGVLGLCTCLRGEAGGRRLRCYYLPGARD
AFRPDAAPYAAQARRDLAVNVLRAGVWGSYRHVALGDAAEAQLQVEHAYVNTLTRGDL
SSLRWIESPLRHARHVPQSPRTDLCRVYCAPLNFRDIMLATGKLPPDALPGNLAGQECILG
LEFSGRSSDGKRVMGMVAACGLASTVLADKGFLWEVPAKWSLEEAATVPVAYATAYYAL
VVRGRMRRGEAVLVHAGTGGVGQAAVAIALHAGCTVYTTVGTADKRAFLRERFPTLPPE
NIGNSRDTSFEQLIKRRTRGRGVDLVLNSLAADKLHASVRCLAEGGRFLEIGKLDLSNDTA
LGMSIFLKNTTFHGILLDALFDADSADSDKAAVVRCVTDGIASGAVRPLPATVFSDHQLEQ
AFRYMATGKHIGKVVLRVREEEAGGARPASKLVSAIPRTYMHPARSYVLVGGMGGFGLEL
AQWMVRRGATRLVLNSRSGVRTGYQAWCIRRWREAGVQVCVSTADACSPAGARALLRE
AAALGPVGGVFNLAAVLRDAFLDKQTPADFQAVAKPKIDATKILDAATRELAPELEYFVVF
SSVSCGRGNPGQSNYGLANSAMERIVEQRQADGLPGLAVQWGAIGEVGLIVETMGGDET
VVGGTVPQRIASCMEALGALLALPHAVAASMVLADKRRAAAAPQQDLLHAVANILGIKD
PSKVSDSANLAELGMDSLMGAEIKQTLERGYDVVLGVQEIRALTFAKLRGMAGGDDAA
AGDAAPPAAGPDDQVQFAALGELMPKQALVKLPSAAAAPEQRAVFMVHPIEGVVELLRG
VAAAVRAPVFGLQCTRAAPLDDMAALARHYVAHVRAQQPSPPYTILGYSFGAGVAFEMA
LQLEQAGCETRLVLVDGSPAYVATHTTRGKQKRSTRSAETDEADALAYFVQLFKDVDAA
KVSAELERLPSWEARLARTTQLVGAAAGPHDADALAAAAGSFYRKLVIADTYKPAGRVR
APVTLFTARDNYVTLGEDYGLREVCAGPLQTQQLAGTHRTILAGDAAAAIAAHLSAMLA
Q

> fatty acid synthase1 [*Helicoverpa zea*]

GVDLVTADDRRWTPGLHGLPERNGKLKDLAHFDATFFGVHAKQAHLMDPQLRLLLELTH
ETIIDAGINPGELRGSRTGVYVGVSNSETEEMWTVDPDKINGYALTGCCRAMFPNRISYTF

DLKGPSFAVDTACSRSMFALAQAATAIRAGHCAAAIVAGTSLCLKPANSLNFHRLSMLSPE
GRCAAFDASGRGYVRSEAAEIGRAH

> fatty acid synthase 2 [*Helicoverpa zea*]

VRRSKNLTQYEQYKIGTWKKLGADVMVSENNINGNTLVKDASSIGTLGGIYVAITNVSND
KVAELGQLIKSIDSSARSICPHLQYFAILSAIKSLGQDICVDRAKCGFAATHLDLSELYQAQS
KASSHDVVDVAERALRSPSPVVAAVPVPVNEPSLLQQLIALSKIQIPQNVDPEATLKDLGLV
DESIPLICSFLDVVYNVSLDEDSIPDLTLKGIQELVETATDIVPENVNGLATFFSKVSADELIA
TTELFAVPTLNKDITLSEDEFDVNKRYLCIVPGMEGHYERFQVLCERLKLPAFVLQPGYDR
PRETIRETAERYAKILLKKTGIQNNFYLLGYEIGVLVALELTAILEDHGLTGTVFCLGCAPEE
FQATLEEQLSSFKTEEQLQDAIIRHMSKLITDEDVPALDDILSETATWSEKVAVCTRSLLGR
MQHSVQYAQAQIESALGNISRGRAYVAPVRALRSQLVLLRAANCKPAARALQQH

> fatty acid synthase 3 [*Helicoverpa zea*]

NCASGEVRDSAMKCVAIEGYVFDLSEDDMKNNVDFGLSYLLDARHYKSLRPASLFKPEY
ADEKKKLQYQIAEGITKGIVRPLHRVVYSPKDVSRAFRLQSLKLFSGNVLINMTDVQVSD
EVLNVTPRFKYPAEGTYIVVCGDTKFGIEVADRLVKRGVRKLLLHVNPNSLTGYLHIKLTS
WKKLNVSVKISSENLTSDKECINLIKRGTKMAPVFGIFVVQNYSTETKELTLEPENMLQKF
NNDVQVVASLDVSSRKLCNNLKHFVVLNNSSTSASDAYAVEAMEKICEARNDARLPALAF
RSHAVTEFDNNVNNETKTRPQKLSTVMNGLETSLKLNYTNVVTFDLKKQNNYDFLEKVA
KIIGDRKSTRLNSSHSFISYAVFCLKK

FATP

>ARD71229.1 fatty acid transport protein [*Spodoptera exigua*]

MSNVEMSVDSNMNKTDIIQHKFEVKNGNADIEKGAVVKAGPKIPWTKAIIAMFALGVLA
GACAVAWVFQDWQASLLVLAILAIVYCIAFYWRWIYVAIRTAPRDFSALYCYIKILRLTKNF
TKKNWSMPDIFHQMVVKHPNKACFLFEDETWTFRQVEEYSLRVSAVLKGKGVKRGDTV
AVMIGNCPEMPSIWLGATRVGAVCPLINTNQTGNTLLHSINIAKCDVVIYSDEFQTAFQEIS
KELSPSLKLFKFTRRPLNTSPDAVKVVESDNDFTAMLENTAPFAWTPSDSDGFNGKLLYIY
TSGTTGLPKAAVISSSRMVFMASGVHYLGSLRASDVIYCPMPLYHSAGGCITMGQAFIFGC
TVALRTKFSASKYFPDCIKYNATAAHYIGEMCRYVLTTPPSPTDTQHKVRTVYGNGMRPAI
WTDFVKRFNIKKVVEFYGATEGNANIVNINNKTGAIGFVSRIIPAVYPIAILKVDPDSGEPIR
DDRGLCQLAKPNEPGVFIGKINPNNPSRAFLGYVDKAASDKKIVKDVFNYGDSAFISGDIL
TADELGYLYFMDRTGDTFRWRGENVSTTEVESAISRVADQRDAVVYGVEIPNTDGRAGM
CGIVDPQGTLDLDKLAKDIAKDLPKYARPIFIRIMASVDMTGTFKMRKVDLQKEGYNPSL
VKDKLYYADPKTGKYLPLGNEEYEKIVSGQIRL

>ARD71230.1 fatty acid transport protein [*Spodoptera exigua*]

MVLVLAALGAACAAVFFSQGFLCMLVSIIILGVVYLLAFHNRWCYVAIKTTPRDLRALLSY
IKILWITRKFSSKDLTLPDIFHDVVNRHPDKPCFLFQDEVWTFKEVEDYSLRVTAVLKAQGI
KKGSIVGLLVNNCPQQPALWMGIARLGAITPLINTNQRGNALIHSVNVAKCDALIFSDEYQ
SAIQDVAKDLSPSLKLFKFSQRPLKTSNSKFEGSGDGIADFTNLVETTSPAPWTLADAEGFQ
GKLLYIYTSGTTGLPKAAVISNARFVFMATGLHYMGLEGNDVFYCPLPLYHTAGGVISVG
QAVIFGCTVALKTKFSASQYFPDCVKYKATAAHYIGEMCRYVLATPPSPADTQHSVRVIYG
NGLRPQIWKDFVKRFNIQSVTEFYGATEGNANIANVDGTPGAIGFVSRIFPKVYPIAIIKVN
QETGEPIRNSKGLCQLAEPNEPGVFIGKIQASNPARQYLGYVDKAASDKKVVKNVFHFGD
SAFISGDILVADEFGYLFFRDRTGDTFRWRGENVSTTEVEAAISRVADHRDAVVYGVLVPN
TEGRAGMCGIVDVDGSLDLDKLCRDLARDLPVYARPVFIRVMDSLDMTGTFKMKKTDL

QKDGFDPKLAKKDRLYYLDLKQGRYLPLGVEEYDKIISGQIRL
>ARD71231.1 fatty acid transport protein [*Spodoptera exigua*]
MTEKTGVRFEEAEKKKGVILTSIAVSCAALGWLTRGCPTVMGAVALLGTYFLTGDRYQWI
YIWKQTHYRDFLGLRVLLYTIFRIWMWERQGKTVVTRWTEVARMSPNKKAFVMEDRAL
TFREGDEFSNRISWYFKRAGYKPGEVVALLMETQPEYVFLWLGLAKIRVTTALINTNLKGS
QLIHCLRIAGCKAVIFGDEMSESVKEIQTEISDIPLFQYNSPDRETAPFVQGTTPLSVELKEM
STEPVIDSEQAKPRDTLLYIYTSGTTGFPKAAIITNIRYLLIPLGVQSSAQLTSSDVIYDPLPL
HHTAGGVLGAGLAIVSGCTVVLRKKFSASNYWSDAAKYGCTATQYIGEICRYLLAVPPGP
NDRAHKVNVIFGNGLRPQIWEEFVKRFGIKRVMEFYGATEGNSNLVNLDSKVGAIGFLSRI
FSTIYPLTLVKCDEITGEILRDSNGRCITCGPHEPGLLLGKIDPKKAILTFAGYADKTASEKK
MVRNVVRTEGDCYFNTGDVLVMDHYGYFYFKDRTGDTFRWRGENVSTAEVEGVISSLIGL
KDAVVYGVKVPNTEGKAGMAAIADPEKTLDLASLAKGLRSSLPVFARPLFVRILPESPLTA
TFKLKKKELMEQGFDVEIVSDPIYFMDQKTGEYVPLTQKLFDDIMKGLVRL
>ARD71232.1 fatty acid transport protein [*Spodoptera exigua*]
MDAILAALVALMALAAAMAAVLSTLSKAAIFVILAVAPCVYRYRRHIYVFIKTLPRDCKFL
WRYANGMIRSKRWGRQDATVAELFTRRALKNPDAPCFFVVGDRDWTFGQMAANSNKVA
RVMQEHMGLKRGDVVCVFMPNCGEYVWTWLGMAKLGAVSALINSNLRHKPLLHCIQV
AKAKAIVFSDQLADAISEVRDQLPEGLKLFQLYGECAPGVLDLAAEMEKHPPEYPIVTDK
PRYKDTLLYIYTSGTTGMPKSAILPNSKYLLVVVATVHMLGLKKSDRMYNPLPLYHMAGG
LVGTGAALVDGIPSVLRTKFSASNYWTDCIKYDCTVAQYIGEMCRYLLAQPARASDAQHR
VRIMVGNGMRSAIWQQIVDRFKVPQINEIYGATEGNANIINVDNTVGAVGFLPKLVPTSLH
PIALVKADEHGTLLRGDDGYCIRCKPHEPGMFIGLIAQGNASREYYGYVDKDDSNKKLVR
DVFCKGDAAFVSGDILVADELGYLYFRDRTGDTYKWKGENVATAEVENAMSPSLQQKAC
VVYGVSIPQTEGRAGMACIADPARALPLSRLARDLDDSLPSYARPLFLRIINDIEITGTFKLK
KLQYQKEGFDPEVIKDPLYFRLGADFVPITPQLYTDICTGKIKL
>ALJ30274.1 putative fatty acid transport protein FATP1 [*Spodoptera litura*]
MSNMEMNVDSNMNKTDVSQQKFELKNGNADIEKGTVVKAGSKIPWTKAIIAMFALGVL
AGACAVAWVFQDWKASLLVLAILALVYCIAFYWRWIYVAIRTAPRDFSALYCYIKILRLTK
NFTKKNWSMPDIFHQLVVKHPNKACFLFEDETWTFQQVEEYSLRVSAVLKGKGVKRGDT
VAVMIGNCPEMPSIWLGATRLGAVCPLINTNQTGNTLLHSITIAKCDVVIYSDEFQTAFQDI
SKELSPSLKLFKFVRRPLNTAPDAVKVVESDDDFTSMLENTAPFPWTPSDSDGFNGKLLYI
YTSGTTGLPKAAVISSSRMVFMASGVHYLGSLRKSDVIYCPMPLYHSAGGCITMGQAFIFG
CTVALRTKFSASRYFPDCIKYNATAAHYIGEMCRYVLTTPPSPTDTQHKVRTVYGNGMRPA
IWTDFVKRFNIKKVVEFYGATEGNANIVNINNKTGAIGFVSRIIPAVYPIAILKVDPDSGEPI
RDDRGLCQLAKPNEPGVFIGKIKPNNPSRAFLGYVDKAASDKKIVRDVFDYGDSAFISGDI
LTADELGYLYFMDRTGDTFRWRGENVSTTEVESAISRVADQRDAVVYGVEIPNTDGRAGM
CGIVDPQETLDLDKLAKDIAKDLPKYARPIFIRIMASVDMTGTFKMRKVDLQKEGYNPSL
VKDKLYYADPKTGKYVPLGNEEYEKIMSGQIRL
>ALJ30275.1 putative fatty acid transport protein FATP2 [*Spodoptera litura*]
MVLVLAALGAACAAVLFTQGFLCMLVSIIILGIVYLLAFHQRWCYVAIKTTPRDLRALLSYI
KILWITRKFSSKDLTLPDIFHDIVSRHPDKPCFLFQDEVWTFKEVEDYSLRVTAVLKAQGIK
KGSIVGLLVNNCPQQPALWMGIARLGAITPLINTNQRGNALIHSVNVAKCDALIFSDEYQS
AIQDVAKDLSPSLKLFKFSQRPLKTSNSKSEGSGDGIADFTNLVETTSPAPWTLADADGFQ
GKLLYIYTSGTTGLPKAAVISNARFVFMATGLHYMGLEGNDVFYCPLPLYHTAGGVISVG

QAVIFGCTVALKTKFSASQYFPDCVKYKATAAHYIGEMCRYVLATPPSPADTQHSVRVIYG
NGLRPQIWKDFVKRFNIQSVTEFYGATEGNANIANVDGTPGAIGFVSRIFPKVYPIAIIKVN
QETGEPIRNSKGLCQLAEPNEPGVFIGKIQASNPARQYLGYVDKAASDKKVVQNVFHFGD
SAFISGDILVADEFGYLFFRDRTGDTFRWRGENVSTTEVEAAISRVADHRDAVVYGVLVPN
TEGRAGMCGIVDVDGTLDLDKLCRDLARDLPVYARPVFIRVMDSLDMTGTFKMKKTDL
QKDGFDPKLAKKDKLYYLDLKQGRYLPLGVEEYDKIISGQIRL
>ALJ30276.1 putative fatty acid transport protein FATP3 [*Spodoptera litura*]
MTEKTGVRFEEAEKKKGVILTSIAVSCAALGWLTRGCPTVMGAVALLGTYFLTGDRYQWI
YIWKQTHYRDFLGLRVLLYTIFRIWMWERQGKTVVTRWADVARMSPNKKAFVMDNRAL
TFREGDEFSNRISWYFKRAGYKPGEVIALLMETQPEYVFLWLGLAKIRVTTALINTNLKGS
QLIHCLRIAGCKAVIFGDEMSESVKEIQSEIPDIPLFQYNSPDREKAPFVQGTAHLSVELKE
MSTEPVIETEQAKPRDTLLYIYTSGTTGFPKAAIITNIRYLLIPLGVQSSAQLTPSDVIYDPLP
LHHTAGGVLGAGLAIVSGCTVVLRKKFSASNYWSDAAKYGCTATQYIGEICRYLLAVPPG
PNDRAHKVNVIFGNGLRPQIWEEFVKRFGIKRVMEFYGATEGNSNLVNLDSKVGAIGFLS
RIFSTIYPLTLVKCDEITGEILRDSNGRCITCGPHEPGLLLGKIDAKKAILTFAGYADKTASEK
KMVRNVRNEGDCYFNTGDVLVMDHYGYFYFKDRTGDTFRWRGENVSTAEVEGVISSLV
GLKDAVVYGVKVPNTEGKAGMAAIADPERTLDLATLAKGLRSSLPVFARPLFIRILPESPLT
ATFKLKKKELMEQGFDVEIVSDPMYFMDQKTGEYVPLTQKLFDDIMQGLVRL
>ALJ30277.1 putative fatty acid transport protein FATP4 [*Spodoptera litura*]
MDAILAALVALMALAAAMAAVLSTLSKAAIFVILAVAPCVYRYRRHIYVFIKTLPRDCKFL
WRYANGMIRSKRWGRQDATVAELFTRRALKNPDAPCFFVVGDRDWTFGEMAANSNKVA
RVMQEHMGLKRGDVVCVFMPNCGEYVWTWLGMAKLGAVSALINSNLRHKPLLHCIQV
AKAKAIVFSDQLADAISEVRDQLPESLKLFQLYGECSPGVLDLAAEMERHPPDYPIVTDKP
RYKDTLLYIYTSGTTGMPKSAILPNSKYLLVVVATVHMLGLKKSDRMYNPLPLYHMAGGL
VGTGAALVDGIPSVLRTKFSASNYWTDCIKYDCTVAQYIGEMCRYLLAQPARPTDAQHRV
RIMVGNGMRSAIWQQIVDRFKVPQINEIYGATEGNANIINVDNTVGAVGFLPKLVPTSLHPI
ALVKADETGTLLRGDDGYCIRCKPHEPGMFIGLIAQGNASREYYGYVDKDDSNKKLVRN
VFCKGDAAFVSGDILVADELGYLYFRDRTGDTYKWKGENVATAEVENAMSPSLQQKACV
VYGVSIPQTEGRAGMACIADPARALPLSRLARDLDDSLPSYARPLFLRIINDIEITGTFKLKK
LQYQKEGFDPEVIKDPLYFRLGADFVPITPQLYTDICTGKIKL
ACD
>AID66666.1 short-chain specific acyl-CoA dehydrogenase, partial [*Agrotis segetum*]
MASSPLLKSSKLGIYSRKCLKTLSQHRTFTTQLTEQQVCIQEMARNFASEHLKPNAAKHDT
EARFPFEPIKKLASMGLMGACVDPKKGGLGLDYLSLALAVEELSR
>AID66667.1 short-chain specific acyl-CoA dehydrogenase [*Agrotis segetum*]
METEGVFRGKKERIMSVSETLRAATACPVTLEEVRIPRDYIVGEPGDGFRIAMEQLDQARI
GIAAHAVGIAQSALDTAVSYAKKRIAFGKPLSRLASVKDRLTEMVMLVETARLATYRAAV
DVSTKNSAMAKYLAGRNATAVADHCVQILGGRGLSVNYDAERHYRDARGTQIYGGVTDI
QKRLVGHYFLKENNAL
>AID66668.1 short-chain specific acyl-CoA dehydrogenase [*Agrotis segetum*]
MIKNFSKLVQTLAPTSVRQTRCIASLSALSEDYQMLYKTCRDFAEGELKPNAAKFDREHL
YPGDAIKKMGELGLMAIAVPEELGGAGLDYLAYAIALEEISRGCASAGVIMSVNNSLYLGP
VLHWGTDKQKEQFVKPFTSGEIVGCFALSEPGNGSDAGAASTTAKDGGDKWVLNGTKC
WITNGYESKASVVFATTDKSLKHKGISAFIVPKPIKGLELGKKEDKLGIRGSSTCSLMFEDC

SIPKENILGQPGLGFKIAMMTLDAGRIGIASQALGIAQASLDVAAEYASKRTAFGKPIMKLQ
SIQNKLADMALQLESARLLTWRAAWLKDNKKPYTKEAAMAKLAASEAATFLSHQCIQIL
GGMGYVSDMPAERHYRDARITEIYEGTSEIQRLVIAGQLIKEYGLN
>AID66669.1 short/branched chain specific acyl-CoA dehydrogenase [*Agrotis segetum*]
MFPLRRVGSKILEQWRSPVVATGMQRNYSSEVTPPRPLSVLTEDEQTMKETIRKLATEQIA
PLVKKMEEEHRIDDSVRQLLFDNGLMGIETPTDYSGSGCGFLTMMVVVEELSRVDPAVAA
FVDIHNTLVNSLFMKLGTEEQKQKYLTKLCTEYAGSFCLTEPSSGSDAFALKTVAKKDGEH
YVISGSKMWISNSDVAGVFLVMANADPSKGYKGITCFIVERDTPGLSVAKPENKLGIRASG
TCMVHFDNVRVHESAILGEYGKGYKYAAGFLNEGRIGIASQMIGLCQGCMDATIPYTLER
KQFGKSIYSFQGISYQIAHLQTQLEAARLLTYNAARLKENGLEFVKEAAMAKYYASEIAQ
KLTSKCIDFMGGVGFTKDFPQEKFFRDAKIGTIYEGTSNMQLQTIAKLIERQYTQ
>AID66670.1 putative medium-chain specific acyl-CoA dehydrogenase [*Agrotis segetum*]
MNPITQVIRATRPIYRKLSTTAPVAAAKPLPTTGMCFELSEEQKALQDLARKFTREEIVPVA
AQYDKTGEYPWPIVKKAWEIGLMNGHIPEHCGGVGGNMGVLEECIAAEEMAFGCTGITT
AVGGTTLGQMPVIIAGNKEQQKKYLGRLVEEPIVAAYCVTEPGAGSDVAGVKTRAEKKG
DEWIINGQKMWITNGGVANWYFVLARTNPDPKCPASKAFTGFIVERDWPGVTPGRKEQN
MGQRASDTRGITFEDVRVPKENVLIEEGAGFKIAMGAFDKTRPPVAAGATGLAQRALTEA
TKYALERKTFGVPIARHQAVAFMLADMAIGVETARLAWMKAAWMADHGIRNTVLASVA
KCHASEIANKAAADAVQIFGGNGFNTDYPVEKLMRDAKIYQIYEGTSQIQRLIISREIITQA
MQSN
>AID66671.1 acyl-CoA dehydrogenase family member 9 [*Agrotis segetum*]
MNIARKLCTIHHSYVSRNLYRKFRFSAITYDNATATQPQVKEEKFDFEDLNVLERTERRKA
KIEPFMKDIFTSIFNKDLLAYPEILNKEETESLERRINAITNVFIDPKKTTEDRKNILKSTRMY
AAPVSLTRNGLASNITENLRYLEAIAGDFQLGQEMSEHWVALQALAQGLTQEQYSMIIDD
LTVGDKPISLAIKERIAERISQADFRTSADIDGQGIWHLNGEKVCHYTNGYVLVLAIVEATR
LKAFLVHPDASGVSSDGNFVTFMKTPATPLEMITEQKLAQILGLSRLYAAVLSRCQLTAAV
RSVVEYTRPRAFSGKPLAELSTIQSTVGNAILDIYASESAEYFTAGLLDGYVEPDAELEVA
MCRNFISNHGLHTMLNLLSIPALDKEEECKQLLDDMRHLATRGESLDSVNMFIALNGIHH
AGKVMADEVKQIRNPLMHPAFIFKKVLANRHQERDDPKLTLHLSEHLHPSLKQPSEQLEY
CVLRMRFACETLMARHGVKVSTAYTELNPLAEAATEILMMTAVLARASRSYCIGLRNAET
EMKLAACFVERTRDKVKRLIKEIDDGEYLNLDHFTVQFGRKMLDSNSSLVEKPTARVFW
>AID66672.1 very long-chain specific acyl-CoA dehydrogenase, partial [*Agrotis segetum*]
MKGAKLLTCANRCIAGKSTQVQLPLHSCRRLATEAAEKRGAARESGSFTLNLFRGRLETA
QVFPFPEPLSDDQRQTLQELVPPVEKFFQEVNDPAKNDADSKIEPNTVSGLWELGAFGLQV
PTDMGGLGLCNTQYARLVEVVGAHDLGVGITLGAHQSIGFKGVLLFGTPEQKAKYLPRV
TGGEYAAFCLTEPSSGSDAGSIKSRAVLSPDGKHFILNGSKIWISNGGIAEIMTVFAQTPIEK
DGKTIDKVTAFIVERSFGGVSSGPPENKMGIKCSNTTEVYYEDVKIPVENVLGGVGNGFK
VAMNILNNGRFGMAAALAGTQRAALRQAAEHAATRVQFGKRIADYGTIQEKLARMALL
QYTTESLAYMVSGNMDSGAQDYHLEAAISKVFASDSAWTVVDEAIQILGGMGFMKATGL
ERVLRDLRIFRIFEGTNDILRLFVALTGIQFAGSHLQELQRAFKNPTAHLGLIFSEAGRRAAG
>AID66673.1 isovaleryl-CoA dehydrogenase, partial [*Agrotis segetum*]
DRKVVIMAVRLGRVTSILRNCTSKTGTRCMSHYPIDEHVFGLSSEQQQLRQSVFDFAQKEL
APKAQQIDKDNNFAELRQFWKKCGEMGLLGITANPEYGGTGGKYSDHCVIMEELSRASG
GVALSYGAHSNLCVNQIN

>AID66674.1 isovaleryl-CoA dehydrogenase, partial [*Agrotis segetum*]
AHSNLCVNQINRNGTDEQKRKYLPKLCSGEHMGALAMSEPGSGSDVVSMKTRAEKKGD
YYVLNGNKFWITNGPDADVLVVYAKTDTTSKPQHGISAFLIEKGFPGFSTAQKLDKLGMR
GSNTCELVFEDCKVPAANLLGEENKGVYVLMSGLDLERLVLAAGPIGIMQASVDTAFDYA
HTRKQFGKSIGEFQLLQGKMADMYTTLSACRSYLYSVARACDEGHINSKDCAGVILYCAE
KATQVALDAIQILGGNGYINDYPTGRLLRDAKLYEIGAGTSEVRRMLIGRALNNEYK
>ADB57042.1 acyl-CoA dehydrogenase [*Heliothis virescens*]
MNPITQVIRATRPIYRKLSTTAPVAAAKPLPTTGMSFELSEEQKALQDLARKFTRGEIVPVA
AQYDKTGEYPWPIVKKAWEVGLMNGHIPEHCGGMNMDVFDGCMVAEELAYGCTGIMT
AMEASGLGQMPVIIAGNKEQQKKYLGRLVEEPIVAAYCVTEPGAGSDVAGVKTRAEKKG
DEWIINGQKMWITNGGVANWYFVLARTNPDPKCPASKAFTGFIVERDWPGVSPGRKEQN
MGQRASDTRGITFEDVRVPKENVLIEEGAGFKIAMGAFDKTRPPVAAGATGLAQRALTEA
TKYALERKTFGVPIARHQAVAFMLADMAIGVETARLAWMRAAWMADHGIRNTVLASVA
KCHASEIANKAAADAVQIFGGNGFNTEYPVEKLMRDAKIYQIYEGTSQIQRLIISREIITNA
MQSN
ECH
>AID66689.1 enoyl-CoA hydratase [*Agrotis segetum*]
MASVGVVTRVLLGKNVVNRVAAVNTGFVKFYSTGPSYENIKIDVVGAKKNVGLIQLNRP
KALNALCGPLFVELGQAVRDFDANEKIAAIIITGNEKAFAAGADIKEMQNNTFSENTKKGF
LKDWEDVSNCGKPLIAAVNGFALGGGCELAMLCDIIYAGEKAKFGQPEINIGTIPGAGGTQ
RLPRYVGKSKAMEIVLTGNFIDATEAERMGLVSRVFPVEKLLEETIKLAERIGTHSPLIVKM
AKAAVNQAYETTLKSGLLFEKAYFYGTFATEDRKEGMTAFVEKRPPNFKNE
>ADB57043.1 fatty acid beta-oxidation complex subunit alpha, partial [*Heliothis virescens*]
MSNSKIFNALKILRTRKDLKYLAGSHIRTYAAAGSQVHTKCKNVNGIYVVTLDSPNTKVN
SLNTAVMEEVNGVLNEIESNPSIQAAVLISGKPGCFIAGADISMLEACKTKDEFVTLSKRGH
EIFHRIERSRKPIIAAIQGSCLGGGLETALACHYRIAVKDPKTGFGLPEVMLGLLPGGGGTQ
RMPVPTSVPTTLDLALTGKTVKADKAKKLGIVDLLVSPLGPGLSKPEESTMRYLEEVAIQI
ARDIANGKIKVDRSKKGLVEKITASVMQWDMVKNMIFNKAKEQVMK
>ADB57046.1 enoyl-CoA hydratase, partial [*Heliothis virescens*]
MRVLLKRLISVTNIPIQRYAVRLCSSDSQAKAAVKEKPTEQDQKEAENIVVEKKNIVLEKF
GAVMTLNIDRQTTRNSLDIATLKEMTEAINAFDNDPEAKVLVFNGEGGSFCSGFNMNDIG
TVGYQNLKDAAMRLERRPLCDKITIAAVSGYAVVEGFELALSCDLRIIEDTAILGCLGRRFG
VPQSLYGARKLTSLIGLSPALDLLITGRLITGVDANRLGLACKLTSTGTALGESIKLAKSLVK
FLWNAMVMDKMAAINSQLHLNSEESMRDEVIMNSLLGMLLKNMKEGVRSFQQGIG
>ADB57047.1 enoyl-CoA hydratase, partial [*Heliothis virescens*]
MLIPRLFKFNSITRNATARFLATQAQQSNENVSPVVYEKLLGTDRGIALYGLNSPKDRNAL
GFDMIEAMREVNQLIREDTKVSVVILHSMVPGIFCAGANLKERFKMADDEVANFVKGLR
GTFIEIEDLPMPTIAAIEGVAVGGGLELALACDVRVVSETAKLRLVKTCRGLIP
>AID66685.1 methylglutaconyl-CoA hydratase [*Agrotis segetum*]
MLIPFPRLSRCSSLIKHGTVRLLATQTQQYNENVSPVVYEKLLGTDRGIALYGLNSPKDRN
ALGFDMIEAMREVNQLIREDTKVSVVILHSMVPGIFCAGANLKERFKMADAEVARFVKG
LRATFIEIEDLPMPTIAAIEGVAVGGGLELALACDIRVVAETAKLGLVETGRGLIPGAGGTQR
LPRAVNINIAKELIYTSRIVSGTEAKDLGIVNHVVPQSNSNNAALEKSLSIAREIILNAPIALR
CAKQAINEGIQLSIKDGYEVEQKFYEMNIPTKDRQEGMISFMEKRKPIYEGH

>AID66686.1 putative enoyl-CoA hydratase [*Agrotis segetum*]
MRVILNRLFAATNLPIRRYAVRLRSNDAKPAENEKKPEEAQKESENIEVNKKNIVVEKYGP
VTTLNIDRQTTRNSLDIPTLREMAAAIDAFDNDQEAKILVINGEGGTFCSGFNMYEIAKEG
YQNMKDAARRLERRPLCDKITIAAVSGYAVAEGFEIALSCDLRVIEETAVLGCLGRRFGVP
QSLFGARKMTGLIGLSAALDLLITGRLITGVDANRLGLACKLTATGTALGESVKLAKSLAK
FPENAMIMDKMAAINSQLNPNSEDSMRDEAIMSSLLGNAIEDMKEGVKKFQAGIGKHGK
FYKLTEVPLKEWELEETVDEVTVQMKDKPETEKKLT
>AID66687.1 trifunctional enzyme subunit alpha [*Agrotis segetum*]
MSNSKIFNALKILRTRKDLKYLTGSHSRTYAAAGSQVHTKCKNVNGVYVVTLDSPNTKV
NSLNTAVMEEVSQVLNEIESNPSIQAAVIISGKPGCFIAGADITMLEACNTKEEFVDLSKRG
HGIFHRIERSRKPIIAAIQGSCLGGGLETALACHYRIAVKDTKTGFGLPEVMLGLLPGGGGT
QRMPVLTSIPTTLDLALTGKTVKADKAKKLGIVDLLVSPLGPGLSLPEESTMKYLENVAIQI
ARDIANGKIKVDRAKKGLVQKITASIMQMDAVKNMIFNKAKEQVMKASRGLYPAPLKILE
VVRTGVDKGPTAGYEAEAQGFGELAVTPQSRGLIGLFRGQTECKKNRFGKSKVDVKTIGV
LGAGLMGAGIVQVSINKGYHVVMKDATNPGLFRGVGQIQNGLATAVKRKRMSGLQRDQ
FLSNLLPTLDYEKMRNCDCVIEAVFEDLNVKHKVIKELEAVIPKHAILATNTSAIPITKIAAG
SSRPDKVIGMHYFSPVDKMQLLEIIRHPGTSDDTAAAAVGVGLRQGKVVITVGDGPGFYT
TRILSTMLSEAVRLLQEGVDPKTLDSLTKNFGFPVGAVTLADEVGIDVGSHIAVDLAKAFG
DRFSGGNLEVMPDFVKAGFLGRKSGKGFYVYEKGSKSKEVNQEAVNILKERYPLEPRGA
NTAEDQQLRMVSRFVNEAVLSLEEKILHSPLEGDVGAVFGLGFPPFTGGPFRWVDQFGAD
KLVKKMEEFHGLYGAPFKPAQTLVDMARDGKKFYKN
>AID66688.1 3-hydroxyisobutyryl-CoA hydrolase, partial [*Agrotis segetum*]
TSMVSKLLPQLQEWESKKTLVIVKGAGDKAFCAGGDVKAAIDKVEGPRFFHTEYNVNYL
IGKYKIPYIAFMNGITMGGGLGLSVHGRYRIATEKTVIAMPETKIGLFPDVGGSYFLPRLQV
NLGLYLGLTGDRLKGWDVVKSGIATHFVPSKRLYELEVLLSRCADEGEISNLLSKFNEPSD
KFSLSDNIKHINYCFAASTIEEIIERLEKVQNEWSVKTLKTLSQMCPGSLKITLRALQRGSQ
LELNQCLKMEYRVACRATENHDFPEGVRALLIDKDNNPQWKPRTLAEVDDDYVESYFKK
LPQERELQYFDSKL
>AID66690.1 enoyl-CoA hydratase domain-containing protein 3 [*Agrotis segetum*]
MLVSLQKKCFRPLYVHCRALHSQYVKINENNGAREITLNHEKTKNSLSLDMMKHLIEAIN
KNKDDTSLRAIVLSAKGNVFSAGHNLKELQSNTGVDQHKLIFSKATELMKAIIQSPVPVIA
KVNSFAAAAGCQLVATCDMIVCSDTSKFSTPGANFGIFCSTPGIAVGRCVPKSRATYMLFT
GEPLTAQEAYESGLVTKVVPASELDNEVNKIIEKIKHKSRSVIALGKEFYYKQIDLSLMDAY
KLGEDIMVKNINTNDGQEGIKSFVEKRKAVWSHE

HCD

>XP_022823313.1 hydroxyacyl-coenzyme A dehydrogenase, mitochondrial-like [*Spodoptera litura*]
MAGDASAEDIDIAMKLGAGYPMGPLELADFTGLDTKKFVLGVMHEKTGLPAFEPIPLLN
KLVSEGKFGRKTGEGFYKYDK
>XP_022822785.1 hydroxyacyl-coenzyme A dehydrogenase, mitochondrial-like [*Spodoptera litura*]
MTKLVQFGVIARNFSSSSAMQSAIKNVTVIGGGLMGSGIAQVSAQAGQNVILVDVSSDVL
AKSQKSIGANLGRVAKKMYKDKPQEGEKFVTDAMARIKTSTDPSEASKSADLVVEAIVEN
MSVKHKLFSQLDGVAPNHTIFASNTSSLSINEICSVVKRKDRFGGLHFFNPVPVMRLLEVV

RGAETSDATYKTMMEWGKAVGKTCITCKDTPGFVVNRLLVPYICEAIRLYERGDASARDI
DTAMKLGAGYPMGPLELADYVGLDTNKFILDGWHKKYPDQPLFNPIPLLDKLVAEGKLG
VKAGEGFYKYDKK
>XP_021195856.1 hydroxyacyl-coenzyme A dehydrogenase, mitochondrial-like [*Helicoverpa armigera*]
MTKLVQFGVIARNFSSSSAMQSAIKNVTVIGGGLMGSGIAQVSAQAGQNVILVDLSSDVL
AKSQKSIGANLGRVAKKVYKDKPQEGEKFVAESMARIKTSTDPAEAAKSADLVVEAIVEN
MNVKHKLFSQLDAVAPNHTIFASNTSSLSINEICSVVKRKDRFGGLHFFNPVPVMRLLEVV
RGAETSDATYKTMMEWGKAVGKTCITCKDTPGFVVNRLLVPYICEAIRLYERGDASARDI
DTAMKLGAGYPMGPLELADYVGLDTNKFILDGWHKKYPDQPLFNPIPLLDKLVAEGKLG
VKAGEGFYKYDKK
>AID66691.1 3-hydroxyacyl-CoA dehydrogenase, partial [*Agrotis segetum*]
MLKGMVSLVTGGASGLGKATVERFVKNGGKVVILDIQGTRAKKVAQELGENVAVATGCV
TSEEDVKKALEIVRDKFGRLDTLVNCAGQSETHQIYNFLKDKSCELDGFMRCINVNTIGTF
NTIRLSAGLIGKNKPDDNGQRGVIVNTASTIAYEGDIGQAAYAASSAAIIGMTLPIARDLAS
QGIRVVTVAPGLFETPLITYLPDKMLDFIKRMTPFPSRLGKPEEFAHLVTSIVENPMLNGEVI
RLDGAQRWFP
>AID66692.1 3-hydroxyacyl-CoA dehydrogenase [*Agrotis segetum*]
MFKGLVGLVTGGASGLGRATVEQLLKQGGRVVICDLPTSTGQETAKQLKENVAFVPIDVT
SEKDVKNALQTTIDKFGRLDVVVNCAGVATASRVYNFKKDQPFDLKSFQRTIEVNLIGTFN
VIRLAAGLIGKNAPDADGQRGVIVNTASVAAFDGQIGQAAYSASKAGVVGMTLPIARDLA
KQGIRVVTIAPGLFRTPMMEQLPEPAIKSLEATVPFPPRLGHPQEFALLVQSIIQNPMLNGET
IRLDGSLRMQP
>AID66693.1 peroxisomal multifunctional enzyme [*Agrotis segetum*]
MDQLRFDGRVAVVTGAGGGLGKAYALLLGSRGAKVVVNDLGGARDGVGKSNFADAVV
KEIKDKGGIAVADYNNVVEGEKIIKTALDNFGRIDILINNAGILRDKSFTKMSDQDWDLIHL
VHLKGAFKTTHAAWETFRKQKYGRVIMTSSNAGIFGNFGQANYSAAKMGLVGLTNTLAI
EGSKYNIKVNTIVPTAASRLTEDILPPEMFEAMKPELIAPVVAYMVHESFPDTGAVIDSTLG
YATKMHYVRAPGAILKKKPSDPVTIESVREFWPQVTNMNGAIHLDKMAEVTVDLVEKIQ
DFEERSKLDDGRESYWSSYKYDSKDLMLYALGIGASVQNESDLKFLYESHEGFAALPTYFI
LPGMALESPLVANSMPPGKHADFTNILHGEQFIEFVGDFPGTEGDFKIRSYVVDLLDKGSS
AVSIVNSEIYQNKQLIARTQQHIFVLGQGGFNGPRNSKLAVDVQPAPKRAPDAVIEQRTAED
QAALYRLSGDMNPLHIDPNVATASGHQKPILHGMATLGFSARHVLAKYGGNEPNNFKAL
KARFVKPVQPGNTLVTEMWLEGKRVHFQTKVKESGNIVIAGAYVDLKNVVAGQVGSSSA
APAAAPSGGSFKSDALFAKIKEEVGKNKDLAKSIGGVFQYNISENGKTAKSWTLDLKTPE
VYEGTPKSGKADTTLTVSDDDMVAIAAGSLSPQVAYMKGKLKIAGNIMLAQKLGPLLKSP
AKI
>AID66694.1 hydroxyacyl-CoA dehydrogenase [*Agrotis segetum*]
MTKLVQFGVIARNFSSSSAMQAAIKNVTVIGGGLMGSGIAQVSAQAGQNVILVDLSPEVL
AKSQKSIGANLGRVAKKMYKDKPQDGEKFVSESMARIKTSTDPAEAAKSADLVVEAIVEN
MSVKHKLFSQLDGVAPNHTIFASNTSSLSINEICSVVKRKDRFGGLHFFNPVPVMRLLEVV
RGAETSDATYKSMMEWGKAVGKTCITCKDTPGFVVNRLLVPYICEAIRLYERGDASARDI
DTAMKLGAGYPMGPLELADYVGLDTNKFILDGWHKKYPNQPLFNPIPLLDKLVAEGKLG
VKAGEGFYKYDKK

>AID66695.1 putative 3-hydroxyacyl-CoA dehydrogenase [*Agrotis segetum*]
MNKLIGITRNFSSSTSLNAIKTVTVVGGGLMGSGIAQVAAQAGQNVTIIDINAELLDKAQK
SIQTNLTRVGKKLYKGDAAKIDSFVKESAERIRVSTKLEDGADVDLIVEAIVEKLDAKQEL
FNKLDVLSPGRTIFATNTSCISVNAIGSGIKRKDRYGGLHFFNPVPVMRLLEVIKCDDTSEE
TYQAMMEWGKAVGKTCITCKDTPGFVVNRLLGPYSAEAIRMLERGDASKEDIDIGMKLG
AGLPMGPFELADYTGLDTNRLAQQALYSMTKNEVFAPIELLEKMVQEGKYGIKSGEGFY
KYNKK
>ADB57043.1 fatty acid beta-oxidation complex subunit alpha, partial [*Heliothis virescens*]
MSNSKIFNALKILRTRKDLKYLAGSHIRTYAAAGSQVHTKCKNVNGIYVVTLDSPNTKVN
SLNTAVMEEVNGVLNEIESNPSIQAAVLISGKPGCFIAGADISMLEACKTKDEFVTLSKRGH
EIFHRIERSRKPIIAAIQGSCLGGGLETALACHYRIAVKDPKTGFGLPEVMLGLLPGGGGTQ
RMPVPTSVPTTLDLALTGKTVKADKAKKLGIVDLLVSPLGPGLSKPEESTMRYLEEVAIQI
ARDIANGKIKVDRSKKGLVEKITASVMQWDMVKNMIFNKAKEQVMK
>ADB57049.1 3-hydroxyacyl-CoA dehydrogenase, partial [*Heliothis virescens*]
VAAQAGQNVTIIDINSDLLGKAQTSIQANLTRVGRKLYKGDEAKINSFVKESFERIRVSTKL
EDGADVDLIVEAIVEKLDAKQELFNKLDQLSPARTIFATNTSCISVNAIGSGIQRKDRYGGL
HFFNPVPVMRLLEVIKCDSTSEQTYQAMMEWGKSVGKTCITCKDTPGFVVNRLLGPYFA
EAIKM

KCT

>AID66700.1 3-ketoacyl-CoA thiolase, partial [*Agrotis segetum*]
ILFLLERRCFNGGNLICGNQGLMVEDQRNRVKLSKIVGATSMFVSGSSDGILTPRHSALKA
GVPYDKPALGVNKLCGSGIQAMVNSAQDILLGSAQISLAGGTENMSAIPFLVRNLRFGTQ
LGQVRPFEDFLKAGALDSYCNYTMAQTAENLAKMYDLKREQLDEFALKSQMKWKAGF
KNGAFEAEMAHVTVTVGGKPVVVNKDEHPRTNTTLESLSKLPALFREGGVGTVGNSTGV
NDGAGALILASEEAIKQHNLTPLARLSCWSHAGVEPRVMGLGPVPAVRQLLAATGYTLDD
MDMFEINEQFAAQALASVLEIGLDQDKLNMNGGALAMGHPAAASGARIAAHLTHELRRR
GLKRGIGATCIGGGQG
>AID66699.1 3-ketoacyl-CoA thiolase, partial [*Agrotis segetum*]
ATIKKKGVVVDKDQPAQLSIKTEELKQFPTLIENGEILTAGNISAPADGAAALLIADEEAVK
SHNLRP
>AID66698.1 trifunctional enzyme subunit beta [*Agrotis segetum*]
MASQISKSLIKVSHVGSTAKFDTARRALSVGAALHAKRNSLPDRTGKNVVLVDGVRTPFL
VSFTDYAKMMPHELARHSLLGLLQKTGISKDVIDYIVYGTVIQEVKTSNIGREAALAAGFS
DKTPAHTVTMACISSNQAITTGVGMIAAGAYDVIVAGGVEFMSDVPIRHSRKMRSLLLRL
NRAKTPAQRLSLIATIRPDFFAPELPAVAEFSSGETMGHSADRLAAAFGASRQEQDEYSLRS
HKLAAEAQQKGYFTDLIPVKVDGKDGVVDKDNGIRVSTPEQLAKLKPAFVKPHGTVTAA
NASFLTDGASACLVMSEAKAKELGLKPKAYLRDFTYVAQDPVDQLLLGPTYGIPKILDKA
GLKISDIDTWEIHEAFAGQILANLKAMDSDWFAQTYLGRQSKVGTPDLEKWNKWGGSLS
IGHPFAATGVRLAMHTAHRLVREDGQFGVISACAAGGQGVAMILERHPDATCN
>AID66697.1 3-ketoacyl-CoA thiolase, partial [*Agrotis segetum*]
MAVAVKKGVYIVAGKRTPFGKYGGLLRDVLAEDLFATAAKAALKAGDVPGDLVDTVNIG
QISPISQSGLSPRHAALKTGIPADRPVLSMNRLSGSSFNAMLCSAQEILLGAAKISLAGGME
TLS
>AID66696.1 3-ketoacyl-CoA thiolase, partial [*Agrotis segetum*]

ETLSSIPFLIYGVRFGTQFGKPIELEDFLRHGNIDTYCNKFLPQTADVVAAKYGITRREADE
YALRSQQRWKHADASGLFSEELVSVPVKIKSREVLMTRDEHPQPDVTLEKLSRLQPVSTG
GITTAGNITGLNDGAAAMILANGQALRDHNLKPLARIVGWSVVGVDPVMMGYAAVPAVE
TLLKTTGLTIDDMDLVEIHETFAATTVVCARHLGVDEDKMNVNGGAIAMGHPSGASGARI
VSHLTHELRRRGLKRGIASAGIAGGQGIAIIIETV

DES

>ALJ30226.1 putative desaturase des2 [*Spodoptera litura*]
MAPAQKHVQMCGDEIQSGLKISPLTYDADKLNEAPQYENNNTVLRNSANDKVNSEADFD
INKYEAIDFKAKIRWPDLTVQILLHLVSIYGLYLMISNQVKLLTLLFALGTIYTSGFGITAGV
HRLWSHRAYRARLPLRIILALLFTVTGQRDIYTWALDHRVHHKYAETVADPHDIRRGFWF
AHVGWLVLTPHPAVEDRRIALKPTCADLLADPVVRLQKQFFIPLFALLNIAIPIWVPWYCW
NETLINSFVISFVMRFTITLNIAFCVNSFAHLWGNKPYDRFVKSVENSLVSLAALGEGWHN
YHHVFPWDYRTSELGKLNISTGFIDFFAKIGWAYDLKAATTDMISNRAKRCGDGTFGESEE
PYPTSEKCHAE

>ALJ30227.1 putative desaturase des3 [*Spodoptera litura*]
MAPNTEKRQVSFPQLEYPIYREAQPKSAQHWLKGKRMQDGAEDLWRIHDSLYDLTDFISS
HPGGSQWIAVNKGTDITEAFETHHLKGIAESLLPNYYVRKATKPRNQPFTFKEDGFYKTLK
LKVMDQIQSIPKDVRKKSDFITDGLLLALIVLAPLSCWGWTQSFIIGASLTLMTSYVLSSVV
TCAHNYFHRGDNWRMYIFNLAGMSFSDWRVSHSMSHHLHTNTAQDIELSMIEPFLQFIPY
KDKPIWAQMGAFYYPLVYATSLLSIMGHELILSATNHEGKTLTWKNLIPFLIPTWMYIMGG
LPLHWTILLWLATMVPASFFFVFYGLTAGHHNHRNFFEGDVPRDENIDWGIHQLDAICERI
DYAGNHFKSITRFGDHALHHLFPTLDHAELKYLYPVLLEHCEKFEFQFKTNTFYETIINAS
KQLIRKRPNNFRDVKATK

>ALJ30228.1 putative desaturase des4 [*Spodoptera litura*]
MAHITKTISSKILNKSTHRCLSTAVSQIRIYEVGPRDGLQNEAKFVPTDIKIELINKLAAAGI
KDIESASFVSPKWVKQMSDGVDVMKNIPRVPGVNYPVLVPNLKGYDIAKQCNIEEVAIFP
AGSEAFSQKNLNCSVEEGLKRFKLVADQAVKDGIRVRGYVSCVVGCPYEGPIHPKGIAKIT
EQLFEMGCYEVSLGDTIGVGTAGSVKRLMREVLTVAKPEQLALHFHDTYGQALSNLVAG
LEFGIKTVDSSISGLGGCPYARGASGNLATEDLVYLLYGLGVNTNVDLVKLIEAGRYISNFL
GKPTESKVNRAISDRFKNHSDIVKIASCDI

>AGH12217.1 delta 11 desaturase [*Spodoptera litura*] des5
MAQTIQTTTILEQKEEKTVTLLVPQAGKRKFEIVYFNLVSFAYWHIAGLYGLYLCFTSAKW
ATILFSFFLFVVAEVGVTAGAHRLWSHKTYKAKLPLQILLMVMNSLAFQNTAIDWVRDHR
LHHKYSDTDADPHNASRGFFYSHIGWLFVRKHPDVKKRGKEIDISDIYNNPVLRFQKKYA
IPFIGAVCFALPTLIPVYGWGETWTNAWHVAMLRYIMNLNVTFLVNSAAHIYGKRPYDKKI
LPSQNIAVSIATFGEGFHNYHHVFPWDYRAAELGNNCLNFTTKFIDFFAWIGWAYDLKTVS
KEMIKQRSKRTGDGTNLWGLEDVDTPEDLKNTKGE

>ALJ30229.1 putative desaturase des6, partial [*Spodoptera litura*]
ATFGEGFNNYNNVFKWDYREDEIGNKCLNLNKKLIDLFEWIGWEYDIKNV

>ALJ30230.1 putative desaturase des7, partial [*Spodoptera litura*]
LLCFVIPAWIPCYFWGENPWYSWYVASITRYTVALHFTWLVNSAAHIWGNRPYDKNIGAT
DNKAVAICAFGEGWHNYHHVFPWDYKAAELGNYSTNLSTALIDFAAKHGLAYDLKTVS

>ALJ30231.1 putative desaturase des8 [*Spodoptera litura*]
MGARVSRTDFEWVYTEEPHASRRKIILEKYPQIKKLFGYDPNFKWVVTAMVLVQIISLPFV

TQLSWPLMLVVAYCFGGVINHSLMLAIHEIAHNLAFGHNRPLANKLFGFFANLPIGLPVSIS
FKKYHLEHHRYQGDEVIDTDLPTLIEAKLFCTTGGKLLWLFLQPFFYSFRPLVVRPKAPTP
MELINLVIQLFFDAVIIKLWGWKALGYLLLGAVMAMGVHPVAGHFVAEHYMFKKGYETY
SYYGPLNWITFNVGYHNEHHDFPAVPGSKLPEVRRIAPEFYDTLPHHDSWSKVLYDFVMD
PDIGPYARIKRKHHGLDS
>AGH12218.1 delta 9 desaturase [*Spodoptera litura*]
MPPQGQTGGSWVLYETDAVNVDSEAPVIVPPSAEKREWKIVWRNVILMGLLHIGGVVYGA
YLFLTTAMWRTSLFAVFLYICSGLGITAGAHRLWAHKSYKARLPLRLLLTLFNTLAFQDAVI
DWARDHRMHHKYSETDADPHNATRGFFFSHVVWLLVRKHPQIKAKGHTIDLSDLKNDPI
LRFQKKHYLILMPLVCFILPCYIPTLWGESLWNAYFVCSIFRYVYVLNVTWLVNSAAHLW
GAKPYDKNINPVETKPVSLVVLGEGFHNYHHTFPWDYKTAELGDYSLNLTKLFIDFMAAI
GWAYDLKTVSSDVIQKRVKRTGDGSHAVWGWDDHEVHQEDKELAAIINPDKTE
>ALJ30232.1 putative desaturase des10 [*Spodoptera litura*]
MPHNTNWEEAAQQRAVDKNTHVSFPQLKYPSLRDEGLRDPVQWLAGKAMDDGAEGLW
RIHDKLYDLTRFIKRHPGGEEWLELTQGTDITEAFESHHLNPSTEKILTQYYIRDAKTPRNSP
FTFKEDGFYKTLKRAAFEELKKIPKDASRTANNITDFLFVSLLISSSLTCWVTNNIAVKFWY
TYASFNLAVLTVACHNYIHRKTNWRMYLFNMSMWSYRDFRVSHVLSHHLYTNTLMDLEL
SSLEPILFYTPRKDKPLHAKLGCITEIFFFPFVFFLSFTKRFLSIFLQQGFFKSHYRWHDAIGL
LLPLWMAITSGAPFLDVISMWLWINCSGSLIFFSIAVNAAHHHPDAIKDGDQPASETPDWG
MHQVEALLDRKDVNGNVFAVMTLFGDHCLHHMFPTLDHSVLKYMHPLFIDLCEKYQAN
YRVSTQFKLVLGQIKETMRTEFKMKND
>ALJ30233.1 putative desaturase des11 [*Spodoptera litura*]
MAPNISEDVNGVLFESDAATPDLALARPPVQKADNKPKQLVWRNIILFAYLHLAALYGGY
LFLFSAKWQTDIFAYILYVISGLGITAGAHRLWAHKSYKAKWPLKVILIIFNTVAFQDAAM
DWARDHRMHHKYSETDADPHNATRGFFFSHIGWLLVRKHPDLKEKGKGLDMSDLLADP
VLRFQKKYYLLLMPLACFVMPTVIPVYLWGETWTNAFFVAAMFRYAFILNVTWLVNSAA
HKWGDKPYDKSIKPSENMSVAMFALGEGFHNYHHTFPWDYKTAEFGNNKLNFTTAFINF
FAKIGWAYDMKTVSEDIVKNRVKRTGDGSHHLWGWGDENQPKEEIEAAIRINPKDD
>ALJ30234.1 putative desaturase des12, partial [*Spodoptera litura*]
MLSLYGTYLLLFEVKMMTLLFFMLLTSVALLGMTTGAHRLWAHQTYQASTGLKIMLMLF
QTLAGVGSIYDWVKYHRFHHAHFATDVDPYDYNQGFIHSHLITRLRKLSPHQEKLMQSID
MSDLEKDTVVMFQKKLYWLLYAIIFVLLPLNAPLEYWDDTILCSAFVIGFLRYLVVLHGS
WLIESAISVWGLKPGEKSPPDTNAVFILTKTFWPHYHYLVPYDYKSGEYGTYDGGCSTAFI
RVWAALGLATKLRTVETASIQKALADAARTKKDLKTCIDAAVNNQQLPEEHYLKRA
>ARD71178.1 desaturase [*Spodoptera exigua*] des2
MAPAQKHVQMCGDEIQSGLKISPVTYDADKLNEGPQYENNNTVLRNSANDKVNSDADF
DISKYEAIDFKAKFRWPDLTVQILLHLVSIYGLYLTFSNQVKILTLLFALGTIYTSGFGITAGV
HRLWSHRAYRARLPLRIILALLFTVTGQRDIYTWALDHRVHHKYAETVADPHDIRRGFWF
AHVGWLVLTPHPAVEDRRIALRPTCADLIADPVVRLQKQFFIPMFALLNIAIPIWVPWYCW
NETLVNSFVISFVMRFTITLNIAFCVNSFAHLWGNKPYDRFVKSVENSLVSLAALGEGWHN
YHHVFPWDYRTSELGKLNISTGFIDFFAKIGWAYDLKAATTDMISKRAKRCGDGTFGESEE
PYPSSEKCHAE
>ARD71179.1 desaturase [*Spodoptera exigua*] des3
MAPNTEKRQVSFPKLEYPIFREAQPKSAQHWLKGKRLQDGAEDLWRIHDSLYDLTDFISS

HPGGTHWISVTKGTDITEAFETHHLKGIAESLLPNYYVRKAIKPRNQPFTFKEDGFYKTLK
LKVMDQMALIPKDVRKKSDFITDSLLLALIILAPLSCWGWTQSFVIGASLTFSTGFVLSSLV
TCAHNYFHRGDNWRMYIFNLAGMSFNDWRVSHSMSHHLHTNTAQDIELSMIEPFLQFIPY
KDKPIWAQMGAFYYPLVYATSLLSIMGHELILSATNHEGKTLSWRNFIPFTIPAWMYLMGG
LPLHSTILLWLVTLVPASFFFVFYGLTAGHHNHRNFFEGDVPRDENIDWGIHQLDAICERID
YAGNHFKSITRFGDHALHHLFPTLDHAELKYLYPVLLEHCEKFDFQFKTNTFYETIINASK
QLIRKRPNNFRDVKATK
>ARD71180.1 desaturase [*Spodoptera exigua*] des4
MAHITKTISSKILNKSTHRSLSTAVSQIRIYEVGPRDGLQNEAKFVPTDIKIELINKLAAAGI
KDIESASFVSPKWVKQMSDGVDVMKNIPRVPGVNYPVLVPNLKGYDIAKQCNIEEVAIFP
AGSEAFSQKNLNCSVEEGLKRFKLVADQAVKDGIRVRGYVSCVVGCPYEGPIHPKGIAKIT
EQLFEMGCYEVSLGDTIGVGTAGSVKRLMREVLTVAKPEQLALHFHDTYGQALSNLLAG
LEFGIKTVDSSISGLGGCPYARGASGNLATEDLVYLLYGLGVNTNVDLVKLIEAGRYISNFL
GKPTESKVNRAISDRFKKHNDIVKIASCDI
>ARD71181.1 desaturase [*Spodoptera exigua*] des5
MAQTIQTTTILEQKEEKTVTLLVPQAGKRKFEFVYQNLITFAYWHIAGLYGLYLCFTSAKW
ATILFSFILFVIAEIGITAGAHRLWSHKSYKVKLPLEILLMVMNSIAFQNTVIDWVRDHRLH
HKYSDTDADPHNASRGFFYSHIGWLFVRKHPEVKKRGKELDMSDIYNNPVLRFQRKYAV
PFIGAVCFGLPTLIPVYCWGESWTNAWHITMLRYIMNLNATFLVNSAAHIYGKRPYDKKIL
PAQNIGVSIATFGEGFHNYHHVFPWDYRAAELGNNGLNLTTKFIDFFAWIGWAYDLKTVS
KEMIKQRSKRTGDGTNLWGLEDKDTPENLKNIKGE
>AFO38465.1 delta-9 desaturase 14-26 [*Spodoptera exigua*] des7
MAAMSGAPLLLANATLTSKLQDDDHRYAEPMKKNRDYEWQVVWRNVFAFVYLHAAAL
YGFYLMFTGKVRIWTILFGLLFAIMAGMGVTAGAHRLWAHRSYKARWPLRLFLALMQT
MAFQNHIYEWVRDHRVHHKFTETDADPHNARRGFFFSHIGWLMVRKHKDVFEKGATVD
MSDLEKDPIVMFQKKTYMVLMPLLCFVIPAWIPCYFWGENPWYSWYVASITRYTVALHFT
WLVNSAAHIWGNRPYDKNIGATDNKAVAICAFGEGWHNYHHVFPWDYKAAELGNYSTN
LSTALIDFAAKHGLAYDLKTVSAEMIRQRVNRTGDGSHPWTKDSQEEEHYHPENPVWGW
EDTDMTEEEKQFAEIVHRKTE
>ARD71184.1 desaturase [*Spodoptera exigua*] des10
MPPNADWEEVAQQRAIDKNTHVSFPQLKYPSLRDDGLRDPVQWLAGKAMDDGAEGLW
RIHDKLYDLTRFIKRHPGGEEWLELTQGTDITEAFESHHLNPSTEKILTQYYIRDAKTPRNSP
FTFKEDGFYKTLKRAAFEELKKIPKDASRTANNITDGLFVSLLISSAMASWVTNYYAVKF
WYTYASINLAILTVCCHNYIHRKTNWRMYLFNMSMWSYRDFRVSHVLSHHLYTNTLMD
LELSSLEPFLFYTPRKDKPLHAKLGFITEIFFFPFVFFLSFVKRFLSIFLHQGFFKSHYRWHD
AIGLLLPLWMIIASGAPILDVISMWLWINCTGSLIFFSIAVNAAHHHPDAIKDGDQPASETPD
WGMHQVEALLDRKDVNGNVFAVMTLFGDHCLHHMFPTLDHSVLKYMHPLFIDLCEKYQ
ANYRVSTQFKLVLGQIKETMRTEFKTKND
>ARD71182.1 desaturase [*Spodoptera exigua*] des8
MGARVSRTDFEWVYTEEPHASRRKIILEKYPQIKKLFGYDPNFKWVVTGMVLVQIISLPFV
TQLSWPMMLVVAYCFGGVINHSLMLAIHEIAHNLAFGHNRPLANKLFGFFANLPIGLPVSI
SFKKYHLEHHRYQGNEVIDTDLPTLLEAKLFCTTGGKLLWLFLQPFFYSFRPLVVRPKPPT
PMELINLVIQLFFDAVVIKLWGWKAIGYLLIGALMAMGVHPVAGHFVAEHYMFKKGYET
YSYYGPLNWITFNVGYHNEHHDFPAVPGSKLPEVRRIAPEFYDTLPHHDSWSKVLYDFVM

DPDIGPYARIKRKHHGLDS
>ARD71183.1 desaturase [*Spodoptera exigua*] des9
MPPQGQTGGSWVLYETDAVNVDSEAPVIVPPSAEKREWKIVWRNVILMGLLHIGGVYGA
YLFLTTAMWRTSLFAVFLYICSGLGITAGAHRLWAHKSYKARLPLRLLLTLFNTLAFQDAVI
DWARDHRMHHKYSETDADPHNATRGFFFSHVGWLLVRKHPQIKAKGHTIDLSDLKNDPI
LRFQKKYYLILMPLICFILPCYIPTLWGESLWNAYFVCSIFRYVYVLNVTWLVNSAAHLWG
AKPYDKNINPVETKPVSLVVLGEGFHNYHHTFPWDYKTAELGDYSLNLTKLFIDFMAAIG
WAYDLKTVSPDVIQKRVKRTGDGSHAVWGWDDHEVQQEDKKLAAIINPDKTE
>AFO38464.1 delta-9 desaturase 16-18 [*Spodoptera exigua*] des11
MAPNISEDVNGVLFESDAATPDLALARPPVQKADNKPKQLVWRNIILFAYLHLAALYGGY
LFLFSAKWQTDIFAYILYVISGLGITAGAHRLWAHKSYKAKWPLKVILIIFNTVAFQDAAM
DWARDHRMHHKYSETDADPHNATRGFFFSHIGWLLVRKHPDLKEKGKGLDMSDLLADP
LLRFQKKYYLVLMPLACFVMPTMIPVYLWGETWTNAFFVAAMFRYAFILNVTWLVNSAA
HKWGDKPYDKSIKPSENMSVAMFALGEGFHNYHHTFPWDYKTAELGNNKLNFATAFINF
FAKIGWAYDMKTVSDDIVKNRVKRTGDGSHHLWGWGDKNQPKEEIEAAIRINPKDD
>ARD71185.1 desaturase [*Spodoptera exigua*] des12
MVEVKEAAPVAEEQKLSKSREANWPAVLFFIHIHLLSLYGTYLLLFEVKMMTLLFFILLTS
VALLGMTTGAHRLWAHQAYQASTGLKITLMLFQTLAGIGSIYDWVKYHRFHHAHFATDV
DPYDYNQGFIHSHLLTRLRKLSPHQEKLMQSIDMSDLEKDSVVMFQKRLYWVLYAIIFALL
PLNAPLEYWDDTILSSAFVIGFLRYLVVLHGSWLIESAICVWGLKPGEKSPPDTNAVFILTK
TFWPHYHYLVPYDYKSGEYGTYDSGCSTAFIRVWAALGLATKLRTVETVTIQKALAESAR
TKKDLKACIDAAVNNQKLPDEHYLKRA
>ATJ44457.1 desaturase IPAE [*Helicoverpa armigera*]
MDNNTNKIKRGITLSEIVQNFEKNLGFKNEIKWSSFIFITLYHVLAVYWCYHYAFPVKWQS
LIFALIMYVASGFGITGGAHRLWTHKSYKARLPLKLFLLLCFSSAGQNSLLHWVRDHRVH
HKYSDTDADPHNANRGLFFSHIGWLMMKKNNEVILRGKQMDMSDIENDPVIQFYERNFT
LLKLTFCYILPTMIGVVLWNEDWKCAVAWQCFIRFLGMFHSELTVNSLAHAYGYRPYNKN
IIPAENRFVATCTLGEGWHNYHHAFPFDYKAAEHFDVLNFATTFIRFFEKIGWAYDLREAS
ADVINSMAKRLGDGTPVHFPVATDTLNERAAG
>ATJ44456.1 desaturase PDSN [*Helicoverpa armigera*]
MVEVKEAVPENEEPISRSREANWPAVLFFIHIHLLSLYGLWLLIFEVKLLTLLFFFTLTSVAIL
GMTTGAHRLWAHGAYKASTGLRVTLMLFQTLAGVGSIYDWVQYHRLHHAHFATEDDPY
DYNKGFVYAHFLTRLRKLSPQQEKLKSAIDMSDLENDSIVMFQKKAYWFLYAILFALLPLN
APLEYWDDTVLSSVFVVGFLRYLIVLHASWLIDSAISVWGLRPGEKSPPDSNTVFILTKTF
WPHYHYLVPYDYKSGEYGTYDCGCSSAFIRVWAALGLATNLQTVETHTIQKALADAART
QKDLKTCIDEAVVNQKLPEEHYLKRG
>ATJ44455.1 desaturase QPGE [*Helicoverpa armigera*]
MGAVQEDPPTMGSEVKTEEVHKPNVPSDHKWEIVWGRAVFAVLVHLAGFYGAFLFFTAA
KWQTCLFTIFLHVAMAISVTAGAHRLWAHRAYKAKLPLRILLLTFFTMAFQNTLIVWARD
HRAHHKYVDTDADPHNSNRGFFFSHIGWLLVRRHPEVRAHKVDLSDLFADPLLKFQNNY
YVWMLPFLVVLTPIYIPTLWGEKKMVALFVCLFLRYLLTIHVFFVVNSVAHMFGTRPYDK
NIQPGESKLVSFFASGEGFHNYHHAFPWDYRTAELGGYLFNTSRLFIDLMAKIGWAYDLKS
VPTDMIERRVKRTGDGSHPVWGWDDPDMSAEDRKLATIINENKES
>ATJ44454.1 desaturase LPAQ [*Helicoverpa armigera*]

MAQSYQSTTVLSEEKEPTLAHLVPQASPRKYQIVYPNLITFGYWHIAGLYGLYLCFTSAK
WSTILFSYILFVLAEIGITAGAHRLWAHKTYKAKLPLEILLMVFNSIAFQNSAIDWVRDHRL
HHKYSDTDADPHNASRGFFYSHVGWLLVRKHPEVKKRGKELNMSDIYNNPVLRFQKKY
AIPFIGAVCFALPTMIPVYFWGETWSNAWHITMLRYIMNLNVTFLVNSAAHIWGNKPYDA
KILPAQNVAVSVATGGEGFHNYHHVFPWDYRAAELGNNSLNLTTKFIDLFAAIGWAYDLK
TVSEDMIKQRIKRTGDGTDLWGHEQKCDEVWDVKDKSS
>ATJ44453.1 desaturase MPVE [*Helicoverpa armigera*]
MAPNISEDVNGVLFESDAATPDLALSTPPVQKADNRPKQLVWRNILLFAYLHLAALYGGY
LFLFSAKWQTDIFAYILYVISGLGITAGAHRLWAHKSYKAKWPLRVILVIFNTVAFQDAAM
DWARDHRMHHKYSETDADPHNATRGFFFSHIGWLLVRKHPDLKEKGKGLDMSDLLADPI
LRFQKKYYLILMPLACFVMPTVIPVYFWGETWTNAFFVAAMFRYAFILNVTWLVNSAAH
KWGDKPYDKSIKPSENLSVAMFALGEGFHNYHHTFPWDYKTAELGNNKLNFTTTFINFFA
KIGWAYDLKTVSDDIVKNRVKRTGDGSHHLWGWGDENQSKEEIDAAIRINPKDD
>ATJ44452.1 desaturase NPVE [*Helicoverpa armigera*]
MPPQGQTGGSWVLYETDAVNEDTDAPVIVPPSAEKREWKIVWRNVILMGMLHIGGVYG
AYLFLTTAMWRTCIFAVVLYICSGLGITAGAHRLWAHKSYKARLPLRLMLTLFNTLAFQDA
VIDWARDHRMHHKYSETDADPHNATRGFFFAHVGWLLVRKHPQIKAKGHTIDLSDLKSD
PILRFQKKYYLFLMPLVCFILPCYIPTLWGESLWNAYFVCSIFRYVYVLNVTWLVNSAAHL
WGAKPYDKNINPVETRPVSLVVLGEGFHNYHHTFPWDYKTAELGDYSLNLTKLFIDTMA
AIGWAYDLKTVSTDVIQKRVKRTGDGSHPVWGWDDHEVHQEDKKLAAIINPEKTE
>ATJ44451.1 desaturase KSVE [*Helicoverpa armigera*]
MAPAQQNLEMCDENMHSELKIRHPTYKNDKVGQFENNNTVLRDSASEVKSDSDFDLKK
YEAMEFKAQIRWPDLTVQVLLHLVSIYGLYLMISNQVKLLTILFALGTIYTSGFGITAGVHR
LWSHRAYRARLPLRILLAILFTITGQRDIYIWALDHRVHHKYSETVADPHDVRRGFWFAHV
GWLVLTPHPAVENRRIALRPTCADLLADPVVRLQKKFFIPLFALLNIALPIWVPWYCWSET
LVNSFVISFVTRFTITLNIAFSVNSFAHMWGNKPYDRFIKSVENSLVSLAALGEGWHNYHH
VFPWDYRTSELGKLNISTGFIDFFARIGWAYDLKAATYDMISKRAQRCGDGTFGEDEEPYP
TSEHCHSE
>ATJ44450.1 desaturase GATD [*Helicoverpa armigera*]
MAAMSSTPLLLANTMLSSKLQDHDDLRYAEPRKPNRDYEWQVVWRNVLAFVYLHVSAV
YGFYLMFTGKVKLYTILFGLLFAIMSGMGVTAGAHRLWAHRSYKARWPLRVFLALMQTM
AFQNHIYEWVRDHRVHHKFTETDADPHNAKRGFFFSHIGWLMVRKHKDVFEKGATVDM
SDLEQDPIVMFQKKTYLVVMPILCFIIPAWIPVHFWDENPWTSWYTAAITRYTVALHFTWL
VNSAAHIWGNRPYDKNIGATDNKMVAICAFGEGWHNYHHVFPWDYKAAELGDYSTNLS
TALIDFAAKHGYAYDLKTVSAEMIRKRVNRTGDGSHPWTKGKVEGDHYHPENPVWGWE
DTDMTEEEKQFAEIVHRKTE
>ATJ44449.1 desaturase MPVE [*Helicoverpa armigera*]
MAPITYTETELIEQPLHTNDYIQYKLHHPAKDTGETRTNGTLYQISPYDQMLNPKEPKFLA
PLRRLEKRMGFVTPIRWVNTIAITAFHIIGVLWFLRFVYFIDKPFKWQTLIFGYLVGQVAGF
GVTGGAHRYWCHRSYKATLPLQWILIICYSTAGQNTIYEWVRDHRVHHKFSETTADPHDA
NRGFLFSHVGWLMMKKHPNVLRQGAKLDLSDITNDPLIQFHTKYFLLFKIVFCFLIPSVIP
ALCWGECWEISVMSQSVLRYLLSLNFTWSVNSFAHLWGNKPYDKNIMPVENWGVSIVA
MGEGWHNYHHTFPWDYKAAELGIPMNLTTTLLNHFASIGWAYDLKEASPSLVRSVAKAR
GEPRDD

>AKU76404.1 acyl-CoA desaturase 5 [*Helicoverpa armigera*]
MAQSYQSTTVLSEEKEPTLTHLVPQASPRKYQIVYPNLITFGYWHIAGLYGLYLCFTSAKW
ATILFSYILFVLAEIGITAGAHRLWAHKTYKAKLPLEILLMVFNSIAFQNSAIDWVRDHRLH
HKYSDTDADPHNASRGFFYSHVGWLLVRKHPEVKKRGKELNMSDIYNNPVLRFQKKYAI
PFIGAVCFALPTMIPVYFWGETWSNAWHITMLRYIMNLNVTFLVNSAAHIWGNKPYDAKI
LPAQNVAVSVATGGEGFHNYHHVFPWDYRAAELGNNSLNLTTKFIDLFAAIGWAYDLKTV
SEDMIKQRIKRTGDGTDLWGHEQKCDKGSGVNDKLS
>AKU76401.1 acyl-CoA desaturase 2 [*Helicoverpa armigera*]
MPPQGQTGGSWVLYETDAVNEDTDAPVIVPPSAEKREWKIVWRNVILMGMLHIGGVYG
AYLFLTTAMWRTCIFAVVLYICSGLGITAGAHRLWAHKSYKARLPLRLMLTLFNTLAFQDA
VIDWARDHRMHHKYSETDADPHNATRGFFFAHVGWLLVRKHPQIKAKGHTIDLSDLKSD
PILRFQKKYYLFLMPLVCFILPCYIPTLWGESLWNAYFVCSIFRYVYVLNVTWLVNSAAHL
WGAKPYDKNINPVETRPVSLVVLGEGFHNYHHTFPWDYKTAELGDYSLNLTKLFIDTMA
AIGWAYDLKTVSTDVIQKRVKRTGDGSHPVWGWDDHEVHQADKKLAAIINPEKTE
>AKU76400.1 acyl-CoA desaturase 1 [*Helicoverpa armigera*]
MAPNISEDVNGVLFESDAATPDLALSTPPVQKADNRPKQLVWRNILLFAYLHLAAQYGGY
LFLFSAKWQTDIFAYILYVISGLGITAGAHRLWAHKSYKAKWPLRVILVIFNTVAFQDAAM
DWARDHRMHHKYSETDADPHNATRGFFFSHIGWLLVRKHPDLKEKGKGLDMSDLLADPI
LRFQKKYYLILMPLACFVMPTVIPVYFWGETWTNAFFVAAMFRYAFILNVTWLVNSAAH
KWGDKPYDKSIKPSENLSVAMFALGEGFHNYHHTFPWDYKTAELGNNKLNFTTTFINFFA
KIGWAYDLKTVSDDIVKNRVKRTGDGSHHLWGWGDENQSKEEIDAAIRINPKDD
>AKU76406.1 acyl-CoA desaturase 7, partial [*Helicoverpa armigera*]
HLMCAIGIGAGSHRIWTHRCFKARTPLRIVLMLWQTMGFQDCIFEWARDHRTHHKYADT
DADPHNAERGLFFSHMGWLCCKKSPEVIEGGKRIDLSDLYADPVVMFQKKHYMKMMPL
LCFVLPTVVPVYFWGETWMNAFFIPTILRYTCGINVVWSVNSFAHTFGYRPYDKSLNPRE
NIGVWMICVEGFHNYHHTFPWDYRATELPLYNMLTPTIVFIELMAKIGQAYDLKYVSPEII
KQRAHRTGDGTHHLWGWDDPEFTEKLKEKYGAVSHS
>AKU76405.1 acyl-CoA desaturase 6 [*Helicoverpa armigera*]
MAPAQQNLEMCDENMHSELKIRHPTYKNDKVGQFENNNTVLRDSASEVKSDSDFDLKK
YEAMEFKAQIRWPDLTVQVLLHLVSIYGLYLMISNQVKLLTILFALGTIYTSGFGITAGVHR
LWSHRAYRARLPLRILLAILFTITGQRDIYIWALDHRVHHKYSETVADPHDVRRGFWFAHV
GWLVLTPHPAVENRRIALRPTCADLLADPVVRLQKKFFIPLFALLNIALPIWVPWYCWSET
LVNSFVISFVTRFTITLNIAFSVNSFAHMWGNKPYDRFIKSVENSLVSLAALGEGWHNYHH
VFPWDYRTSELGKLNISTGFIDFFARIGWAYDLKAATYDMISKRAQRCGDGTFGEDEEPYP
TSEHCHSE
>AKU76403.1 acyl-CoA desaturase 4 [*Helicoverpa armigera*]
MAAMSSTPLLLANTMLSSKLQDHDDLRYAEPRKPNRDYEWQVVWRNVLAFVYLHVSAV
YGFYLMFTGKVKLYTILFGLLFAIMSGMGVTAGAHRLWAHRSYKARWPLRVFLALMQTM
AFQNHIYEWVRDHRVHHKFTETDADPHNAKRGFFFSHIGWLMVRKHKDVFEKGATVDM
SDLEQDPIVMFQKKTYLVVMPILCFIIPAWIPVHFWDENPWTSWYTAAITRYTVALHFTWL
VNSAAHIWGNRPYDKNIGATDNKMVAICAFGEGWHNYHHVFPWDYKAAELGDYSTNLS
TALIDFAAKHGYAYDLKTVSAEMIRKRVNRTGDGSHPWTKGKVEGDHYHPENPVWGWE
DTDMTEEEKQFAEIVHRKTE
>AKU76402.1 acyl-CoA desaturase 3 [*Helicoverpa armigera*]

MAPITFTETELIEQPLHTNDYIQYKLHHPAKDTGETRANGKLYQISPYDQMLNPKEPKFLA
PLRRLEKRMGFVTPIRWVNTIAITAFHIIGVLWFLRFVYFIDKPFKWQTLIFGYLVGQVAGF
GVTGGAHRYWCHRSYKATLPLQWILIICYSTAGQNTIYEWVRDHRVHHKFSETTADPHDA
NRGFLFSHVGWLMMKKHPNVLRQGAKLDLSDITNDPLIQFHTKYFLLFKIVFCFVIPSVIP
ALCWDECWEISVMSQSVLRYLLSLNFTWSVNSFAHLWGNKPYDKNIMPVENWGVSIVA
MGEGWHNYHHTFPWDYKAAELGIPMNLTTTLLNYFASIGWAYDLKEASPSLVRSVAKAR
GEPRDD
>AKU76414.1 acyl-CoA desaturase 8 [*Helicoverpa assulta*]
MGARVSRTDFEWVYTEEPHASRRKIILEKYPQIKKLFGYDPNFKWVVTAMVLIQIISLPFV
VQLSWPVMLVVAYCFGGVINHSLMLAIHEIAHNLAFGHNRPLANRLFGFFANLPIGLPVSI
SFKKYHLEHHRYQGDEVIDTDLPTLLEAKLFCTTGGKLAWLFLQPFFYSFRPLIVRPKPPTP
MELINLVIQLFFDAIIIKLFGWKALGYLIFGAVMAMGVHPVAGHFVAEHYMFKKGYETYS
YYGPLNWITFNVGYHNEHHDFPAVPGSKLPEVRRIAPEFYDNLPHHDSWSKVLYDFVMDP
DIGPYARMKRKHKGLDS
>AKU76412.1 acyl-CoA desaturase 6 [*Helicoverpa assulta*]
MAPAQQNLEMCDENMHSELKIRHPTYKNDKVGQFENNNTVLRDSASEVKSDSDFDLKK
YEAMEFQAQIRWPDLTVQVLLHLVSIYGLYLMISNQVKLLTILFALGTIYTSGFGITAGVHR
LWSHRAYRARLPLRILLAVLFTITGQRDIYIWALDHRVHHKYSETVADPHDVRRGFWFAH
VGWLVLTPHPAVENRRIALRPTCADLLADPVVRLQKKFFIPLFALLNIALPIWVPWYCWSE
TLVNSFVISFVTRFTITLNIAFSVNSFAHMWGNKPYDRFIKSVENSLVSLAALGEGWHNYH
HVFPWDYRTSELGKLNISTGFIDFFARIGWAYDLKAATYDMISKRAQRCGDGTFGEDEEPY
PTSEHCHSE
>AKU76413.1 acyl-CoA desaturase 7, partial [*Helicoverpa assulta*]
LFFIHIHLLSLYGLWLLIFEVKLLTLLFFFILTSVAILGMTTGAHRLWAHGAYKASTGLRVTL
MLFQTLAGVGSIYDWVQYHRLHHAHFATEDDPYDYNKGFVYAHFLTRLRKLSPQQEKLK
SAIDMSDLENDSIVMFQKRAYWFLYAILFALLPLNAPLEYWDDTVLSSVFVVGFLRYLIVL
HASWLIDSAISVWGLRPGEKSPPDSNTVFILTKTFWPHYHYLVPYDYKSGEYGTYDCGCS
SAFIRVWAALGLATNLQTVETHTIQKALADAARTQKDLKTCIDEAVVNQKLPEE
>AKU76411.1 acyl-CoA desaturase 5 [*Helicoverpa assulta*]
MAQSYQSTTVLSEEKEPTLTHLVPQASPRKYQIVYPNLITFGYWHIAGLYGLYLCFTSAKW
ATILFSYILFVLAEIGITAGAHRLWAHKTYKAKLPLEILLMVFNSIAFQNSAIDWVRDHRLH
HKYSDTDADPHNASRGFFYSHVGWLLVRKHPEVKKRGKELNMSDIYNNPVLRFQKKYAI
PFIGAVCFALPTMIPVYFWGETWSNAWHITMLRYIMNLNVTFLVNSAAHIWGNKPYDAKI
LPAQNVAVSVATGGEGFHNYHHVFPWDYRAAELGNNSLNLTTKFIDFFAAIGWAYDLKTV
SEDMIKQRIKRTGDGTDLWGHEQKCDKGSGVNDKL
>AKU76410.1 acyl-CoA desaturase 4 [*Helicoverpa assulta*]
MAAMSSTPLLLANTMLSSKLQDHDDLRYAEPRKPNRDYEWQVVWRNVLAFVYLHVSAV
YGLYLMFTGKVKLYTILFGALFAIMSGMGVTAGAHRLWAHRSYKARWPLRVFLALMQT
MAFQNHIYEWVRDHRVHHKFTETDADPHNAKRGFFFSHIGWLMVRKHKDVFEKGATVD
MSDLEQDPIVMFQKKTYLVVMPILCFVIPAWIPVHFWDENPWTSWYTAAITRYTIALHFT
WLVNSAAHIWGNRPYDKNIGATDNKMVAICAFGEGWHNYHHVFPWDYKAAELGDYST
NLSTALIDFAAKHGYAYDLKTVSADMIRKRVNRTGDGSHPWTKGKVEGDHYHPENPVW
GWEDTDMTEEEKQFAEIVHRKTE
>AKU76409.1 acyl-CoA desaturase 3 [*Helicoverpa assulta*]

MAPITFTETELIEQPLHTNDYIQYKLHHPAKDTGETRANGKLYQISPYDQMLNPKEPKFLA
PLRRLEKRMGFVTPIRWVNTIAITAFHIIGVLWFLRFVYFIDKPFKWQTLIFGYLVGQVAGF
GVTGGAHRYWCHRSYKAKLPLQWILILCYSAAGQNTIYEWVRDHRVHHKFSETTADPHD
ANRGFLFSHVGWLMMKKHPHVLRQGAKLDLSDITNDPLIQFHTKYFLLFKIVFCFVIPSVI
PALCWDECWEISIMSQSVFRYLLSLNFTWSVNSFAHLWGNKPYDKNIMPVENWGVSIVA
MGEGWHNYHHTFPWDYKAAELGIPMNLTTTLLNYFASIGWAYDLKEASPSLVRSVAKAR
GEPRED
>AKU76408.1 acyl-CoA desaturase 2 [*Helicoverpa assulta*]
MPPQGQTGGSWVLYETDAVNEDTDAPVIVPPSAEKREWKIVWRNVILMGMLHIGGVYG
AYLFLTTAMWRTCIFAVVLYICSGLGITAGAHRLWAHKSYKARLPLRIMLTLFNTLAFQDA
VIDWARDHRMHHKYSETDADPHNATRGFFFAHVGWLLVRKHPQIKAKGHTIDLSDLKSD
PILRFQKKHYLFLMPLVCFILPCYIPTLWGESLWNAYFVCSIFRYVYVLNVTWLVNSAAHL
WGAKPYDKNINPVETRPVSLVVLGEGFHNYHHTFPWDYKTAELGDYSLNLTKLFIDTMA
AIGWAYDLKTVSTDVIQKRVKRTGDGSHPVWGWDDHEVHQEDKKLAAIINPEKTE
>AKU76407.1 acyl-CoA desaturase 1 [*Helicoverpa assulta*]
MAPNISEDVNGVLFESDAATPDLALSTPPVQKADNRPKQLVWRNILLFAYLHLAALYGGY
LFLFSAKWQTDIFAYILYVISGLGITAGAHRLWAHKSYKAKWPLRVILVIFNTVAFQDAAM
DWARDHRMHHKYSETDADPHNATRGFFFSHIGWLLVRKHPDLKEKGKGLDMSDLLADPI
LRFQKKYYLILMPLACFVMPTVIPVYFWGETWTNAFFVAAMFRYAFILNVTWLVNSAAH
KWGDKPYDKSIKPSENLSVAMFALGEGFHNYHHTFPWDYKTAELGNNKLNFTTTFINFFA
KIGWAYDLKTVSDDIVKNRVKRTGDGSHHLWGWGDENQSKEEIDAAIRINPKDD
>ATJ44515.1 desaturase QPGE, partial [*Helicoverpa assulta*]
MFGTRPYDKNIQPGESKLVSLFASGEGFHNYHHAFPWDYRTAELGGYLFNTSKLFIDLMA
KIGWAYDLKSVPSDMIERRVKRTGDGSHPVWGWDD
>ATJ44514.1 desaturase PDSN [*Helicoverpa assulta*]
MVEVTEAVPENEEPISRSREANWPAVLFFIHIHLLSLYGLWLLIFEVKLLTLLFFFILTSVAILG
MTTGAHRLWAHGAYKASTGLRVTLMLFQTLAGVGSIYDWVQYHRLHHAHFATEDDPYD
YNKGFVYAHFLTRLRKLSPQQEKLKSAIDMSDLENDSIVMFQKRAYWFLYAILFALLPLNA
PLEYWDDTVLSSVFVVGFLRYLIVLHASWLIDSAISVWGLRPGEKSPPDSNTVFILTKTFW
PHYHYLVPYDYKSGEYGTYDCGCSSAFIRVWAALGLATNLQTVETHTIQKALADAARTQ
KDLKTCIDEAVVNQKLPEEHYLKRG
>ATJ44513.1 desaturase LPAQ [*Helicoverpa assulta*]
MAQSYQSTTVLSEEKEPTLTHLVPQASPRKYQIVYPNLITFGYWHIAGLYGLYLCFTSAKW
ATILFSYILFVLAEIGITAGAHRLWAHKTYKAKLPLEILLMVFNSIAFQNSAIDWVRDHRLH
HKYSDTDADPHNASRGFFYSHVGWLLVRKHPEVKKRGKELNMSDIYNNPVLRFQKKYAI
PFIGAVCFALPTMIPVYFWGETWSNAWHITMLRYIMNLNVTFLVNSAAHIWGNKPYDAKI
LPAQNVAVSVATGGEGFHNYHHVFPWDYRAAELGNNSLNLTTKFIDFFAAIGWAYDLKTV
SEDMIKQRIKRTGDGTDLWGHEQKCDKGSGVNDKLS
>ATJ44512.1 desaturase NPVE [*Helicoverpa assulta*]
MPPQGQTGGSWVLYETDAVNEDTDAPVIVPPSAEKREWKIVWRNVILMGMLHIGGVYG
AYLFLTTAMWRTCIFAVVLYICSGLGITAGAHRLWAHKSYKARLPLRIMLTLFNTLAFQDA
VIDWARDHRMHHKYSETDADPHNATRGFFFAHVGWLLVRKHPQIKAKGHTIDLSDLKSD
PILRFQKKHYLFLMPLVCFILPCYIPTLWGESLWNAYFVCSIFRYVYVLNVTWLVNSAAHL
WGAKPYDKNINPVETRPVSLVVLGEGFHNYHHTFPWDYKTAELGDYSLNLTKLFIDTMA

AIGWAYDLKTVSTDVIQKRVKRTGDGSHPVWGWDDHEVHQEDKKLAAIINPEKTE
>ATJ44511.1 desaturase MPVE [*Helicoverpa assulta*]
MAPITFTETELIEQPLHTNDYIQYKLHHPAKDTGETRANGKLYQISPYDQMLNPKEPKFLA
PLRRLEKRMGFVTPIRWVNTIAITAFHIIGVLWFLRFVYFIDKPFKWQTLIFGYLVGQVAGF
GVTGGAHRYWCHRSYKAKLPLQWILILCYSAAGQNTIYEWVRDHRVHHKFSETTADPHD
ANRGFLFSHVGWLMMKKHPHVLRQGAKLDLSDITNDPLIQFHTKYFLLFKIVFCFVIPSVI
PALCWDECWEISVMSQSVFRYLLSLNFTWSVNSFAHLWGNKPYDKNIMPVENWGVSIVA
MGEGWHNYHHTFPWDYKAAELGIPMNLTTTILNYFASIGWAYDLKEASPSLVRSVAKAR
GEPRED
>ATJ44510.1 desaturase GATD [*Helicoverpa assulta*]
MAAMSSTPLLLANTMLSSKLQDHDDLRYAEPRKPNRDYEWQVVWRNVLAFVYLHVSAV
YGLYLMFTGKVKLYTILFGALFAIMSGMGVTAGAHRLWAHRSYKARWPLRVFLALMQT
MAFQNHIYEWVRDHRVHHKFTETDADPHNAKRGFFFSHIGWLMVRKHKDVFEKGATVD
MSDLEQDPIVMFQKKTYLVVMPILCFVIPAWIPVHFWDENPWTSWYTAAITRYTIALHFT
WLVNSAAHIWGNRPYDKNIGATDNKMVAICAFGEGWHNYHHVFPWDYKAAELGDYST
NLSTALIDFAAKHGYAYDLKTVSADMIRKRVNRTGDGSHPWTKGKVEGDHYHPENPVW
GWEDTDMTEEEKQFAEIVHRKTE
>ATJ44509.1 desaturase KSVE [*Helicoverpa assulta*]
MAPAQQNLEMCDENMHSELKIRHPTYKNDKVGQFENNNTVLRDSASEVKSDSDFDLKQ
YEAMEFQAQIRWPDLTVQVLLHLVSIYGLYLMISNQVKLLTILFALGTIYTSGFGITAGVHR
LWSHRAYRARLPLRILLAVLFTITGQRDIYIWALDHRVHHKYSETVADPHDVRRGFWFAH
VGWLVLTPHPAVENRRIALRPTCADLLADPVVRLQKKFFIPLFALLNIALPIWVPWYCWSE
TLVNSFVISFVTRFTITLNIAFSVNSFAHMWGNKPYDRFIKSVENSLVSLAALGEGWHNYH
HVFPWDYRTSELGKLNISTGFIDFFARIGWAYDLKAATYDMISKRAQRCGDGTFGEDEEPY
PTSEHCHSE
>ATJ44508.1 desaturase KPSE [*Helicoverpa assulta*]
MAPNISEDVNGVLFESDAATPDLALSTPPVQKADNRPKQLVWRNILLFAYLHLAALYGGY
LFLFSAKWQTDIFAYILYVISGLGITAGAHRLWAHKSYKAKWPLRVILVIFNTVAFQDAAM
DWARDHRMHHKYSETDADPHNATRGFFFSHIGWLLVRKHPDLKEKGKGLDMSDLLADPI
LRFQKKYYLILMPLACFVMPTVIPVYFWGETWTNAFFVAAMFRYAFILNVTWLVNSAAH
KWGDKPYDKSIKPSENLSVAMFALGEGFHNYHHTFPWDYKTAELGNNKLNFTTTFINFFA
KIGWAYDLKTVSDDIVKNRVKRTGDGSHHLWGWGDENQSKEEIDAAIRINPKDD
>AID66656.1 desaturase [*Agrotis segetum*]
MAPAQKNMEMCGEEMHSELKISPVTYKNGKGAHFENNNTVLRDSASVVTSDSDFDIKKY
EAMEFKAQWRWPDLAAQVFLHLVSIYGLYLIISNQLKLYTILFVFGTIYTSGFGITAGVHRL
WSHRAYRARLPLRVLLAILFTITGQRDIYTWALDHRVHHKYAETVADPHDIRRGFWFAHV
GWLVLTPHPAVEDRRIALRPTCADLLADPVVRLQKQFFIPMFALLNIGIPIFVPWYFWSETL
VNSFIVSFVLRFTITLNIAFCVNSFAHLWGNKPYDKFVKSVENSLVSLAALGEGWHNYHH
VFPWDYRTSELGKMNVSTGFIDLFAKIGWAYDLKAATYDMIAKRAKRSGDGTFGESEEPY
PTTEHCHAE
>AID66657.1 desaturase [*Agrotis segetum*]
MPPQGQTGGSWVLYETDAVNEDTDAPPVIVPPSAEKRVWKIVWRNVILMGLLHIGGVYG
AYLFLTKAMWTTCFFAVFLYICSGLGITAGAHRLWAHKSYKARMPLRLLLTLFNTLAFQDA
VIDWARDHRMHHKYSETDADPHNATRGFFFAHVGWLLVRKHPQIKAKGHTIDLSDLKSD

PILRFQKKHYLILMPLVCFVLPSYIPTLWGESLWNAYFVCSIFRYVYVLNVTWLVNSAAHL
WGAKPYDKNINPVETKPVSLVVLGEGFHNYHHTFPWDYKTAELGDYSLNITKLFIDTMA
AIGWAYDLKTVSTDVIQKRVKRTGDGSHAVWGWDDKEVHQEDKKLADIINPEKTE
>AID66658.1 desaturase [*Agrotis segetum*]
MGAKVSRTDFEWVYTEEPHASRRKIILEKYPQIKKLFGYDPNFKWVVTAMVLVQIISLPFV
TQLSWPMMLLVAYCFGGVINHSLMLAIHEIAHNLAFGHNRPLANRLFGFFANLPIGLPVSIS
FKKYHLEHHRYQGNEVIDTDLPTLLEAKLFDTTGGKFLWLILQPFFYSFRPLIVRPKPPTPM
ELINLVIQLFFDAIVIKLWGWKALGYLIFGAVMAMGVHPVAGHFVAEHYMFKKGYETYSY
YGPLNWITFNVGYHNEHHDFPAVPGSKLPEVRRIAPEFYDNLPHHDSWTKVLYDFVMDPE
IGPYARIKRKELGLKS
>AID66659.1 desaturase [*Agrotis segetum*]
MAQGVQTTTILREEEPSLTFVVPQEPRKYQIVYPNLITFGYWHIAGLYGLYLCFTSAKWQT
ILFSFMLVVLAELGITAGAHRLWAHKTYKAKLPLQIILMILNSIAFQNSAIDWVRDHRLHH
KYSDTDADPHNATRGFFYSHVGWLLVRKHPEVKRRGKELDMSDIYNNPVLRFQKKYAIP
FIGAMCFGLPTFIPVYFWGETWSNAWHITMLRYILNLNITFLVNSAAHIWGYKPYDIKILPA
QNIAVSIVTGGEGFHNYHHVFPWDYRAAELGNNYLNLTTKFIDFFAWIGWAYDLKTVSSD
VIKSKAERTGDGTNLWGLEDKGEEDFLKIWKDN
>AID66660.1 desaturase [*Agrotis segetum*]
MVEVHDAVSGPEETKLRKGRDANWPAVLFFIHIHLLSLYGTWLLIFEAKLMTIIFFVALTSV
AILGMTTGAHRLWAHGAYKASTGLRIALMLCQTLAGVGSIYDWVQYHRLHHAHFATDD
DPYNYNKGFFYSHFLTRLRKLSPHQEKLKDAIDMSDLEKDSVVMFQKRLYWGLHAVLFL
LLPLNAPLEYWDDTILNSVFVIGFLRYLIVLHASWLIESAICVWGLRPGEKSPPDSNTVFIIS
KTFWPHYHYLVPYDYKSGEYGTYDSGCSTAFIRVFAALGLATNLQTVETAAAQKALADA
AKTKKDLKTCIDAAVS
>AID66661.1 desaturase [*Agrotis segetum*]
MTHVTKTINSKLIKSLIYRSLSTAVPQIRIYEVGPRDGLQNESKFVPTEIKIELINKLAAAGIK
DIESASFVSPKWVKQMSDGVDVMKNVPRAPGVNYPVLVPNLKGYDTAKQCNVEEVAIFP
AGSEGFSQKNLNCSVEEGLKRFKLVADQAVKDGLRVRGYVSCVVGCPYDGPVHPKGIAKI
TEQLFEMGCYEVSLGDTIGVGTAGSVKRLMQEVLTVAKPEQLALHFHDTYGQALSNLLA
GLEFGIKTVDSSISGLGGCPYARGASGNLATEDLVYFLYGLGLNTHIDLVKLIEAGRYISNY
LGKPTESKVNRAIGDRFKNHNDIAKLASCDV
>AID66662.1 desaturase [*Agrotis segetum*]
MAPNTERHQISFPRLEYPILREVMPKSAHNWLKGKRMQDGAEDLWRIHDNLYDLTDFVT
AHPGGTYWISVTKGTDITEAFETHHLKGVAETLLPNYYIRKATKPRSHPFTFKEDGFYKTL
KLKVMAQLPNIPKDLRKKSDFVSDSLLLALIILSPMSCWGWTQSFLLGASLTILNGLVLSSII
TCAHNYFHRSDSWRMYLFNLGGMSYSDWRISHAMSHHLHTNTAQDVELSMIEPFLQFLP
YKDKPIWAQMGAFYYPFVYGASFLVLFVTELVLCATNHEGKSLSWKNLIPFTIPTWMYLM
GGLPLHWTIAIWLLTMIPASLFFVIYGLTAGHHSHRNFFEGDVPRDENIDWGLHQLDTIVE
RIDYAGNHFKSITRFGDHALHHLFPTLDHAELNALYPTLFEHCEKFESQLKTNTFYEALISA
SKQLIRKRPNNFRDKKF
>AID66663.1 desaturase, partial [*Agrotis segetum*]
MPPNSEWEEGAQQRALDKNTHVTFPQLKYPSLRDESLRDPVQWLAGKAMDDGAEGLW
RIHDKLYDLTRFIKRHPGGEEWLELTQGTDITEAFESHHLNPSTEKMLTQYYIRDAKTPRNS
PFTFKEDGFYKTLKREAFEQLKKIPKDASKTADNITDGLFLSLLISSALSCWVTNEYAAKF

WYAYASVNLAFLTVACHNFIHRKTNWRMYLFNMSMWSYRDFRVSHVLSHHLYTNTLMD
LEISSLEPILFYNPRKDKPLHAKLGFITEIFFFPFIFLISFVKRFLSIFLRQGFFKSHYRWHDAI
GLLLPVW
>AID66664.1 desaturase [*Agrotis segetum*]
MEEENQYDKEDQPTIAVPFKKVYLWPNILFLIYAHIAGVYGLYLLFTSAKWTTIVFFMISFII
NTIGITAGAHRLYSHKSYKAKKPLQVFLMLCHCHAYQRTLATWIRDHRLHHKYSDTDAD
PHNINRGFFFAHYGWLLVKSHPEVEKRRATVDMRDVYSNEVVMFQKRHKEWMLPLFAFI
IPTVIPWLLGDTFSNSWHLNIFRFILTSNLTFLTNSLAHSWGYKPYDKTMRASQNLAVVAFN
FGEGYHNFHHAFPWDYRSAELGNNKWNLVAKVIDFFEMLGLAYDLKMASPGMIQTRRK
RTGDGTDLWGREIKEEY
>AID66665.1 desaturase, partial [*Agrotis segetum*]
GKVKLWTVLFGVSFAIMSGMGVTAGAHRLWAHRSYKARWPLRLFLAFMQTMAFQNHIY
EWVRDHRVHHKFTETDADPHNAKRGFFFSHIGWLMVRKHKDVFEKGASVDMSDLEKDP
IVMFQKKTYLVVMPILCFIIPAWIPVYFWGENAWISWYVASITRYTVALHFTWLVNSAAHI
WGNRPYDKNIGATDNKAVAICAFGEGWHNYHHVFPWDYKAAELGNYSTNLSTALIDFAA
KHGLAYDLKTVSAEMIRQRVNRTGDGSHAWSKKSLEEEEHYHPENPVWGWEDADMTEE
EKQFAEIVHRKTE
>AGR49311.1 acyl-CoA delta 9 desaturase [*Agrotis ipsilon*]
MAPNISDDVNGVLFESDAATPDLALASPPVQKADNRPKQYVWRNILLFAYLHAAALYGG
YLFLTSAKWQTDVFAYILYVMSGLGITAGAHRLWAHKSYKAKWPLKVILIIFNTIAFQDAA
MDWARDHRMHHKYSETDADPHNATRGFFFSHIGWLLVRKHPDLKEKGKGLDMSDLQA
DPILRFQKKYYLLLMPLACFVMPTVIPVYFWGETWNNAFFVAAMFRYAFILNVTWLVNS
AAHKWGDKPYDKSIKPSENMSVAMFALGEGFHNYHHTFPWDYKTAELGNNKLNFTTTFI
NFFAKLGWAYDMKTVSDDIVKNRVKRTGDGSHHLWGWGDKNQSKEEIASAIRINPKDD
>AGR49312.1 acyl-CoA delta 11 desaturase [*Agrotis ipsilon*]
MAQGVQTTTIFREEEPALTFVVPQEPRKYQIVYPNLITFGYWHIAGLYGLYLYFTSAKWQT
MLFSFMLVVLAELGITAGAHRLWAHKTYKAKLPLQIILMVLNSIAFQNSAIDWVRDHRLH
HKYSDTDADPHNANRGFFYSHVGWLLVRKHPEVKRRGKELDMSDIYNNPVLRFQKKYAI
PFIGAMCFGLPTFIPVYCWGETWTNAWHITMLRYIVNLNITFLVNSAAHIWGNKPYDSKIL
PAQNIAVSIVTGGEGFHNYHHVFPWDYRAAELGNNYLNLTTKFIDFFAWIGWAYDLKTVS
SDVIKSRAQRTGDGTNLWGLEDKGEEEILKIWKDN
>AGR49312.1 acyl-CoA delta 11 desaturase [*Agrotis ipsilon*]
MAQGVQTTTIFREEEPALTFVVPQEPRKYQIVYPNLITFGYWHIAGLYGLYLYFTSAKWQT
MLFSFMLVVLAELGITAGAHRLWAHKTYKAKLPLQIILMVLNSIAFQNSAIDWVRDHRLH
HKYSDTDADPHNANRGFFYSHVGWLLVRKHPEVKRRGKELDMSDIYNNPVLRFQKKYAI
PFIGAMCFGLPTFIPVYCWGETWTNAWHITMLRYIVNLNITFLVNSAAHIWGNKPYDSKIL
PAQNIAVSIVTGGEGFHNYHHVFPWDYRAAELGNNYLNLTTKFIDFFAWIGWAYDLKTVS
SDVIKSRAQRTGDGTNLWGLEDKGEEEILKIWKDN
>AGR49314.1 desaturase, partial [*Agrotis ipsilon*]
MAPAQKNMEMCGEEIHSELKISPVTYKNGKGAHYENNNTVLRDSASEVTSDSDFDIKKY
EAMEFNAQWRWPDLAA
>AGR49315.1 acyl-CoA desaturase GATD, partial [*Agrotis ipsilon*]
LWAHRSYKARWPLRLFLAFMQTMAFQNHIYEWVRDHRVHHKFTETDADPHNAKRGFFF
SHIGWLMVRKHKDVFEKGASVDMSDLEKDPIVMFQKKTYLVVMPILCFIIPAWIPVYFWG

ENAWISWYVASITRYTVALHFTWLVNSAAHIWGNRPRFERPSSA
>ACX53794.1 desaturase [*Heliothis virescens*]
MAQSYQSTTVLSEEKEPTLTLVVPQAAPRKYQIVYPNLITFGYWHIAGLYGLYLCFTSAK
WATILFSYFLFVVAEIGITAGAHRLWAHKTYKAKLPLEILLMVLNSIAFQNSAIDWVRDHR
LHHKYSDTDADPHNASRGFFYSHVGWLLVRKHPEVKKRGKELNMSDIYNNPVLRFQKK
YAIPFIGAVCFVLPTLIPVYCWGETWSNAWHITMLRYIMNLNVTFLVNSAAHIWGYKPYD
AKILPAQNVAVSVATGGEGFHNYHHVFPWDYRAAELGNNSLNLTTKFIDFFAWIGWAYDL
KTVSEDMIKLRTKRTGDGTDLWGHEQKYDEVLDVKDK
>AGO45844.1 acyl-CoA desaturase HvirIPAE [*Heliothis virescens*]
MDNNTNKIKRGITLSEIVQNFEKHLGFKNEIKWSSFIFITLYHVLAVYWCYHYAFPVKWQS
LVFALIMYVASGFGITGGAHRLWTHKSYKAKLPLKLFLLLCFSSAGQNSLLHWVRDHRVH
HKYSDTDADPHNANRGLFFSHIGWLMMKKNSEVILRGKQMDMSDIENDPVIQFYERNFT
WLKLTFCYILPTMIGVVLWNEDWKCAVAWQCFIRFLGMFHSELTVNSLAHAYGYKPYNK
NIIPAENRFVATCTLGEGWHNYHHAFPFDYKAAEHFDVLNFATTFIRFFEKIGWAYDLREA
SADVINSMAKRLGDGTPVHFPVPTDTFNERAAG
>AGO45843.1 acyl-CoA desaturase HvirKPVE [*Heliothis virescens*]
MGLVQEEHSSTMDSDATAEEDHKSNVPSKWQWEIVWERVAFAIIMHIGGFYGAYLFFTEA
KWQTCLFTIFLHVAMATSVTAGAHRLWSHRAYKAKLPLKIILLTFFTMAYQNTVMVWAR
DHRAHHKYCDTDADPHNSNRGFFFSHIGWLLVRRHPEVRANKIDLSDLFEDPLLRFQNKY
YLWVVPFLTVLTPIYIPTLWGETKMVALFVCLFLRYIMTVHAFFIVNSVAHKWGTKPYDKS
IKPVETKLVSFLATGEGFHNYHHAFPWDYKAAELGGYLFNTSRLFIDLMAKIGWAYDLKS
VPSDMIERRVKRTGDGSHPVWGWDDPDLSAEDRKSAIVN
>AGO45842.1 acyl-CoA desaturase HvirKSVE [*Heliothis virescens*]
MAPAQQNIEMCDENMHSEIKIRHPAYKNDKVGQFENNNTVLRDSASEVKSDSEFDLKKY
EAMEFKAQIRWPDLTVQVLLHLVSIYGLYLMICNQVKLLTILFALGTIYTSGFGITAGVHRL
WSHRAYRARLPLRILLAILFTITGQRDIYIWALDHRVHHKYSETVADPHDVRRGFWFAHV
GWLVLTPHPAVENRRIALRPTCADLLADPVVRLQKKFFIPLFALLNIALPIWVPWYCWNET
LVNSFVISFVTRFTITLNIAFSVNSFAHMWGNKPYDRFIKSVENSLVSLAALGEGWHNYHH
VFPWDYRTSELGKLNISTGFIDFFARIGWAYDLKAATYDMISKRAQRCGDGTFGEDEEPYP
TSDHCHSE
>AGO45841.1 acyl-CoA desaturase HvirNPVE [*Heliothis virescens*]
MPPQGQTGGSWVLYETDAVNEDTDAPVIVPPSAEKREWKIVWRNVILMGMLHIGGVYG
AYLFLTTAMWRTCIFAVVLYICSGLGITAGAHRLWAHKSYKARLPLRLLLTLFNTLAFQDA
VIDWARDHRMHHKYSETDADPHNATRGFFFAHVGWLLVRKHPQIKAKGHTIDLSDLKSD
PILRFQKKHYLILMPLVCFVLPCYIPTLWGESLWNAYFVCSIFRYVYVLNVTWLVNSAAHL
WGAKPYDKNINPVETRPVSLVVLGEGFHNYHHTFPWDYKTAELGDYSLNLTKLFIDTMA
AIGWAYDLKTVSTDVIQKRVKRTGDGSHPVWGWDDHEVHQEDKKLAAIINPDKTE
>AGO45840.1 acyl-CoA desaturase HvirGATD [*Heliothis virescens*]
MAMSSTPLLMANTMLSSKLQDHEDIRYAEPRKPNRDYEWQVVWRNVLAFVYLHVAAVY
GLYLMFTFKVKLYTILFGALFAMMSGMGVTAGAHRLWAHRSYKARWPLRLFLALMQTM
AFQNHIYEWVRDHRVHHKFTETDADPHNAKRGFFFSHIGWLMVRKHKDVFEKGATVDM
SDLEQDPIVMFQKKTYLVVMPILCFVIPAWIPVHFWGENPWTSWYTAAITRYTIALHFTWL
VNSAAHIWGNRPYDKNIGATDNKMVAICAFGEGWHNYHHVFPWDYKAAELGDYSTNLS
TALIDFAAKHGYAYDLKTVSAEMIRKRVNRTGDGSHQWTKAKVDEDGHFHPENPVWGW

EQTDMTEEEKQFAEIAHRKTE
>AGO45839.1 acyl-CoA desaturase HvirLPAQ [*Heliothis virescens*]
MAQSYQSTTVLSEEKEPTLTLVVPQAAPRKYQIVYPNLITFGYWHIAGLYGLYLCFTSAK
WATILFCYFLFVVAEIGITAGAHRLWAHKTYKAKLPLEILLMVLNSIAFQNSAIDWVRDHR
LHHKYSDTDADPHNASRGFFYSHVGWLLVRKHPEVKKRGKELNMSDIYNNPVLRFQKK
YAIPFIGAVCFVLPTLIPVYCWGETWSNAWHITMLRYIMNLNVTFLVNSAAHIWGYKPYD
AKILPAQNVAVSVATGGEGFHNYHHVFPWDYRAAELGNNSLNLTTKFIDFFAWIGWAYDL
KTVSEDMIKLRTKRTGDGTDLWGHEQKYDEVLDVKDK
>AGO45838.1 acyl-CoA desaturase HvirKPSE [*Heliothis virescens*]
MAPNISEDVNGVLFESDAATPDLALATPPVQKADNRPKQLVWRNILLFAYLHLAALYGGY
LFLFSAKWQTDIFAYILYVISGLGITAGAHRLWAHKSYKAKWPLRVILVIFNTVAFQDAAM
DWARDHRMHHKYSETDADPHNATRGFFFSHIGWLLVRKHPDLKEKGKGLDMSDLLADPI
LRFQKKYYLILMPLACFVMPTVIPVYFWGETWTNAFFVAAMFRYAFILNVTWLVNSAAH
KWGDKPYDKSIKPSENLSVAMFALGEGFHNYHHTFPWDYKTAELGNNKLNFTTTFINFFA
KIGWAYDLKTVSDDIVKNRVKRTGDGSHHLWGWGDENQSKEEIDAAIRINPKDD
>LPAQ [*Helicoverpa zea*]
MAQSYQSTTVLSEEKELTLQHLVPQASPRKYQIVYPNLITFGYWHIAGLYGLYLCFTSAK
WATILFSYILFVLAEIGITAGAHRLWAHKTYKAKLPLEILLMVFNSIAFQNSAIDWVRDHRL
HHKYSDTDADPHNASRGFFYSHVGWLLVRKHPEVKKRGKELNMSDIYNNPVLRFQKKY
AIPFIGAVCFALPTMIPVYFWGETWSNAWHITMLRYIMNLNVTFLVNSAAHIWGNKPYDA
KILPAQNVAVSVATGGEGFHNYHHVFPWDYRAAELGNNSLNLTTKFIDLFAAIGWAYDLK
TVSEDMIKQRIKRTGDGTDLWGHEQNCDEVWDVKDKSS*
>PDSN [*Helicoverpa zea*]
SEFYSPTIFFCSFDLHTVTRSLFQHYPFFEDADGCKKLTNRSPDKMVEVKEAVPENEEPISR
SREANWPAVLFFIHIHLLSLYGLWLLIFEVKLLTLLFFFILTSVAILGMTTGAHRLWAHGAYK
ASTGLRVTLMLFQTLAGVGSIYDWVQYHRLHHAHFATEDDPYDYNKGFVYAHFLTRLRK
LSPQQEKLKSAIDMSDLENDSIVMFQKKAYWFLYAILFALLPLNAPLEYWDDTVLSSVFV
VGFLRYLIVLHASWLIDSAISVWGLRPGEKSPPDSNTVFILTKTFWPHYHYLVPYDYKSGE
YGTYDCGCSSAFIRVWAALGLATNLQTVEAHTIQKALADAARTQKDLKTCIDEAVVNQK
LPEEHYLKRG*
>NPAE [*Helicoverpa zea*]
MVCFSGFGITAGAHRYWAHKAFKATTPLRIIMLLGFASAGQNTIYQWVRNHRIHHKYSDT
ESDPHNRERGLFFSHIGWLLMKKKPEVTSKAKEIDMSDIENDALLTWHRKHLDIVNPLMT
FVIPTLIGMVLWGETWKAAVAWQCCIRFLFVYHSELTVNSLGHTIGYKPYDTSINPAENAII
SALTGGEGWHNFHHSFPFDYKAAEWSHTFDFTTDLIHFFEKFGWVYDKREVTKDFIKKY
AEQHAKFSS*
>GATD [*Helicoverpa zea*]
IFFFFFFNDTATTEIYTFVVLQSDDLSAIYGLYLMFTGKVKLYTILFGALFAIMSGMGVTAG
AHRLWAHRSYKARWPLRVFLALMQTMAFQNHIYEWVRDHRVHHKFTETDADPHNAKR
GFFFSHIGWLMVRKHKDVFEKGATVDMSDLEKDPIVMFQKKTYLVVMPILCFVIPAWIPV
HFWDENPWTSWYTAAITRYTVALHFTWLVNSAAHIWGNRPYDKNIGATDNKMVAICAFG
EGWHNYHHVFPWDYKAAELGDYSTNLSTALIDFAAKHGYAYDLKTVSADMIRKRVNRT
GDGSHPWTKGKVEGDHYHPENPVWGWEDTDMTEEEKQFAEIVHRKTE*
>QPVE [*Helicoverpa zea*]

MFIWCRDHRLHHRYSDTDGDPHNSKRGFFFCHMGWLMHKKHPYVIELGRRIDMSDMQS
DWMVMFQKKYYYPLYLLLAIFIPMYVPLYFFGEHWWHSLLVCYFLRYVFSLHGTWFVNS
IAHLYGTRPYDKNLQPVESWFVSVVTLGEGWHNYHHAFPWDYKAAELSYFINHSATFIEF
LDMIGLAYDLKTASPAMVLNRIARTGDGSHYLLGDEETRKAVTAWGPLHPLNPTYNSTLQ
PPSAVLKPEGLPLFHEKDVLKLKISRRSASTA*

>NPVE [*Helicoverpa zea*]

MPPQGQTGGSWVLYETDAVNEDTDAPVIVPPSAEKREWKIVWRNVILMGMLHIGGVYG
AYLFLTTAMWRTCIFAVVLYICSGLGITAGAHRLWAHKSYKARLPLRLMLTLFNTLAFQDA
VIDWARDHRMHHKYSETDADPHNATRGFFFAHVGWLLVRKHPQIKAKGHTIDLSDLKSD
PILRFQKKYYLFLMPLVCFILPCYIPTLWGESLWNAYFVCSIFRYVYVLNVTWLVNSAAHL
WGAKPYDKNINPVETRPVSLVVLGEGFHNYHHTFPWDYKTAELGDYSLNLTKLFIDTMA
AIGWAYDLKTVSTDVIQKRVKRTGDGSHPVWGWDDHEVHQADKKLAAIINPEKTE*

>KPSE [*Helicoverpa zea*]

MAPNISEDVNGVLFESDAATPDLALSTPPVQKADNRPKQLVWRNILLFAYLHLAALYGGY
LFLFSAKWQTDIFAYILYVISGLGITAGAHRLWAHKSYKAKWPLRVILVIFNTVAFQDAAM
DWARDHRMHHKYSETDADPHNATRGFFFSHIGWLLVRKHPDLKEKGKGLDMSDLLADPI
LRFQKKYYLILMPLACFVMPTVIPVYFWGETWTNAFFVAAMFRYAFILNVTWLVNSAAH
KWGDKPYDKSIKPSENLSVAMFALGEGFHNYHHTFPWDYKTAELGNNKLNFTTTFINFFA
KIGWAYDLKTVSDDIVKNRVKRTGDGSHHLWGWGDENQSKEEIDAAIRINPKDD*

>KSVE [*Helicoverpa zea*]

MAPAQQNLEMCDENMHSELKIRHPTYKNDKVDQFENNNTVLRDSASEVKSDSDFDLKK
YEAMEFKAQIRWPDLTVQVLLHLVSIYGLYLMISNQVKLLTILFALGTIYTSGFGITAGVHR
LWSHRAYRARLPLRILLAILFTITGQRDIYIWALDHRVHHKYSETVADPHDVRRGFWFAHV
GWLVLTPHPAVENRRIALRPTCADLLADPVVRLQKKFFIPLFALLNIALPIWVPWYSWSET
LVNSFVISFVTRFTITLNIAFSVNSFAHMWGNKPYDRFIKSVENSLVSLAALGEGWHNYHH
VFPWDYRTSELGKLNISTGFIDFFARIGWAYDCKFTMRDLL*

>DES9 [*Helicoverpa zea*]

MGARVSRTDFEWVYTEEPHASRRKIILEKYPQIKKLFGYDPNFKWVVTAMVLIQIISLPFV
VQLSWPVMLVVAYCFGGVINHSLMLAIHEIAHNLAFGHNRPLANRLFGFFANLPIGLPVSI
SFKKYHLEHHRYQGDEVIDTDLPTLLEAKLFCTTGGKLAWLFLQPFFYSFRPLIVRPKPPTP
MELINLVIQLFFDAIIIKLFGWKALGYLIFGAVMAMGVHPVAGHFVAEHYMFKKGYETYS
YYGPLNWITFNVGYHNEHHDFPAVPGSKLPEVRRIAPEFYDNLPHHDSWSKVLYDFVMDP
DIGPYARMKRKHKGLDS*

FAR

>AID66646.1 fatty acyl reductase, partial [*Agrotis segetum*]

DTDTDTVLMFFNFAVISSQRTPFPGWIENLNGPSGVIVGAGKGVLHVLSCGGGQRADLLP
VDLAIDTLLAAAWETAVDDLRETRVYNCSTCSNPITWGQFRSYMLSGVRAHPLDNALWY
PYGLIIEGTMMQKLLETVLQTTPLYLIHYVSKMCGMKARPSLSTVSNRLQAMNEALKFFA
LREWHFNTDNVQRLKQRLSPADAAVFNLDTSTIDWTEVCTDFVKGTRKYLLQEKDEDVE
QAQRRMHMLHMMHNATKLFLSIMLCRLAMRTTPAILRAIASLIRLRRKNTMLHTM

>AID66647.1 fatty acyl reductase [*Agrotis segetum*]

MVPRPMPPSPAEPLLPRFYAGRSILITGATGFMGKVLIERILSTCPDVGGLHLLMRDKKGHS
PQKRLAQLKQSQVFDNVRARNHRQLDKLFVISGDVSKPRLGMDSEAISQLKEVSIVFHSA
ATLKFDEPLRVAIDQNVRSVQRLLDICDELPNIEAFIHVSTAYSNAELTYVEERVYPPPVPLE

QAFTIADSVPEELLVKINAEYISPKPNTYTFTKALAENVVQEHGNKGYPVAIFRPTIVISSLR
HPYPGWIENLNGPSGVVVGAGKGLLHVFRCKDTAKADMLPVDMAIDTLLAVAWETAVD
RPEQVRVYNCSTYENPTTWGEFEGALRQYLRGHPLDNAYWYPSGLAVENKIAHKSLETL
LQTAPLHIAEYLTKILGIKTRMSLITVSQRLVAMGDVLKFFSIREWHFATDNVKKLHARLSP
QDAAIYNLDPHTINWSDHYENFIKGTRKYLLQEKDQDIDVAKKHLRKMYYVHQALLFFV
VALLCRFALLNPYIRTFVYRTFRMFMTILTAAYIRIQQS
>AID66648.1 fatty acyl reductase [*Agrotis segetum*]
MPVLTSREDEKLSVPEFYAGKSIFVTGGTGFLGKVFIEKLLYCCPDIDKIYMLIREKKNLSID
ERMSKFLDDPLFSRLKEERPGDLEKIVLIPGDITAPNLGLSAENERILLEKVSVIINSAATVK
FNEPLPIAWKINVEGTRMLLALSRRMKRIEVFIHISTAYSNASSDRIVVDEILYPAPADMDQ
VYQLVKDGVTEEETERLLNGLPNTYTFTKALTEHLVAEHQTYVPTIIIRPSVVASIKDEPIRG
WLCNWFGATGISVFTAKGLNRVLLGKASNIVDVIPVDYVANLVIVAGAKSGGQKSDELKI
YNCCSSDCNPVTLKKIIKEFTEDTIKNKSHIMPLPGWFVFTKYKWLLTLLTIIFQMLPMYLA
DVYRVLTGKIPRYMKLHHLVIQTRLGIDFFTSHSWVMKTDRVRELFGSLSLAEKHMFPCD
PSSIDWTDYLQSYCYGVRRFLEKKK
>AID66649.1 fatty acyl reductase [*Agrotis segetum*]
MAVEALTDSQLFESKQGGDITFMDMVDEQEPLGESQIQKLFAGSAVLLTGGTGFLGKLVV
EKLLRSCPDLKKIFLLARPKKNKDITKRLQEQFDDVLYDRLRKERPDFISKISIVEGDMGQP
ELGMCAEDRAKIMNEVDVIFHGAATVRFDEPLKTAVEINVRGTREMFKLARGCTKLKAF
VHISTAYSNCPQTSIDEKFYDSPLPGEKLIDLVETMDEQTINNITPGLLGNFPNTYAYTKAVA
ENIVLEYSKGLPVALFRPAIVIGTAKEPVAGWIDNVYGPTGVVVGAAVGLLHVLNCNPKV
KADLVPGDMVVSAAIAAAWRTARDYPTTCNHEDAPPADLPPPVYNYVSSEQKPLTWERF
MKYNEVYGFQVPTVQAIYYYVFMLTASKPLYAFYCFMMHWIPAYIVDAIAVLIGKKPMLR
KTYAKITKFSEVMAYFATREWKFDNSNTQRLFAEMCPADKKMFDFDMSALDWNDYFYS
YIRGVRVYLLKDPVNTVPAGLTKHNRLRFLHYTFCTILGLLFLRILWAVFSMIVGF
>AID66650.1 fatty acyl reductase [*Agrotis segetum*]
MNNILEESPLKQTPLTGEKMEKWVEAQLKGEHLDIDIYGKPSEQTIKELENVRNLSKELQ
DNLHELENSVRIAEVENQAMNPTAPILDFSEDHEFVPDNQNTYYAGEDKVDAKEEEKQKL
TKGKGPKTAIQQFYKDQCVFLTGGTGFLGKVLIEKLIRSCGDVDTVFVLARSKKGKDAQT
RLNDLLDEFLFQRAHEENPKGVHKVVPVIGDMELPGLGISDEDRKMLASKVSIIINVAATV
KFDEKLSVSTAINVKGTKEVLKLAKECRNLRAVTHVSTAFSNTHVKHIEEKFYEPPISVEA
LEAISDVDENLIESILPTLLGDRPNTYCFTKAIAEEAVRVFGEGLPLCILRPSIVVSTYEEPVR
GWTDSVYGPTGLVVGIGTGVLRTMYMDQQKVADMVPVDLCVNAILTSAWYTAKNYKEN
QTSDIPIYNFVSGAQNPITWGEFIERNRKHGIDKPTTKAVWYYGLNPTNNYYMFLFYNFFL
HYMPALFVDLYCTLTGKRRAMLKLYSKVMKLANILFYFSTQDWKFSDNNVRNMWNSLS
PDDRVVFPFSIGEMSWERMCETFLVGLRVYLVKDDLSTLPEARKKWTKLYYLHQLLKALT
IVVVLNLVYFVVKAVFALTHW
>AID66651.1 fatty acyl reductase [*Agrotis segetum*]
MTSEVNEWYKGRSVLVTGALGLMGKVLIEKLLYSVPDLGCVYALVRSKRGKSPETRIEEM
WKLPLFKRIREEKPHVMKKLIPVTGDIMFEELGINGSHLKEIYDEVSIVFHFAASLRLEAPL
KEGLEMNTRGTLRVLDMAKKMKKLVAFIHLSTAFCYPDYERMAEKVFDPPADPHEVLRA
AGWLTEEQLNLLAPSIYQKHPNSYTYSKRLAEALVRESYPQLPAVVVRPSIVTPSYKEPTPG
WVDNLNGPIGLMVGAGKGVIRSMHCYGHYHAEVIPVDIAINSIIIIAYKTGKDTQRQPEIPV
YNITTGDDRHTTWKEVLDIGKATVRKFPFEGPLWYPDGNIRHNKFIHDLCVFFYHIIPAYFI

DFLLLLFRQRRFMVRIQNRITIGLEVLQYFTTREWWFDTNNFKSLVGLLNPVDKQTFPMD
LTIIEDEPYIESCMIGGKLYCLKEKMENLPKARLQNHILYILDRLVSLFFYLVLVYWIVSYFE
PVRELLSYGGPAIRYLPLVGKAVFRDV
>AID66652.1 fatty acyl reductase [*Agrotis segetum*]
MKVLITGGTGFMGKVLVEKLLRKCPDIGQILLFVRSKKGKNPKQRLEEIFNGVLFEKVRA
MRGGVEPLIEKVTLVTGDVSEPDLGMSEEDRQMVMKDVDIIIHAAATIRFDEELKKAVLL
NVRGTKLMVELAKTCKKLKLFIHISTSYCHLHEKLLEEKAYPPPADPHQIIQAVEWMDEET
ITALTPKLLNKLPNSYAFTKALGEALVVESMEHIPAMVLRPSIVIPIWQEPVPGWTDNINGP
TGLLIGAGKGVIRSMYCKSNSYADYLPVDVFINGIMIAAWNYMKNGETKANIINFTSSAEI
KVTWSEMIDAGREIIMNRVPLNGVVWYPGGSMKHSRLYHNICVFFFHWIPAFIIDTLLFCL
GYKPVLCRVQRRITKGFEVFEYYTNNQWDFKSDIAQKVRTKLNPRERREYKVDAVGLDIS
KYFEDCIRAARVFILKEYDDTLPAARRHMRVMYWVDVIVRCLFWGLMLYWISGWFSSSN
SIVADQHTLPTVIAMDA
>AID66653.1 fatty acyl reductase [*Agrotis segetum*]
MPDVGKIYLLMRPKKGKEISERLQEFPKNLIFEKLLETNSTDIFQKLIPIAGDVGEDNLGLS
PQDRQTLVDNVNVVIHSAATLDFQESLRPTVNINLLGTRRIMELCKDAKNLKVMIHVSSA
YVNSFLTEAHEKVYEAPEDAEKVISLVGTLNDQALLEIEPKLLKSHPNTYTFTKHLAEHEV
VKCADLFPCTIVRPTMIVATWKEPVPGWTCSKVGPQGFLMGAAKGVVRRLPLAKENIAD
YIPVDVVVNQLLVAGWEASKSNSGLSVYHCSSSTCKPFTWSMLDSTVNSMLHKYPLKSA
VWYPCLQFVPSLLMFRISAIFVHFFPALLLDMMLRLTGGRPILIRLHKNVWNSLSRLERFIF
SEWKFYNPNTLELCKKLNQTDKELFYIDLTMLHWVEYFKSLHLGVRRYLNREKESTLPAA
RKKDMVLLMFHVIWQLFIIGLLWYIFACLTGLTLAHSAFIAPVIYILFSFL
>AID66654.1 fatty acyl reductase [*Agrotis segetum*]
MAPSMSIAEYYAGKTLFITGATGFMGKVMVEKLLRCCPDVKKMYLLMRPKKGHSSKERL
DDLLSFRVFDRLKAESPKIFDKLHVIPGDILSEDLGMSNEDRMLIQNEAQMIFHCAACVRF
DMFLRDAVKMNTMGTKMVLELAEGVKNLEAFVHVSTSYCRCELELFEEKLYPSKHRPEH
VMHCVGWMDDELLGHMQPKIIEPQPNTYAYTKSLTEDLVSQYEGKFPIVIARPSIVAAAYK
EPMPGWVDNLNGPTGLLVGAGKGVIRTMHCNENYAADVVPVDVAVNACIILGYLTGMEK
PKQISICNITQSEINPITWGQALDMGRIHVQEFPFTVCLWYPGGSPKSSRLAHQLALFFTHL
LPAYFVDLLMFLMGKKTFMIKIQKRINYGLEVLQYYTTKEWHFKNDNFVALQNRISEKDN
ETFYTDMKDMNWSMYIRNYIKGAREYCCKEDPSTLPAARRLQRQLYYLDKAVQIMVGLL
ISYITYYYLNMLYSLISA
>AID66655.1 fatty acyl reductase [*Agrotis segetum*]
MNNMFRLRILINKDSVVLSKRMKINHVLGYSSMPENLTKATEYTTTYQPIADFYAGKSVF
VTGGTGFLGKVYLEKLLYSCKKVDKVYLLVREKKGHNITKRIEDLFANPLFSRLKKTNPE
YFKKVVPVSGDITLPNLGLTPKDEQTLIDKVSVVYHAAATVRFNEPLPVAMNINFEGTQK
VLELSRRMKNIEAFLYISTAYTQTQRKVLMETVYPPPAKEEDIYKFIEEFGNDAKETEKYLC
DHEKPNSYTFTKALAESYIAKNHGDVPAVIIRPSAVVSIKDEPLKSWLDNWFGLTFYFYTA
AKGWNRFNLGNSNNSVDLIPVDYVSNFTIIAGARAKSKYNEVQVFNTTSSSVNPVTFGEA
HKYFSEDIISRGKNDMPCPALIFVNSKAILNIGAFFCQTIPVHIADMWLKMTGKKPKFVKL
AADFTELARMADYFTSKNWQFRADRMRELFDSLSPEDKRIFPCDPTQIDWSEYLRDYGK
GVRKYLKPK
>AGR49316.1 fatty-acyl CoA reductase 6, partial [*Agrotis ipsilon*]
GCIRGRTQWCPRPVPQSPPPPLIPEFFAGREVFITGGTGFMGKVLVERLLWTCRDVARVHLL

LRRKQDCAPQKRLAQLKQSKVFDVIRAHCPQQLDKLNAVPGDVTQPRLGLDQHHLNQL
QQVSVVFHSAATLKFTEPLEAALAQNVRPVITLMDICDELPNMQVLVHVSTAYSNAELSV
VEERVYPSPTSPAQVLALVEHLPPELLADTTHKLISPKPNTYTFTKALAERAVAEHAAAAS
YATAIFRPTIVISSQRTPFPGWIENLNGPSGVMVGAGKGVLHVLSCDGGQRADMLPVDLAI
DTLLAVAWETAVDDLRETRVYNCSTCSNPITWEQFRSYMLSGVRAHPFDNAMWYPYGLIT
ESTMMQKLLETVLQTAPLYLIHYVSKMCGIKPRPSLSTVSKRMQAMNEALKFFALREWHF
NTDNVQRLKQRLSPADAAVFNLDPSTIDWTEVCTDFVKGTRKYLLREKDEDIEHAQRRM
HLLHMMHNATMLFLTVLLCRLAMRTTPAILRAIATLIRLRRKSSILHNI
>AGR49317.1 putative fatty acyl-CoA reductase, partial [*Agrotis ipsilon*]
ILKTMAVEALTNSQLFESKQGGDITFMDMVDEQEPLGDSQIQKLFAGSAVLLTGGTGFLGK
LVVEKLLRSCPDLKKIFLLARPKKNKDITKRLQEQFEDVLYDRLRKERPDFISKISIVEGDM
GQPELGMCAEDRAKIMNEVDVIFHGAATVRFDEPLKTAVEINVRGTREMFKLARGCTKL
KAFVHISTAYSNCPQTSIDEKFYDSPLPGEKLIDLVETMDEQTINNITPGLLGDFPNTYAYTK
AVAENIVLEYSKGLPVALFRPAIVIGTAKEPVAGWIDNVYGPTGVVVGAAVGLLHVLNCNP
KVIADLVPG
>AGR49318.1 putative fatty acyl-CoA reductase [*Agrotis ipsilon*]
MPDDSQVRAFYAGKNFFITGGTGFVGLCLIEKILRCMPDVGKIYLLMRPKKGKEISERLQE
FPKNLVFEKLLETNSTDIFQKLIPIAGDVGEDNLGLSPQDRQTLVDNVNVVIHSAATLDFQE
SLRPTVNINLLGTRRIMELCKDAKNLKVMIHVSSAYVNSFLTEAHEKVYEAPEDAEKVISL
VGTLNDEALLEIEPKLLKSHPNTYTFTKHLAEHEVVKCADLFPCTIVRPTMIVATWKEPVP
GWTCSKVGPQGFLMGAAKGVVRRLPLAKENIADYIPVDVVVNQLLVAGWEASKSNSGL
SVYHCSSSTCKPFTWSMLDSTVNSMLHKYPLKSAVWYPCLQFVPSLLMFRISAIFVHFFPA
LLLDLMLRLTGGRPILIRLHKNVWNSLSRLERFIFSEWKFYNPNTLELCKKLNQTDKELFY
IDISMLHWVEYFKTLHLGVRRYLNREKESTLPAARKKDMVLLMFHVIWQLFIIGLLWYIFA
SLTGLTLAHSAFIAPVIYILLSFL
>AGR49319.1 putative fatty acyl-CoA reductase [*Agrotis ipsilon*]
MTSEVNEWYKGRSVLVTGALGLMGKVLIEKLLYSVPDLGCVYALVRSKRGKSPETRIEEM
WKLPLFQRIREEKPHVMKKLIPVTGDIMYEELGINGSHLKEIYDEVSIVFHFAASLRLEAPL
KEGLEMNTRGTLRVLDMAKKMKKLVAFIHLSTAFCYPDYERMAEKVFDPPADPHEVLRA
AGWLTEDQLNLLAPSIYQKHPNSYTYSKRLAEALVRESYPQLPAVVVRPSIVTPSYKEPTP
GWVDNLNGPIGLMVGAGKGVIRSMHCYGHYHAEVIPVDIAINSIIIIAYKTGKDTQRQPEIP
VYNITTGDDRHTTWKEVLDIGKATVRKFPFEGPLWYPDGNIRHNKFIHDLCVFFYHIIPAYF
IDFLLFLFRQRRFMVRIQNRITIGLEVLQYFTTREWWFDTNNFKSLVGLLNPVDKQTFPMD
LTIIEDEPYIESCMIGGKLYCLKEKMENLPKARLQNHILYILDRLVSLFFYLVLVYWIVSYVE
PVRELLSYGGPAVRYLPLVGKAVFRDA
>AGR49320.1 fatty-acyl CoA reductase 3 [*Agrotis ipsilon*]
MATETLSSADIDALPDRIADTFSGMKVLITGGTGFMGKVLLEKLLRKCPDIGQILLFVRSK
KGKNPKQRLEEIFSGVLFDKVRTMRGGVEPLVEKVTLVTGDVSEPDLGMSEEDRQMVIK
DVDIIIHAAATIRFDEELKKAVLLNVRGTKLMVELAKTCKKLKLFIHISTSYCHLHEKLLEE
KAYPPPADPHQIIQAVEWMDEETITALTPKLLDKLPNSYAFTKALGEALVVESMQHIPAMV
LRPSIVIPIWQEPVPGWTDNINGPTGLLIGAGKGVIRSMYCKSNSYADYLPVDVFISGIMIV
AWNYLKTEIQRPTL
>AGR49321.1 fatty-acyl CoA reductase 6, partial [*Agrotis ipsilon*]
TNYRDISLRHPYPGWIENLNGPSGVVVGAGKGLLHVFCCKDTAKADMLPVDMAIDTLLA

VAWETAVDRPEQVKVYNCSTYENPTTWGEFESALRLYLRGHPLDNAYWYPSGLAVENKI
AHKSLETLLQTAPLHIAEYLTKLLGIKTRMSLITVSQRLVAMSDVLKFFSMREWHFKTDNV
KKLHARLSPQDAAIYNLDPQTINWSNHYENFIKGTRKYLLQEKDQDIDVAKKHLRKMYY
VHQGLLLFVLAILCRFALENQYIRAFVYRTFRMLLSILTAAYMRIQQS
>AGR49322.1 fatty-acyl CoA reductase 4, partial [*Agrotis ipsilon*]
VGWMDDELLGHMQPKIIEPQPNTYAYTKSLTEDLVSQYEGKFPIVIARPSIVAAAYKEPMP
GWVDNLNGPTGLLVGAGKGVIRTMHCNENYAADVVPVDVTVNACIILGYLTGIEKPKQIS
VCNITQSEINPITWGQALDMGRIHVQEFPFTVCLWYPGGSPKSSRLAHQLALFFTHLLPAY
FVDLLMFLMGKKTFMIKIQKRIN
>AGR49323.1 fatty-acyl reductase, partial [*Agrotis ipsilon*]
RMAVIISREEEKLSVPEFYAGKSIFITGGTGFLGKVFIEKLLYSCPDIDKIYMLIREKKNLSID
ERMTMFLDDPLFSRLKEKRPGDVEKIVLIPGDISSPNLGLSAENERILIENVSVIIHSAATIKF
NEPLPIAWKINVEGTRMLMDLSRRMKRIKVFIHISTAYSNANSERAAVEEILYPAPADMDQ
VYQLVKDGVTEEETEILLNGLPNTYTFTKALAEHLAAEHQVHVPTVIIRPSIVGSIKDEPIR
GWLCNWFGATGISVFTAKGLNRVLLGKASNIVDVIPVDYVANLVIVAGAKNGGEKSEELK
IYNCCSSDCNPVTVKKILKEFIDDTIKNKSHIMPLPGWFVFTKYKWLMTLLTIIFQMIPMYL
ADVYRVLMGKNPRYMKLHHLVIQTRLVINFFTFHSWVMKTDRARELFGSLSPVEKHMFP
WDPSGIDWTEYLQSYCYGVRHFLEKRK
>AGR49324.1 fatty-acyl CoA reductase 5, partial [*Agrotis ipsilon*]
EYRGSSPPLEPPIFNYVSSVENRITWGDFLQQNMQWIHCFPFSDAVWFISVRLTKSAFMNKI
YMFFLHLIPAILVDGLAICLGRKPKMLKVYRKIHKFSAVLSYFCTREIKFCNSRTRELWENF
>AGR49325.1 fatty-acyl-CoA reductase, partial [*Agrotis ipsilon*]
HVAASVRFDDTLKFAAKMNLRGTVEVMELAKEVRELSAVVHVSTSYSNTNRDPIEEVLYP
PHADWRDTLEVCEKIDPHALKVLTPKYLGELPNTYTFSKQLAENVVAEYKGILPIVIIRPS
>AGR49326.1 fatty-acyl CoA reductase 6 [*Agrotis ipsilon*]
MVPRPVSPSPAEPLLPRFYAGRSILITGATGFMGKVLIERILSTCPDVGRLHLLMRDKKGHS
PQKRLAQLKQSQVFDNVRARNHRQLDKLFVICGDVSKPHLGMDSEAIAELREVSIVFHSA
ATLKFDEPLRVAIDQNVRSVQRLLEICDKLPNIAAFIHVSTAYSNAELTHVEERVYPPPVPLE
QAFTIADSLPEELLVKINAEYISPKPNTYTFTKALAENVVQEHGIKGYPVAIFRPTIVISPSAI
HTLVG
>AGR49327.1 fatty-acyl-CoA reductase, partial [*Agrotis ipsilon*]
SKYSRVLQRKNCFYTGGSGFMGKVLVEKLLYSCPDLDRIYLLLRNKKGVKSEDRLNELFA
SPCFDRLRKERPEFRSKVFVIAG
>AGR49328.1 putative fatty acyl-CoA reductase, partial [*Agrotis ipsilon*]
EKCHAPPVDPDHVMKLVQWLDNNQLALLTPSLLGPHPNCYTFSKRLAENLVEQAHPHMP
VVIARPSIVCPAVKEPMPGWVDNLNGPVGVMLGAGKGVIRTMLCNGNLIAQVVPVDIAIN
AIIAIGMLEGSRTEKPESLPVYNVNNGHQKPTTWGDVLNVAKAYGRQYPLSWPLWYPNG
DITTNKFLHEYRRICYHLVPAYLIDLLLFLLGQKRIMVRIQERVSQGLEVLQYFTMRPWN
>ACX53790.1 fatty-acyl reductase [*Heliothis virescens*]
MVVLTSKETKPSVAEFYAGKSVFITGGTGFLGKVFIEKLLYSCPDIVNIYMLIREKKGLSVS
ERIKQFLDDPLFTRLKDKRPADLEKIVLIPGDITAPDLGITAANEKMLIEKVSVIIHSAATVK
FNEPLPTAWKINVEGTRMMLALSRRMKRIEVFIHISTAYTNTNREVVDEILYPAPADIDQVY
QYVKEGISEEDTEKILNGRPNTYTFTKALTEHLVAENQAYVPTIIVRPSVVAAIKDEPLKGW
LGNWFGATGLTVFTAKGLNRVIYGHSNYIVDLIPVDYVANLVIAAGAKSNTSSELKVYNC

CSSSCNPVKIGTLMSMFADDAIKQKSYAMPLPGWYIFTKYKWLVLLLTFLFQVIPAYITDLS
RHLVGKSPRYIKLQSLVNQTRSSIDFFTNHSWVMKADRVRELYASLSPADKYLFPCDPVNI
NWTQYLQDYCWGVRNFLEKKT
>ACX53770.1 fatty-acyl-CoA reductase, partial [*Heliothis virescens*]
VNTEVLEQFYPCPVHPDAIIGMAESMEDDRLNAITEHLITGWPNTYTFTKAIAEELVRASG
ADLPVCVVRPPIVTPSYYEPTPGWMDLTALSGPTGILAGIIMGILHVFYVDKDCKLPLTPVD
YVNNATIAAGWDAECRRKNGEKDIQVYTVSNKDNFITWDFIGVLMRTEGKRSPSPKALW
YCWLIETNSKVIYWILAFFLHYIPAYVMDAMGALLGNMPKEINSYVAVFRKIDKFALIYHF
FLSNEWGFKDDNVQ
>ACX53773.1 fatty-acyl-CoA reductase, partial [*Heliothis virescens*]
FSLNWNLQLNYFNQLNFSDKNHGFPRRTGLSDVPTIPEFYKGKTIFITGGSGFIGKVLIEKL
LYSCTDLDRIYLLLRNKKGVKSEDRLSQLYAKPCFQRLKAERPGVFESKVFVVSGNVMEI
GLGLTQEDRALLVNRVNVIFHVAASVRFDDTLKYSTQLNLRGTVEVMELAKEMRDLCSL
VHVSTSYANTNRDPIEEVLYPPLADWRETLDICENADEHTL
>ACX53775.1 fatty-acyl-CoA reductase, partial [*Heliothis virescens*]
MAEESQVRAFYAGKNFFITGGTGFVGLCLIEKILRCMPDVGKIYLLMRPKKGKEIAERLEE
FPKNPVFEKLLESNSTDIFKKLVPVAGDVGEVNLGLSPADRQMLIDNINVVIHSAATLDFQE
SLRPTVNINLLGTRRIMELCKDAKDLKVMIHVSSAYVNSYLTEAHEKV
>ALJ30235.1 putative fatty acyl reductase FAR1 [*Spodoptera litura*]
MAAETLTENQLFEAKQGGDITYMDMIEETQPLGDSQIQKLFAGSAVLLTGGTGFLGKLVV
EKLLRSCPDLKKIFLLARPKKNKTITKRLQEQFDDVLYDRLRKECPDFINKISIVEGDVGQL
DLGMCPEDRIKIMNEVEVIFHGAATVRFDEPLKTAVEINVRGTREMLKLARGCSKLKAFV
HISTAYSNCPQNMIGEKFYESPLPGDKLIDLVETMEEKVINNITPGLLGDFPNTYAYTKAVA
ENIVKEYSKGLPVALFRPSIVIGTSKEPVSGWIDNVYGPTGVVVGAAVGLLHVLNCNPKV
KADLVPGDMVVNACIATAWKTAKEYPSNHEDAPPPDLTPPVYNYVSSEQRPLTWEKFMN
YNEVYGFQVPTVQAIYYYLFHLTSSKFLYNLYCFLLHWIPAYIIDGIAVIIGKKPILRKAYKKI
TKFSEVMAYFATREWKFDNSNTQQLFKELCDADKYLFDFDMSALQWNEYFYNYIRGVRV
YLLKDPVDTVPEGLKKHHRLKFLHYTFCGILGLLFFRLLWAMISGILSF
>ALJ30236.1 putative fatty acyl reductase FAR2, partial [*Spodoptera litura*]
MLWVDFSVSLNLDFKIFATMVPRPVSPSPAEPLIPRFYAGRSILITGATGFMGKVLVERILST
CPEVGRLHLLMRDKKGHSPQKRLAQLKQSQVFDNVRARNHRQLDKLCVVSGDVSKPQL
GMDADAIAQLREVSIVFHSAATLKFDEPLPVAIDQNVRSVERLLDICDKLPNMEAFIHVST
AYSNAELTVVEERVYPAPVPLAQACTLAETLPVDLLGQINTQYISPKPNTYTF
>ALJ30237.1 putative fatty acyl reductase FAR3 [*Spodoptera litura*]
MVVLTSKEKSNMSVADFYAGKSVFITGGTGFLGKVFIEKLLYSCPDIDKIYMLIREKKGQSI
RERLTKIVDDPLFNRLKDKRPGDLGKIILIPGDITVPGLGISEENETILIEKVSVVIHSAATVK
FNEPLATAWNVNVEGTRMIMALSRRMKRIEVFIHISTAYTNTNRAVIDEVLYPPPADINDVH
QHVKNGVTEEETEKILNGRPNTYTFTKALTEHLVAENQSYMPTIIVRPSIVGAIKDDPIRGW
LANWYGATGLSVFTAKGLNRVIYGHSNHVVDLIPVDYVANLVIVAGAKTYHSNEVTIYNS
CSSSCNPITMKRLVGLFIDYTVKHKSYVMPLPGWYVYSNYRWLVFLVTLIFQVIPAYLGDI
GRRLLGKNPRYYKLQNLVAQTQEAVHFFTSHTWEIKSKRTSELFSSLSLTDQRMFPCDANR
IDWTDYITDYCSGVRQFLEKIK
>ALJ30238.1 putative fatty acyl reductase FAR4, partial [*Spodoptera litura*]
MAPSVNIAEYYAGKTLFITGATGFMGKVMVEKLLRDCSDVKKMYLLMRPKKGHSSKER

LDELLNFKIFDRLKAENPKLFEKLQVVAGDILLEDLGLSAEDRLLIQEEAQIIFHCAACVRF
DMFLRDAVKMNTMGTKKVLELAEGVKNLEAFVHVSTSYCRCELPLFEEKLYPSKHRPEH
VMHCVNWMDDELLGHLQPKIIEPQPNTYAYTKSLTEDLVSQYEGKFPIVIARPSIVAAAYK
EPLPGWVDNLN
>ALJ30239.1 putative fatty acyl reductase FAR5, partial [*Spodoptera litura*]
MDPALAVELEALSRQKAMFEATERGDSTVQQFYKDSTVFLTGASGFLGKQLVEKLFRACN
IRKIFILLRPKKNMTIQERLEEMLQDPVFNLVKKKKPDFAENIVPVKGDVAETKLGLSDTD
WTMITSEVDVIFHVAATTRFDEALRVSTMINIRGTRETVLLGKDCQKLKSFVYVSTTYSTA
TQANVDKEVMERFYPCPLPPELMIDMAENIDDERMEAIEANLIKGYPNTYTFTKSIAEEVV
RSLAGEMPTCIIRPAVVISSYREPVPGWADASCAFGASGLILGPATGLIHAIYASNDVKFSLV
PVDYVNNAILVAGWHTATEKPNDVQIYSVSSARNLFHWEPISSKIRDIGKVLPTPLAVWYT
FIINTSNKPLFFILTWLLHYIPGYILDAGCILLGKPTMFIKLYNRVNRSSLALSYFTTHTWVF
NDSNTDKLFNSLSKTDRLIFNFDTSDINISEFVTLWCVGLRKYLMKDGIKNTEYARKKQFL
LKYLHYVVSFMYVYVLFKITCLVCYLILCLFG
>ALJ30240.1 putative fatty acyl reductase FAR6 [*Spodoptera litura*]
MVPRPAPQSSTPPLIPEYFAGREVFITGATGFMGKVLVERLLWTCPDISRLHLLMRHKKDC
APDKRLALLKQSQVFDVVREQCPQQLDKLCVVPGDVTKRRFGFDQPTLNQLNQVSVVFH
SAATLKFDEPLSVAVEQNVRPVLTLMDICDQLPNMQVFIHVSTAYSNAELSTVEERVYPAP
VSPSHLLALVDALPATMLQEITPRLIAPKPNTYTFTKAVAESAVSERATSAHYACAIFRPSIV
VSSLRHPFPGWIENLNGPSGVIAGAGKGLLRVLRCGAQRRADMMPVDICIDTLIAVAWET
GVDNLREGRVYTCASSCHAATWGQFRARMLRLVREHPFDNVLWYPYGIICENTVIQKVL
ETVLQTAPLCLAHCVWRACGLKQKPSLWTACKRLQAMNHALQFFATRHWSFSTTHVQK
LADRLHGEDKLRYNLRPETIDWEQHCVDFVKGARRYLLRERDDDIHTARRRMKLLTVIH
NATLLFAIFFVCRLTIRTAPAILRGVAVLTRLRNKAIQES
>ALJ30241.1 putative fatty acyl reductase FAR7 [*Spodoptera litura*]
MSNPSIRDFYKGRNILVTGGTGFMGKVLIEKMLYSIPDLGNIYILMRPKRGKSVAQRIEDM
QRLRLFERIRTEKPDAFKKMKPLQGDVLFDNLGLSDSDIELLCNEVSVVFHFAATLRLEAP
LKDNVNMNTCGTQRALDIAKKFKKLDIFVHLSTAFCYPDYEVLGEKCFGPPVKPENVMK
LIQWLDDKQLALLTPSLLGPHPNCYTFSKRLAETIVEQAHDELPVVIARPSIVCPSLKEPVP
GWVDNLNGPVGVMLGAGKGVIRTMLCDGSLTAQVCPVDIAINGIIAIGMIEGNKKEKHTS
LPVYNVNNGHQKPTTWGDVLTIAKDYGRKYPLSWPLWYPNGDITTNAVLHEYRRIFYHL
VPAYLIDFLLFLLGQKRIMIRIQERISQGLEVLQYFTMRPWNFPCPNYDAIREKLSPEEQEIY
NTDTTDVDRHEYMKMCVEGGRVYCFKEDPNKIPYNRIYHRFLYVLDWFVKIMFWLFVLS
FIASWCGPIKTVFSFGEPIVKHLPFLGKVVSKVEEL
>ALJ30242.1 putative fatty acyl reductase FAR8 [*Spodoptera litura*]
MASETISSAELECLPDRIADTFSGMKVLITGGTGFMGKVLVEKLLRKCPDIDQILLFVRSKK
GKNPKQRLEEICSGVLFEKLREMRGGVEPLLEKVTLINGDVSEPDLAMSPEDRQMIIDQV
DIIIHAAATIRFDEELKKAVLLNVRGTKLMVELGKACKNLKVFIHISTAYCHLHEKLLEEKA
YSPPADPHQIIQAVEWMDEETIATMTPKLLNKLPNSYAFTKALAEALVVEAMEKANLPAM
VLRPSIVIPIWQEPVPGWTDNINGPTGLLIGAGKGVIRSMYCKSNSYADYLPVDVFINGIMI
VAWNYLKNGDKKCNIINFTSSAEIKVTWSEMIDAGREIIMNRVPLNGVVWYPGGSMKHSR
LYHNICVFFFHWIPAFIIDTLLFCLGYKPVLCRVQRRITKGFEVFEYYTNNQWDFKSDIAQT
LRQKLNAKERRDYKVDAVGLDISKYFEDCIRAARVFILKEYDDTLPAARRHMRIMYWVD
VIVNCLFWGFLLYWLSGWMTTSKAIVPDTASPTVIAMDA

>ALJ30243.1 putative fatty acyl reductase FAR9 [*Spodoptera litura*]
MHCNENYEADVVPVDVTVNACIILGYLTGMEKPKKINFCNITQSQINPITWGQALDMGRV
HVQEFPFTVCLWYPGGSAKSSWIAHQFALFFTHMLPAYFVDLLMFLMGKKTFMIKIQKRI
NYGLEVLQYYTTKEWHFTNDFFVSLQNRISKRDNEIFYTNMKEMDWSQYIRNYIRGARE
YCCKEDPSTLPAARRLQKQLYYLDKAVQIMVGLLVSYFIYYYFNMLYSMISS
>ALJ30244.1 putative fatty acyl reductase FAR10 [*Spodoptera litura*]
MNYAIEKSPLSQTPVTREKMEKWVDAQVKGEKADIDVYGKPTDKMLKELENVRNLSKE
LQDNLHELENSVRIAEVENQAMNPTAQILDFSEDHEFVPDNKDTYYAEEDKVDAKEEEK
KKLTKGKGPKTEIQEFYKDQCVFLTGGTGFLGKVLIEKIIRSCGDINTIYVLARNKKGKDPR
VRLHEMMDEFLFHRAHEENPKGIHKVVPILGDMELPGLGINEEDRKMLASKVTIIINAAAT
VKFDEKLSVSTAINVKGTKEVLKLAKECRNLKAITHVSTAFSNTQVKHIEEKFYEPPMSVE
ALEAISEVDEKLVESILPTLLGTRPNTYCFTKAVAEEAVRTYGEGLPICIVRPSIVVSTYEEPV
RGWTDSVYGPTGLVVGIGTGVLRTMYMDQEKVADMVPVDLCVNAILASAWHTAKNYK
ENQTSHIPIYNYVSGAQNPLTWGEFIERNRRYGIDKPTTKAVWYYGLNPTNNYYLFLFYNF
FLHYLPALMIDTYCAITGKRRAMLKLYSKVMKLANILFYFSTQDWKFSDMNVRNMWNS
LSDADRVVFPFSMGEMSWEYMCETFLVGLRVYLIKDDLSTLPEARKKWNKLYYLHQILKI
ITLSLVLYLTYFVLQPIIALVFN
>ALJ30245.1 putative fatty acyl reductase FAR11 [*Spodoptera litura*]
MSTEPGDGPLLPGFYAGRAVLITGGTGFLGKVLIERLLWTCPEVGEVHLLLRDKRGQPPRV
RLNQLKQSQAFDNVRAHCPGQLDKLRVVCGDVAQPRLGLDDAALLQLREVSLVFHSAAT
VKFWESLETALHQNVTSVVALMELCDQLPRLEALVHVSTAYSNAERRHIEERVYEAPAQL
AGLRAMLDALPPSLLDDLTARYIAPKPNTYVFSKAVAEATIAQRPRKHYATAIVRPSIVVSS
HRHPYPGWIENLAGPSGVVVGCGKGLVHAFNLDLAARADLIPVDITIDTMLAVAWEIATD
KSEEVRVYNSCSQQNPITWGTFRDRVVRNARAHPFDQLMYYPFTFGIKNRYVYKALELVL
QTIPLYVADYIARLCGIKLQLSLVTVSERLQAMNRVLAFFATREWYFSTRNVQALRRRLTR
ADQDIYNLDVTSVDWDEHVSNFVKGTRKFLLKEKDDNIPRAKKFVERLRRVHQFVLLML
SVLVYRFLMLLLPRFLFRSSPVLAGLAAPH
>ALJ30246.1 putative fatty acyl reductase FAR12 [*Spodoptera litura*]
MADPSQVRSFYAGKNFFITGGTGFVGLCLIEKILRCIPDAGKIYLLMRSKKGKEIADRLQEF
PKNPVFEKLLESNSADIFKKLIPIAGDVGEENLGLSPQDRQTIIDNVNVVIHSAATLDFQESL
RPTVNINLLGTRRVMQLCKDAKNLKVMIHVSSAYVNSYLTEAHEKVYEAPEDPEKVISLV
GTLNDEALLEVEPKLLKSHPNTYTFTKHLAEHEVVKCADLFPCTIVRPTMIVAAWKEPIPG
WTCSKVGPQGFLMGAAKGVVRRLPLAKENVADYIPVDVVVNQLLVAGWEAANSRSGLT
VYHCSSSTCHPFTWTMLDDTVNSMLHKYPLKSAVWYPHLKFVPSLLMFRISAIFVHFFPA
LLLDLMLRMTGGRPILIRLHKNVWNSLNRLERFIFSEWKFHNPNTLELATKLNQTDKELFF
IDISKLYWVEYFKTLHLGVRRYLNKEKESSLPAARKKDMVLLLFHVIWQLFIMGLVWYIF
ACFTGLTLAHSAWIAPIIYILFTFL
>ALJ30247.1 putative fatty acyl reductase FAR13 [*Spodoptera litura*]
MAARADMLPVDMAIDTLLAVAWETAVDRPEAVRVYNCSTCENPTTWRDFETALRHRLRV
NPLDNAFWYPSGFTVENKLTQKTLETILQTAPLHIAEYISKILGIKTRLSLITVSQRLIAMNE
VLRFFSVREWHFVTDNVRKLHARLTPQDAAIYNLDPQTINWNEHYCNFIIGARKYLLQEK
DQDINEAKKHLRRMYYLHHGVMFFVVTLLCRLALRNQYLRAFIYRTFRMLLTVAGSAYM
RIRQS
>ARD71186.1 fatty acyl reductase [*Spodoptera exigua*]

MAVETLTEHQLFEAKQGGDITYKDMVEESQPLGDSQIQKLFAGSSVLLTGGTGFLGKLIVE
KLLRSCPDLKKIFLLARPKKNKTITKRLQEQFDDVLYDRLRKECPDFINKISLVEGDVGQL
DLGMCPEDRIKIMNEVEIIFHGAATVRFDEPLKTAVEINVRGTREMFKLARGCSKLKAFVH
ISTAYSNCPQNMIGEEFYESPLPGDKLIDLVETMEEKVINNITPGLLGDFPNTYAYTKAVAE
NIVKEYSKGLPVALFRPSIVIGTSKEPVSGWIDNVYGPTGVVVGAAVGLLHVLNCNPKVIA
DLVPGDMVVNACIATAWKTAKEYPSNHEDAPPPDLTPPVYNYVSSEQRPLTWEKFMNYN
EVYGFQVPTVQAIYYYLFHLTSSKFLYNLYCFLLHWIPAYIIDGIAVIIGKKPMLRKAYKKIT
KFSEVMAYFATREWKFDNTNTQQLFKELCDADKYMFDFDMSSLEWNEYFYNYIRGVRV
YLLKDPVETVPQGLKKHNRLKYLHYTFCAILGLLFLRLLWAIFSGIFSF
>ARD71187.1 fatty acyl reductase [*Spodoptera exigua*]
MTYRQINEFDAEKFTAAAVPTSYVSVPDFYAGKSIFITGGTGFLGKVFLEKLLYSCKDVET
VYILIREKKGKTPQQRVEDLFNKPIFSRLKQKDSQCMKKVTAIIGDLSEPGLGISKDDEELL
LQKVSVVFHVAANVQFYKEFKEIINTNVGGTKYVLQLCQRIKDIKAFVHISTAYCHTDQK
VLEERIYPPPAELSEVLKFLQQPQHDKKQIKELFKKQPNSYTFAKALAETYIAENCGRVPTII
IRPSIISASLKEPLPGWVDSWNGATGLITASYNGANRVLLGEGGNFLDLIPVDFVANLAIVA
AAKCTSSLKVYNCCSSGCNPLTLKQLVSHMNNVGFDKNVSIIFTNNKASLSTLTFFLQTTP
SFTADMFLRVTGKSPRYMKIQSKLTIARNALNFFTCHSWVMKADNSRRLYASLSLHDRHT
FPCDPTDIDWKKYITIYIEGINQFLMKKRS
>ARD71188.1 fatty acyl reductase [*Spodoptera exigua*]
MVVLTSKEKSNMSVADFYAGKSVFITGGTGFLGKVFIEKLLYSCPDIDKIYMLIREKKGQSI
RERLTKIVDDPLFNRLKEKRPGDLDKIVLIPGDVTVPGLGISDENEAILIDKVSVVIHSAATV
KFNEPLATAWNVNVEGTRMIMALSRRMKRIEIFIHISTAYTNTNRAVVDEVLYPPPADINEV
HQYVKNGITEEETEKILNGRPNTYTFTKALTEHLVAENQAYMPTVIVRPSIVGAIKDDPIRG
WLANWYGATGLSVFTAKGLNRVIYGQSSHVVDLIPVDYVANLVIVAGAKTYRSNEVTIYN
SCSSSCNPITMERLVGLFIDDTVKHNSYVMPLPGWYVYSNYRWLVYLVTIIFQMIPAYLADI
GRRLLGKNPRYYKLQSLVAQTQEAVHFFTSHTWEIKSKRTSELFASLSHTDQRMFPCDAK
KIDWTDYITDYCSGVRQFLEKRK
>ARD71189.1 fatty acyl reductase, partial [*Spodoptera exigua*]
MCHTYCFYYIKKLIMAPSMNIAEYYAGKTLFITGATGFMGKVMVEKLLRDCSDVKKMYL
LMRPKKGHSSKERLDDILSFRIFDRLKAENPKLFEKLQVVAGDILSEDLGLSPEDRLLIQEE
AQIIFHCAACVRFDMFLRDAVKMNTMGTKKVLELAEGVKNLQAFVHVSTSYCRCELPIFE
EKLYPSKHRPEHVMHCVNWMDDELLGHLQPKIIEPQPNTYAYTKSLTEDLVSQYEGKFPIV
IARPSIVAAAYKEPLPGWVDNL
>ARD71190.1 fatty acyl reductase, partial [*Spodoptera exigua*]
MPTPLAVWYTFIINTSNKPLFFLLTWLLHYIPGYILDAGCILLGKPTMFIKLYNRVNRSSLAL
SYFTSRTWVFNDNNSDKLFQSLSKSDKLIFNFDTTDINIPEFVTIWCVGLRKYLMKDGIKN
TEYARKKQ
>ARD71191.1 fatty acyl reductase [*Spodoptera exigua*]
MVPRPAPQFFPTPPLIPEYFAGREVLITGATGFMGKVLVERLLWTCPDIGRLHLLMRHKRDV
APDKRLALLKQSQVFDVVRERCPQQLDKLCMVPGDVTKRRFGFDQSALNQLNQVSVVF
HSAATLKFDEPLSVAAEQNVRPVLTLMDICDQLPNMQVLVHVSTAYSNAELAEVEERVYP
APVTPEHLLALVDALPASMLQEITPRLIAPKPNTYTFTKAVAESAVSERAVTARYACAIFRPT
IVVSSLRHPFPGWIENLNGPSGVVAGAGKGLLRVLRCGAQRRADMMPVDICIDTLIAVAW
ETGIDNLREARVYQCASSSHAATWGQFRERMLRLVREHPFDNVLWYPYGVICENTVVQK

VLEAVLQTAPLCVAHCVARACGLKQKPSLWTACKRLQAMNQALQFFATRHWSFRTTRVQ
QLAHRLHPDDQKLYNLRPETIDWEQHCVDFVKGARRYLLRERDDDIHAARRRMRILYLIH
KATLLFAIFTMCRLTIRTAPAILWGVAALTRQRNKSALLS
>ARD71192.1 fatty acyl reductase [*Spodoptera exigua*]
MANPTIRDFYRGRNILVTGGTGFMGKVLIEKVLYSIPDVGNIYILMRPKRGKSVAQRIEDM
QRLRLFERIRSEKPDALKKMKPLQGDVLFENLGLSDSDIEKLSNEVSVVFHFAATLKLEAP
LKDNVNMNTCGTQRALEIAKKFKKLDIFVHLSTAFCYPDYEVLGERCYGPPVRPENVMK
LIQWLDDKQLALLTPSLLGPHPNCYTFSKRLAETIVEQAHDELPVVIARPSIVCPSVAEPMP
GWVDNLNGPVGVMLGAGKGVIRTMLCDGSLIAQVCPVDIAINAIIAIGMIEGNRKEKHTT
LPVYNVNNGHQKPTTWGDVLTIAKDYGRKYPLSWPLWYPNGDITTNAVLHEYRRIFYHL
VPAYLIDFLLFLLGQKRIMIRIQERISQGLEVLQYFTMRPWNFPCPNYDAIKEKLSPEEQVIF
NTDITDADRDEYMKMCVEGGRVYCFKEDPTKIPYNRIYHRFLYVLDWFVKIMFWLFVLS
YLASWFEPIKTLFSLGEPVVKHLPFLGKVVCKTEEL
>ARD71193.1 fatty acyl reductase [*Spodoptera exigua*]
MASETISSAELECLPDRIADTFSGMKVLITGGTGFMGKVLVEKLLRKCPDIEQIYLFVRAK
KGKNPKQRLEEIFSGPLFEKLRDMRGGVEPLLAKMTIVNGDVSEPDLGMSPEDRQMIINQ
VDIIIHAAATIRFDEELKKAVLLNVRGTKLMVELGKACKNLKVFIHISTAYCHLHEKLLEEK
PYPPPADPHQIIQAVEWMDEETIANMTPKLLNKLPNSYAFTKALAEALVVEAMEKANLPA
MVLRPSIVIPIWQEPVPGWTDNINGPTGLLIGAGKGVIRSMYCKSNSYADYLPVDVFINGI
MIVAWNYLKNGDKKCNIINFTSSAEIKVTWSEMIDAGREIIMNRVPLNGVVWYPGGSMKH
SRMYHNICVFFFHWIPAFIIDTLLFCLGYKPVLCRVQRRITKGFEVFEYYTNNQWDFKSDIA
QTLRQKLNAKERRDYKVDAVGLDISKYFEDCIRAARIFILKEYDDTLPAARRHMRIMYWV
DVIVNCLFWGFLLYWLSGWMTTSKAIVPDTATPTVIAMDA
>ARD71194.1 fatty acyl reductase [*Spodoptera exigua*]
MNTNFESTKKLLELSQRMKNIEVFLYISTAFTHTQKKVLVETVYPPPAKVEDIYKFIEENGD
DEQATLKFINGQPNTYTFTKSLSEAYLSKHHGNVPAVIIRPSIVSATNQEPIPGWLDNWYGS
TPFLMNASEGWLRIVRGNYNSGIDFIPVDFVTNLSIVAAAKAKRTNEVQVFHSTTSADNPT
DWSDFKNYFLDEVVRRGKNDLPYPNIVFVESKIALAIGSFLMQTVPAHMLDLWLKITGKE
PRYVKILAQVIRLRDSYEHFSSNDWIMRSDRTRQLHASLSPEDQEKFQCDPTQINWPEYLK
DYCRGVLKYLKPRKMY
>ARD71195.1 fatty acyl reductase [*Spodoptera exigua*]
MNYTAEKSPLSQTPVTREKMDKWVDAQVKGEKLDIDVYGKPTDKMLKELENVRNLSKE
LQDNLHELENSVRIAEVENQAMNPTAQILDFSEDHEFVPDNKDTYYAEEDKLDAKEEEKK
KLTKGKGPKTEIQEFYKDQCVFLTGGTGFLGKVLIEKLIRSCGDINTIYVLARNKKGKDPR
VRLHEMMDEFLFHRAHEENPKGIHKVVPILGDMELPGLGINEEDRKMLASKVTIIINAAAT
VKFDEKLSVSTAINVKGTKEVLKLAKECRNLKAITHVSTAFSNTQVKHIEEKFYEPPMSVE
ALEAISEVDEKLVESILPTLLGSRPNTYCFTKAVAEEAVRTYGEGLPICIVRPSIVVSTYEEPV
RGWTDSVYGPTGLVVGIGTGVLRTMYMDQEKVADMVPVDLCVNAILASAWHTAKNYK
ENQTSHIPIYNFVSGAQNPLTWGEFIERNRKYGIDKPTTKAVWYYGLNPTNNYYLFLFYNF
FLHYLPALMIDTYCAITGKRRAMLKLYSKVMKLANILFYFSTQDWKFSDMNVRNMWNS
LSDADRVIFPFSIGEMSWEYMCETFLVGLRVYLIKDDLSTLPEARKKWNKLFYLHQTLKAI
TLSLVVYLTYFVLQPIIALVFN
>ARD71196.1 fatty acyl reductase [*Spodoptera exigua*]
MASEVNEWYKGRSVLVTGALGLMGKVLIEKLLYSVPDVGCVYALVRSKRGKSPETRIEE

MWKLPLFKRIREEKPHVMKKLIPVTGDIMYDELGISAENLKNIYNEVSIVFHFAASLRLEA
PLKEGLEMNTRGTLRVLEMAKKMKSLVAFIHLSTAFCYPDYERMAEKVFDPPTDPHEVLR
AASWLTEEQLNLLAPSIYQKHPNSYTYSKRLAEALVRESYPNLPAVVVRPSIVTPSYKEPTP
GWVDNLNGPIGLMVGAGKGVIRSMHCYGHYHAEVIPVDIAINSIIVIAYKTGKDTERQPEI
PVYNLTTGDDRNTTWKEVLDIGKATVRKFPFEGPLWYPDGNIRHNKFIHDLCVFFYHIIPA
YFIDFLMFLFRQKRFMVRIQNRISIGLEVLQYFTTREWWFDTNNYKSLVHLLNPVDKETFP
MDTTIIEDEPYIESCMIGGKLYCLKEKLENLPKARLQNHILYILDRLVSLFFYLVLLYWIVSY
FEPARELLSYGGPAVRYLPLVGKAVFKDV
>ARD71197.1 fatty acyl reductase [*Spodoptera exigua*]
MADPSQVRSFYAGKNFFITGGTGFVGLCLIEKILRCIPDSGKIYLLMRPKKGKEIADRLQEF
PKNPVFEKLLESNSTDVFNKLIPIAGDVGEENLGMSPQDRQTIINNVNVVIHSAATLDFQE
NLRPTVNINLLGTRRVMQLCKDAKNLKVMIHVSSAYVNSYLTEAHEKIYEAPEDPEKVISL
VGTLNDEALLEIEPKLLKSHPNTYTFTKHLAEHEVVKCADLFPCTIVRPTMIVASWKEPIPG
WTCSKVGPQGFLMGAAKGVVRRLPLAKENVADYIPVDVVVNQLLVAGWEAANSSSGLT
VYHCSSSTCHPFTWTMLDDTVNGMLHKYPLKSAVWYPHLKFVPSLLMFRISAIFVHFFPA
LLLDLMLRMTGGRPILIRLHKNVWNSLNRLERFIFSEWKFHNPNTLELATKLNQTDKELFF
IDISKLYWVEYFKTLHLGVRRYLNKEKESTLPAARKKDMVLLLFHVMWQLFIMGLVWYI
FACATGLTLAHSAWVAPIIYILFTFL
>ARD71198.1 fatty acyl reductase [*Spodoptera exigua*]
MAPNVKEYYRGKNIFITGGTGFVGKALLEKLLRNCSEVNAIYLLMRQKKGVSAEERLKD
LCSKPVFDMVREKNPSSFKKLMIINGDITEAGLGISEDDVKLLQKECNIIFHSAACVRFDQ
KLKDAVNMNTTGTLRMLTLAESMQNLEVFVHLSTAYCRCDLDVLEEKVYTAVHKPRKIM
DIVEWMDDETLAYLEPKIISSEPNTYSYTKAITEDLVNEYSGKFPIAIARPSIVTAVWKEPIP
GWVDNLNGPTGIVIGSGKGVIRTMHCEPSYKADAISVDVVANACILIAYVTGLDKPKETQ
VYNLTLSGVINLTWQEIIDLGEKWVNEFPYTMALWYPGGSIKSYRITHQIDKFFSHLVPAYL
VDALLFLLGKKTFMINLQKRISHGLNVLQYYTTKEWHFRNNNYKGLRNRVTPEDNEVFY
TDASTLDPDEYLKNYVLGTRKFCCNEDPANLPRARKLHRIRYMADRFFKLLFIFLILWTLY
SNSHVFTSSVELLDNSLKSLPLMNQANAEEISNIAL
>AKD01773.1 fatty acyl-CoA reductase 12, partial [*Helicoverpa armigera*]
MVVLTSKETKPSVAEFYAGKSVFITGGTGFLGKVFIEKLLYSCPDIGNIYMLIREKKGLSVS
ERIKHFLDDPLFTRLKEKRPADLEKIVLIPGDITAPDLGITSENEKILIEKVSVIIHSAATVKFN
EPLPTAWKINVEGTRMMLALSRRMKRIEVFIHISTAYTNTNREVVDEILYPAPADIDQVHQF
VKDGISEEETEKILNGRPNTYTFTKALTEHLVAENQAYVPTIIVRPSVVAAIKDEPIKGWLG
NWYGATGLTVFTAKGLNRVIYGHSSYIVDLIPVDYVANLVIAAGAKSSKSTELKVYNCCSS
ACNPITIGKLMSMFAEDAIKQKSYAMPLPGWYIFTKYKWLVLLLTLLFQVIPAYITDLYRHL
IGKNPRYIKLQSLVNQTRSSIDFFTSHSWVMKADRVRELFASLSPADKYLFPCDPTDINWT
HYIQDYCWGVRHFLEKKTTNK
>AKD01763.1 fatty acyl-CoA reductase 2, partial [*Helicoverpa armigera*]
MVPRPAPQSSTPPLIPEFFAGREVFITGATGFMGKVLVERLLWTCRDISRLHLLLREKKDVA
PEKRLSQLKQSQVFDVIRQHCPKQLDKLSMLAGDVTKHRFGLDHHAISQLNQVSVVFHS
AATLKFDEPLPVALQQNVHSVVTLMDICDQLPNMQVLVHVSTAYSNAELTSVEERVYPAP
AQLQQLSALVEALPAGLLAEITPQLISPKPNTYTFTKAMAESVVAERANTANYAVAIFRPTI
VISSLRHPFPGWIENLNGPSGVVVGAGKGLLHVLSCGAVRRADMMPVDIAIDTLIAVAWE
AANDQPGYARVYNCSSCMDGTSWGQFRARMMRCVREHPFDSVLWYPFGVLSENTLMQ

RFLETTLQTVPLYFVHYISKLCGIKSRPSMTTVSKRLHAMNEALKFFALREWHFNTDNVQ
QLMHRLAPADAAVYNLDPGTIDWESHCEDFVKGTRKYLLREKDQDIEAARRRMHVLHM
IHSLTKILLTLLMARLAYRSTPAILRAVAVLTRLRRRGATVALSA
>AKD01774.1 fatty acyl-CoA reductase 13, partial [*Helicoverpa armigera*]
MAPSVSVAEYYAGKTLFITGATGFMGKVMVEKMLRSCPDVKKMYLLMRPKKGHSSKER
LDDLLSFKIFDRLKAENPKIFDKLHVIPGDILSEDLGISDEDRRLIQSEAQVIFHCAACVRFD
MFLRDAVKMNTVGTKKVLQLAEGVKNLEAFIHVSTSYCRCELPELEEKLYPSKHRPEHV
MHCVSWMDDDLLTHLQPKIIEPQPNTYAYTKSLTEDLVSQYEGKFPIAIARPSIVAAAYKEP
LPGWVDNLNGPTGLLVGAGKGVIRTMHCNDSYTADVVPVDVTVNACIILGYLTGLEQPK
QINVVNVTQSEINPITWGQALDMGRVHVQEFPFTVCLWYPGGSPKSSRVAHQLALFFTHL
LPAYLVDMLMFLMGKKTFMIKIQKRINYGLEVLQYYTTKEWFFRNDNFVALQHRISKSDN
ETFYTDMKDMDWSGYIRNYIRGAREYCCKEDPATLPAARRLQTQLYYLDKAVQIMVGLL
VSYFIYYYFNMLYSVISS
>AKD01770.1 fatty acyl-CoA reductase 9, partial [*Helicoverpa armigera*]
MSSPSIRDFYKGRNILVTGGTGFMGKVLIEKLLYSVPEVGNIYILMRPKRGKSVSQRTEDL
QRLKLFERLRTENPNALKKLKPLQGDVLFDNLGLSDADIELLTKEVSVVFHFAATLRLEAP
LKDNVNMNTCGTQRAIDLAKRMKKLQIFVHLSTAFCYPDYEVLGEKCHAPPVKPDNVM
KLIEWLDDKQVDLLTPSLLGPHPNCYTFSKRLAENIVEQAYQDLPIVIARPSIVCPSVKEPM
PGWVDNLNGPVGVMLGAGKGVIRTMLCDGSLVAQVIPVDIAINAIIAIGMIEGSRTEKPES
LPVYNVNNGHQKPTTWGDVLSVAKAYGRKYPLSWPLWYPNGDITTNPILHEYKRIFYHL
VPAYLIDFLLFLLGQKRLMIRIQERISRGLEVLQYFTMRPWNFPCPNYDAVREKLNPEEQAI
FNTDIKDVDRDEYMKICIEGGRVFCFKEDPTKVPMNRAYHNFLYVLDWFVKIMFWLFVFS
VLASWFSPVKSLFSLGEPVVKHLPFLGKVIEKQD
>AKD01769.1 fatty acyl-CoA reductase 8, partial [*Helicoverpa armigera*]
MEADGMAQLKEVSIVFHSAATLKFDEPLRVAMEQNVRSVERLLEICDKLPNIQAFIHVSTA
YSNAELTRVEERVYAPPVPLAQALTVADSVPEHLLATINAQYIAPKPNTYTFTKALAETVV
EEHGNRGYPVAIFRPSIVISSLRHPFPGWIENLNGPSGVVVGAGKGLLHVFCCRSRAGADM
LPVDIAIDTLLAVAWETAVDRPEHVRVYNCSTCENPTTWGDFEDALRKNLRGHPLDNTFW
YPSGYSVENKVTQKAMETLLQTLPLHIAEYVTKLLRIKTRMSLITVSQRLKAMNEVLRFF
SVREWHFETNNVKRLQARLTPQDAAIYNLDPQTINWDDHYENFVKGTRKYLLKEKDQDI
QEARKHLRKMYYVHYGFLFFVVTLICRLMLQNHYIRTLVFRTFKLLLTVIGSVFLRIQS
>AKD01778.1 fatty acyl-CoA reductase 18, partial [*Helicoverpa armigera*]
MLSVISTAKEPLKGWLDNMYGPTGVAVGSATGILRTLQCDEMVSADIVPVDSVVNCLMV
AACSVHHSYKQSSPPLEPPIFNYVSSVENRITWGEFMLQNMAWIHYYPFSEAVWFISLRLT
KSALMNKIYVLFLHLIPAALVDGLAVCLGRKPKMLKVYRKIHKFSSVLSYFCTREIKFCNT
RTRELWE
>AKD01777.1 fatty acyl-CoA reductase 17, partial [*Helicoverpa armigera*]
TAKNFKENQTSHIPIYNFVSGAQKPITWGDFIERNRKYGIDKPTTKAVWYYGLNPTNNYY
LFLFYNFFLHYLPALMIDTYCAITGKRRAMIKLYNKVMKLANILFYFSTQDWQFSDYNVR
NMWKSLSDEDRVVFPFSIGEMSWEYMCETFLVGLRVYLIKDDLSSLPEARKKWNKLYYL
HQILKAVTLGLVINLAYFVLKPVLALVFGH
>AKD01776.1 fatty acyl-CoA reductase 15, partial [*Helicoverpa armigera*]
MGKVLIEKLLYSVPDLGCVYALVRSKRGKSPETRIEEMWKLPLFQRIREEKPHVMKKLIPV
TGDIMYDELGISADRLNDIYNEVSIVFHFAASLRLEAPLKEGLEMNTKGTLRVLTMAKKM

KKLVAFLHLSTAFCYPDYERMAEKVFDPPADPHEVLRAASWLTEEQLNLLAPSIYQKHPN
SYTYSKRLAEALVRESYPELPAVVVRPSIVTPSYKEPTPGWVDNLNGPIGLMVGAGKGVIR
SMHCYGHYHAEVIPVDIAINSIVVIAYKTGKDTQRQPEIPVYNLTTGDDRNTTWKQVLDIG
KATVRKFPF
>AKD01772.1 fatty acyl-CoA reductase 11, partial [*Helicoverpa armigera*]
MASETMSAADLESLPDRIADTFSGMRVLVTGGTGFMGKVLLEKLLRKCPDIGQIMLFVRN
KKGKNPKQRLEEIFNGVLFEKVRNMRGGVEPLLQKVTLVAGDVSEPDLGLSDQDRAMIV
DQVDIIIHAAATIRFDEELKKAVLLNVRGTKLMVELAKTCKNLKLFIHISTSYCHLHEKLLE
EQAYPPPADPHHVIQAVEWMDDEAIAMLTPKLLDKLPNSYAFTKALGEALVVEAMEHIPA
MVLRPSIVIPIWQEPVPGWTDNINGPTGLLIGAGKGVIRSMYCKSNSYADYLPVDVFINGI
MIVAWNYLQNGDTKCNIINFTSSAEIKVTWSEMIDAGREIIMNRVPLNGVVWYPGGSMKH
SRLYHNICLVLFHWVPAVLVDILLFCLGYKPILCRVQRRINKGFEVFEYYTNNQWDFKSDI
AQTLRQKLNPRERRDYKVDAVGLDISKYFEDCIRAARVFILKEYD
>AKD01768.1 fatty acyl-CoA reductase 7, partial [*Helicoverpa armigera*]
MAAPQLISVSDVISDNLKNEKEINYCSKCIDNVDENMNNTNENLTEVQKFYNGKNILITGA
TGFLGKILVEKLLRCCPGVENLYLLVRQKRGKDIYTRMEEIFDDPVFSRLKDEVPKFRHKV
VVVPADCEAAGLGLTLTDRQMLTEKVNIIFHSAATVKFDEHLRAALLTNVKAPLHLLRLA
RDMKKLDVLMHISTAYSNSHLPEIEERYYPCEADCEQLHQMIDKMSDNEINKILPKILGPW
PNTYTFTKALAEKELRENAGGMPIGIFRPA
>AKD01766.1 fatty acyl-CoA reductase 5, partial [*Helicoverpa armigera*]
MLVDMITQNQLFEAKQGGDITFMDMVDEREALGESQIQKMFAGSSVLLTGGTGFLGKLV
LEKLLRSCPDLKKIYLLARPKKNKDITKRLQEQFDDVLYDKLRKECPNFIQKIRIVEGDMG
QLELGMCPEDRIKIMNEVDVIFHGAATVRFDEPLKTAVEINVRGTREMFKLARGCTKLKA
FVHISTAYSNCPQTNIDEKFYESPLPGDKLIDMVETIDARTLDSITPGLLGDFPNTYAYTKAV
AENIVLEYSQGLPVALFRPSIVIGTAKEPVSGWIDNVYGPTGVVVGAAVGLLHVLNCDAK
VVADLVPGDMVVCACIAAAWRTARDSRSNHEDAPPPDLPPPVYNYVSSEQKPLTWEKFM
HYNEVYGFQVPTVQAIYYYLFTITSSRFLYTLYCFLLHWVPAYIIDGIAVIIGKKPMLRKAY
TKITKFSEVMAYFATREWKFDNSNTQKLYSEMCEADKHLFDFDMSTMDWNDYFYNYIRG
VRVYLLKDPVDTVPAGLKKLNRLRLLHYTFCAILGLLFLRLIWAIFSGILGFSF
>AKD01765.1 fatty acyl-CoA reductase 4, partial [*Helicoverpa armigera*]
MNHTIEESPLMQTPMTQEKMDKWVDAQIKGEKIEIDVYGKPTEQMLKELENVRNLSKEL
QDNLHELETSVRIAEVENQAMNPTAEILDFSEDHEFVPDNQDTYYAEEDKMDAKEEEKQ
KLTKGKGAKTEIQSFYKDQCVFLTGGTGFLGKVLIEKLIRACGDINTIYVLARSKKGKDAT
VRLHEMMDEFLFHRAHEVNPKGIHKVVPIVGDMELPGLGISEEDRKMLTSKVTIIINAAAT
VKFDEKLSVSTAINVKGTKEVLKLAKECRNLKAITHVSTAFSNTHVNHIEEKFYEPPMSVE
ALEALTEVDNKLIENILPTLLGKRPNTYCFTKAIAEEAVRKYGEGLPISIVRPSIVVSTYEEP
VRGWTDSVYGPTGLVVGIGTGVLRTMYMDQSKVADMVPVDLCVNAILASAWFTAKNFK
E
>AKD01764.1 fatty acyl-CoA reductase 3, partial [*Helicoverpa armigera*]
MSVIAAARAKKSSDVQVYNCTSSAENPIIWSNVHKYFNREMVARGKNEIPYPHVIYLKSK
PLMNIGTFILQTTPAQIADMWLKITGREPKYTETLSKVLKVRDGYEFFTANSWVMKAERA
RELYSSLSPEDRAEFPCDVTQIVWSEYMRDYCRGILKYITPRTNGK
>ATJ44516.1 fatty acyl reductase 1 [*Helicoverpa assulta*]
MVVLTSKETKPSVAEFYAGKSVFITGGTGFLGKIFIEKLLYSCPDIGNIYMLIREKKGLSVSE

RIKQFLDDPLFTRLKDKRPADLEKIVLIPGDITAPDLGITSENEKMLIEKVSVIIHSAATVKFN
EPLPTAWKINVEGTRMMLALSRRMKRIEVFIHISTAYTNTNREVVDEILYPAPADINQVHQY
VKDGISEEETEKILNGRPNTYTFTKALTEHLVAENQAYVPTIIVRPSVVAAIKDEPIKGWLG
NWYGATGLTVFTAKGLNRVIYGHSSYIVDLIPVDYVANLVIAAGAKSSKSTELKVYNCCSS
ACNPITIGKLMSMFAEDAIKQKSYAMPLPGWYVFTKYKWLVLLLTILFQVIPAYVTDLYRH
LIGKNPRYIKLQSLVNQTRSSIDFFTSHSWVMKADRVRELFASLSPADKYLFPCDPTDINWT
HYIQDYCWGVRHFLEKKTTNK

>ATJ44527.1 fatty acyl reductase 16 [*Helicoverpa assulta*]

MDTKIMTTDKMDDYQKHIEMLNGNDLNTLNLNYEGDHINLEQPDKSSEAIADFYEDSVI
MVTGGTGFVGKALLEKLLRSCPGIKTIYVLMRPKRGLTVDQRYKELLKNQVFDRIRARWP
ERLGKLYPITGDVSAPNLGVSPEQRELLAKVTTLFHSAATVRFTEPLHAATTLNVQGTASL
LKLAEDMHKLKALVHVSTAYSNAPRPSIEERVYPPPYDPDSIVRCTRMLPAETVEVIAETL
QGEHPNPYTLTKALAESIVYSHTNLPVCIVRPSIVTAALQEPYPGWIDNVYGVTGLIMEISR
GTYRSGYSREQYVVDLVPVDLVVNSCIVAAWRQGVKRPGHCPVYNVTSGSINPIQWGQF
TKLCVKWAIENPTKYVMWYPNFSFTESRFMNTFWEVSCHFLPAFLYDMLLRAQGRKAIM
MKLARRFKMAAATGEYFANHEWEFGISELKALHDDVTATRDGAVFPHWSDFEWDSYIGA
YMFGIRRYILKDTAESLPVARTKLRRLYWVHKLFQAATGYYLFRFLAGRLR

>ATJ44526.1 fatty acyl reductase 12 [*Helicoverpa assulta*]

MGFLEDRDLSSVPSIAEFYKGKTIFISGGSGFMGKVLVEKLLYSCPDLDRIYLLLRNKKGV
KSEDRLAQLFSSLCFDRLRRERPSFASKVFVIAGDVLEVGLGLSEEDRTLLVNRVNIVFHVA
ASVRFDDPLEYAVRMNLRGTKEMVELAADMRNLCSFIHVSTSYSNTNRDPIEEILYPPHAD
WRDTLEVCEKTDPHILKVLTPKYLGELPNTYTFSKQLAENVVAEYAGRLPVVIIRPSIVISS
VEEPIPGWIENFNGPAGLLVACGKGIMRSLYTDPDLIADYMPVDISIKSFIVASWLRGTKELS
PSDDVPIYNCCAGKLNNITMGEMVTIGKQIYPSVPLNDMLWHVGGDLTTSKTVHYIKVIL
LHLLPAILVDTILWVMGRKPLLVKIQRRIYIANLALMYYITKQWTFDNKNLVLLRSKIKEV
DRKSFYYEIENVDKYEYFVNSVKGGKKYLLKEKDEDLPKAKIHYQRMLILDTIVQILFHG
YVFWWFLNLSFVQNFISYLFSNV

>ATJ44525.1 fatty acyl reductase 10 [*Helicoverpa assulta*]

MAPSVSVAEYYAGKTLFITGATGFMGKVMVEKMLRSCPDVKKMYLLMRPKKGHSSKER
LDDLLSFKIYDRLKAENPKIFDKLHVIPGDILSEELGISDEDRRLIQSEAQVIFHCAACVRFD
MFLRDAVKMNTVGTKKVLQLAEGVKNLEAFIHVSTSYCRCELPELEEKLYPSKHRPEHV
MHCVSWMDDDLLTHLQPKIIEPQPNTYAYTKSLTEDLVSQYEGKFPIAIARPSIVAAAYKEP
LPGWVDNLNGPTGLLVGAGKGVIRTMHCNDSYTADVVPVDVTVNACIILGYLTGLEQPK
QINVVNVTQSEINPITWGQALDMGRVHVQEFPFTVCLWYPGGSPKSSRVAHQLALFFTHL
LPAYLVDMLLFLMGKKTFMIKIQKRINYGLEVLQYYTTKEWFFRNDNFVALQHRISKSDN
ETFYTDMKDMDWSGYIRNYIRGAREYCCKEDPATLPAARRLQTQLYYLDKAVQIMVGLL
VSYFVYYYFNMLYSVISS

>ATJ44524.1 fatty acyl reductase 11 [*Helicoverpa assulta*]

MVPRPAPQSSTPPLIPEFFAGREVFITGATGFMGKVLVERLLWTCRDISRLHLLLREKKDVA
PEKRLSQLKQSQVFDVIREHCPEQLDKLSMLAGDVTKRRFGLDHHAISQLNKVSVVFHSA
ATLKFDEPLPVALQQNVHSVVTLMDICDQLPNMQVLVHVSTAYSNAELTSVEERVYPAPA
QLQHLSALVEALPAGLLAEITPQLISPKPNTYTFTKAMAESVVAERANTANYAVAIFRPTIVI
SSLRHPFPGWIENLNGPSGVVVGAGKGLLHVLSCGSVRRADMMPVDIAIDTLIAVAWEAA
NDQPGYARVYNCSSCMDGTSWGQFRARMMRCVREYPFDSVLWYPFGVLSENTLMQRFL

ETTLQTVPLYFVHYISKLCGIKSRPSMTTVCKRLHAMNEALKFFALREWHFNTDNVQQLM
HRLAPADAAVYNLDPGTIDWESHCEDFVKGTRKYLLREKDQDIEAARRRMHVLHMIHSL
TKILLTLLMVRLAYRSTPAILRAVAVLTRLRRRGATVALSG
>ATJ44523.1 fatty acyl reductase 9 [*Helicoverpa assulta*]
MEADGMAQLKEVSIVFHSAATLKFDEPLRVAMEQNVRSVERLLEICDKLPNIQAFIHVSTA
YSNAELTRVEERVYAPPVPLAQALTVADSVPEHLLATINAQYIAPKPNTYTFTKALAETVV
EEHGNRGYPVAIFRPSIVISSLRHPFPGWIENLNGPSGVVVGAGKGLLHVFCCRSRAGADM
LPVDIAIDTLLAVAWETAVDRPEHVRVYNCSTCENPTTWGDFEDALRKNLRGHPLDNTFW
YPSGYSVENKVTQKAMETLLQTLPLHIAEYVTKLLRIKTRMSLITVSQRLKAMNEVLRFF
SVREWHFETNNVKRLQARLTPQDAAIYNLDPQTINWDDHYENFVKGTRKYLLKEKDQDI
QEARKHLRKMYYVHYSFLFFLVTLICRLVLQNQYIRTLVFRTFKLLLTVIGSVFMRIQS
>ATJ44522.1 fatty acyl reductase 6 [*Helicoverpa assulta*]
MLVDMITQNQLFEAKQGGDITFMDMVDEREALGESQIQKMFAGSSVLLTGGTGFLGKLV
LEKLLRSCPDLKKIYLLARPKKNKDITKRLQEQFDDVLYDKLRKECPNFIQKIRIVEGDMG
QLDLGMCPEDRIKIMNEVDVIFHGAATVRFDEPLKTAVEINVRGTREMFKLARGCTKLKA
FVHISTAYSNCPQTNIDEKFYESPLPGDKLIDMVETIDARTLDSITPGLLGDFPNTYAYTKAV
AENIVLEYSQGLPVALFRPSIVIGTAKEPVSGWIDNVYGPTGVVVGAAVGLLHVLNCDAK
VVADLVPGDMVVCACIAAAWRTARDSRSNHEDAPPPDLPPPVYNYVSSEQKPLTWEKFM
HYNEVYGFQVPTVQAIYYYLFTITSSRFLYTLYCFLLHWVPAYIIDGIAVIIGKKPMLRKAY
TKITKFSEVMAYFATREWKFDNSNTQKLYSEMCEADKHLFDFDMSTMDWNDYFYNYIRG
VRVYLLKDPVDTVPAGLKKLNRLRLLHYTFCAILGLLFLRLLWAIFSGILGFSF
>ATJ44521.1 fatty acyl reductase 5 [*Helicoverpa assulta*]
MAAPQLISVSDVITDNLKNEKEINYCSQCIDNVDENMNNTNENLTEVQKFYNGKNILITG
ATGFLGKILVEKLLRCCPGVENLYLLVRQKRGKDIYTRMEEIFDDPVFSRLKDEVPKFRHK
VVVVPADCEAAGLGLTLTDRQMLTEKVNIIFHSAATVKFDEHLRAALLTNVKAPLHLLRL
ARDMKKLDVLMHISTAYSNSHLSEIEERYYPCEADCEQLHQMIDKMSDNEINKILPKILGP
WPNTYTFTKALAEKELRLNAGGMPIGIFRPAIVISTAKEPLKGWLDNMYGPTGVAVGSAT
GILRTLQCDEMVSADIVPVDSVVNCLMVAACSVHHSYKQSSPPLEPPIFNYVSSVENRITW
GEFMLQNMAWIHYYPFSEAVWFISLRLTKSALMNKIYVLFLHLIPAALVDGLAVCLGRKP
KMLKVYRKIHKFSSVLSYFCTREIKFCNSRTRELWEKTSEADKQIYPFSMSEMNWEEYFQ
HYLGGIRRFLFKENDDTLPQARIKWKRLYYLHQIARFIFFILAVYCLWWILSLIW
>ATJ44520.1 fatty acyl reductase 2 [*Helicoverpa assulta*]
MASETMSTADLETLPDRIADTFSGMRVLVTGGTGFMGKVLLEKLLRKCPDIGQIMLFVRN
KKGKNPKQRLEEIFNGVLFEKVRNMRGGVEPLLQKVTLVAGDVSEPDLGLSDQDRAMIID
QVDIIIHAAATIRFDEELKKAVLLNVRGTKLMVELAKSCKNLKLFIHISTSYCHLHEKLLEE
QAYPPPADPHHVIQAVEWMDDEAIAMLTPKLLDKLPNSYAFTKALGEALVVEAMEHIPAM
VLRPSIVIPIWQEPVPGWTDNINGPTGLLIGAGKGVIRSMYCKSNSYADYLPVDVFINGIMI
VAWNYLQNGDTKCNIINFTSSAEIKVTWSEMIDAGREIIMNRVPLNGVVWYPGGSMKHSR
LYHNICLVLFHWVPAVLVDILLFCLGYKPILCRVQRRINKGFEVFEYYTNNQWDFKSDIAQ
TLRQKLNPRERRDYKVDAVGLDISKYFEDCIRAARVFILKEYDDTLPAARRHMRVMYWV
DVIVRCLFWGLILYWLSGFVTSPSTITEHNATVIAMDA
>ATJ44519.1 fatty acyl reductase 7 [*Helicoverpa assulta*]
MTSEVNEWYKGRSVLVTGALGLMGKVLIEKLLYSVPDLGCVYALVRSKRGKSPETRIEEM
WKLPLFQRIREEKPHVMKKLIPVTGDIMYDELGISADRLNDIYNEVSIVFHFAASLRLEAPL

KEGLEMNTKGTLRVLTMAKKMKKLVAFLHLSTAFCYPDYERMAEKVFDPPADPHEVLRA
ASWLTEEQLNILAPSIYQKHPNSYTYSKRLAEALVRESYPELPAVVVRPSIVTPSYKEPTPG
WVDNLNGPIGLMVGAGKGVIRSMHCYGHYHAEVIPVDIAINSIVVIAYKTGKDTQRQPEI
PVYNLTTGDDRNTTWKQVLDIGKATVRKFPFEGPLWYPDGNIRHNKFIHDLCVFFYHIVP
AYFIDFLLFIFRQRRFMVRIQNRITIGLEVLQYFTTREWWFDTNNFKSLVGLLNPVDKETYP
MDLTIIEDEPYIESCMIGGKLYCLKEKLENLPKARLQNHILYILDRLVSLVFYLVLLYWIVSY
FEPARELLSYGGPAVRYLPLVGKAVFRDA
>ATJ44518.1 fatty acyl reductase 4 [*Helicoverpa assulta*]
MADESQVRAFYAGKNFFITGGTGFVGLCLIEKILRCMPDVGKIYLLMRPKKGKEISERLEE
FPKNPVFEKLLESHSTDIFKKLIPVSGDVGEANLGLSPADRQMLIDNINVVIHSAATLDFQE
SLRPTVNINLLGTRRIMELCKDAKDLKVMIHVSSAYVNSFLKEAHEKVYEAPEDAQKVIS
LVETLNDESLLQIEHKLLKSHPNTYTFTKHLAEHEVIKCIDMFPCTIVRPTMIVASWKEPVP
GWTCSKVGPQGFLMGAAKGVVRRLPLAKEKVADYIPVDVVINQLLVAGWEAAKSKSGL
TVYHCSSSTCNPFTWTMLDNTVNNMLHKYPLKSAVWYPHLKFVPTLLMFRISAIFVHFFP
AFLLDLMLRVTGGRPILIRLHKNVWNSLNRLETFIFSEWKFYNPNTLELATKLSKKDKELF
YIDITSLQWVEYFSTLHLGVRRYLNREKESSLPAARNKDMVLLVFHVIWQLFIMGLLWYIF
AWQTGLTLATSAWIAPIIYVLYNLL
>ATJ44517.1 fatty acyl reductase 3 [*Helicoverpa assulta*]
MNHTIEESPLMQTPMTQEKMDKWVDAQIKGEKIEIDVYGKPTEQMLKELENVRNLSKEL
QDNLHELETSVRIAEVENQAMNPTAEILDFSEDHEFVPDNQDTYYAEEDKMDAKEEEKQ
KLTKGKGAKTEIQSFYKDQCVFLTGGTGFLGKVLIEKLIRACGDINTIYVLARSKKGKDAT
VRLHEMMDEFLFHRAHEVNPKGIHKVVPIVGDMELPGLGISEEDRKMLTSKVTIIINAAAT
VKFDEKLSVSTAINVKGTKEVLKLAKECRNLKAITHVSTAFSNTHVNHIEEKFYDPPMSVE
ALEALTEVDNKLIENILPTLLGKRPNTYCFTKAIAEEAVRKYGEGLPISIVRPSIVVSTYEEP
VRGWTDSVYGPTGLVVGIGTGVLRTMYMDQSKVADMVPVDLCVNAILASAWFTAKNFK
ENQTSHIPIYNFVSGAQKPITWGDFIERNRKYGIDKPTTKAVWYYGLNPTNNYYLFLFYNF
FLHYLPALMIDTYCAITGKRRAMIKLYNKVMKLANILFYFSTQDWQFSDYNVRNMWKSL
SDEDRVVFPFSIGEMSWEYMCETFLVGLRVYLIKDDLSSLPEARKKWNKLYYLHQILKAV
TLGLVINLAYFVLKPVLALVFGH
>ATJ44471.1 fatty acyl reductase 1 [*Helicoverpa armigera*]
MVVLTSKETKPSVAEFYAGKSVFITGGTGFLGKVFIEKLLYSCPDIGNIYMLIREKKGLSVS
ERIKQFLDDPLFTRLKEKRPADLEKIVLIPGDITAPDLGITSENEKILIEKVSVIIHSAATVKFN
EPLPTAWKINVEGTRMMLALSRRMKRIEVFIHISTAYTNTNREVVDEILYPAPADIDQVHQY
VKDGISEEETEKILNGRPNTYTFTKALTEHLVAENQAYVPTIIVRPSVVAAIKDEPIKGWLG
NWYGATGLTVFTAKGLNRVIYGHSSNIVDLIPVDYVANLVIAAGAKSSKSTDLKVYNCCSS
ACNPITIGKLMSMFAEDAIKQKSYAMPLPGWYIFTKYKWLVLLLTILFQVIPAYITDLYRHLI
GKNPRYIKLQSLVNQTRSSIDFFTSHSWVMKADRVRELFASLSPADKYLFPCDPTDINWTH
YIQDYCWGVRHFLEKKTTNK
>ATJ44472.1 fatty acyl reductase 8 [*Helicoverpa armigera*]
MSSPSIRDFYKGRNILVTGGTGFMGKVLIEKLLYSVPEVGNIYILMRPKRGKSVSQRTEDL
QRLKLFERLRTENPNALKKLKPLQGDVLFDNLGLSDADIELLTKEVSVVFHFAATLRLEAP
LKDNVNMNTCGTQRAIDLAKRMKKLQIFVHLSTAFCYPDYEVLGEKCHAPPVKPDNVM
KLIEWLDDKQVDLLTPSLLGPHPNCYTFSKRLAENIVEQAYQDLPIVIARPSIVCPSVKEPM
PGWVDNLNGPVGVMLGAGKGVIRTMLCDGSLVAQVIPVDIAINAIIAIGMIEGSRTEKPES

LPVYNVNNGHQKPTTWGDVLSVAKAYGRKYPLSWPLWYPNGDITTNPILHEYKRICYHL
VPAYLIDFLLFLLGQKRLMIRIQERISRGLEVLQYFTMRPWNFPCPNYDAVREKLNPEEQAI
FNTDIKDVDRDEYMKICIEGGRVFCFKEDPTKVPMNRAYHNFLYVLDWFVKIMFWLFVFS
VLASWFSPVKSLFSLGEPVVKHLPFLGKVIEKQD
>ATJ44470.1 fatty acyl reductase 15 [*Helicoverpa armigera*]
MGFLEERDLSGVPTIPDFYKGKTIFVTGGSGFIGKVLIEKLLYSCTDLDRIYLLLRNKKGVK
SEDRLAELYAAPCFQRLKAERPGVFESKVFVVSGNVMEAGLGLSQEDRALLVNRVNVIFH
VAASVRFDDTLKYSTQLNLRGTVEVMELAKEMRELCSLVHVSTSYANTNRDTIEEVLYPP
LADWRETLAICEKADEHTLKILTPKFLGELPNTYTFTKQLAEHVVNEYKGQLPIIIVRPSIVI
SSIDEPIPGWIESFNGPVGIFVACGKGIMRTIHTKADIKSDFMPVDVCVKNIIAGAWIRGTKI
MDPTDDIEIYNCCSGNLHPILMGDLIAMSKQVAKDVPLDNMVWYMGGTITASEKYHYVK
VLLQHLLPAMLIDTLLWLFGKKPMLVKIQRRIYIANLALRYYTTQQWTFTNTNFTKLRSVI
KLEDIEQFYYELESTNVVEYFKQCCLGGRRFLLKEKDEDIPKARLHCQRMMVVDRIFQIA
FYTVLIWWVCSKIVKVFNLHSNIF
>ATJ44469.1 fatty acyl reductase 12 [*Helicoverpa armigera*]
MGFLEDRDLSSVPSIAEFYKGKTIFISGGSGFMGKVLVEKLLYSCPDLDRIYLLLRNKKGV
KSEDRLAQLLSSLCFDRLRRERPSFASKVFVIAGDVLEVGLGLSEEDRTLLVNRVNIVFHVA
ASVRFDDPLEYAVRMNLRGTKEMVELAADMRNLCSFIHVSTSYSNTNRDPIEEILYPPHAD
WRDTLEVCEKTDPHILKVLTPKYLGELPNTYTFSKQLAENVVAEYAGKLPVVIIRPSIVISS
VEEPVPGWIENFNGPAGLLVACGKGIMRSLYTDPDLIADYMPVDISIKSFIVASWLRGTKEL
SPSDDVPIYNCCAGKLNNITMGEMVTIGKQIYPSIPLNDMLWHVGGDLTTSKTVHYIKVIL
LHLLPAILVDTLLWAMGRKPLLVKIQRRIYIANLALMYYITKQWTFDNKNLVLLRSKIKEV
DRKSFYYEIENVDKYEYFVNSVKGGKKYLLKEKDEDLPRAKIHYQRMLILDTMVQILFH
GYVFWWFLHLSFVQNFISYLFSNV
>ATJ44468.1 fatty acyl reductase 13 [*Helicoverpa armigera*]
METEAMDPAQEFVAKVHARQKPVLEAIARWDSPVQQFYENTTVFITGGSGFLGKQLIEKL
FRATKISKILLLLRSKKGKPIEQRLLDMLQDPVFDAVKELHPNFAEKIIPVAGDVAEMKLGL
SEKDWNLVADETDIFMHVAATTRFDEPLKIATLINVRGAREALLLGKACKKLKSYVHVST
AYSHACENMINTEVLEDFYKSPIDPETLIQLAETLDEEKLNEISSGLIKNWPNTYSFGKAVA
EETVRSMAEGLPLCIVRPAIVIVAHKEPTPGWLDMSNVYGASGVVLGPGIGLMHTIMADN
DVNIGLVPVDYVNNAIIVSAYETYKKVQKGETKPKIYTVTTSTRNPTRWGWLVDFTEGYI
AKQYPSPSAIAYAFAWGTNNPTVFWLYSWLLHFIPAYVIDAVCFVLGKERRFVKIYTKMFK
MSMALSYFTVNDWRFIDDNTAALYDGLSTIDKTIFNFDVTQLQWTEYMVLWCLGLRKFI
VKDGLKGSVYAVKKQFFFKILFCVLFPAYLFLLYKVFVFAVSSLYLVLRLFF
>ATJ44467.1 fatty acyl reductase 14 [*Helicoverpa armigera*]
MASCLSGGHYVPGSQEYVPVAEFYADKSVFVTGGTGFMGKVLVEKLLRSCPKIKKIYLLM
RPKRGQDVASRLTELTQSPLFETLRRERPQELNKIVPIVGDITEPELGISAADQTMLCQKVS
VVFHSAATVKFDEKLKLSVTINMLGTQQLVQLCHRMLSLEALVHVSTAYCNCERERVEET
VYAPPAHPEHVVTLVQTLPDELVDRITPDLVGDRPNTYTFTKALAEDMLIKESGNLPVSIV
RPSIVLSSLREPVKGWVDNWNGPNGIIAAVGKGIFRTMLGTGTKVADLVPVDTVINLMIVC
AWRTHLRRGEGVVVYNCCTGQQNPITWQRFVKTSFKYMRKHPFNEVLWYPGGDITSNR
LKHGTLSLLQHRAPAALMDLVSTATGKKPMMVRVQNKLEKAAACLEYFTTRQWAFADD
NVQALCASLSPDDRRTFDFNVRNIDWDAYIESYVLGIRRFLFKESPDTLPKSRAVLRRLHIV
HILTQVATVFFLWRFLFSRSNALRNVWRRVLELLTRAARLLAIA

>ATJ44466.1 fatty acyl reductase 11, partial [*Helicoverpa armigera*]
MVPRPAPQSSTPPLIPEFFAGREVFITGATGFMGKVLVERLLWTCRDISRLHLLLREKKDVA
PEKRLSQLKQSQVFDVIRQHCPKQLDKLSMLAGDVTKRRFGLDHHAISQLNQVSVVFHS
AATLKFDEPLPVALQQNVHSVVTLMDICDQLPNMQVLVHVSTAYSNAELTSVEERVYPAP
AQLQQLSALVEALPAGLLAEITPQLISPKPNTYTFTKAMAESVVAERANTANYAVAIFRPTI
VISSLRHPFPGWIENLNGPSGVVVGAGKGLLHVLSCGAVRRADMMPVDIAIDTLIAVAWE
AANDQPGYARVYNCSSCMDGTSWGQFRARMMRCVREYPFDGVLWYPFGVLSENTLMQ
RFLETTLQTVPLYFVHYVSKLCGIKSRPSMTTVSKRLHAMNEALKFFALREWHFNTDNVQ
QLMHRLAPADAAVYNLDPGTIDWESHCEDFVKGTRKYLLREKDQDI
>ATJ44465.1 fatty acyl reductase 10 [*Helicoverpa armigera*]
MAPSVSVAEYYAGKTLFITGATGFMGKVMVEKMLRSCPDVKKMYLLMRPKKGHSSKER
LDDLLSFKIFDRLKAENPKIFDKLHVIPGDILSEDLGISDEDRRLIQNEAQVIFHCAACVRFD
MFLRDAVKMNTVGTKKVLQLAEGVKNLEAFIHVSTSYCRCELPELEEKLYPSKHRPEHV
MHCVSWMDDDLLTHLQPKIIEPQPNTYAYTKSLTEDLVSQYEGKFPIAIARPSIVAAAYKEP
LPGWVDNLNGPTGLLVGAGKGVIRTMHCNDSYTADVVPVDVTVNACIILGYLTGLEQPK
QINVVNVTQSEINPITWGQALDMGRVHVQEFPFTVCLWYPGGSPKSSRLAHQLALFFTHL
LPAYLVDMLMFLMGKKTFMINLQKRISHGLNVLQYYTTKEWHFRNDNYKSLRTRITSQE
NDTFYTDPSQLDPDEYLKNYVLGTRQFCCKEDPANLPRARKLHKIRYIADRFIKALFIILIL
WTLYSHSQTFTSSVELLDSTLKSIPPISTAAQEAYDIQP
>ATJ44464.1 fatty acyl reductase 7 [*Helicoverpa armigera*]
MTSEVNEWYKGRSVLVTGALGLMGKVLIEKLLYSVPDLGCVYALVRSKRGKSPETRIEEM
WKLPLFQRIREEKPHVMKKLIPVTGDIMYDELGISADRLNDIYNEVSIVFHFAASLRLEAPL
KEGLEMNTKGTLRVLTMAKKMKKLVAFLHLSTAFCYPDYERMAEKVFDPPADPHEVLRA
ASWLTEEQLNLLAPSIYQKHPNSYTYSKRLAEALVRESYPELPAVVVRPSIVTPSYKEPTPG
WVDNLNGPIGLMVGAGKGVIRSMHCYGHYHAEVIPVDIAINSIVVIAYKTGKDTQRQPEI
PVYNLTTGDDRNTTWKQVLDIGKATVRKFPFEGPLWYPDGNIRHNKFIHDLCVFFYHIVP
AYFIDFLLFIFRQRRFMVRIQNRITIGLEVLQYFTTREWWFDTNNFKSLVGLLNPVDKETYP
MDLTIIEDEPYIESCMIGGKLYCLKEKLENLPKARLQNHILYILDRLVSLVFYLVLLYWIVSY
FEPARELLSYGGPAVRYLPLVGKAVFRDA
>ATJ44463.1 fatty acyl reductase 5 [*Helicoverpa armigera*]
MAAPQLISVSDVISDNLKNEKEINYCSKCIDNVDENMNNTNENLTEVQKFYNGKNILITGA
TGFLGKILVEKLLRCCPGVENLYLLVRQKRGKDIYTRMEEIFDDPVFSRLKDEVPKFRHKV
VVVPADCEAAGLGLTLTDRQMLTEKVNIIFHSAATVKFDEHLRAALLTNVKAPLHLLRLA
RDMKKLDVLMHISTAYSNSHLPEIEERYYPCEADCEQLHQMIDKMSDNEINKILPKILGPW
PNTYTFTKALAEKELRENAGGMPIGIFRPAIVISTAKEPLKGWLDNMYGPTGVAVGSATGI
LRTLQCDEMVSADIVPVDSVVNCLMVAACSVHHSYKQSSPPLEPPIFNYVSSVENRITWGE
FMLQNMAWIHYYPFSEAVWFISLRLTKSALMNKIYVLFLHLIPAALVDGLAVCLGRKPKM
LKVYRKIHKFSSVLSYFCTREIKFCNSRTRELWEKTSEADKQIYPFSMSEMNWEEYFQHYL
GGIRRFLFKESDDTLPQARIKWKRLYYLHQIARFIFFILAVYCLWWILSLIW
>ATJ44461.1 fatty acyl reductase 3 [*Helicoverpa armigera*]
MMDEFLFHRAHEVNPKGIHKVVPIVGDMELPGLGISEEDRKMLTSKVTIIINAAATVKFDE
KLSVSTAINVKGTKEVLKLAKECRNLKAITHVSTAFSNTHVNHIEERFYEPPMSVEALEAL
TEVDNKLIESILPTLLGKRPNTYCFTKAIAEEAVRKYGEGLPISIVRPSIVVSTYEEPVRGWT
DSVYGPTGLVVGIGTGVLRTMYMDQSKVADMVPVDLCVNAILASAWFTAKNFKENQTS

HIPIYNFVSGAQKPITWGDFIERNRKYGIDKPTTKAVWYYGLNPTNNYYLFLFYNFFLHYL
PALMIDTYCAITGKRRAMIKLYNKVMKLANILFYFSTQDWQFSDYNVRNMWKSLSDEDR
VVFPFSIGEMSWEYMCETFLVGLRVYLIKDDLSSLPEARKKWNKLYYLHQILKAVTLGLVI
NLAYFVLKPVLALVFGH

>ATJ44460.1 fatty acyl reductase 6 [*Helicoverpa armigera*]
MLVDMITQNQLFEAKQGGDITFMDMVDEREALGESQIQKMFAGSSVLLTGGTGFLGKLV
LEKLLRSCPDLKKIYLLARPKKNKDITKRLQEQFDDVLYDKLRKECPNFIQKIRIVEGDMG
QLELGMCPEDRIKIMNEVDVIFHGAATVRFDEPLKTAVEINVRGTREMFKLARGCTKLKA
FVHISTAYSNCPQTNIDEKFYESPLPGDKLIDMVETIDARTLDSITPGLLGDFPNTYAYTKAV
AENIVLEYSQGLPVALFRPSIVIGTAKEPVSGWIDNVYGPTGVVVGAAVGLLHVLNCDAK
VVADLVPGDMVVCACIAAAWRTARDSRSNHEDAPPPDLPPPVYNYVSSEQKPLTWEKFM
HYNEVYGFQVPTVQAIYYYLFTITSSRFLYTLYCFLLHWVPAYIIDGIAVIIGKKPMLRKAY
TKITKFSEVMAYFATREWKFDNSNTQKLYSEMCEADKHLFDFDMSTMDWNDYFYNYIRG
VRVYLLKDPVDTVPAGLKKLNRLRLLHYTFCAILGLLFLRLIWAIFSGILGFSF

>ATJ44459.1 fatty acyl reductase 4 [*Helicoverpa armigera*]
MADESQVRAFYAGKNFFITGGTGFVGLCLIEKILRCMPDVGKIYLLMRPKKGKEISERLEE
FPKNPVFEKLLESHSTDIFKKLIPVSGDVGEANLGLSPADRQMLIDNINVVIHSAATLDFQE
SLRPTVNINLLGTRRIMELCKDAKDLKVMIHVSSAYVNSFLTEAHEKVYEAPEDAQKVISL
VETLNDESLLQIEHKLLKSHPNTYTFTKHLAEHEVIKCIDMFPCTIVRPTMIVASWKEPIPG
WTCSKVGPQGFLMGAAKGVVRRLPLAKEKVADYIPVDVVINQLLVAGWEAAKSKSGLT
VYHCSSSTCNPFTWTMLDNTVNNMLHKYPLKSAVWYPHLKFVPTLLMFRISAIFVHFFPA
FLLDLMLRVTGGRPILIRLHKNVWNSLNRLERFIFSEWKFYNPNTLELATKLSKKDKELFY
IDVTSLQWVEYFSTLHLGVRRYLNKEKESSLPAARNKDMVLLVFHVIWQLFIMGLLWYIF
AWQTGLTLATSAWIAPIIYVLYNLL

>ATJ44458.1 fatty acyl reductase 2 [*Helicoverpa armigera*]
MASETMSTADLESLPDRIADTFSGMRVLVTGGTGFMGKVLLEKLLRKCPDIGQIMLFVRN
KKGKNPKQRLEEIFNGVLFEKVRTMRGGVEPLLQKVTLVAGDVSEPDLGLSDQDRAMIID
QVDIIIHAAATIRFDEELKKAVLLNVRGTKLMVELAKTCKNLKLFIHISTSYCHLHEKLLEE
QAYPPPADPHHVIQAVEWMDDEAIAMLTPKLLDKLPNSYAFTKALGEALVVEAMEHIPAM
VLRPSIVIPIWQEPVPGWTDNINGPTGLLIGAGKGVIRSMYCKSNSYADYLPVDVFINGIMI
VAWNYLQNGDTKCNIINFTSSAEIKVTWSEMIDAGREIIMNRVPLNGVVWYPGGSMKHSR
LYHNICLVLFHWVPAVLVDILLFCLGYKPILCRVQRRINKGFEVFEYYTNNQWDFKSDIAQ
TLRQKLNPRERRDYKVDAVGLDISKYFEDCIRAARVFILKEYDDTLPAARRHMRVMYWV
DVIVRCLFWGLILYWLSGFVTSPSTVTEHNATVIAMDA

>ATJ44462.1 fatty acyl reductase 9, partial [*Helicoverpa armigera*]
MRKNLRGHPLDNTFWYPSGYSVENKVTQKAMETLLQTLPLHIAEYVTKLLRIKTRMSLIT
VSQRLKAMNEVLRFFSVREWHFETNNVKRLQARLTPQDAAIYNLDPQTINWDDHYENFV
KGTRKYLLKEKDQDIQEARKHLRKMYYVHYG

> fatty acyl reductase 1 [*Helicoverpa zea*]
MVVLTSKETKPSVAEFYAGKSVFITGGTGFLGKVFIEKLLYSCPDIGNIYMLIREKKGLSVS
ERIKQFLDDPLFTRLKEKRPADLEKIVLIPGDITAPDLGITSENEKMLIEKVSVIIHSAATVKF
NEPLPTAWKINVEGTRMMLALSRRMKRIEVFIHISTAYTNTNREVVDEILYPAPADINQVHQ
YVKDGISEEDTEKILNGRPNTYTFTKALTEHLVAENQAYVPTIIVRPSVVAAIKDEPIKGWL
GNWYGATGLTVFTAKGLNRVIYGHSSYIVDLIPVDYVANLVIAAGAKSSKSTELKVYNCCS

SACNPITIGKLMSMFAEDAIKQKSYAMPLPGWYIFTKYKWLVLLLTMLFQVIPAYITDLYR
HLIGKNPRYIKLQSLVNQTRSSIDFFTSHSWVMKADRVRELFASLSPADKYLFPCDPTDIN
WTHYIQDYCWGVRHFLEKKSTNK
> fatty acyl reductase 2 [*Helicoverpa zea*]
MTSEVNEWYKGRSVLVTGALGLMGKVLIEKLLYSVPDLGCVYALVRSKRGKSPETRIEEM
WKLPLFQRIREEKPHVMKKLIPVTGDIMYDELGISADRLNDIYNEVSIVFHFAASLRLEAPL
KEGLEMNTKGTLRVLTMAKKMKKLVAFLHLSTAFCYPDYERMAEKVFDPPADPHEVLRA
ASWLTEEQLNLLAPSIYQKHPNSYTYSKRLAEALVRESYPELPAVVVRPSIVTPSYKEPTPG
WVDNLNGPIGLMVGAGKGVIRSMHCYGHYHAEVIPVDIAINSIVVIAYKTGKDTQRQPEI
PVYNLTTGDDRNTTWKQVLDIGKATVRKFPFEGPLWYPDGNIRHNKFIHDLCVFFYHIVP
AYFIDFLLFIFRQRRFMVRIQNRITIGLEVLQYFTTREWWFDTNSFKSLVGLLNPVDKETYP
MDLTIIEDEPYIESCMIGGKLYCLKEKLENLPKARLQNHILYILDRLVSLVFYLVLLYWIVSY
FEPARELLSYGGPAVRYLPLVGKAVFRDA
> fatty acyl reductase 3 [*Helicoverpa zea*]
NTYSYTKAITEDLVDEYSGKFPVAIARPSIVTAVWKEPIPGWVDNLNGPTGLVIGSGKGVIR
TMHCEPSYKADAISVDVVANACILIGYVTALDKPKETQVYNLTLSGVINLTWAEIIQLGEK
WVNEFPYSVALWYPGGSIKSYRLAHQLDVFLSHVVPAYLVDALLFLLGKKTFMINLQKRI
SHGLNVLQYYTTKEWHFRNDNYKSLRTRITSQENDTFYTDPSQLDPDEYLKNYVLGTRQ
FCCKEDPANLPRARKLHKIRYIADRFIKALFIVLVLWTLYSHSQTLTSSVELLDSTLKSIPPIS
TAAQEAYDIQP
> fatty acyl reductase 4 [*Helicoverpa zea*]
MSSPSIRDFYKGRNILVTGGTGFMGKVLIEKLLYSVPEVGNIYILMRPKRGKSVSQRTEDL
QRLKLFERLRTENPNALKKLKPLQGDVLFDNLGLSDADIELLTKEVSVVFHFAATLRLEAP
LKDNVNMNTCGTQRAIDLAKRMKKLQIFVHLSTAFCYPDYEVLGEKCHAPPVKPDNVM
KLIEWLDDKQVDLLTPSLLGPHPNCYTFSKRLAENIVEQAYQDLPIVIARPSIVCPSVKEPM
PGWVDNLNGPVGVMLGAGKGVIRTMLCDGSLVAQVIPVDIAINAIIAIGMIEGSRTEKPES
LPVYNVNNGHQKPTTWGDVLSVAKAYGRKYPLSWPLWYPNGDITTNPILHEYKRIFYHL
VPAYLIDFLLFLLGQKRLMIRIQERISRGLEVLQYFTMRPWNFPCPNYDAVREKLNPEEQAI
FNTDIKDVDRDEYMKICIEGGRVFCFKEDPNKVPMNRAYHNFLYVLDWFVKIMFWLFVF
SVLACWFSPVKSLFSLGEPVVKHLPFLGKVIEKQD
> fatty acyl reductase 5 [*Helicoverpa zea*]
MASETMSAADLESLPDRIADTFSGMRVLVTGGTGFMGKVLLEKLLRKCPDIGQIMLFVRN
KKGKNPKQRLEEIFNGVLFEKVRNMRGGVEPLLQKVTLVAGDVSEPDLGLSDQDRAMIID
QVDIIIHAAATIRFDEELKKAVLLNVRGTKLMVELAKTCKNLKLFIHISTSYCHLHEKLLEE
QAYPPPADPDHVIQAVEWMDDEAIAMLTPKLLDKLPNSYAFTKALGEALVVEAMEHIPAM
VLRPSIVIPIWQEPVPGWTDNINGPTGLLIGAGKGVIRSMYCKSNSYADYLPVDVFINGIMI
VAWNYLQNGDTKCNIINFTSSAEIKVTWSEMIDAGREIIMNRVPLNGVVWYPGGSMKHSR
LYHNICLVLFHWVPAVLVDILLFCLGYKPILCRVQRRINKGFEVFEYYTNNQWDFKSDIAQ
TLRQKLNPRERRDYKVDAVGLDISKYFEDCIRAARVFILKEYDDTLPAARRHMRVMYWV
DVIVRCLFWGLILYWLSGFVTSPSTITEHNATVIAMDA
> fatty acyl reductase 6 [*Helicoverpa zea*]
KDNETTTSITHHSSVPEFYAGKSVFITGGTGFLGKVFLEKLLYSCKDIVTIYVLIRDKKGKS
AQQRIEELVNKPLFTRLRSERPHDLKKLVPVVGDTSLPNLGISTEDEEILIQKVTAVFHVAA
NVKFHEELEIPVNTNIRGTSLVLDLCRRMENLEVFVHISTVFCHTSQKILEEKLYPPPAELSE

VFKYLEQSNQDRRQLKTLLNGQPNTYTFTKALAETYVAENHGNIPTVIVRPAIVTGSLKEP
LPGWVDHWLGATGLFAATAKGANRVLLGNPNYNLDLIPVDYVANLAIVAATRCRTDEVSI
YNCCTSSCNPITEGKIYEYLQIVCKNNGFDIPRLIFTENKLILSIQTFLLQTTPAYFMDLVRRV
RGKKPMYMKIQSQVVSVRNVLNYFTSRSWEMKADRTRELHASLSSEDRLQFPCDPCHID
WEDYTNVYLKGIEQFLMVRK

> fatty acyl reductase 7 [*Helicoverpa zea*]

LHSADHYIPVDVVINQLLVAGWEAAKSKSGLTVYHCSSSTCNPFTWTMLDNTVNNMLHK
YPLKSAVWYPHLKFVPTLLMFRISAIFVHFFPAFLLDLMLRVTGGRPILIRLHKNVWNSLN
RLERFIFSEWKFYNPNTLELSTKLSKKDKELFYIDVTSLQWVEYFSTLHLGVRRYLNKEKE
SSLPAARNKDMVLLVFHVIWQLFIMGLLWYIFAWQTGLTLATSAWIAPIIYVLYNLL

> fatty acyl reductase 8 [*Helicoverpa zea*]

MGFLEDLDLSSVPSIAEFYKGKTIFISGGSGFMGKVLVEKLLYSCPDLDRIYLLLRNKKGV
KSEDRLAQLLSSLCFDRLRRERPSFASKVFVIAGDVLEVGLGLSEEDRTLLVNRVNIVFHVA
ASVRFDDPLEYAVRMNLRGTKEMVELAADMRNLCSFIHVSTSYSNTNRDPIEEILYPPHAD
WRDTLDVCEKTDPHILKVLTPKYLGELPNTYTFSKQLAENVVAEYAGKLPVVIIRPSIVISS
VEEPVPGWIENFNGPAGLLVACGKGIMRSLYTDPDLIADYMPVDISIKSFIVASWLRGTKEL
SPSDDVPIYNCCAGKLNNITMGEMVTIGRQIYPSVPLNDMLWHVGGDLTTSKTV

> fatty acyl reductase 9 [*Helicoverpa zea*]

CYAADIELAFYRVNYICRNVNYERLEEMLKDPVYNMVRKKKSNFAEKIIPVAGNVADIRL
GMSDQDWAIVTKEVNVIFHMAATTRFDEALKIATMINVRGTREAVLLGKACQKLKSFVY
VSTTYANATDNFVEKEVLETFYPPPVPPELMISMAETIDEDRLQGIEHDLIKGYPNTYTFAK
AIAEEVVRSRAGNMPISIVRPAVVISSYREPMPGWADTSCAYGASGLILGPATGLIHATYAG
DNTRYSLVPVDYVNNAILAAGWKTSSMPGDVKIYSVSSARNLFHWQPVSTKIREIGRVLP
TPLAVWYMFIINTANKPLFVLLTWLLHYIPGYILDGGCVLLGKPPMFIKLYNRVYRASLAL
SYFTTHSWLFRDDNTDKLFQDLSTEDKLIFNFDTTNINIMEYVTLWCVGLRKYLMKDGIK
NTEYAIKKQFWLQKLHYIVAALYVYVLYKICSYVLFVVLLLFGWV

> fatty acyl reductase 10 [*Helicoverpa zea*]

MAAPQLISVSDVISDNLKNEKEINYCSKCIDNVDENMNNTNENLTEVQKFYNGKNILITGA
TGFLGKILVEKLLRCCPGVENLYLLVRQKRGKDIYTRMEEIFDDPVFSRLKDEVPKFRHKV
VVVPADCEAAGLGLTLTDRQMLTEKVNIIFHSAATVKFDEHLRAALLTNVKAPLRLLRLA
RDMKKLDVLMHISTAYSNSHLPEIEERYYPCEADCEQLHQMIDKMSDNEINKILPKILGPW
PNTYTFTKALAEKELRENAGGMPIGIFRPAIVISTAKEPLKGWLDNMYGPTGVAVGSATGI
LRTLQCDEMVSADIVPVDSVVNCLMVAACSVHHSYKQSSPPLEPPIFNYVSSVENRITWGE
FMLQNMAWIHYYPFSEAVWFISLRLTKSALMNKIYVLFLHLIPAALVDGLAVCLGRKPKM
LKVYRKIHKFSSVLSYFCTREIKFCNSRTRELWEKTSEADKQIYPFSMSEMNWEEYFQHYL
GGIRRFLFKESDDTLPQARIKWKRLYYLHQIARFIFFILAVYCLWWILSLIW

> fatty acyl reductase 11 [*Helicoverpa zea*]

CRKFVKMADESQVRAFYAGKNFFITGGTGFVGLCLIEKILRCMPDVGKIYLLMRPKKGKE
ISERLEEFPKNPVFEKLLESHSTDIFKKLIPVSGDVGEANLGLSPADRQMLIDNINVVIHSAA
TLDFQESLRPTVNINLLGTRRIMELCKDAKDLKVMIHVSSAYVNSFLTEAHEKVYEAPED
AQKVISLVETLNDESLLQIEHKLLKSHPNTYTFTKHLAEHEVIKCIDMFPCTIVRPTMIVAS
WKEPIPGWTCSKVGPQGFLMGAAKGVVRRLPLAKEKVADYIPDRKSTRLNSSH

> fatty acyl reductase 12 [*Helicoverpa zea*]

LMIRRPPRSTRSEFYSPTIKPQLGMEADGMAQLKEVSIVFHSAATLKFDEPLRVAMEQNVR
SVERLLEICDKLPNIQAFIHVSTAYSNAELTRVEERVYAPPVPLAQALTVADSVPEHLLATIN
AQYIAPKPNTYTFTKALAETVVEEHGNRGYPVAIFRPSIVISSLRHPFPGWIENLNGPSGVV
VGAGKGLLHVFCCRSRAGADMLPVDIAIDTLLAVAWETAVDRPEHVRVYNCSTCENPTT
WGDFEDALRKNLRGHPLDNTFWYPSGYSVENKVTQKAMETLLQTLPLHIAEYVTKLLRI
KTRMSLITVSQRLKAMNEVLRFFSVREWHFETNNVKRLQARLTPQDAAIYNLDPQTINW
DDHYENFVKGTRKYLLKEKDQDIQEARKHLRKMYYVHYGFLFFVVTLICRLMLQNHYIR
TLVFRTFKLLLTVIGSVFMRIQS

> fatty acyl reductase 13 [*Helicoverpa zea*]

MAPSVSVAEYYAGKTLFITGATGFMGKVMVEKMLRSCPDVKKMYLLMRPKKGHSSKER
LDDLLSFKIFDRLKAENPKIFDKLHVIPGDILSEDLGISDEDRCLIQSEAQVIFHCAACVRFD
MFLRDAVKMNTVGTKKVLQLAEGVKNLEAFIHVSTSYCRCELPELEEKLYPSKHRPEHV
MHCVSWMDDDLLTHLQPKIIEPQPNTYAYTKSLTEDLVSQYEGKFPIAIARPSIVAAAYKEP
LPGWVDNLNGPTGLLVGAGKGVIRTMHCNDSYTADVVPVDVTVNACIILGYLTGLEQPK
QINVVNVTQSEINPITWGQALDMGRVHVQEFPFTVCLWYPGGSPKSSRLAHQLALFFTHL
LPAYLVDMLMFLMGKKTFMIKIQKRINYGLEVLQYYTTKEWFFRNDNFVALQHRISKSDN
ETFYTDMKDMDWSGYIRNYIRGAREYCCKEDPATLPAARRLQTQLYYLDKAVQIMVGLL
VSYFIYYYFNMLYSVISS

> fatty acyl reductase 14 [*Helicoverpa zea*]

MNHTVEESPLMQTPMTQEKMDKWVDAQIKGEKIEIDVYGKPTEQMLKELENVRNLSKE
LQDNLHELETSVRIAEVENQAMNPTAEILDFSEDHEFVPDNQDTYYAEEDKMDAKEEEKQ
KLTKGKGAKTEIQSFYKDQCVFLTGGTGFLGKVLIEKLIRACGDINTIYVLARSKKGKDAT
VRLHEMMDEFLFHRAHEVNPKGIHKVVPIVGDMELPGLGISEEDRKMLTSKVTIIINAAAT
VKFDEKLSVSTAINVKGTKEVLKLAKECRNLKAITHVSTAFSNTHVNHIEEKFYEPPMSVE
ALEALTEVDNKLIENILPTLLGKRPNTYCFTKAIAEEAVRKYGEGLPISIVRPSIVVSTYEEP
VRGWTDSVYGPTGLVVGIGTGVLRTMYMDQSKVADMVPVDLCVNAILASAWFTAKNFK
ENQTSHIPIYNFVSGAQKPITWGDFIERNRKYGIDKPTTKAVWYYGLNPTNNYYLFLFYNF
FLHYLPALMIDTYCAITGKRRAMIKLYNKVMKLANILFYFSTQDWQFSDYNVRNMWKSL
SDEDRVVFPFSIGEMSWEYMCETFLVGLRVYLIKDDLSSLPEARKKWNKLYYLHQILKAV
TLGLVINLAYFVLKPVLALIFGH

> fatty acyl reductase 15 [*Helicoverpa zea*]

MGQLDLGMCPEDRIKIMNEVDVIFHGAATVRFDEPLKTAVEINVRGTREMFKLARGCTKL
KAFVHISTAYSNCPQTNIDEKFYESPLPGDKLIDMVETIDARTLDSITPGLLGDFPNTYAYTK
AVAENIVLEYSQGLPVALFRPSIVIGTAKEPVSGWIDNVYGPTGVVVGAAVGLLHVLNCDA
KVVADLVPGDMVVCACIAAAWRTARDSRSNHEDAPPPDLPPPVYNYVSSEQKPLTWEKF
MHYNEVYGFQVPTVQAIYYYLFTITSSRFLYTLYCFLLHWVPAYIIDGIAVIIGKKPMLRKA
YTKITKFSEVMAYFATREWKFDNSNTQKLYSEMCEADKHLFDFDMSTMDWNDYFYNYIR
GVRVYLLKDPVDTVPAGLKKLNRLRLLHYTFCAILGLLFLRLLWAIFSGILGFSF

> fatty acyl reductase 16 [*Helicoverpa zea*]

TRNFIYVSTAFSHATYDRVNTEVLDQFYPCPVQPETIIGMAESMEEDRLNSIAEDLIVGWPN
TYTFTKAIAEELVRTYDPDLPVCVVRPPIVTPTYYEPTPGWMDLSALSGPTGVLAGIIMGF
LHIFYVDKDCKLPLTPVDYVNNATIAAAWDADLKRKSGNKDIQVYTVSNNDHFITWDFIG
VILRSEGKKSPSPKAVWYCWLMETNSKFVFWVISFFVHYIPAYIMDLVGGILGNMPKEINS

FVAVFRKIDKFALIYHYFLSNEWFFKDHNVQEMVSRMSPADKAIFNCDLRTIDFTEYVMIW
GIGIRKYLVKDELKDSELAYRKQQKLKIANIFFISLNVIVVLSLLYQLFKVVIWLF
> fatty acyl reductase 17 [*Helicoverpa zea*]
QSADPVAWETATQRQETRSVRVYNVCSQENRVSWRILYYPTLIAVRNWYLYKFLELVLQT
VPLHIVDCVTRACRTPMKVRLSSVPARLRDMSAALSYFATREWRFDSHNVTHLQERLTQA
DREIYNLDVNTVDWEEHLTDFVKGVRAYLLRESDAELPRARRHMHRLRVVHRAVLLVAH
AALCALLVYVALALYHVITS
> fatty acyl reductase 18 [*Helicoverpa zea*]
MVPRPAPQSSTPPLIPEFFAGREVFITGATGFMGKVLVERLLWTCRDISRLHLLLREKKDVA
PEKRLSQLKQSQVFDVIRQHCPKQLDKLSMLAGDVTKRRFGLDNHAISQLNQVSVVFHS
AATLKFDEPLPVALQQNVHSVVTLMDICDQLPNMQVLVHVSTAYSNAELTSVEERVYPPP
AQLQQLSALVEALPADLLAEITPQLISPKPNTYTFTKAMAESVVAERANSTNYAVAIFRPTI
VISSLRHPFPGWIENLNGPSGVVVGAGKGLLHVLSCGAVRRADMMPVDIAIDTLIAVAWE
AANDQPGFARVYNCSSCMDGTSWGQFRERMMRCVREYPFDSVLWYPFGVLSENTLMQR
FLETPLQTVPLYIVHYISKLCGIKSRPSMTTVSKRLHAMNEALKFFALREWHFNTDNVQQL
MHRLAPADAAVYNLDPGTIDWESHCEDFVKGTRKYLLREKDQDIEAARRRMHVLHMIHS
LTKILLTVLMVRLAYRSTPAILRAVAVLTRLRRRGATVALSG
>fatty acyl reductase 19 [*Helicoverpa zea*]
MASCLSGGHYVPGSQEYVPVAEFYADKSVFVTGGTGFMGKVLVEKLLRSCPKIKKIYLLM
RPKRGQDVASRLTELTQSPLFETLRRERPQELNKIVPIVGDITEPELGISAADQTMLCQKVS
VVFHSAATVKFDEKLKLSVTINMLGTQQLVQLCHRMLSLEALVHVSTAYCNCERERVEET
VYAPPAHPEHVVTLVQTLPDELVDRITPDLVGDRPNTYTFTKALAEDMLIKESGNLPVSIV
RPSIVLSSLREPVKGWVDNWNGPNGIIAAVGKGIFRTMLGTGTKVADLVPVDTVINLMIVC
AWRTHLRRGEGVVVYNCCTGQQNPITWQRFVKTSFKYMRKHPFNEVLWYPGGDITSNR
LKHGTLSLLQHRAPAALMDLVSTATGKKPMMVRVQNKLEKAAACLEYFTTRQWAFADD
NVQALCASLSPDDRRTFDFNVRNIDWDAYIESYVLGIRRFLFKESPDTLPKSRAVLRRLHIV
HILTQVATVFFLWRFLFSRSNALRNVWRRVLELLTRAARLLAIA
> fatty acyl reductase 20 [*Helicoverpa zea*]
FVLKVFKRLKVSNPESIKKVVPMIGDVTLPNLGLNQKDEETLVDKVSVVYHAAATIKFNE
PLQVAMNINFEGTQKILELSKRMKNIEAFIYISTAFTNTSRRVLLETVYPPPAKVDDVYKFIE
EYGHDAQETKKFLNEQPSTYTFTKSLSEAYVAKNHGDVPTVIIRPSVVSSAKDEPLKGWL
DNWYGGTALVQNAGRGWNRFALGNNDAPLDLIPVDYVSNMSVIAAAKAKKSSDVQVY
NCTSSAENPIIWSDVHKYFNREMVALGKNEIPCPHVIYLKSKLLMNIGTFVFQTTPAQIADF
WLKITGREPKYTETLSKVLKVRDGYEFFTANSWVMKAERARELYSSLSPEDKEEFPCDVT
QIVWSEFMRDYCRGILKYISSKTNGK

ACT

>ALJ30248.1 putative acetyltransferase ACT1 [*Spodoptera litura*]
MSVAAKGIFIVGAKRTAFGTFGGVFRNTTATELQTTATIAALKEAGVAPEKVDSIVVGQVM
TASQTDGIFIPRHVMLKAGIPQDKPALGVNRLCGSGFQSVVNSAQDILTGSAKISIAGGVEN
MSQAPFAVRNVRFGTALGSSYAFEDTLWAGLTDSYCGLPMGMTAEKLGAQFGITRDEVD
NFALRSQQRWKAAQDAGVFKAEITPVTLTVKRKEVKVEVDEHPRPQTTIEGLKKLPPVFK
KEGLVTAGTASGISDGAGAIVLAGEEAAKGLKPLARLVGWSYVGVDPSIMGVGPVPAIEN
LLKVTKMSLNDIDLIEINEAFCAQTLACAKALKLDMEKLNVNGGATALGHPLGASGSRIT
AHLVHELRRRGLKRGIGSACIGGGQGIALMVETV

>ALJ30249.1 putative acetyltransferase ACT2 [*Spodoptera litura*]
MISLSILKQSTIVHLCFAISYFTSGLILTFIQAILYFGLRPFNKSLYRKINYYLAYSFYSQLVFM
SEWWSNSKLIIYIKKDEYEKYYGKEHGYLIMNHSYEIDWLMGWHFCNTIGVLGNCKAYA
KKSIQYLPPIGWMWKFSEFVFLERSFEKDKETIKHQISELCDYPDPVWLLMTPEGTRYTKK
KHEASLNFAKEKNLPLLKHHLTPRTRGFTTSLQFFRGKIPVIYNIQLAFEKDSKTPPTLTSLL
YGKPVNAHLYIERIPVEKVPEDEGEAAKWLHELFVVKDKMQDSFFNTGDFFLESGVERRE
SFTVPPPIWSLVNALGWAVVTLTPMLYYLLGLLFSGKLLYFSIACAIFGAFFILLQKSIGMSK
ISQGSSYGTEKK
>ALJ30250.1 putative acetyltransferase ACT3 [*Spodoptera litura*]
MSSRKPLTKSQKLQYEKKYEKRSLYIPIKYFLIAIVILLAASYKLYFVKTDYEMPEIDLEQW
WGSYPMNEIDTSIRPFTIEFSDVKVNDLKERLLHRAQFAPPLDSAGFSYGFNSLFLPKVLDF
WQKEYNFEERERFLNKYNHFITGMQGLDVHYMHVKPDLGVGDDITVVPLLLIHGWPGSI
REFYELIPKLVTPRPNHKFVFEVIAPSIPGFGYSQAPVQQGMGAKEVAVVFYNLMKRIGYT
KYYVQGGNYGAKIGSVMATLFPDSVLGFHTNTPSVSWSPMSIFYTLLGTIWPNFIVEPSLA
DRMYPLSQYLSTIVQETGHFHMQATKPDTVGIALSDSPAGLAAYILEKFSAWTNIENKQAI
DGALLQKFSLTHLLDNVMIYWTTNSITSSMRHYTEHKQLWTLDRVPTDVPTWGIKFKYN
LCFQPDSILRLKYRNYLHSSIVEDGGHFAAMELPDILADDIFDAVDTFIKFNEERNKSGPLP
EPVESNAQQTKSKTASTEPTEKPTESAKKPTEPAKQPNESAKQPSEPANKPTKPVQQPTEPS
KQPTQVDYMKAKSVHEFTVKDINGNEVKLDRYKGQVLIIVNVASNCGYTNVHYKQLNEL
YEKYSNKGLRILAFPCNQFAYQEPGSPEEILQFTKAKQVKFDLFEKVAVNGEDAHPLWNFL
KRMQGGTLGDFVKWNFSKFIVDKNGVPVERFGPNTDPLELVPYLEKLFDQ
>ALJ30251.1 putative acetyltransferase ACT4 [*Spodoptera litura*]
MSANVILGCVMALVIILFTISSMARYYIKFTLFIVMSLIFAAAPMPLMLIKPFDPRNALIPAFF
LRCFARILGLRWKVRGLENVDNSRGAVVLLNHQSSLDLYALAIIWPLMSRCTVVSKRSLQ
YLVPFGTATWLWGTVFIDRGAKSARDALNKQVDAIKNEKRKLLLFPEGTRHSGDRLLPFR
KGAFHVAMDAGAPIQPVVISKYHYLDGKRHKFGSGEFIVSFLPMIETEGLTKDDIVSLVDK
TQLNMQEEFTKISMETLERRNRIKAD
>ALJ30252.1 putative acetyltransferase ACT5 [*Spodoptera litura*]
MAPSNLSLNEVVIVSAVRTPIGSFKGSLANVTATELGAIVVRAAVERAGIPSSEVKEVFMGN
VCSAGLGQNPARQAAIFGGLEKSTICTTVNKVCASGLKAVTLAVQGLQTGANDVILAGG
MESMSNIPFYIRRGEIPYGGTQLLDGILYDGLTDVYDQIHMGDCAENTAKNLNLSRKQQD
DYAIISYKRSAAAHAAKAFDAEVIPVPVPQKKGGAPVIFAEDEEYKRVDFDKLVKLPTVFK
KENGTVTAGNASALNDGAAAVVMMTAEAAKRLNVKPLARVIGYADGEREPIDFPIAPSVA
IPKLLEKTGVKKENVAMFEINEAFSVVTLGNQKLLDIDLEKINVHGGAVSLGHPIGMSGTR
IVGHLCHALKKGEIGVATACNGGGGASAIMIEKL
>ALJ30253.1 putative acetyltransferase ACT6 [*Spodoptera litura*]
MDSKTTKMPKVAKVKNKAPAEIQITAEQLLREAKERDLEILPPPPKQKISDPEELRDYQHR
KRKAFEDNIRKNRLVIGNWLKYAQWEESQKQVQRARSIYERALDVDHRNVTLWLKYTE
MEMRNRQVNHARNLWDRAVTILPRVSQFWYKYTYMEEMLENVAGARQVFERWMEWQ
PDEQAWQTYINFELRYKELDRARQIYERFVMVHPDVKNWIKYARFEENHGFINGARKVL
ERAVEFFGDEDLDERLFIAFAKFEENQKEHDRARVIYKYALDHIPKDRNKELYKAYTIHEK
KYGDRSGIEDVIVNKRKYMYEQEVIENPTNYDAWFDYIRLVENEGNVDDIRDTYERAIAN
VPPSKDKQFWRRYIYLWINYALYEELEAEDAERTRQVYRTCLELIPHKIFTFSKIWLMYAQ
FEVRCKDLKQARKTLGMALGICPRDKLYRGYIDLEIQLREFDRCRILYQKFLEYGPENCIT

WIKFAELETLLGDTDRARAIYEIAVGQPRLDMPELLWKSYIDFEVQQGETEKARQLYERLL
ERTVHVKVWLSYAKFELNAENADNINVDLARRVYERANDSLRSAGEKEARVLLLEAWK
DFETEIGEEEKLEKVLSKMPRRVKKRQKIISESGVEEGWEEVFDYIFPEDEMVRPNLKLLA
AAKQWRKQKEVSQPPESESRNDHEERRENDDDDDDDSEEEEQTPPQPQNRNEKEDES
>ALJ30254.1 putative acetyltransferase ACT7 [*Spodoptera litura*]
MESMSNVPYYLKRGETPYGGIQLIDGIVFDGLTDVYNKFHMGNCAENTAKKLNISRQQQ
DDYAISSYKRSAAAYEAKAFADELVSVPVPQKRGAPPVLFAEDEEYKKINFEKFTKLSTVF
QRENGTVTAGNASTLNDGAAAMVLMTAEAAQRLNIKPIARVVGYADGECDPIDFPIAPAV
AIPKLLEKTGVKKDDVAMWEINEAFSVVAVANQKLLELDPAKVNIHGGAVSLGHPIGMSG
ARIVVHLCHALKKGEKGVASICNGGGGASSIMIEKL
>ALJ30255.1 putative acetyltransferase ACT8 [*Spodoptera litura*]
MNIRCARPSDLMNMQHCNLLCLPENYQMKYYFYHGLSWPQLSYVAEDEKGHIVGYVLA
KMEEDGEDNRHGHITSLAVKRSHRRLGLAQKLMNQASLAMVECFKAKYVSLHVRKSNR
AALNLYTNSLGFKILEIEPKYYADGEDAYSMMRDLSAFAAENKTEPQPTENLEIKSESAIIS
QC
>ALJ30256.1 putative acetyltransferase ACT9 [*Spodoptera litura*]
MLRRCSKHLQTLYRRQGQALRFKSTEAPKVFGALSQAAARTAQPRSLTAAHNQVATIHFT
NPLLAEQDIMTPSFPDSVSEGDAKIEKKVGDAVAMDEVVMEIETDKTALPVMAPGNGIIKE
FYVNNGDTVKAGQKLFRLELTEGGPPPKAAAAPAPEPPKAEAPPPPPPAAAAPPPPPPPAA
AAPPPPPPPPPAPAAAAPKPAAPISSIPVAAIRHAQSIETASVKVPPTDYSKEIAGTRTEQRVK
MNRMRQRIAQRLKEAQNTNAMLTTFNEIDMSHIMAFRKKHLDAFTKKHGVKLGLMSPF
VKASATALMDQPVVNAVIEDNEIIYRDYVDISVAVATPKGLVVPVVRNVQNMTYADIELTI
ANLAEKAKAGKLTIEEMDGGTFTISNGGVFGSLMGTPIINPPQSAILGMHGIFERPIAVNGQ
VVIRPMMYIALTYDHRLIDGREAVMFLRKIKQGVEDPATIIAGL
>ALJ30257.1 putative acetyltransferase ACT10 [*Spodoptera litura*]
MALIMSFVSVAISILYTPLLLLILCIIFLASIGKSLGVRRLYVNILLKLFEYGRQHIEVAKIKIQ
RTDSSDEEDLPPVPDDKPPSAIIKENGVNGTKMTVIERQEILGPSPELNYKRSTSQERVQNG
PKTTQGNGESNMEFDLSNCLDLVKAGMESIIEDQVTSVFEAEELRSWNLLTRTNRQYEFLT
WRLTIIWAMGFVVRYMFLLPLRIMIFVIGVWWLIACTACIGTLPDGKTKQRINYAVSVMCF
NFLSRCISAVITYHDAHYKPKNGICVANHTSPIDALVLMCDNCYSLIGQRHNGFLGILQRAL
ARASPHIWFERSEVKDRHAVAKRLKEHISVPDNPPILIFPEGTCINNTSVMQFKKGSFEVGG
TIYPVAIKYDPRFGDAFWNSSRYGMLHYLLNMMTSWAIVCDVWYLPAMTRAADESAVDF
ANRVKAVIARRGGLVDLMWDGQLKRMKPKKEWRELQQEEISKRLKGE
>ALJ30258.1 putative acetyltransferase ACT11 [*Spodoptera litura*]
MAFAGLKKQINKANQYVTEKMGGAEGTKLDLDFVEMERKTDVTCELVEELQTKTKEFL
QPNPTARAKMAAVKGISKLSGQAKSNTYPQPEGVLGDCMLLYGKKLGEDTVFSNCLIEM
GEALKQMADVKYSLDDNIKQNFLEPLHHLQTKDLKEVMHHRKKLQGRRLDFDCKRRRQ
AKGAHIADDEIRQAEEKFAESLQLAQIGMFNLLDNDVEQVAQLTFFAESLLEYHQQCTEIL
KGLVSTLMEKKEEAVNRPKMEFVPKTLADLHIEGIHDLNNGRRYGSTQSLSRPRQHIPPSS
SVGDLSTTDPFKAWEAPSPVRTQVRPAPGFKPHPAPRNQFNGRDPWTGDFSFICRVIYVWF
WLWIAGDGGGTWWKAVHGRNLTCT
>ALJ30259.1 putative acetyltransferase ACT12 [*Spodoptera litura*]
MSFLMRKCIVNLKNVNRCRTVCVFLQTERQLSRCSANILKRSILLSEVHLRHRQFHTSQIF
NKVVAFKLSDIGEGIREVVIKEWFVKVGDKVQQFDNICEVQSDKAAVTITSRYDGVVTKL

YHDVDQTALVGQPLVDIEVQGAADEASSESVEKPAAVNQQELKEEKPQRVKVLTTPSVRR
IAAQFKVDLSTVKATGRNGRVLKEDMLAHLNIDSDGSNRVSDPTSVDAVQIPMTPAQAKV
EVLLEDRVVPVSGFTKAMVKSMTEAMKIPHFGYSDEYDVSKLVESREALKNIALSRGVKL
TYMPIIIKATSLGLENIPVLNSSLDTTCEHLTYKANHNIGVAMDTPNGLVVPVIKNVQNKTI
LDIARELNTLQEKGSKGQLGLSELSGGTFTLSNIGIVGGTYTKPVILPPQVAIGALGKIQVLP
RFDAEGNIRKAHILTVSFSADHRVIDGVTMARFSNHLKNYLENPYTLLLDL
>ALJ30260.1 putative acetyltransferase ACT13 [*Spodoptera litura*]
MRQSGIILLTVLVVQAYSAPQFITFSEGKLGVNFGGYHAGVGLGGLAGGKGNTAGGLYAE
AGTPFGPAAKAGLGGAVDGSSGTAGGLYAGATAGGNVNAAAGLGGAVAGGKAVGGGYS
TAQSGGHSATSVLGGESGASGSAGFSVSAHKSVEVPVTVVKETEVSVIPVEEVKTVHKNV
YGETKYEASNEITPVAKAGVEATANVNVNAQQGFAKEVHVEPTEVVYVRKHKPHRHHV
HKAVYVGGFVGAGGEVAAPETKSVYKTVQPIEKRVDVEAHAEAHGEAGAGYNGGYYQS
PKVATVHKEVVVNAKPSTFFQDIFNIPISTLKAVSGFLTNTAENTGVSVQKSATFKAGGYSG
FSGNAGYSGHSGYSSYY
>ALJ30261.1 putative acetyltransferase ACT14 [*Spodoptera litura*]
MIGANKLISKNNVCQKIIQQRNFTKKNIKDANYQYLQRSKLPTMHFQKSLPRLPIPELSKT
GERYLNALRPLLTASQFEEAQQRTNNFITKEGKILQEKLTAKDKRNKHTSYISDYWFDLYL
RDRVPLPINYNPMIVFQNDVRPEYNDQLIRSTNILISAVRFMLSLREQILEPEVYHMNPKKS
DTPLFRNFTRMLPEAISWYGAYLFKVFPLDMSQFVGLFGATRLPRQNKDEIFRDPKSKHV
VVQRKGNFYVFDVLDADGNLLSPQEILGNLSKVINDNSPSSEYPLGVLTTQNRDQWAQQR
VHLESTGNSEVLRKIDSAIFNLVLDEDVINDDKRVLLRKYLHSDGTNRWFDKSFSLIVTGD
GVSGVNFEHSWGDGVAVLRFFQDIYAETTKKPFIHPESKPADSNISVQKLEFKLDDKSKQFI
DNAKKEYKAWCDSLSIDYILYEGLNKAACKKFKVSPDCIMQLSFQAAHHLLKGSFVGTY
ESCSTSAFKHGRTETMRPCTLKTKAFCETLHSNNRSDDDLRSKLTECSKLHLELVKESAM
GQGFDRHMFALMKMAEDNNMPRPEIFDSYEYKYLNKSILSTSTLSSPSVMAGGFGPVVK
EGFGIAYSAFPDKLGAAVASYKSHNNSTHYVEALHKSFLDITKILSA
>ALJ30262.1 putative acetyltransferase ACT15 [*Spodoptera litura*]
MIEKLAHTSDGPPVLTFYTKDPCGLCDIVMEELEPYKNRLIIQKVDITEKENIRWLRLYRHD
IPVLFLNGQYLCMHKLNKHLLEKRLQMIEEEKS
>ALJ30263.1 putative acetyltransferase ACT16 [*Spodoptera litura*]
MWYQIILFTTVCVFTYVLKKLHDTGPNKFKFYFNFFIFYFLTSVLSAVIWPYFLLSPKNVRN
SKIAVRLLKHITKLYDLEWHLRDGKILAEDRGAVIISNHQSSLDILGMFNIWDVVDKLAAI
AKKELFYIWPFGLSAYLAGVVFIDRSNAKGAYRQLKVTSEVMVKNKTKIWLFPEGTRNK
DYTKLQPFKKGAFNIAVAAQVPIIPVVFSPYYFINKEKYIFNKGHVIIQCLEPVPTTGLTMDD
VPDLINRVHEKMTIAYKELSKEVVSALPADYPFTLLG
>ALJ30264.1 putative acetyltransferase ACT17 [*Spodoptera litura*]
MMENLSSIVEALSKIFSQVSTLLGIQWAPMDIPMSRRLQTFAAFLWIYLILFGEAFAVYLFIR
LVYSKYWWAAILYGVWMLNDVEICNRGGRSSEWVRNWIWWRYLADYFPIKLVKTVDLD
PSKNYMFACFPHGVISLGAFGSFCTNALDFKKLFPGMTCHLITLGGHFLVPFFRELALALGI
CSSSEQSLLYLLDKKKYEGNCACMIIGGAAEALDAHPKEYKVILSRRKGFIRVAMKSGAA
LVPVFSFGETDLFRPPNNPENSLLRRFQEKVRQITGISPMFPMGRGLFQYSYGVLPIRAPVT
TVVGAPMEVKRNLEPTNEEIDAVHAEFTQRLQTLFETEKVKYLKYHEEARLVIT
>ALJ30265.1 putative acetyltransferase ACT18 [*Spodoptera litura*]
MGARSLKVLQVISGWQAVELILTCVFVGIWQIIEISVKRLWKGYRRKVDESQPVELTVDSS

IGTHCYIKVMGVKYHYVETGPRSGQKVLILKDAPDTGNLWGPNWANVVRRLAETDHHV
VTLDLRGTGGSEGGSRSELSPPRAVEELSALLKALGVSENRQAVVIGFGIGGMLTWYLVHT
RGPLISKFAVINAPHPNLYWQYPPATFCHRALQFIQWPHFPERWLAEGELNDREGRWTSSR
ACDWTGALNYVRGAAWWQVKQGLKTSAPALLVGNKDSAAQLVASAQHCTASTLRLVTK
PEPSSKEVTDVLLDFLIEKEKLIEEVPRGLMGRVFGAVADRGRELTARLVLPPTQA
>ALJ30266.1 putative acetyltransferase ACT19 [*Spodoptera litura*]
MVFDNNWTGGWFSWTRQSDAMLRNVEKKILSCLKTAYKRFYVDIGSVVGQSDKIWTISL
NEESPKTPLVLLHGMGAGLALWCPNLDSFAATRPVYAIDLLGFGRSSRPKFACDAQKAEA
QWVESVEEWRREVNLGQFILLGHSLGGYIATAYAIKYPERVRHLVLADPWGFAERPQNAY
EKAQLPLWVRAIATAVQPLNPLWAVRAAGPAGKWLVSKTRPDISRKYLNYVPDAERVIPE
YIYQCNSQTPSGESAFHTLMTGFGWAKNPMVRRVDELDPALPITVLYGSRSWVDNSSGQV
LVEHRGPSNTFVQVINGAGHHVYLDKPELFNKFVLDACTRADEHDPRPALKAVPAEPGTE
TPALPPGGEAPSTTVATTNKATASSDAAPTS
>ALJ30267.1 putative acetyltransferase ACT20 [*Spodoptera litura*]
MARRLLCRMILNSNTTSIKSSLPVLGKKLHSQVPTKEIQIPVKFGHIAGKLWGNSSERPILA
LHGWQDNAGTWDPLIPMIIKDRPILALDFPGHGFSSWIPPDMQYYQWELPRIILYLKEYFK
MEKVSILSHSMGAIASMRFASVFPDDVDFYIAVDSLIYDDYDLDAVVGKIPTTLKKALIAQ
TRLNDEPPAYSLEEMTKIWHLGTRKSVALESVQHLLKRGIKPSKADPNKYYFSRDSRLKY
TLFNPEDKKFVEALVRRLKCPTLYIKAIDSPYSSDAYSIEMREILEQNNENYEFHFVPGTHH
VHLNNPELVAPLIKNFIQNHNLSL
>ALJ30268.1 putative acetyltransferase ACT21 [*Spodoptera litura*]
MAVVINKGIFIVAAKRTPFGRFGGAFKEVYPSDLLAVAAKDALKAGSVAPEVIDTVNIGQV
YGLSGSSDGGLSPRHAALKAGIPQEKPALGISRLCGSGFQAVVNSAQDIITGAAQTSLAGG
TENMSTVPFVVRNTRFGVGLGVKMPFEDLLTTSSLDTSCNFTMPQTAENLAEKYGLQRM
EVDQFALQSQQRWKAAHEQGVFKAEMAPVTVRVKKQDKVVEVDEHPRPETTTEMLSRL
PVLFRKGGVVTAGNSSGVNDGAGAIVLASEESVKQNGFTPLVRLLAWSAVGVDPSIMGIG
PVPAIQNILSATGLKLDDIDLIEINEAFAAQTLACAKELGLDQSKLNVNGGAIAMGHPVGA
SGARITAHLAHELRRRGLKRGIGSACIGGGQGIALLFETV
>ALJ30269.1 putative acetyltransferase ACT22 [*Spodoptera litura*]
MFGLLLTLLGWVGLSPVPFLAGVIGATEPALKLLISILIAYPLAIVYHKYVRQHVEYRNLYF
IATGLDMAYYNFGISMYHNAIPALVIYLTTKLFGPGKVNSVITFAFNMAYLLAGYVVTESE
EYDITWTMPHCVLTLKLIALSFDLWDGKKMLKGQELSANNKLTAIESSPTFLELIGFVYFP
ACFLVGPIFSFRRYKDYITDKFPLDKEKAVYEAQAIKRLVQGLIYLVAYQVGVTVFNIKYM
LSDEFRETSIFYRHFYCGLWAHFALYKYISCWLLTEASCIRFGLSFNGMETKGYPQVSKWD
GCNNIKLLRFEGATRFQHYIDSFNCNTNYFAAEYVVYKRLRFLGNRNLSQLITLAFLALWH
GTQSGYYMTFFNEFIIMVMEKDIEVMLTKTQFYHKMWGNTIFKYLLYIILKTYTIVFMGW
SLAPFDAKSFSKWWSIYASLYFSGFILFLPWTFVYKPLIKSGLKSLEQNESKTQ
>ALJ30270.1 putative acetyltransferase ACT23 [*Spodoptera litura*]
MTYYDYYDGSRIFSFISTRVGLPLDLVNFLIAQVAALCLARLFRKPLRYASPEFRHSVCLVI
GLTMGYFCFGRQAIHLSVLPMLTYTMLKSVSHNIMGNVILAVSMVYLSCLHLHRQIYHTA
DYSLDITGPLMVITQRVTSLAYSLQDSLTVKERPTSANSSEANGRLVKIEKIPSPLEYFAYTL
AFQTLMCGPVVFYSDYIKFIEGARVDEFEKSKHATEPSPRRAVFYKVCGSVAAALLYLTLA
KKYPLAVLEELTDPSSEVSRSWSALYLLWYAYLSTLVVRCKYYHAWLLSEAICNNCGMGF
NGYNNDGTPKWDKMSNIDIFGFEFAQNFRVAIASWNKNTNAWLRDVAYERGGAAWRTA

RVYALSAVWHGFHPGYYLTFFAGGLFTIAARKIRYVARPMFLDSVPKKLFYNFVTFFTTRV
AMTYATVPFVLLHLTPSLAFYGKFYYSLHFIALGAMLIPEKSTRSKATQIQENISCKLSAEA
LPTLESVESLNGKLKIT
>AIN34682.1 fatty alcohol acetyltransferase [*Agrotis segetum*]
MLFLKGSRIITIKMRPTNKLFKAMAAYSSKVTLNEVVIASAVRTPIGSFRGSLASLSASELG
AVAVKAAVERAGIPKEEIKEVYIGNVCSAGMGQAPARQAVIFSGLPKSTICTTVNKVCSSG
MKAIVLAAQGLQTGTHDVILAGGMESMSNVPFYMKRGDIPYGGTQLIDGIVFDGLTDVY
NKFHMGNCAENTAKKFNISRQQQDEYAISSYKRSAAAYESKAFADELVPVPVPQKRGAAP
IMFSEDEEYKKVNFEKFSKLGTVFQKENGTVTAGNASTLNDGASAMVLMTAEAAQRLNV
KPIARVVGYADGECDPIDFPIAPAVAIPKLLAKTGVKKEDVAMWEINEAFSVVALANIKML
ELDPSKLNIHGGGVSLGHPIGMSGNRIVVHLCHALKKGEKGVAAICNGGGGASSIMIEKLE
HTTDGLPVMTFYTKDPCGLCDIVMEELEPYKNRIVIQKVDITQKENVRWLKLYRHDIPVL
FLNGQFLCMHKLDKHLLENRLQKIEDGKLH
>AIN34683.1 fatty alcohol acetyltransferase [*Agrotis segetum*]
MAVAINKGIFIVAAKRTPFGRFGGAFKDVYPSDLLAAAAKDALKAGSIAPEVIDTVNIGQV
YGLSGSSDGGLSPRHAALKSGIPEDKPALGISRLCGSGFQAVVNSAQDIITGVAQTSLAGGT
ENMSTVPFVVRNTRFGVNLGVKMPFEDLLTASSLDTSCNNTMPETAENLAEKYGLHRME
VDQYALQSQQRWKAAQDQGAFKAEMTPVTVKVKRQDKVIEVDEHPRPETTTEMLSKLP
VLFRKGGVVTAGNSSGVNDGAGALVLASEESVKQNGFKPLVRLLGWSVVGVDPSIMGIG
PVPAIQNLLKVTGLKLDDIDMVEINEAFSAQTLACAKELGLDQSKLNINGGAIAMGHPVG
ASGARITAHLAHELRRRGLKRGIGSACIGGGQGIALLLETV
>AIN34684.1 fatty alcohol acetyltransferase [*Agrotis segetum*]
MTGSVSMEMIKLRIEEQHPGTIVYNVNRFESWSSLETMWHQVLEIGMDIANISAKHPDGI
NLIGYSQGGLIARGIVETFPNVSVSTFISLSSPQAGQYGAGFLHLVFPGLVKDTAYELFYSR
VGQHTSVGNYWNDPYHQSLYESYSVFLPYINNHILSAKSADFKNNLLRLKRLVLIGGPDD
NVITPWQSSQFGYYDANETIIEMKGQDIYMEDKIGLRTLDESGRLHIVTVPGVNHFSWHM
NISIVDDCLLPFLD
>AIN34685.1 fatty alcohol acetyltransferase [*Agrotis segetum*]
MDGKTTKMPKVAKVKNKAPAEIQITAEQLLREAKERDLEILPPPPKQKISDPEELRDYQHR
KRKAFEDNIRKNRLVIGNWLKYAQWEESQKQVQRARSIYERALDVDHRNVTLWLKYTE
MEMRNRQVNHARNLWDRAVTILPRVSQFWYKYTYMEEMLENVAGARQVFERWMEWQ
PDEQAWQTYINFELRYKELDRARQIYERFVMVHPDVKNWIKYAKFEENHGFINGARKVF
ERAVEFFGDEELDERLFIAFAKFEENQKEHDRARVIYKYALDHIPKDRNKELYKAYTIHEK
KYGDRSGIEDVIVNKRKYMYEQEVIENPTNYDAWFDYIRLVENEGNVDDIRDTYERAIAN
VPPSKDKQFWRRYIYLWINYALYEELEAEDAERTRQVYRTCLELIPHKIFTFSKIWLMYAQ
FEVRCKDLKQARKTLGMALGICPRDKLYRGYIDLEIQLREFDRCRILYQKFLEYGPENCIT
WIKFAELETLLGDIDRARAIYEIAVGQPRLDMPELLWKSYIDFEVQQGETEKARQLYERLL
ERTVHVKVWLSYAKFELNAENADNINVDLARRVYERANDSLRSAGEKEARVLLLEAWK
DFETEIGEEEKLEKVMAKMPRRVKKRQKIISESGVEEGWEEVFDYIFPEDEMVRPNLKLL
AAAKNWRKQKEVTQPTETENKQDEEEEGQTPPQRMNEVEDD
>AIN34686.1 fatty alcohol acetyltransferase [*Agrotis segetum*]
MPRKVFVVGVGMTNFIKPSTGPDYPELGKEAVLAALADARIKYTDIQQAVCGYVFGDSTC
GQRVLYQVGMTGIPIFNVNNNCSTGSNALYLAKKLIEGGISDVMLAVGFEKMAPGALASS
VFNDRTNPLDRHTIKMADMAELTGAPMTAQYFGNAATEHMKKYGTTEVHLAKIAAKNH

RHGVKNPRAQGKREYTVEEVLASRRIYGPLTKLECCPTSDGAGAAVLMSEEAVIRYGLQA
KAVEIIGMEMATDTPAVFEENSLMKVAGFDMTALAAQRLYQNTGISPKQVDVVELHDCFA
ANELITYEGLQLCGEGEAGKFVDAGDNTYGGRVVVNPSGGLIAKGHPLGATGLAQCAEL
VWQLRGEAGDRQVPRARIGLQHNLGLGGAVVVTMYKKGFSDVAPRAVAAAGNPEDFKV
FKYMKILEDAMENDTDNLIEKVRGIYGFKVKNGPNGAEGYWVINAKEGKGKVTYNGSE
KPDVTFTVSDEDVVDLISGKLNPQKAFFQGKIKIQGNMGLAMKLTDLQRQAAGRIDAIRS
KL
>AIN34687.1 fatty alcohol acetyltransferase [*Agrotis segetum*]
MASQISKSLIKVSHVGSTAKFDTARRALSVGAALHAKRNSLPDRTGKNVVLVDGVRTPFL
VSFTDYAKMMPHELARHSLLGLLQKTGISKDVIDYIVYGTVIQEVKTSNIGREAALAAGFS
DKTPAHTVTMACISSNQAITTGVGMIAAGAYDVIVAGGVEFMSDVPIRHSRKMRSLLLRL
NRAKTPAQRLSLIATIRPDFFAPELPAVAEFSSGETMGHSADRLAAAFGASRQEQDEYSLRS
HKLAAEAQQKGYFTDLIPVKVDGKDGVVDKDNGIRVSTPEQLAKLKPAFVKPHGTVTAA
NASFLTDGASACLVMSEAKAKELGLKPKAYLRDFTYVAQDPVDQLLLGPTYGIPKILDKA
GLKISDIDTWEIHEAFAGQILANLKAMDSDWFAQTYLGRQSKVGTPDLEKWNKWGGSLS
IGHPFAATGVRLAMHTAHRLVREDGQFGVISACAAGGQGVAMILERHPDATCN
>AIN34688.1 fatty alcohol acetyltransferase [*Agrotis segetum*]
MNIRCARPSDLMNMQHCNLLCLPENYQMKYYFYHGLSWPQLSYVAEDEKGHIVGYVLA
KMEEDGEDNRHGHITSLAVKRSHRRLGLAQKLMNQASLAMVECFQAKYVSLHVRKSNR
AALNLYTNSLGFKILEIEPKYYADGEDAYSMMRDLSAFVAESKTELPPIENLEIKSESAIISQ
C
>AIN34689.1 fatty alcohol acetyltransferase [*Agrotis segetum*]
MSAAAKGIFIVGAKRTAFGTFGGAFRNTSATELQTVAAVAALKEAGVAPDKVDSVVVGQV
MTASQTDGIYLPRHVMLKAGIPQDRPALGVNRLCGSGFQSVVNSAQDILTGSAKISLAGG
VENMSQAPFAVRNVRFGTALGQNYAFEDTLWAGLTDSYCGLPMGMTAEKLGAKFGITRD
EVDNFALRSQQRWKAAQDAGAFKAEIAPVTLTVKRKEVKVEVDEHPRPQTTIEGLKKLPP
VFKKEGIVTAGTASGISDGAGAIVLASEEAAKGLKPLARLVGWSYVGVDPSIMGVGPVPAI
ENLLKVTKLTLNDIDLIEINEAFCAQTLSCAKALKLDVEKLNVNGGATALGHPLGASGSRI
TAHLVHELKRRGLKRGIGSACIGGGQGIALMIETV
>AIN34690.1 fatty alcohol acetyltransferase [*Agrotis segetum*]
MLLERRCFNGGNLICGNQGLMVEDQRNRVKLSKIVGATSMFVSGSSDGILTPRHSALKAG
VPYDKPALGVNKLCGSGIQAMVNSAQDILLGSAQISLAGGTENMSAIPFLVRNLRFGTQL
GQVRPFEDFLKAGALDSYCNYTMAQTAENLAKMYDLKREQLDEFALKSQMKWKAGFK
NGAFEAEMAHVTVTVGGKPVVVNKDEHPRTNTTLESLSKLPALFREGGVGTVGNSTGVN
DGAGALILASEEAIKQHNLTPLARLSCWSHAGVEPRVMGLGPVPAVRQLLAATGYTLDDM
DMFEINEQFAAQALASVLEIGLDQDKLNMNGGALAMGHPAAASGARIAAHLTHELRRRG
LKRGIGATCIGGGQG
>AIN34691.1 fatty alcohol acetyltransferase [*Agrotis segetum*]
MKTLFIFLFVIKLISAKPTSIVLWHGMGDTCCVSFSLGGFKLFLEKAIPGVYVDSLQIGNSTI
EDLENGYFLNPNTQVEKVCKYLAEHPKLKDGFNAIGFSQGSQFMRAVVQRCGHTLPTIKN
LISMGGQHQGVYGLPHCGALMHPTCDYIRQLLNYAAYDTWVQHALVQATYWHDPLDEE
TYIHKTIFLPDINNEVFVNKTYIQNLNNLEHFVLVKFDNDTIVQPRETEWFGFYEPGQSKK
MLPMQETRVYKEDRLGLKKMEKEGKLVLISTEGDHLRFSDKWFIENIIKPYLLN
>AIN34692.1 fatty alcohol acetyltransferase [*Agrotis segetum*]

MELQDTYYNKSEYVETASGNKVSRQTVLCGSQNIVLHGKVIVQSDAIIRGDLANVKTGRF
CIISKGSVIRPPFKKFSKGVAFFPLQMGDHVFVGENTVVNAAVVGSYVYIGKNVVIGRRCV
LKDCCMIEDNSVLPAETVVPSFARYSGSPARLITTLPEAMPDLMTEFTKSYYQHFLPTTVQ
>AIN34693.1 fatty alcohol acetyltransferase [*Agrotis segetum*]
MLEHLSYAMEIVTKIFSQISALLGIQWAPMDIPMSRRLQTLAAFVWIYLILFGEALSIYLFIQ
LVYSRFWWMGILYGVWFLNDIEICSRGGRASEWVRNWTWWRYLCDYFPIKLVKTVELDP
SKNYMFACFPHGVISLGAFGSFCTNATGFHKLFPGMTCHLITLGGHFLVPFFRDLALALGI
CSSSEQSLLHLLDNKKYEGNCACMIIGGAAEALDAHPKEYKVILSRRKGFIRVAMKSGAA
LVPVFSFGETDLFRPPNNPENSLLRRFQEKVRQYTGISPMFPMGRGLFQCSYGVLPMRAPV
TTVVGAPMEVKRNLEPTNEEINAVHAEFTERLKTLFETEVKYLQYHEEAKLVIT
>AIN34694.1 fatty alcohol acetyltransferase [*Agrotis segetum*]
MMFGLLLNVLGLIGLSPIPFLSEVIGATEPALKLLISILLGYPLAVIYHKYVKHHKEYRNLYF
VLTGFDMAFYNFGISMYHNAIPAIVIYLSTKFLGPGKNNAIVTFAFNMTYLLAGYVVTESE
DYDITWTMPHCVLTLKLIALSFDLWDGKKMLKGEELSANNKLTALESQPSFLELLGFVYF
PACFLVGPIFSFRRYKDFISDKFPLEREVKVYEAQAVKRLVQGVIYLAAYQIGVTVFSMKY
MLSDEFWDNSVFYRNFYCGLWAHFALYKYISCWLLTEAACIRFGLSYNGSRTENGVSVSQ
WDGCNNIKLLRFEGATRFQHYIDSFNCNTNHFAAEYVYKRLRFLGNRNLSQLITLAFLAL
WHGTQSGYYMTFLNEFLIMVMEKDLESMLLKTEFYHKMWNNSIIKYLLYFILKMYTIVF
MGWSLAPFDVKSFSKWWTVYTSLYFSGFILFVPWSFVYKPLVKKALKASGAHPKAQ
>AIN34695.1 fatty alcohol acetyltransferase [*Agrotis segetum*]
MGKNPVLFLPTHRSYADFCLMTYLCYHFDIDFPAVAAGMDFYSMAVIGRRMRETCAFYIR
RTLAGDPLYAATLKQYVRTVVGKHAAPIEFFLEGTRSRSNKSMPPKYGMLSMTLVPLFAH
EVSDITIVPVNISYDRVMEHSLFAYEHLGVPKPKESTGGFLKALHSLNDHFGNIYINLGSPL
SVREYLKNDTSHSKETLKPLDIQQLTPEQFKKVQSIADYVISLQQKNTVATISNLLSLVLMQ
SLMKDSPLEFEEVVQEVGWMVQELRNLGATVFENDVRSSVERILVVQKKMMRLDKERK
LRLISGVLTDLSVDVKKKMKGHILQPQTMVAAVPIVQLQLYVNPILHYLVPPAIICLIVHRS
AVTRDNLEVDYHRVRKLLSHEFFHLEREEVNTFNKALDYCMQNGVITYSSELYTLGEDTK
LQYLLKWSVLPALTTLLKCAEVMTEQTNCAHKQALKLVQQRVESERVHPYCLSLEATAN
CLSGLVAAHALVKHKGESDVIYDLVPTTMLECSNLVNSILPSFNVDFERNSVVIDHKELSR
L
>AIN34696.1 fatty alcohol acetyltransferase [*Agrotis segetum*]
MTRDEHPQPDVTLEKLSRLQPVSTGGITTAGNITGLNDGAAAMILANGQALRDHNLKPLA
RIVGWSVVGVDPVMMGYAAVPAVETLLKTTGLTIDDMDLVEIHETFAATTVVCARHLGV
DEDKMNVNGGAIAMGHPSGASGARIVSHLTHELRRRGLKRGIASAGIAGGQGIAIIIETV
>AIN34697.1 fatty alcohol acetyltransferase [*Agrotis segetum*]
MTANVILGCVMALLVILFTISSIARYYIKFTLFVVMSLIFATAPVPLMLIKPFDPRNALIPAFF
LRCFAKLLGLRWKVRGLENVDNSRGAVVLLNHQSCLDLYALAIIWPLMSRCTVVSKRSLQ
YLVPFGTATWLWGTVFIDRGAQSARDALNKQVDAIKNQKRKLLLFPEGTRHSGDKLLPLR
KGAFHVAMDAAAPIQPVVISKYHHLDGERQRFGSGEFIVSILPMIETEGMTKEDITGLIEKV
QTSMQEEFTKISMETLARRNLRTKAD
>AIN34698.1 fatty alcohol acetyltransferase [*Agrotis segetum*]
MINLHILKQSTVVHLCFAISYFTSGLILTFIQAILYFGLKPFNKSLYRKINYYLSYSFYSQLVF
MSEWWSNSKLSIYIKKDEYEKFYGKEHGYLIMNHSYEIDWLMGWHFCNTIGVLGNCKA
YAKKSIQYLPPIGWMWKFSEFVFLERSFEKDKETIKYQISELCDYPDPVWLLMTPEGTRYT

KKKHEASLSFAKEKNLPLLKHHLTPRTRGFTTSLQFFRGKIPVIYNIQLAFEKDSKTPPTLTS
LLYGKPVHAHLYIERIPVERVPEDEAEAAKWLHDLFVVKDKMQDSFFNTGDFFLESGVER
REPFSVPPPIWSLVNALGWAVVTLTPMLYYLLGLLFSGKLLYFSIGCGIFGAFFILLQKSIGM
SKISQGSSYGTEKK
>AIN34699.1 fatty alcohol acetyltransferase [*Agrotis segetum*]
MAFAGLKKQINKANQYVTEKMGGAEGTKLDLDFVEMERKTDVTCELVEELQAKTKEFL
QPNPTARAKMAAVKGISKLSGQAKSNTYPQPEGVLGDCMLLYGKKLGEDTVFSNCLIEM
GEALKQMADVKYSLDDNIKQNFLEPLHHLQTKDLKEVMHHRKKLQGRRLDFDCKRRRQ
AKGAHIADDEIRQAEEKFAESLQLAQIGMFNLLDNDVEQVAQLTYFAESLLEYHQQCTEIL
KGLVATLMEKKEEAVNRPKMEFVPKTLADLHIEGIHDLNNGRRYGSTQSLSRPRQHIPPSS
SVGDLSNTDPFTAWEAPPAYRAQARPAQTRPAPGFKPHPAPRNQINGRDPWKASPLPSPVK
SPARTPVAPNKTPCCTALYDFEAENQGELGFKENDVITLINKVDDNWFEGSVHGKTGYFPI
SYVQVTVPLPNM
>AIN34700.1 fatty alcohol acetyltransferase [*Agrotis segetum*]
MSSKRFNTLNVLLVSLSVVAVAYVIRTPWLPIKRETKASLGYPKDSLMNFTELTGKYGYISE
EHHVITDDGYILTMFRIVKATNCHKQKRSPPVLLMHGLLQSSDSWIDSGPNAGLAYLISDA
CYDLWLGNVRGNYYSRGHVHLNPDKDAAYWKFYIEEIGIYDVPAMIDYVLDYTGFEKLN
YIGFSQGTGTFLVMCSERPGYCDKAQLVIALAPAARNLNTKSMIFRTLTQTFAKIEGALSM
YGVQEVFSKGAFSQEFVAFFCQLSDFTERLCETIIDTFDHADFSHMGSITNETTRVLFGHFP
AGTSVHNMARYGQSTRSTTFKKFDYGKEQNLVVYGSEQPPLYNLSATTVPVLCIYGNND
GLVDTKDVEWLMSKLPNVLESVKVKDPLWNHLDVTYSQYTVGSIFPKINEYLLKYTSA
>AIN34701.1 fatty alcohol acetyltransferase [*Agrotis segetum*]
MTENGVRNRQKNGKQSGKQNGVATHQEEKIQDEEFSVRESPLTSLLASSLHLRAIYHIFVV
ILLVLICDTVIFDLVESGKINIGLSIVAAGFGDVSRGIKLWLYDFCVVMSFYPLLVVYSWTG
AISRKYPVLRPTVVLLGVIGIVAVEVAVAAVPVYELGKKHLELGSSVAVTCEMFRFMMKLV
SVASACGPRCVNGNIPLPTFKHYVYFMFAPTLLYRDQYPRTKKIRWGLVVFHFMEVGAIVF
YNCFLWERFIMPYWSDYGKEKTVEAGAVVRGMFACVLPGVISFLCGFYCVLHAWLNAW
SEMLRFGDRLFYEDWWTTSRFSLYYRRWNRVVHSWLRDHIYLPLAPYFGRPLATFAVFFV
SSIAHEVILALSFGFFYPVLLVEFGILGVIMVPLTATAGRRFPNVFNVIMWLGFFIGNGILWS
LYPMEYFARRNCPPSENDSFFVPKSWSCPEVILKPNWSFQNPLSILFTK
>AIN34702.1 fatty alcohol acetyltransferase [*Agrotis segetum*]
MWQSSIIIFAVLLVQVYSAPQFITFKEGKLGVNFGGYHAGVGLGGVAGGSNTAGGLFAEA
GTPFGQGAKAGLGGAVNGNSGTAGGLYAAATAGGNVNAAAGLGGAVAGGKSVGGGFST
AQAGGKSATSVLGGESDVSGSSGFSIEAHKSIGVPTTVVKETKVSIVPVEEVKNVQGEAKF
EATNEIAPSANAGAEGNINAYVNVNAKPEIVKEVSTWKGPYYHTSKIPPFDFQDFMSSLFR
SPQGSYSPPMWAPPPQYNYIQQIHAEPTPVVQTIYLRKHKPHRHHVHKAVYVGGYAGVG
GEVAPPVQQTVVYKTVQPIEKRVDVNVDVNAHGGAGAAVSGEHYGPSSGVTYTKQVAV
NSRPSTFFQDIFNIPISTLKAVSGFLSNTAQNTGISVQKSASFNAGGYSGFSGKAGYSGHSG
YYSY
>AIN34703.1 fatty alcohol acetyltransferase [*Agrotis segetum*]
MAMAHNPYINKVSFSAVFGMPWAVIGRSAILYTDSFLYLSGFLNAHNLLTDLEKKGTINLK
DRLIARWFRLFPLFMSLMLFCTYILPDLNNGPQWNLVVEEHSRVCEKNMWKSFLFIHNYF
GFEDMCLTHTHQIGMDMQLYVATLPLMVLIWKYKTLGWSLLALIAVASTALRYLAIYWY
DISMFVYYGISVQKLLDAARYSYILPTHRATIYLIGVAMAYLMKNKKLKFTLSTTQTRLLW

VFCFALMTATIATPYKWGLEGYKYENFGAALFASLTPILWGVFMCVSHWAIANDYAGIGT
KFIESRLFKFFNKIAYSVYLTQFPIFFYNVGVQRNPDYYSPLLLLYIPELLIVTVISILTTVAIE
MPFNQVYRIYFGQSQKKLKEK
>AIN34704.1 fatty alcohol acetyltransferase [*Agrotis segetum*]
MVFDNNWTGGWFSWTRQSDAMLRNVEKKILSCLKTAYKRFYVDIGSVVGQSDKIWTISL
NDESPKTPLVMLHGMGAGLALWCPNLDSFAATRPVYAIDLLGFGRSSRPKFASDAQKAEA
QWVESVEEWRREVNISQFILLGHSLGGYIATAYAIKYPERVRHLVLADPWGFSERPPNAYE
KAQLPLWVRAIATAVQPLNPLWAVRAAGPAGKWLVSKTRPDISRKYLNFLPDAERVIPEYI
YQCNSQTPSGEAAFHSLMTGFGWAKNPMVRRVDEIDPALPITVLYGSRSWVDNTTGQVL
AEHRGPTNTYVQVINGAGHHVYLDKPELFNKFVLEACARADAHDPRPSLAGASPSAIEAP
PSKLAIEAAPATSATSTESTGKVNISTEAQSS
>AIN34705.1 fatty alcohol acetyltransferase [*Agrotis segetum*]
MGARNLKVLQVISGWQAVELILTCVFVGIWQIIEISVKRLWKGHRRKIEDTSPVELTIDSSI
GTHCYIKVMGVKYHYVETGPRTGQKVLILKDAPDSGNLWGPNWASVVRRLAETDHHVV
TLDLRGTGGSEGGSRSDLAPPRAVEELSALLKALGVSENRQAVVIGFGVGGMLAWYLVHS
RGPLISKFAVINAPHPNLYWQYPPAPFCHRALQFIQWPHFPERWLAEGEMYDREGSWASSR
ACDWTGALNYVRGAAWWKIKPGLRTSAPALLVGHKDSAGQLVASAQYCTASTLRLVTKP
DPSSKELTGVLLDFLIAKEKLLEEQVPRGLMGRVFGAVADRGRELTARLVLPPMQA
>AIN34706.1 fatty alcohol acetyltransferase [*Agrotis segetum*]
MLRRCSKHLQTLYRRQGQTLRFKSSEAPKVFGALSQAAARTTQPRVLTAAHNQVATIHFT
NPLFAEQDVMTPSFPDSVSEGDAKLGDKKVGDAVAVDEVVMEIETDKTALPVMAPGNGII
KEFYVKDGDTVKAGQKLFRLELTEGGPPPKAAAPAPEPPKADAPPPPPPAAAAPPPPPPPA
AAIPTPPPPPPQAPPAAKPAAPISSIPVAAIRHAQSIETATVKVPPTDYSKEIAGTRTEQRVKM
NRMRQRISQRLKEAQNTNAMLTTFNEIDMSHIMAFRKKHLDAFTKKHGVKLGLMSPFVK
AAANALVDQPVVNAVIEDTEIIYRDYVDISVAVATPKGLVVPVVRNVQNMTFADIELTIAGL
AEKAKKGKLTIEEMDGGTFTISNGGVFGSLMGTPIINPPQSAILGMHGIFERPIALNGQVVI
RPMMYIALTYDHRLIDGREAVMFLRKIKEGVEDPATIIAGL
>AIN34707.1 fatty alcohol acetyltransferase [*Agrotis segetum*]
MRATIAKRLSAAKQTIPHYQLTATVNVEKTMAMRKTVNEKLEAEKAGVKVSMNDFIVKA
VAAACKRVPTVNSHWMDSFIRQFANVDVSVAVATPSGLITPILFNCDSRGIIDLSTNMKELA
AKAREGKLQPNEFMGGTVTVSNLGMYGITMFNAIINPPQSLILACGGLQELVIPDKEDPRG
FRSAKFVTFTASADHRVIDGAVGAQWMKAFKENMEDPANMIL
>AIN34708.1 fatty alcohol acetyltransferase [*Agrotis segetum*]
MIGANKLIGKNNIVYQKFIQQRKFTNKNIKDVNYQYLQRSKLPTMHFQKSLPRLPIPELSK
TGDRYLKALRPLLNDNQFEEAEKRTSNFINNEGKVLQEKLISKDKRNKHTSYISDYWFDL
YLRDRAALPINYNPMIVFQNDVRPEYNDQLIRSTNILITAVRFMLSLREQILEPEVYHLNPK
KSDTQLYRTFTRMLPEAISWYGAYLMKVFPLDMSQFVGLFGATRLPRLNKDEIFRDPKSK
HVLVQKQGNFYVFDVLDTDGNLLSPLELLGNLNKIMNDKTPASEHPLGILTTQNRDEWAK
QRDHLEATGNSEVLRKIDSAIFNLILDDDDINDDKRVLLKKYLHSDGTNRWFDKSVSLIVT
RDGVGGVNFEHSWGDGVAVLRFFQDIYAETTKKPFIHPDSKPVDSNISVQKLEFKLDDKS
KHFIDNAKKEYKAWTDSLSIDYILYEGLNKAACKKFKVSPDCIMQLSFQAAHHLLKGNFV
GTYESCSTSAFKHGRTETMRPCTVKTKAFCETLHSNKSSIEELRGKLTECSKLHLELVKDA
AMGQGFDRHMFALMKMAEDNNMPRPEIFDSYEYKFLNKSILSTSTLSSPSVMAGGFGPV
VKEGYGIAYSAFPDKLGAAVASYKAHNNSTQYVEALHKSFLDITKILSG

>AIN34709.1 fatty alcohol acetyltransferase [*Agrotis segetum*]
MSSRTPLTKAEKVAYALRHEKSYSWHKWFSVLAVLTIIVSLLTYYLFGMWTEPPPLPKLDL
EQHWGPYPIDMKPDNSIRPFTIEFSDVIVNDLRERLLHRRSFTPPLENAGFTYGFNTHFLTQ
VLDFWQNKYNFKEREQFLNKYEHFVTNIQGLDIHYMHVKPKVPGNVTVVPLLLIHGWPG
SIREFYEIIPKLTTPRPNQEFVFEVIAPSIPGFGFSQAPVRAGMGPIQVSVIFRNLMQRIGHDE
YYVQGGDYGSAIGSVMATLFPENILGYHTNMPMVAVNTWVSIYTVLGSLWPNFIVEPSVQ
DRMYPLSKHIGKVIEETGYFHIQATKPDTVGIALSDSPAGLAAYILEKFSTWTNMENKKAS
DGALLQKFSLTHLLDNVMIYWASNTITSSMRHYVEGYKQLMFTDRIPTEVPTWGIKFKHE
ISFQPDSILKLKYKNYLHSSVVEDGGHFAAMELPDVLADDIFDAVHMFRTFHRKKRNNKA
SDKPITKESTKPDAETVNKVEKESKVNFETVNKVEKEPKVNFETAKTVYEFTVKDIQGQE
VKLERYKGKVLIIVNVASHCGYTNSHYTELNELYEKYSKKGLRILAFPCNQFGGQEPGTLK
EILQFTKEKKVKFDLFEKIEVNGENAHPLWKFLKRIQGGTLGDFIKWNFSKFIIDRNGVPV
ERFGPNTSPLELEPYLEKLLG
>AIN34710.1 fatty alcohol acetyltransferase [*Agrotis segetum*]
MSFLMRKCIVNLKNVNRCSSVCVLMQTGKHRSQYSTSNILKRSIVPSDVHLRQRKFHTSQ
IVNKIVAFKLSDIGEGIREVVIKEWFVKVGDKVQQFDNICEVQSDKAAVTITSRYDGVVTK
LYHEVDQTALVGQPLVDIEVQGGADEGTSSAPESIPAAAAKQESVADKSQKVKILTTPSVR
RIAAQFKVDLSSVKATGRNGRVLKEDMLAHLNISSDKSNEIHEPSSISAMAIPLVPAQAKM
EVMLEDRVVPVSGFTKAMVKSMTEAMKIPHFGYSDEYDVTKLVESRESLKKLAEAKGVK
LTYMPIIIKATSLGLEQIPVLNSSLDSTCEHLTYKASHNIGVAMDTPNGLIVPVIKNVQAKTI
LEVARELNTLQEKGSKGQLGLSELTGGTFTLSNIGIVGGTYTKPVILPPQVAIGALGKIQAL
PRFDVEGNLRKAHILTVSFSADHRVIDGVTMARFSNLLKNYLENPYSLLLDL
>AIN34711.1 fatty alcohol acetyltransferase [*Agrotis segetum*]
MDLTEHEWYIQAPWGRIAIIAWGDCYDPPVLLVHGSMDSAVSFRPLVSKLPKNFYYIGMD
LPGNGKSDRFLPGLMISVYDMVYSVHAVVKHFRWKTYTLIGHSFGAYLGQFYNLCYPGR
LDKLVNLDPINFFAVPPEEFGRWYHVFFTDYYKNYDKFNTQPENAPKIKWTEALQSIKSSR
PSLTEEQAAAVLERLSMPAGDGYVKYTYDLRMKRVNGPAYSPEHIKQLFTTTKTPILTIAC
QKSLKRKLFRNTDFLLDEAEFPGRNLRFRTVDGTHDVHVSHPERVAAYVGQFLVYGLDGL
DNKAKL
>AIN34712.1 fatty alcohol acetyltransferase [*Agrotis segetum*]
MALVMPFVSVAISILYTPLLLLILCIIFLASIGKSLGVRRLYVNILLKLFEYGRQHIEVAKIKIQ
RTDSSDDEEVPPAPDDDKPPSATIKENGVNGTLNTVIERQEILGPSPELNYKRSTSQERVQN
GHSKPSQGNGENNIEFHLSNCLDLVKAGMESIIEDQVTSVFEAEELRSWNLLTRTNRQYEF
LTWRLTIIWAMGFVVRYMFLLPLRIMIFVIGVWWLVACTACVGTLPDGKTKQRVNYAVSL
MCFNFLSRCISAVITYHDTHYKPRNGICVANHTSPIDALVLMCDNCYSLIGQRHNGFLGILQ
RALARASPHIWFERSEVKDRHAVARRLKEHISVPDNPPILIFPEGTCINNTSVMQFKKGSFE
VGGTIYPVAIKYDPRFGDAFWNSSRYGMLHYLLNMMTSWAIVCDVWYLPAMTRAHDES
AVDFANRVKAVIARRGGLVDLMWDGQLKRMKPKKEWRELQQEEISKRLKGE
>AIN34713.1 fatty alcohol acetyltransferase [*Agrotis segetum*]
MAKRLLSSLILNSSGTNLKSSLPVLSKKLHTQQVPTKEIQIPVKFGHLAGKLWGSGDQPPIL
ALHGWQDNAGTWDPLIPMIIKDRPILALDFPGHGFSSWIPPGMLYYQWELPRIILYLKEYF
KMEKVALLAHSMGAIAGMRFATVFPDDVEFYIAIDSLIYDDYDLDAVVDRISKTIRKGLLA
QSRLDKEPPLYTLEDMIKIWHAGTRKSVALESVPHLLKRGANQSKTDPSKYYFSRDSRLK
YSLFNPEDKKFVEALVRRLKCPTLYVKAIDSPYSADAYSIEMREILEQVNEKYEFHFVRGT

HHVHLNNPELVAPLIKNFIQKHNLTI
>AIN34714.1 fatty alcohol acetyltransferase [*Agrotis segetum*]
MFNIWEVVNKMAAIAKKELFYIWPFGLSAYLAGVVFIDRSNPKNAYKQLQQTSDVMVKS
KTKIWLFPEGTRNKDYTRIKPFKKGAFNIAVAAQVPIIPVVFSPYYFINKEKYIFNKGHIIIQC
LEPVPTKGLTMDDVPELINKVHQQMSATYKELSKEVVNALPADYPFTLLG
>AIN34715.1 fatty alcohol acetyltransferase [*Agrotis segetum*]
MGNVILAVSMIYLSCHHLHRQIYHTADYSLDITGPLMVITQRVTSLAYTLQDSLAVKEIGSN
GVTETARDPELAKIEKIPSPLEYFAFTLAFQTLMCGPVVFYSDYIQFIEGARVDACEKGPSV
AKEEPSPRNAVIFKVAGSVAAAVLYLSLAKKYPMTALEELTDPASEVSRTWSALYLLWYAY
LATLVVRCKYYHAWLLSEAICNNCGMGFNGYNNDGTAKWDKLSNIDIFGFEFAQNFRTAI
SSWNKNTNAWLRHVAYQRGGAAWRTARVYALSAVWHGFYPGYYMTFFAGGIFTVAARK
IRFLARPVFLDSAPKKLFYDSLSFITTRVAMTYTTVPFVLLHLTPSLAFYGKFYYSLHFIALG
AMLLPEKSKQPTENYQQSSKETTETSNGKLKIT
>ATJ44623.1 acetyltransferase 30 [*Helicoverpa armigera*]
MENLSSLPLLVRDMRFGNPLGKYFKVDDFLHMGFFDSYCNLFLVQTADIVAAKFGVTREE
ADEFALRS
>ATJ44622.1 acetyltransferase 29 [*Helicoverpa armigera*]
MATSTKAIFIIGAKRTPFCSYGGPLRELPAYQVFATAAKEAIRSANLEPSLIDNTVVGNVNFL
SQCDGGKTPRYCGIYSGVPISSPALGVSKACGTGLQAIINSAVDTITGNSKVTLAGGTDLM
SSMPMLVRNVRFGTALGTPYRFEDHIQRQIPDGYTGLTMQKMVEDLANKYGVTRKDVDE
FALQSHLKWKAAEESKAFEQELVSLEVTLKKKQVLVDKDQTPQSLKSEDLSTLPVLIENG
NILTPGNTSAPADGAAALLLAHEEAVKGHSLQPLARVAGWTCVGVNPEDAGLGGVLAIRN
LLDSQKLTVGDVDLFEINENFASQAVMATRELKIDQSKVNVSGGALAIGDPMSATGARMA
THLVHELRRRNLKRGIAASSCGGGQGVAILLEKM
>ATJ44621.1 acetyltransferase 28 [*Helicoverpa armigera*]
MASSYSYPELFLAGFIFTLPFLYEKSNVFRYYLKFFLYYAYVLITCTVLLPVVLIYPRDVTNL
IVASRFCRYASYIVGIEWELRGMEHWNSEQCYIVISNHQSSLDILGMFEMWPQMKRCTVV
AKRPLMFTGAFGFGAWLSGLVFIDRLRTERARQLMKDATARVIKEKTKLWIFPEGARFNK
GSIQNFKKGAFYLAIDAQIPIMPVVFSQYYFLDSDTKTFEPGKVIITTLPPIPTSGMTRNDVE
TLSEMARQQMIEVFHESSKDLVMQKKIAI
>ATJ44620.1 acetyltransferase 27 [*Helicoverpa armigera*]
MGARNLKVLQVISGWQAVELILKCVFVGIWQIIEISVKGLWKGARRKVKDTPPVELTIDSS
VGTHCYIKVMGVKYHYVETGPRTGQKVLILKDAPDSGNLWGPNWANAVRRLAETDHHV
MTLDLRGTGGSEGGSRSELSPPRAVEELSALLKALGVTENRPAVVIGFGVGGMLAWYLVH
SRGPLISKFAVINAPHPNLYWQFPPAAFCHRVLHFIQWPHFPERWFAEGELNDCDGRWASS
RACDWTGALNYVRGAAWWQVRPGLRTSAPALLVGDKDSAGQLVASAQHCTTSTLRLVT
KPEPNSKELAVVLLDFLISKEKLIEEVPRGLMGRMFGAVADRGRELTARLVLPPTQA
>ATJ44619.1 acetyltransferase 26 [*Helicoverpa armigera*]
MWDKIIYMIVIIVVTYILKQLFSETPNFVKFKSKFLVFYIWTSVTAVILLPFFVFNPKNVKNS
LFGSQIVKHVTKVIEVKWLLRNGKVLAEDRGAVVVSNHQSSIDILGMFNIWHVADKVAAI
ARKEIFYVWPFGLAAYLAGVVFIDRNNSKDAYKQLKITSEVMIKNKTKIWLFP
>ATJ44618.1 acetyltransferase 25 [*Helicoverpa armigera*]
MSEQVEFVDILEPRRTQSGIFSFMTRNWHPQRTLKLDKYFTPQELKDIAANSVYLDAFIEA
ECSRSGQSKDKLHQEVHNYLEEMGLDKKMHVIRWMGVIFLKISFMMKIKMFVNEAAAF

NLKSVMGNNPVLFLPTHRSYADFCLMTYLCYHFDIDFPAVAAGMDFYSMAVIGRRMRET
CAFYIRRTLAGDPLYAATLKQYVRTVVGKHAAPIEFFLEGTRSRSNKSMPPKYGMLSMTL
VPYFAHEVTDITVVPVNISYDRLMEHSLFAYEHLGVPKPKESTGGFLKALHTLNDHFGNIY
INLGAPLSIREFLKNDTSHSQETLKPLDMQQLTPDQFKQVQSIADYVITLQQKNTVATISNL
LSLVLMQSLMKNVPLEFEEVLQEVGWMVQELRNLGATVFENDVRSSVERILVVHRKMM
RLDKERRLRLISGVLVDLSSDVKKKMKGHILQAQTMVAAVPVIQLQLYVNPILHYLVPPAII
CLIVHRSATARDRLEADYHRVRKLLSHEFFHLEKEEPNTFAKALEYCIQNSVISYNGELYAL
GEDTKLQYLLKWSVWPALTSLLKCAQVMTEQSICAHKQALKLVQQRVESERVHPYCLSL
EATANCLNGLVAANALVRNKGEGDIIYELVPHTMQECHNLVSSILPTFSVDFTNNAVVVDH
KALSRL

>ATJ44617.1 acetyltransferase 24 [*Helicoverpa armigera*]

MKILFILLCVIKLISGTPTPIVLWHGMGDTCCLSFSLGGIKVFLEKNIPGVYVNSLKVGNSSI
EDLENGYFMNPNQQVEYVCGLLAADPQLKDGFNAIGFSQGSQFLRAVVQRCGHILPKIKN
LISLGGQHQGVYGLPHCGALMHPTCDYIRQLLNYAAYENWVQNALVQATYWHDPLDDE
TYIHKSIFLSDINNEIMANKTYIQNLNNLDHLVLVKFDNDTIVQPRETEWFGYYEPGQSKK
LLPLRETKIYTEDRLGLKKMDKEGKLILLSTVGDHLRFSDTWFIDNILKPYLLN

>ATJ44616.1 acetyltransferase 23 [*Helicoverpa armigera*]

MTYYDYYDGSRIFLFISNKVGLPLDLVNFLIAQVAALCLARLFRKPLRYASPEFRHSVCLVI
GLTMGYFCFGRQAIHLSVLPMLTYTLLKSVKHQIMGNVILAVSMIYLSCLHLHRQIYHTAD
YTLDITGPLMVITQRVTSLAYSLQDSLTVKDLKSKATALQTTGGEDLVKIEKIPSPLEYFAFT
LAFQTLMCGPVVFYTDYIKFIEGARVDELEKSADTKEPSPRTAVFYKVAGSLAAALLYLTL
AKKYPLTVLEELTDPTSEVSRSWSALYLLWYAYLSTLVVRCKYYHAWLLSEAICNNCGMG
FNGYNNDGSPKWDKMSNIDIFGFEFAQNFRIAIASWNKNTNAWLRDVAYSRGGAAWRTA
RVYALSAVWHGFHPGYYLTFFAGGIFTVAARKIRFVARPMFLDSVPKKLFYDSVSFITTRVA
MTYATVPFVLLHLAPSLAFYAKFYYSLHFIALGAMLIPEKAKRPKPAVVQEQQSKSPTLSK
EAMEESLEELNGKLKIS

>ATJ44615.1 acetyltransferase 22 [*Helicoverpa armigera*]

MELQDTYYNKSEYVETASGNKVSRQTVLCGSQNIVLHGKVIVQSDAIIRGDLANVKTGRF
CIISKGSVIRPPFKKFSKGVAFFPLQMGDHVFVGENTVVNAAVVGSYVYIGKNVVIGRRCV
LKDCCMIEDNSVLPAETVVPSFARYSGSPARLITTLPEAMPDLMTEFTKSYYQHFLPTTVQ

>ATJ44614.1 acetyltransferase 21 [*Helicoverpa armigera*]

MSFLMRKCVVNLKNVNRYSAVCVLMQSERHRSRFSSSNVLKRNRLLSDVHLQHKKIHTS
QIVNKTVAFKLSDIGEGIREVVIKEWFVKVGDKVQQFDNICEVQSDKAAVTITSRYDGVVT
KLYHDVDTTALVGQPLVDIEVQGEADEGSSSSSPEEQPKVTKQESVEEKSQRIKVLTTPAVR
RIAAQFNVDLSTVKATGRNGRVLKEDMLAHLNIDSDGSNEVPAPSSVQAMSIPLTQAKAK
VEVLLEDKVVPVTGFTKAMVKSMTEAMKIPHFGYSDEYDVTKLVESREALKKIAEARGA
KLTYMPIIIKATSLSLEQLPVLNSSLDSTCEHLTYKASHNIGVAMDTPNGLIVPVIKNVQNK
TILEIARELNTLQERGSKGQLGLNELSGGTFTLSNIGIVGGTYTKPVILPPQVAIGALGKIQV
LPRFDAEGNVRKAHILTVSFSADHRVVDGVTMARFSNFLKNYLENPYTLLLDL

>ATJ44613.1 acetyltransferase 20 [*Helicoverpa armigera*]

MMEHISNIFEVLTKTFSQISDLLGIQWAPMNIPMSRRLQTLAAFVWIYLILFGEALAIYLFIQ
LVYSRFWWAAILYGVWMLNDIDICHRGGRVSQWVRNWTWWRYLCDYFPINLVKTVDLD
PSKNYMFAIFPHGVISLGAFGSFCTNATNFHKLFPGMSCHLITLGGHFLVPFFRDLALAIGM
CASSEQSLLHLLDQKKYEGNAVCMIIGGAAEALDAHPKEYKVILSRRKGFIRVAMKSGAS

LVPVFSFGETDLFHPPNNPENSLLRRFQEKVRQWTGISPMFPMGRGLFQYSYGVLPIRSPV
TTVVGAPMEVKRNLEPTNEEIDAVHAEFTKRLQTLFETEKVKYLKYHEEAKLVIT
>ATJ44612.1 acetyltransferase 19 [*Helicoverpa armigera*]
MAVVINKGIFIVAAKRTPFGRFGGAFKEVYPSDLLAAAAKDALKSGSVAPEIIDTVNIGQV
YGISGSSDGGLSPRHAALKSGIPQEKPALGISRLCGSGFQAVVNSAQDIITGAANISLAGGT
ENMSTVPFVVRNTRFGVGLGAKMPFEDVLTTSSLDTSCNFTMPETAENLAEKYGLQRME
VDQFALQSQQRWKAAHDQGVFKAEMTPVTVKVKRQEKVVEVDEHPRPDTTTEMLSRLP
VLFRKGGVVTAGNSSGVNDGAGALILATEESVKQHGLKPLVRLLAWSVVGVDPSVMGIG
PVPAIQNLLSATGLKLDDIDLVEINEAFAAQTLACAKELGLDQSKLNVNGGAIAMGHPVG
ASGARITAHLAHELRRRGLKRGIGSACIGGGQGIALLLETV
>ATJ44611.1 acetyltransferase 18 [*Helicoverpa armigera*]
MFGVALCLLDVIGLNPIPFIAGIIGATVPAVNLLISILLGYPLAIIYHKYVRKTVELRNLYFIVT
GMDLAYFNFGTSMYHNAIPAIVIYLSTACLGPGKMNVIFTFAFNMIYLLAGYVMTESEDY
DITWTMPHCVLTLKLIALSFDIWDGDKFLKGEELSKNNLQTALPTQPTFLELIGFVYFPACF
LVGPIFSFRRYKDYITDVFPLDTAPEVYEELALKRLFQGVCYLAAYQIGVSVFSMKSMLSD
EFRDTSIFYRHFYCGLWAHFALYKYISCWLLTEAACIRFGLSFNGFVGPVKTLTKWDGCNN
IKLMRFESATRFQHYIDSFNCNTNHFASEYVYKRLRFLGNRNLSQLITLAFLALWHGTQSG
YYMTFLNEFLIMVMEKDIEAILTKTAFYDKMWSTPYVKYPLYVILKTYTIVFMGWSLAPF
DVKSFGKWWSIYSSLYFSGFILFLPWSFVYKPMIIKLLKRNAASHSKAS
>ATJ44610.1 acetyltransferase 17 [*Helicoverpa armigera*]
MASQISKSLLKVSHVSSTAKFDTARRALSVGAALQQKKKSLPDRTGKNVVLVDGVRTPFL
VSFTDYAKMMPHELARHSLLGLLQKTGISKDLIDYIVYGTVIQEVKTSNIGREAALAAGFS
DKTPAHTVTMACISSNQAITTGVGMIAAGAYDIIVAGGVEFMSDVPIRHSRKMRSLLLRLN
RAKTPAQRLSLIASIRPDFFAPELPAVAEFSSGETMGHSADRLAAAFGASRQEQDDYALRSH
KLAHEAQQKGYFTDLMPVKVDGKDGVVDKDNGIRVSTPEQLAKLKPAFIKPHGTVTAAN
ASFLTDGASACLVMSEAKAKELGLKPKAYLRDFTYVAQDPVDQLLLGPAYGIPKILDKAG
LKMSDVDTWEIHEAFAGQILANLKAMDSDWFAQTYLGRQTKVGAPDLDKWNKWGGSL
SIGHPFAATGVRLAMHTAHRLVREDGQFGVISACAAGGQGVAMILERHPDATCN
>ATJ44609.1 acetyltransferase 16 [*Helicoverpa armigera*]
MWYKIFIFTIVCVLTYILKKLHDTGPNRVKFYFNFFLFYFLSSMLAAVIWPYFLLSPRNVRN
AKIAVRLLKHITKLYDLKWHLRDGKILAEDRGAVIISNHQSSLDILGMFNIWEVVDKLAAI
AKKELFYVWPFGLSAYLAGVVYIDRRNAKGAYKQLKVTSEVMVKNKTKIWLFPEGTRN
KDYTKLQPFKKGAFNIAVAAQVPIIPVVFSPYYFINKEKYIFNKGHVIIQCLEPVPTVGLTME
DVPDLIDRVHHKMSVAYQEISKEVFSSLPSDYPVTLKG
>ATJ44608.1 acetyltransferase 15 [*Helicoverpa armigera*]
MAKRLLSRLILNSNGTTLKSPLPILSKKLHTQIPVKEIQIPVKFGHMSGKLWGSGDKQPILA
LHGWQDNAGTWDPLIPMIIKDRPILALDFPGHGLSSWIPPGMLYYQWELPRVILYLKEYFK
MEKVSLMSHSMGAIASMRFATVFPDDVEFFIAIDSLIYDDYDLNSVVNRISKTLRKGLIAQ
TRLDQEPPAYTMEEMIKIWHLGTRKSVSMESVPHLLKRGAKQTKSDPSKYYFSRDSRLKY
TLFNPEDRKFVEALVKRLKCPTLYVKAIDSPYSADPYSIEMREILEQINDKYEFHFVPGTHH
VHLNNPELVAPLIKNFVQKHNLSI
>ATJ44607.1 acetyltransferase 14 [*Helicoverpa armigera*]
MFASEVLRSVDNMASKRSNTFKVLLASLAVAALGYVLRSPSLYLRRETKSSLGYPKDSLL
NFTELTAEYGYLSEEHKVLTDDGYILTMFRIVKARNCHRAKRSPPVLLMHGLLQSSDSWID

SGPDAGLAYLISDACYDLWLGNVRGNYYSREHVRLDPDKDPAYWKFYIEEIGIYDVPAMI
DYVLNYTGFEKLNYIGFSQGTGTFLVMCSEKPGYCDKVKLVISLAPASRQMHTQSKIFRT
MTQTFYRMEGLLSMTGLQEVFSKG
>ATJ44606.1 acetyltransferase 13 [*Helicoverpa armigera*]
MAFAGLKKQINKANQYVTEKMGGAEGTKLDLDFVEMERKTDVTCELVEELQTKTKEFL
QPNPTARAKMAAVKGISKLSGQAKSNTYPQPEGVLGDCMLLYGKKLGEDTVFSNCLIEM
GEALKQMADVKYSLDDNIKQNFLEPLHHLQTKDLKEVMHHRKKLQGRRLDFDCKRRRQ
AKGAHIADDEIRQAEEKFAESLQLAQIGMFNLLDNDVEQVAQLTYFAESLLEYHQQCTEIL
KGLVSTLMEKKEEAVNRPKMEFVPKTLADLHIEGIHDLNNGRRYGSTQSLSRPRQHIPPSS
SVGDLSTTDPFKAWEAPSPVRAQVRPAPGFKPHPAPRNQFNGRDPWKASPLPSPVKSPART
PVVANKTPCCTALYDFEPENQGELGFKENDVITLINKVDDNWFEGSVNGKTGYFPISYVQ
VTVPLPNM
>ATJ44605.1 acetyltransferase 12 [*Helicoverpa armigera*]
MALKIVFLGLVLFITPILCYKPVVLIHGVMTGSASMEMIKFRIEEQHPGTIVYNVNRFESWS
SLETMWHQVLEIGMDIANISASHPEGINLIGYSQGGLIARGIVETFPNVSVSTFISLSSPQAG
QYGAGFLHLVFPGLVKDTVYELFYSRVGQHTSVGNYWNDPYHQSLYETYSVYLPYINNHI
KSAKSEDFKKNLLRLKRLVLIGGPDDNVITPWQSSQFGYYDANETIIEMKSQDIYMEDKIG
LRTLDESGRLHVVTVPGVNHFSWHMNISIVDDYLLPYLD
>ATJ44604.1 acetyltransferase 11 [*Helicoverpa armigera*]
MIRANTIIFKNNSVCQKFNQQRNFNKNVKDVEYQYLQRSKLPTMHFQKSLPRLPIPDLSK
TGERYLKALRPLLNDNQYEEAQQRTGNFIAKEGKILQEKLIAKDKRNKHTSYISEYWFDL
YLRDRVPLPINYNPMIVFQNDVRPEYNDQLIRSANILISSVRFMLSLREQILEPEVYHMNPK
KSDTPLFRNFTRMLPEAISWYGAYLMKVFPLDMSQFVGLFGATRLPRQTKDEIFRDPKSK
HVVVQKQGNFYVFDVLDANGNLLSPQEILGNLAQIMNDNTTAAEHPLGILTTQNRDVWA
KQRSHLESTGNSEVLNKIDSAIFNLILDDDTINDDKRVLLKKYLHSDGLNRWFDKSFSLIVT
RDGVAGVNFEHSWGDGVAVLRFFQDIYAETTKKPFIHPDSKPADSNISVQKLEFKLDDKSK
QFIDNAKKEYKAWCDSLSIDYILYEGLNKAACKKFKVSPDCIMQLSFQAAHHLLKGNFVG
TYESCSTSAFKHGRTETMRPCTDKTKAFCETLHSNNTSIDELRAKLTECSKLHLELVKDAA
MGQGFDRHMFALMKMAEDNNMPRPEIFDSYEYKFLNKSILSTSTLSSPSVMAGGFGPVV
KEGFGIAYSAFPDKLGAAVASYKPHNDSSQYIEALHKSFLDITKILSG
>ATJ44603.1 acetyltransferase 10 [*Helicoverpa armigera*]
MDLVEHEWYIQAPWGRIAIIAWGDCCNPPVLLCHGSMDSAVSFRPLVSKLPRNYYYIGLD
LPGNGKSDRFLPGLMISVYDMLYAIHALVKHFRWKTFTLIGHSFGAYLGQFYNLCYPDKL
ELLINLDPINFFAVPPEEFSRWYHIFFTNFYKNYDKYNTPKEKSPTIKWTEALQSLMRNRPS
LNEEQAAAVLERLSEPVGDGCVRYTYDLRMKRINGPAYSPEHVKKLFTAVRTPILTIACQK
SLKNKLFRNTAFLLDEAEYPGGNFRFKSVEGSHDVHISHPERVAGFIGQFLEYGVEGLDKK
SKL
>ATJ44602.1 acetyltransferase 9 [*Helicoverpa armigera*]
MALIMPFVSVAISILYTPLLLLILCIIFLASIGKSLGVRRLYVNILLKLFEYGRQHIEVAKIKIQ
RSDSSDEEELPPIPDDTPPSAIVKENGANGTKMTVIERHEILGPSPELNYKRSTSQERVQNG
HKTPQGNGENNMEFHLSNCLDLVKAGMESIIEDQVTSVFEAEELRSWNLLTRTNRQYEFL
TWRLTIIWAMGFVVRYMFLLPLRIMIFVIGVWWLVACTACIGTLPDGRTKQRVNYAISVMC
FNFLSRCISAVITYHDTDYKPKNGICVANHTSPIDALVLMCDNCYSLIGQRHNGFLGILQRA
LARASPHIWFERSEVKDRHAVARRLKEHISVADNPPILIFPEGTCINNTSVMQFKKGSFEVG

GTIYPVAIKYDPRFGDAFWNSSRYGMLHYLLNMMTSWAIVCDVWYLPAMTRAADESAV
DFANRVKAVIARRGGLVDLMWDGQLKRMKPKKEWRELQQEEISKRLKGE
>ATJ44601.1 acetyltransferase 8 [*Helicoverpa armigera*]
MNIRCARPSDLMNMQHCNLLCLPENYQMKYYFYHGLSWPQLSYVAEDEKGHIVGYVLA
KMEEDGEDNRHGHITSLAVKRSHRRLGLAQKLMNQASLAMVECFQAKYVSLHVRKSNR
AALNLYTNSLGFKILEIEPKYYADGEDAYSMMRDLSAFAAESKDTQPTENLEIKSESAIISQ
C
>ATJ44600.1 acetyltransferase 7 [*Helicoverpa armigera*]
MLRRCSKHLQTLYRRQGQTLRFKSTEAPKVFGALSQAAARTTQPRVLSAAQNQIATIHFT
NQLFAEQDVMTPSFPDSVSEGDAKIEKKVGDAVAMDEVVMEIETDKTALPVMAPGNGIIK
EFYVKNGDTVKAGQKLFRLELTEGAPPPKAAAPPAPEPPKAEAPPPPPPAAAAPPPPPPPAA
AAPPPPPPPPAPKAEAPRAAPISSIPVAAIRHAQSIETATVKVPPTDYSKEMAGTRTEQRVKM
NRMRQRIAQRLKEAQNTNAMLTTFNEIDMSHIMAFRKKHLDAFTKKHGVKLGLMSPFVK
AAANALMDQPVVNAVIEDQEIIYRDYVDISVAVATPKGLVVPVVRNVQNMTYADIELTIAG
LAEKAKGGKLTIEEMDGGTFTISNGGVFGSLMGTPIINPPQSAILGMHGIFERPIALNGQVV
IRPMMYIALTYDHRLIDGREAVMFLRKIKEGVEDPATIIAGL
>ATJ44599.1 acetyltransferase 6 [*Helicoverpa armigera*]
MNHSYEIDWLMGWHFCNTIGVLGNCKAYAKKSIQYLPPIGWMWKFSEFVFLERSYEKDK
ETIKHQISELCDYPDPVWLLMTPEGTRYTKKKHEASLSFAKEKNLPLLKHHLTPRTRGFTT
SLQFFRGKIPVIYNIQLAFEKDSKTPPTLTSLLYGKPVHAHLYIERIPVENIPVDEAEAAKWL
HDLFVVKDKMQDSFFNTGDFFTESGVERTEPFTVPPPIWSLVNALGWAVVTLTPMLYYLL
GLLFSGKLLYFSIACAIFGAFFILLQKSIGMSKISQGSSYGTEKK
>ATJ44598.1 acetyltransferase 5 [*Helicoverpa armigera*]
MAAYSSKLSLNDVVIASAVRTPIGSFKGSLSSLSATELGGVAVKAAVERAGIPKEEVKEVY
MGNVCSAALGQAPARQASIFGGLPKSTICTTVNKVCSSGMKAIVLATQGLQTGTQDVILA
GGMESMSNVPYYLKRGDTPYGGVQLNDGILYDGLTDVYNKIHMGNCAENTAKKLNISR
KEQDDYAISSYKRSAAAYENKTFADELVPVPVPQKRGAAPVIFAEDEEYKKINFDKFTSLA
TVFQKENGTVTAGNASTLNDGAAAMVLMTADAAQRLNVKPLARVIGYADGECDPIDFPI
APAVAIPKLLAKTGVKKEDVAMWEINEAFSVVALSNMKMLELDSNKINIHGGAVSIGHPIG
MSGARIVVHLCHALKKGEKGVAAVCNGGGGATSIMIEKL
>ATJ44597.1 acetyltransferase 4 [*Helicoverpa armigera*]
MDGKTTKMPKVAKVKNKAPAEIQITAEQLLREAKERDLEILPPPPKQKISDPEELRDYQHR
KRKAFEDNIRKNRLVIGNWLKYAQWEESQKQVQRARSIYERALDVDHRNVTLWLKYTE
MEMRNRQVNHARNLWDRAVTILPRVSQFWYKYTYMEEMLENVAGARQVFERWMEWQ
PDEQAWQTYINFELRYKELDRARQIYERFVMVHPDVKNWIKYAKFEENHGFINGSRKVFE
RAVEFFGDEDLDERLFIAFAKFEENQKEHDRARVIYKYALDHIPKDRNKELYKAYTIHEKK
YGDRSGIEDVIVNKRKYMYEQEVIENPTNYDAWFDYIRLVENEGNVDDIRDTYERAIANV
PPSKDKQFWRRYIYLWINYALYEELEAEDAERTRQVYRTCLELLPHKIFTFSKIWLMYAQF
EVRCKDLKQARKTLGMALGICPRDKLYRGYIDLEIQLREFDRCRILYQKFLEYGPENCITW
IKFAELETLLGDIDRARAIYEIAVGQPRLDMPELLWKSYIDFEVQQGETEKARQLYERLLER
TVHVKVWLSYAKFELNAENADNFNVELARRVYERANDSLRSAGEKEARVLLLEAWKDF
ETEIGDEEKLEKVMAKMPRRVKKRQKIISESGVEEGWEEVFDYIFPEDEMVRPNLKLLAA
AKQWRKQKEVAQPAEPETQNEDQDDEGHTPPQRNDDEIENENN
>ATJ44596.1 acetyltransferase 3 [*Helicoverpa armigera*]

MSVAAKGIFIVGAKRTAFGTFGGVFRNTSATELQTIAATAAIKEAGIAPEKVDTVVVGQVM
TASQTDGIFIPRHVALKSGIPQDRPALGVNRLCGSGFQSVVNSAQDILTGAAKISLAGGVEN
MSQAPFAVRNVRFGTALGSTYAFEDTLWAGLTDSYCGLPMGMTAEKLGAQFKITRDEAD
NFALRSQQRWKAAQDAGVFKNEITPVTLTVKRKEVKVEVDEHPRPQTTIEGLKKLPPVFK
KEGLVTAGTASGISDGAGAIVLASEEAAKGLKPLARLVGWSYVGVDPSIMGVGPVPAIEN
LLKVTKMTLNDIDLIEINEAFCAQTLSCAKALKLDMEKLNVNGGATALGHPLGASGSRIT
AHLVHELKRRGLKRGIGSACIGGGQGIALMVEAV

>ATJ44595.1 acetyltransferase 2 [*Helicoverpa armigera*]

MTALAAKRLYQNTGISPKQVDVVELHDCFAANELITYEGLQLCGEGEAGKFVDAGDNTY
GGRVVVNPSGGLIAKGHPLGATGLAQCAELVWQLRGEAGERQVPRAKIALQHNLGLGGA
VVVTMYRKGFADITPRPVAASGNPEDFKVFKYMKILEEAMENDTENLIEKVRGIYGFKVR
NGPNGAEGYWVINAKEGKGKVTYNGGEKPDVTFTISDEDVVDLISGKLNPQKAFFQGKI
KIQGNMGLAMKLTDLQRQAAGRIESIRSKL

>ATJ44594.1 acetyltransferase 1 [*Helicoverpa armigera*]

MSANVVFGCVMALLIILFTISSMARYYIKFTLFTVMSLIFATAPVPLMLIKPFDPRNALIPAF
FLRCFAKILGLRWTVRGLENVDNSRGAVVLLNHQSALDLYALAIIWPLMSRCTVVAKRSL
QYLVPFGTATWLWGTVFIDRGAQTARDALNKQVDAIKNHKRKLLLFPEGTRHSGDKLLPL
RKGAFHVAMDAGAPIQPVVVSKYHYLDGKRQRFGSGEFIVSILPMIETEGMSKDDIGALIE
KTQTSMQEEFTKISMETLARRNLRNKAD

>ATJ44564.1 acetyltransferase 1 [*Helicoverpa assulta*]

MSANVVFGCVMALLIILFTISSMARYYIKFTLFTVMSLIFATAPVPLMLIKPFDPRNALIPAF
FLRCFAKILGLRWTVRGLENVDNSRGAVVLLNHQSALDLYALAIIWPLMSRCTVVAKRSL
QYLVPFGTATWLWGTVFIDRGAQTARDALNKQVDAIKNHKRKLLLFPEGTRHSGDKLLPL
RKGAFHVAMDAGAPIQPVVVSKYHYLDGKRQRFGSGEFIVSILPMIETEGMSKDDIGALIE
KTQTSMQEEFTKISMETLARRNLRNKAD

>ATJ44566.1 acetyltransferase 2 [*Helicoverpa assulta*]

MPRKVFVVGVGMTNFIKPSTGPDYPELGKEAVLAALADARIKYSDIQQAVCGYVFGDSTC
GQRVLYQVGMTGIPIFNVNNNCSTGSNALYLAKKLIEGGVSDVMLAVGFEKMAPGALSA
GVFNDRTNPMDRHTLKMAELAELTGAPMTAQYFGNAAAEHMKKYGTTELHLAKIAAKN
HRHGVKNPRAQGKREYTVEEVLNSRRIYGPLTKLECCPTSDGAGAAVLMSEEAVIKYGLQ
AKAVEIIGMEMATDTPAVFEENSLMKVAGFDMTALAAKRLYQNTGISPKQVDVVELHDCF
AANELITYEGLQLCGEGEAGKFVDAGDNTYGGRVVVNPSGGLIAKGHPLGATGLAQCAE
LVWQLRGEAGDRQVPRAKIALQHNLGLGGAVVVTMYRKGFADITPRPVAASGNPEDFKV
FKYMKILEEAMENDTENLIEKVRGIYGFKVRNGPNGAEGYWVINAKEGKGKVTYNGGE
KPDVTFTISDEDVVDLISGKLNPQKAFFQGKIKIQGNMGLAMKLTDLQRQAAGRIESIRSK
L

>ATJ44567.1 acetyltransferase 3 [*Helicoverpa assulta*]

MSVAAKGIFIVGAKRTAFGTFGGVFRNTSATELQTIAATAAIKEAGIAPEKVDTVVVGQVM
TASQTDGIFIPRHVALKSGIPQDRPALGVNRLCGSGFQSVVNSAQDILTGAAKISLAGGVEN
MSQAPFAVRNVRFGTALGSTYAFEDTLWAGLTDSYCGLPMGMTAEKLGAQFKITRDEAD
NFALRSQQRWKAAQDAGVFKNEITPVTLTVKRKEVKVEVDEHPRPQTTIEGLKKLPPVFK
KEGLVTAGTASGISDGAGAIVLASEEAAKGLKPLARLVGWSYVGVDPSIMGVGPVPAIEN
LLKVTKMTLNDIDLIEINEAFCAQTLSCAKALKLDMEKLNVNGGATALGHPLGASGSRIT
AHLVHELKRRGLKRGIGSACIGGGQGIALMVEAV

>ATJ44568.1 acetyltransferase 4 [*Helicoverpa assulta*]
MDGKTTKMPKVAKVKNKAPAEIQITAEQLLREAKERDLEILPPPPKQKISDPEELRDYQHR
KRKAFEDNIRKNRLVIGNWLKYAQWEESQKQVQRARSIYERALDVDHRNVTLWLKYTE
MEMRNRQVNHARNLWDRAVTILPRVSQFWYKYTYMEEMLENVAGARQVFERWMEWQ
PDEQAWQTYINFELRYKELDRARQIYERFVMVHPDVKNWIKYAKFEENHGFINGSRKVFE
RAVEFFGDEDLDERLFIAFAKFEENQKEHDRARVIYKYALDHIPKDRNKELYKAYTIHEKK
YGDRSGIEDVIVNKRKYMYEQEVIENPTNYDAWFDYIRLVENEGNVDDIRDTYERAIANV
PPSKDKQFWRRYIYLWINYALYEELEAEDAERTRQVYRTCLELLPHKIFTFSKIWLMYAQF
EVRCKDLKQARKTLGMALGICPRDKLYRGYIDLEIQLREFDRCRILYQKFLEYGPENCITW
IKFAELETLLGDIDRARAIYEIAVGQPRLDMPELLWKSYIDFEVQQGETEKARQLYERLLER
TVHVKVWLSYAKFELNAENADNFNVELARRVYERANDSLRSAGEKEARVLLLEAWKDF
ETEIGDEEKLEKVMAKMPRRVKKRQKIISESGVEEGWEEVFDYIFPEDEMVRPNLKLLAA
AKQWRKQKEVALPAEPETKNEDQDDEGHTPPQRNDDEIEN
>ATJ44565.1 acetyltransferase 5 [*Helicoverpa assulta*]
MAAYSSKLSLNDVVIASAVRTPIGSFKGSLSSLSATELGGVAVKAAVERAGIPKEEVKEVY
MGNVCSAALGQAPARQATIFGGLPKSTICTTVNKVCSSGMKAIVLATQGLQTGTQDVILA
GGMESMSNVPYYLKRGDTPYGGVQLNDGILYDGLTDVYNKIHMGNCAENTAKKLNISR
KEQDDYAISSYKRSATAYENKTFADELVPVPVPQKRGAAPVIFAEDEEYKKINFDKFTSLAT
VFQKENGTVTAGNASTLNDGAAAMVLMTADAAQRLNVKPLARVIGYADGECDPIDFPIA
PAVAIPKLLAKTGVKKEDVAMWEINEAFSVVALSNMKMLELDSNKINIHGGAVSLGHPIG
MSGARIVVHLCHALKKGEKGVAAICNGGGGATSIMIEKL
>ATJ44574.1 acetyltransferase 6 [*Helicoverpa assulta*]
MINLNILKQSTIVHLCFAISYFTSGLILTFIQAVLYFGLKPFNKSLYRKINYYLSYSFYSQLVF
MSEWWSNTKLSIYIKKDEYDKYYGKEHGYLIMNHSYEIDWLMGWHFCNTIGVLGNCKA
YAKKSIQYLPPIGWMWKFSEFVFLERSYEKDKETIKHQISELCDYPDPVWLLMTPEGTRYT
KKKHEASLSFAKEKNLPLLKHHLTPRTRGFTTSLQFFRGKIPVIYNIQLAFEKDSKTPPTLTS
LLYGKPVHAHLYIERIPVENIPVDEAEAAKWLHDLFVVKDKMQDSFFNTGDFFTESGVER
TEPFTVPPPIWSLVNALGWAVVTLTPMLYYLLGLLFSGKLLYFSIACAIFGAFFILLQKSIGM
SKISQGSSYGTEKK
>ATJ44571.1 acetyltransferase 7 [*Helicoverpa assulta*]
MLRRCSKHLQTLYRRQGQTLRFKSTEAPKVFGALSQAAARTTQPRVLSAAQNQIATIHFT
NQLFAEQDVMTPSFPDSVSEGDAKIEKKVGDAVAMDEVVMEIETDKTALPVMAPGNGIIK
EFYVKNGDTVKAGQKLFRLELTEGAPPPKAAAPPAPEPPKAEAPPPPPPAAAAPPPPPPPAA
AAPPPPPPPPAPKAEAPRAAPISSIPVAAIRHAQSIETATVKVPPTDYSKEMAGTRTEQRVKM
NRMRQRIAQRLKEAQNTNAMLTTFNEIDMSHIMAFRKKHLDAFTKKHGVKLGLMSPFVK
AAANALMDQPVVNAVIEDQEIIYRDYVDISVAVATPKGLVVPVVRNVQNMTYADIELTIAG
LAEKAKGGKLTIEEMDGGTFTISNGGVFGSLMGTPIINPPQSAILGMHGIFERPIALNGQVV
IRPMMYIALTYDHRLIDGREAVMFLRKIKEGVEDPATIIAGL
>ATJ44577.1 acetyltransferase 8 [*Helicoverpa assulta*]
MNIRCARPSDLMNMQHCNLLCLPENYQMKYYFYHGLSWPQLSYVAEDEKGHIVGYVLA
KMEEDGEDNRHGHITSLAVKRSHRRLGLAQKLMNQASLAMVECFQAKYVSLHVRKSNR
AALNLYTNSLGFKILEIEPKYYADGEDAYSMMRDLSAFAAESKDTQPTENLEIKSESAIISQ
C
>ATJ44569.1 acetyltransferase 9 [*Helicoverpa assulta*]

MALIMPFVSVAISILYTPLLLLILCIIFLASIGKSLGVRRLYVNILLKLFEYGRQHIEVAKIKIQ
RSDSSDEEELPPIPDDTPPSAIVKENGANGTKMTVIERHEILGPSPELNYKRSTSQERVQNG
HKTPQGNGENNMEFHLSNCLDLVKAGMESIIEDQVTSVFEAEELRSWNLLTRTNRQYEFL
TWRLTIIWAMGFVVRYMFLLPLRIMIFVIGVWWLVACTACIGTLPDGRTKQRVNYAISVMC
FNFLSRCISAVITYHDTDYKPKNGICVANHTSPIDALVLMCDNCYSLIGQRHNGFLGILQRA
LARASPHIWFERSEVKDRHAVARRLKEHISVADNPPILIFPEGTCINNTSVMQFKKGSFEVG
GTIYPVAIKYDPRFGDAFWNSSRYGMLHYLLNMMTSWAIVCDVWYLPAMTRAADESAV
DFANRVKAVIARRGGLVDLMWDGQLKRMKPKKEWRELQQEEISKRLKGE
>ATJ44573.1 acetyltransferase 10 [*Helicoverpa assulta*]
MDLVEHEWYIQAPWGRIAIIAWGDCCNPPVLLCHGSMDSAVSFRPLVSKLPRNYYYIGLD
LPGNGKSDRFLPGLMISVYDMLYAIHALVKHFRWKTFTLIGHSFGAYLGQFYNLCYPDKL
DRLINLDPINFFAVPPEEFSRWYHIFFTNFYKNYDKYNTPKEKSPTIKWTEALQSLMRNRPS
LNEEQAAAVLERLSEPVGDGCVRYTYDLRMKRINGPAYSPEHVKKLFTAVRTPILTIACQK
SLKNKLFRNTAFLLDEAEYPEGNFRFKSVEGGHDVHISHPERVAGFIGQFLEYGLEGLDKK
SKL
>ATJ44576.1 acetyltransferase 11 [*Helicoverpa assulta*]
MIRANTIIFKNNSVCPKFNQQRNFNKNVKDVEYQYLQRSKLPTMHFQKSLPRLPIPDLSKT
GERYLKALRPLLNDNQYEEAQQRTGNFIAKEGKILQEKLIAKDKRNKHTSYISEYWFDLY
LRDRVPLPINYNPMIVFQNDVRPEYNDQLIRSANILISSVRFMLSLREQILEPEVYHMNPKK
SDTPLFRNFTRMLPEAISWYGAYLMKVFPLDMSQFVGLFGATRLPRQTKDEIFRDPKSKH
VVVQKQGNFYVFDVLDANGNLLSPQEILGNLAQIMNDNTTAAEHPLGILTTQNRDVWAK
QRTHLESTGNSEVLNKIDSAIFNLILDDDTINDDKRVLLKKYLHSDGLNRWFDKSFSLIVTR
DGVAGVNFEHSWGDGVAVLRFFQDIYAETTKKPFIHPDSKPADSNISVQKLEFKLDDKSKQ
FIDNAKKEYKAWYNSLSIDYILYEGLNKAACKKFKVSPDCIMQLSFQAAHHLLKGNFVGT
YESCSTSAFKHGRTETMRPCTDKTKAFCETLHSNNTSIDELRAKLTECSKLHLELVKDAA
MGQGFDRHMFALMKMAEDNNMPRPEIFDSYEYKFLNKSILSTSTLSSPSVMAGGFGPVV
KEGFGIAYSAFPDKLGAAVASYKPHNDSSQYIEALHKSFLDITKILSG
>ATJ44575.1 acetyltransferase 12 [*Helicoverpa assulta*]
MALKIVFLGLVLFISPILCYKPVVLIHGVMTGSASMEMIKFRIEEQHPGTIVYNVNRFESWS
SLETMWHQVLEIGMDIANISASHPEGINLIGYSQGGLIARGIVETFPNVSVSTFISLSSPQAG
QYGAGFLHLVFPGLVKDTVYELFYSRVGQHTSVGNYWNDPYHQSLYETYSVYLPYINNHI
KSAKSEDFKKNLLRLKRLVLIGGPDDNVITPWQSSQFGYYDANETIIEMKSQDIYMEDKIG
LRTLDESGRLHVVTVPGVNHFSWHMNISIVDDYLLPYLD
>ATJ44588.1 acetyltransferase 13 [*Helicoverpa assulta*]
MAFAGLKKQINKANQYVTEKMGGAEGTKLDLDFVEMERKTDVTCELVEELQTKTKEFL
QPNPTARAKMAAVKGISKLSGQAKSNTYPQPEGVLGDCMLLYGKKLGEDTVFSNCLIEM
GEALKQMADVKYSLDDNIKQNFLEPLHHLQTKDLKEVMHHRKKLQGRRLDFDCKRRRQ
AKGAHIADDEIRQAEEKFAESLQLAQIGMFNLLDNDVEQVAQLTYFAESLLEYHQQCTEIL
KGLVSTLMEKKEEAVNRPKMEFVPKTLADLHIEGIHDLNNGRRYGSTQSLSRPRQHIPPSS
SVGDLSTTDPFKAWEAPSPVRAQVRPAPGFKPHPAPRNQFNGRDPWKASPLPSPVKSPART
PVVANKTPCCTALYDFEPENQGELGFKENDVITLINKVDDNWFEGSVNGKTGYFPISYVQ
VTVPLPNM
>ATJ44570.1 acetyltransferase 14 [*Helicoverpa assulta*]
MASKRSNTFKVLLASLAVAALGYVLRSPSLYLRRETKSSLGYPKDSLLNFTELTAEYGYLS

EEHKVLTDDGYILTMFRIVKARNCHRAKRSPPVLLMHGLLQSSDSWIDSGPDAGLAYLISD
ACYDLWLGNVRGNYYSRGHVRLDPDKDPAYWKFYIEEIGIYDVPAMIDYVLDYTGFEKL
NYIGFSQGTGTFLVMCSEKPGYCDKVKLVISLAPASRQMHTQSKIFRTVTQTFYKMEGLLS
MTGLQEVFSKGGFSQEFVAFFCQLSGVTERLCEKVIDAFDHVDSTHLGSITNHTTRVLFGH
FPAGTSVHNMARYGQSMNSGRFEKFDYGREQNLVLYGSEEPPQYNLSATTVPVMCIYGK
NDGLVDTKDVEWLMAQLPNVLEMVKVEDPQWNHMDVTYSQYTGDTIFPKINEYLLKYT
ST
>ATJ44580.1 acetyltransferase 15 [*Helicoverpa assulta*]
MAKRLVSRLILNSNGTTLKSPLPILSKKVHTQIPVKEIQIPVKFGHMSGKLWGSGDKQPILA
LHGWQDNAGTWDPLIPMIIKDRPILALDFPGHGLSSWIPPGMLYYQWELPRVILYLKEYFK
MEKVSLMSHSMGAIASMRFATVFPDDVEFFIAIDSLIYDDYDLNSVVNRISKTLRKGLIAQ
TRLDQEPPAYTMEEMIKIWHLGTRKSVSMESVPHLLKRGAKQTKSDPTKYYFSRDSRLKY
TLFNPEDRKFVEALVKRLKCPTLYVKAIDSPFSADPYSIEMREILEQINDKYEFHFVPGTHH
VHLNNPELVAPLIKNFVQKHNLSI
>ATJ44590.1 acetyltransferase 16 [*Helicoverpa assulta*]
MWYKIFIFTIVCVLTYILKKLHDTGPNRVKFYFNFFLFYFLSSMLAAVIWPYFLLSPRNVRN
AKIAVRLLKHITKLYDLKWHLRDGKILAEDRGAVIISNHQSSLDILGMFNIWEVVDKLAAI
AKKELFYVWPFGLSAYLAGVVYIDRRNAKGAYKQLKVTSEVMVKNKTKIWLFPEGTRN
KDYTKLQPFKKGAFNIAVAAQVPIIPVVFSPYYFINKEKYIFNKGHVIIQCLEPVPTVGLTME
DVPDLIDRVHHKMSVAYQEISKEVFSSLPSDYPVTLKG
>ATJ44579.1 acetyltransferase 17 [*Helicoverpa assulta*]
MASQISKSLLKVSHVSSTAKFDTARRALSVGAALQQKKKSLPDRTGKNVVLVDGVRTPFL
VSFTDYAKMMPHELARHSLLGLLQKTGISKDLIDYIVYGTVIQEVKTSNIGREAALAAGFS
DKTPAHTVTMACISSNQAITTGVGMIAAGAYDIIVAGGVEFMSDVPIRHSRKMRSLLLRLN
RAKTPAQRLSLIASIRPDFFAPELPAVAEFSSGETMGHSADRLAAAFGASRQEQDDYALRSH
KLAHEAQQKGYFTDLMPVKVDGKDGVVDKDNGIRVSTPEQLAKLKPAFIKPHGTVTAAN
ASFLTDGASACLVMSEAKAKELGLKPKAYLRDFTYVAQDPVDQLLLGPAYGIPKILDKAG
LKMSDVDTWEIHEAFAGQILANLKAMDSDWFAQTYLGRQTKVGAPDLDKWNKWGGSL
SIGHPFAATGVRLAMHTAHRLVREDGQFGVISACAAGGQGVAMILERHPDATCN
>ATJ44585.1 acetyltransferase 18 [*Helicoverpa assulta*]
MFGVALCLLDIIGLNPIPSIAVTIGATVPAVKLLISILLGYPLAIIYHKYVRKTVELRNLYFIVT
GMDLAYFNFGTSMYHNAIPAIVIYLSTKYLGPGKMNAIFTFAFNMVYLLAGYVMTESEDY
DITWTMPHCVLTLKLIALSFDIWDGEKFLKGEELSENNLKTALPTQPTFLELIGFVYFPACF
LVGPIFSFRRYRDYISDTFPLDASADVYEQLALKRLIQGVCYLAAYQIGVSVFSMKSMLSD
EFWDTSIFYRHFYCGLWAHFALYKYISCWLLTEAACIRFGLSFNGFVDSTQTMTKWDGCN
NIKLMRFESATRFQHYIDSFNCNTNHFAAEYVYKRLRFLGNRNLSQLITLVFLALWHGTQS
GYYMTFLNEFLIMVMEKDVEALLTKTEFYHKMWNIPYVKYPLYVILKTYTIVFMGWSLA
PFDVKSFGKWWSIYTSLYFSGFILFLPWSFVYKPIIVKLLKRNAASHSKTS
>ATJ44587.1 acetyltransferase 19 [*Helicoverpa assulta*]
MAVVINKGIFIVAAKRTPFGRFGGAFKEVYPSDLLAAAAKDALKAGSVAPEIIDTVNIGQV
YGISGSSDGGLSPRHAALKSGIPQEKPALGISRLCGSGFQAVVNSAQDIITGAANISLAGGT
ENMSTVPFVVRNTRFGVGLGAKMPFEDVLTSSSLDTSCNFTMPETAENLAEKYGLQRME
VDQFALQSQQRWKAAHDQGVFKAEMTPVTVKVKRQEKVVEVDEHPRPDTTTEMLSRLP
VLFRKGGVVTAGNSSGVNDGAGALILATEESVKQHGLKPLVRLLAWSVVGVDPSVMGIG

PVPAIQNLLSATGLKLDDIDLVEINEAFAAQTLACAKELGLDQSKLNVNGGAIAMGHPVG
ASGARITAHLAHELRRRGLKRGIGSACIGGGQGIALLLETV
>ATJ44578.1 acetyltransferase 20 [*Helicoverpa assulta*]
MMEHISNIFEVLTKTFSQISDLLGIQWAPMNIPMSRRLQTLAAFVWIYLILFGEALAIYLFIQ
LVYSRFWWAAILYGVWMLNDIDICHRGGRVSQWVRNWTWWRYLCDYFPINLVKTVDLD
PSKNYMFAIFPHGVISLGAFGSFCTNATNFHKLFPGMSCHLITLGGHFLVPFFRDLALAIGM
CASSEQSLLHLLDQKKYEGNAVCMIIGGAAEALDAHPKEYKVILSRRKGFIRVAMKSGAS
LVPVFSFGETDLFHPPNNPENSLLRRFQEKVRQWTGISPMFPMGRGMFQYSYGVLPIRSPV
TTVVGAPMEVKRNLEPTNEEIDAVHAEFTKRLQTLFETEKVKYLKYHEEAKLVIT
>ATJ44584.1 acetyltransferase 21 [*Helicoverpa assulta*]
MSFLMRKCVVNLKNVNRYSAVCVLMQSERHRSRFSSSNVLKRNRLLSDVHLQHKKIHTS
QIVNKTVAFKLSDIGEGIREVVIKEWFVKVGDKVQQFDNICEVQSDKAAVTITSRYDGVVT
KLYHDVDTTALVGQPLVDIEVQGEADEGSSSSSPEEQPKVTKQESVEEKSQRIKVLTTPAVR
RIAAQFNVDLSTVKATGRNGRVLKEDMLAHLNIDSDGSNEVPAPSSVQAMSIPLTQAKAK
VEVLLEDKVVPVTGFTKAMVKSMTEAMKIPHFGYSDEYDVTKLVESREALKKIAEARGA
KLTYMPIIIKAASLSLEQLPVLNSSLDSTCEHLTYKASHNIGVAMDTPNGLIVPVIKNVQNK
TILEIARELNTLQERGSKGQLGLNELSGGTFTLSNIGIVGGTYTKPVILPPQVAIGALGKIQL
LPRFDAEGNVRKAHILTVSFSADHRVVDGVTMARFSNFLKNYLENPYTLLLDL
>ATJ44583.1 acetyltransferase 22 [*Helicoverpa assulta*]
MELQDTYYNKSEYVETASGNKVSRQTVLCGSQNIVLHGKVIVQSDAIIRGDLANVKTGRF
CIISKGSVIRPPFKKFSKGVAFFPLQMGDHVFVGENTVVNAAVVGSYVYIGKNVVIGRRCV
LKDCCMIEDNSVLPAETVVPSFARYSGSPARLITMLPEAMPDLMTEFTKSYYQHFLPTTVQ
>ATJ44582.1 acetyltransferase 23 [*Helicoverpa assulta*]
MTYYDYYDGSRIFLFIANKVGLPLDLVNFLIAQVAALCLARLFRKPLRYASPEFRHSVCLVI
GLTMGYFCFGRQAIHLSVLPMLTYTLLKSIKHQIMGNVILAVSMIYLSCLHLHRQIYHTAD
YTLDITGPLMVITQRVTSLAYSLQDSLTVKDLKSKATGLQTTGGDDLVKIEKIPSPLEYFAF
TLAFQTLMCGPVVFYTDYIKFIEGARVDELEKSADTKEPSPRTAVFYKVAGSLAAALLYLT
LAKKYPLTVLEELTDPTSEVSRSWSALYLLWYAYLSTLVVRCKYYHAWLLSEAICNNCGM
GFNGYNNDGSPKWDKMSNIDIFGFEFAQNFRIAIASWNKNTNAWLRDVAYSRGGAAWRT
ARVYALSAVWHGFHPGYYLTFFAGGIFTVAARKIRFVARPMFLDSVPKKLFYDSVSFITTRV
AMTYATVPFVLLHLAPSLAFYAKFYYSLHFIALGAMLIPEKAKRPKPAVVQEQQSKSPKMS
KEAIEESLEELNGKLKIS
>ATJ44572.1 acetyltransferase 24 [*Helicoverpa assulta*]
MKKLFILLCVIKLISGTPTPIVLWHGMGDTCCLSFSLGGIKVFLEKNIPGVYVNSLKVGNSS
IEDLENGYFMNPNKQVEYVCGLLAADPQLKDGFNAIGFSQGSQFLRAVVQRCGHILPKM
KNLISLGGQHQGVYGLPHCGALMHPTCDYIRQLLNYAAYENWVQNALVQATYWHDPLD
DETYVHKSIFLSDINNEIMANKTYIQNLNNLDHLVLVKFDNDTIVQPRETEWFGYYEPGQS
KKLLPLRETKLYTEDRLGLKKMDKEGKLILLSTVGDHLRFSDTWFIDNILKPYLLN
>ATJ44581.1 acetyltransferase 25 [*Helicoverpa assulta*]
MSEQIEFVDILEPRRTQSGIFSFMTRNWHPQRTLKLDKYFTPQELKDIAASSVYLDAFIEAE
CSRSGQSKEKLHQEVHNYLEEMGLDKKMHVIRWMGVIFLKISFMMKIKMFVNEAAAFN
LKSVMGNNPVLFLPTHRSYADFCLMTYLCYHFDIDFPAVAAGMDFYSMAVIGRRMRETC
AFYIRRTLAGDPLYAATLKQYVRTVVAKHSAPIEFFLEGTRSRSNKSMPPKYGMLSMTLVP
YFAHEVTDITVVPVNISYDRLMEHSLFAYEHLGVPKPKESTGGFLKALHTLNDHFGNIYIN

LGAPLSIREFLKNDTSHSQETLKPLDMQQLTPDQFKQVQSIADYVITLQQKNTVATISNLLS
LVLMQSLMKNVPLEFEEVLQEVGWMVQELRNLGATVFENDVRSSVERILVVHRKMMRL
DKERRLRLISGILVDLSSEVKKKMKGHILQAQTMVAVVPVIQLQLYVNPILHYLVPPAIICLI
VHRSAAGRDCLEADYHRVRKLLSDEFFHLEKEEPNTFAKAVEYCIQNSVISYNGELYAMG
EDTKLQYLLKWSVWPALTSLLKCAQVMTEQSSCAHKQALKLVQQRVESERVHPYCLSLE
ATANCLNGLVAANALVRNKGEGDIVYELVPHTMQECNKLVSSILPTFSVDFTNNAVVVDH
KALSRL

>ATJ44592.1 acetyltransferase 26 [*Helicoverpa assulta*]
MWDKIIYMIVIIVVTYILKQLFSETPNFVKFKSKFLVFYIWTSVTAVILLPFFVFNPKNVKNS
LFGSQIVKHVTKVIEVKWLLRNGKVLAEDRGAVVVSNHQSSIDILGMFNIWHVADKVAAI
ARKEIFYVWPFGLAAYLAGVVFIDRNNSKDAYKQLKITSEVMIKNKTKIWLFPEGTRNKD
FTKLLPFKKGAFNIAVAAQVPIIPVVFSPYYFINRKKYIFNKGHAVIQCLEPVPTVGLTMED
VPALINKVRNTMDAAYKELSKEVLSALPPNYPLATD

>ATJ44586.1 acetyltransferase 27 [*Helicoverpa assulta*]
MGARNLKVLQVISGWQAVELILKCVFVGIWQIIEISVKGLWKGARRKVKDTPPVELTIDSS
VGTHCYIKVMGVKYHYVETGPRTGQKVLILKDAPDSGNLWGPNWANAVRRLAETDHHV
MTLDLRGTGGSEGGSRSELSPPRAVEELSALLKALGVTENRPAVVIGFGVGGMLAWYLVH
SRGPLISKFAVINAPHPNLYWQFPPAAFCHRVLHFIQWPHFPERWFAEGELNDCDGRWASS
RACDWTGALNYVRGAAWWQVRPGLRTSAPALLVGDKDSAGQLVASAQHCTTSTLRLVT
KPEPNSKELAVVLLDFLISKEKLIEEVPRGLMGRMFGAVADRGRELTARLVLPPTQA

>ATJ44593.1 acetyltransferase 28 [*Helicoverpa assulta*]
MEHWNSEQCYIVISNHQSSLDILGMFEMWPQMKRCTVVAKRPLMFTGAFGFGAWLSGLV
FIDRLRTERARQLMKDATARVIKEKTKLWIFPEGARFNKGSIQNFKKGAFYLAIDAQIPIMP
VVFSQYYFLDSETKTFEPGKVIITTLPPIPTSGMTRNDVETLSEMARQQMIEVFHESSKDLV
MQKKIAI

>ATJ44591.1 acetyltransferase 29 [*Helicoverpa assulta*]
MSSMPMLVRNVRFGTALGTPYRFEDHIQRQIPDGYTGLTMQKMVENLANKYGVTREDA
DEFALQSHLKWKEAEESKAFQQEVVSLEVTLKKKQILVDKDQIAQPLKSEDLSRLPTLIDN
GTILTTGNTSAPVDGAAALLLADEEAVRGHSLQPLARVAGWTCVGVNPEDAGLGGVLAI
KKLLDSQKLTVGDVDIFEINENFASQAVIATRELKIDQSKVNVSGGALALGDPMSATGARM
ATHLVHELRRRNLKRGIAASSCGGGQGVAILLEKM

>ATJ44589.1 acetyltransferase 30 [*Helicoverpa assulta*]
MAVAVKKGVYIVAGKRTPFGKFGGLLQDVLAEDLFAIAATAAFQAGNVDAELVDTVNIGQ
VSPVSQNGLAPRHAALKAGIPSDRPVLGVNKLSGSGFNAIICGAQEILTGSAQITLAGGME
NLSSLPLLIRDMRFGNPLGKYFKVDDLLHMGFFDSYCNLFLVQTADIVAAKFGVTREEAD
EFALRSQQRWKTADAAGLFGEELVPVPVKLNNREILITKDEYPQPDTTLEKLSKLQPMFEG
GIATPGNSSGINDGAAAIILANDEALKTHNLKPLARLVGWSCTGVDPSMLGIAAAPAAQNL
LNCTGLSIDDVDLVEIHETYAATSLFCARQLAVDDNRLNVKGGAISIGHAFGASGVRIISHL
THELRRRRLKRALATTAIAGGQGVAVMIETV

>AGQ45622.1 acetyltransferase 1 [*Agrotis ipsilon*]
MAVAINKGIFIVAAKRTPFGRFGGAFKDVYPSDLLAAAAKDALKAGSVAPEVIDTVNIGQV
YGLSGSSDGGLSPRHAALKSGIPEDKPALGISRLCGSGFQAVVNSAQDILTGVAQTSLAGG
TENMSTVPFIVRNTRFGVNLGVKMPFEDLLTASSLDTSCNNTMPQTAENLAEKYGLHRME
VDEYALQSQQRWKAAQDQGAFKAEMTPVTVKVKRQDKVIEVDEHPRPETTTEMLSKLP

VLFRKGASLLLATLLVSTTVLER
>AGQ45623.1 acetyltransferase [*Agrotis ipsilon*]
MTANVILGCVMALLVILFTISSIARYYIKFTLFVVMSLIFATAPVPLMLIKPFDPRNALIPAFF
LRCFARILGLRWKVRGLENVDNSRGAVVLLNHQSCLDLYALAIIWPLMSRSTVVSKRSLQ
YLVPFGTATWLWGTVFIDRGAQSARDALNKQVDAIKNQKRKLLLFPEGTRHSGDKLLPLR
KGAFHVAMDAAAPIQPVVISKYHHLDGERHKFGSGEFIVSILPMIETEGMTKEDITELIEKV
QTSMQEEFTKISLETLERRNLRTKAD
>AGQ45624.1 acetyltransferase [*Agrotis ipsilon*]
MSAAAKGIFIVGAKRTAFGTFGGAFRNTSATELQTVAAVAALKEAGVAPEKVDSVVVGQV
MTASQTDGIYLPRHVMLKAGIPQDKPALGVNRLCGSGFQSVVNSAQDILTGSAKISLAGG
VENMSQAPFAVRNVRFGTALGQNYAFEDTLWAGLTDSYCGLPMGMTAEKLGAKFGITRD
EVDNFALRSQQRWKAAQDAGAFKAEIAPVTLTVKRKEVKVEVDEHPRPQTTIEGLKKLPP
VFKKEGIVTAGTASGISDGAGAIVLASEEAAKGLKPLARLVGWSYVGVDPSIMGVGPVPAI
ENLLKVTKMTLNDIDLIEINEAFCAQTLVLCQGPQAGR
>AGQ45625.1 acetyltransferase [*Agrotis ipsilon*]
MNIRCARPSDLMNMQHCNLLCLPENYQMKYYFYHGLSWPQLSYVAEDEKGHIVGYVLA
KMEEDGEDNRHGHITSLAVKRSHRRLGLAQKLMNQASLAMVECFQAKYVSLHVRKSNR
AALNLYTNSLGFKILEIEPKYYADGEDAYSMMRDLSAFVAESKTELPPIENLEIKSESAIISQ
C
>AGQ45626.1 acetyltransferase, partial [*Agrotis ipsilon*]
PFELFITAMAPTKLSLNEVVIASAVRTPIGSFKGTLASVPATELGAICVRAAVERAGIPPEEV
KEVFMGNVCSAAMRQAPARQAAIFGGLSKSTICTTVNKVCSSGMKTILLAVQGLQTGTH
DVILAGGMESMSNIPFYLKRDNIPYGGTQLLDGILYDGLTDVYNQIHMGDCGEHVAKTFN
FSREQQDDYAIAGYKKVAAAYEANAFADELVPVPVPQKKGAAPIIISEDEEYKKVDFEKLR
KLPPVFQENGTITAANAAALNDGGAAMILMTAEAAQRLNVKPLARVIGYADAECDPIDFPI
APTLAIPKLLEKTGVRKEDIALWEINEPFSIV
>ACX53699.1 acetyl-CoA acetyltransferase, partial [*Heliothis virescens*]
MAVAVKKGVYIVAAKRTPFGKFGGLLRDVLAEDLFAIAATAALRAGNVAAELVDTVNIGQ
ASPVSQSGLSPRHAALKAGIPSDRPVLGMNRLSGSGFHAIICGAQEILIGSAQ
>ACX53799.1 acetyltransferase, partial [*Heliothis virescens*]
MSVAAKGIFIVGAKRTASGTFGGVFRNTSATELQTIAATAAIKEAGIAPEKVDTIVVGQVM
TTSQTDGIFIPRHVALKSGIPQDRPALGVNRLCGSGFQSVVNSAQDILTGAAKISLAGGVEN
MSQAPFAVRNVRFGTALGSTYAFEDTLWAGLTDSYCG
>ACX53783.1 acetyltransferase, partial [*Heliothis virescens*]
EIIDTVNIGQVYGISGSSDGGLSPRHAALKSGIPQEKPALGISRLCGSGFQAVVNSAQDIITG
AANVSLAGGTENMSTVPFVVRNTRFGVGLGAKMPFEDVLTSSSLDTSCNFTMPETAENL
AEKYGLQRMEVDQFALQSQQRWKAAHDQGVFKAEMTPVTVKVKRQDKVVEVDEHPRP
DTTTDMLSRLPVLFR
>AIN34698.1 fatty alcohol acetyltransferase [*Agrotis segetum*]
MINLHILKQSTVVHLCFAISYFTSGLILTFIQAILYFGLKPFNKSLYRKINYYLSYSFYSQLVF
MSEWWSNSKLSIYIKKDEYEKFYGKEHGYLIMNHSYEIDWLMGWHFCNTIGVLGNCKA
YAKKSIQYLPPIGWMWKFSEFVFLERSFEKDKETIKYQISELCDYPDPVWLLMTPEGTRYT
KKKHEASLSFAKEKNLPLLKHHLTPRTRGFTTSLQFFRGKIPVIYNIQLAFEKDSKTPPTLTS
LLYGKPVHAHLYIERIPVERVPEDEAEAAKWLHDLFVVKDKMQDSFFNTGDFFLESGVER

REPFSVPPPIWSLVNALGWAVVTLTPMLYYLLGLLFSGKLLYFSIGCGIFGAFFILLQKSIGM
SKISQGSSYGTEKK
>AIN34709.1 fatty alcohol acetyltransferase [*Agrotis segetum*]
MSSRTPLTKAEKVAYALRHEKSYSWHKWFSVLAVLTIIVSLLTYYLFGMWTEPPPLPKLDL
EQHWGPYPIDMKPDNSIRPFTIEFSDVIVNDLRERLLHRRSFTPPLENAGFTYGFNTHFLTQ
VLDFWQNKYNFKEREQFLNKYEHFVTNIQGLDIHYMHVKPKVPGNVTVVPLLLIHGWPG
SIREFYEIIPKLTTPRPNQEFVFEVIAPSIPGFGFSQAPVRAGMGPIQVSVIFRNLMQRIGHDE
YYVQGGDYGSAIGSVMATLFPENILGYHTNMPMVAVNTWVSIYTVLGSLWPNFIVEPSVQ
DRMYPLSKHIGKVIEETGYFHIQATKPDTVGIALSDSPAGLAAYILEKFSTWTNMENKKAS
DGALLQKFSLTHLLDNVMIYWASNTITSSMRHYVEGYKQLMFTDRIPTEVPTWGIKFKHE
ISFQPDSILKLKYKNYLHSSVVEDGGHFAAMELPDVLADDIFDAVHMFRTFHRKKRNNKA
SDKPITKESTKPDAETVNKVEKESKVNFETVNKVEKEPKVNFETAKTVYEFTVKDIQGQE
VKLERYKGKVLIIVNVASHCGYTNSHYTELNELYEKYSKKGLRILAFPCNQFGGQEPGTLK
EILQFTKEKKVKFDLFEKIEVNGENAHPLWKFLKRIQGGTLGDFIKWNFSKFIIDRNGVPV
ERFGPNTSPLELEPYLEKLLG
>AGG54993.1 acyltransferase AGPAT2 [*Heliothis virescens*]
MSANVVFGCVMALLIILFTISSMARYYIKFTLFIVMSLIFATAPVPLMLIKPFDPRNALIPAFF
LRCFAKILGLRWTVRGLENVDNSRGAVVLLNHQSALDLYALAIIWPLMSRCTVVAKRSLQ
YLVPFGTATWLWGTVFIDRGAQTARDALNKQVDAIKNQKRKLLLFPEGTRHSGDKLLPLR
KGAFHVAMDAGAPIQPVVISKYHYLDGKRQRFGSGEFIVSILPMIETEGMTKDDIGALIEK
TQMNMQEEFTKISMETLARRNLRNKAD
>AIN34682.1 fatty alcohol acetyltransferase [*Agrotis segetum*]
MLFLKGSRIITIKMRPTNKLFKAMAAYSSKVTLNEVVIASAVRTPIGSFRGSLASLSASELG
AVAVKAAVERAGIPKEEIKEVYIGNVCSAGMGQAPARQAVIFSGLPKSTICTTVNKVCSSG
MKAIVLAAQGLQTGTHDVILAGGMESMSNVPFYMKRGDIPYGGTQLIDGIVFDGLTDVY
NKFHMGNCAENTAKKFNISRQQQDEYAISSYKRSAAAYESKAFADELVPVPVPQKRGAAP
IMFSEDEEYKKVNFEKFSKLGTVFQKENGTVTAGNASTLNDGASAMVLMTAEAAQRLNV
KPIARVVGYADGECDPIDFPIAPAVAIPKLLAKTGVKKEDVAMWEINEAFSVVALANIKML
ELDPSKLNIHGGGVSLGHPIGMSGNRIVVHLCHALKKGEKGVAAICNGGGGASSIMIEKLE
HTTDGLPVMTFYTKDPCGLCDIVMEELEPYKNRIVIQKVDITQKENVRWLKLYRHDIPVL
FLNGQFLCMHKLDKHLLENRLQKIEDGKLH
>AIN34685.1 fatty alcohol acetyltransferase [*Agrotis segetum*]
MDGKTTKMPKVAKVKNKAPAEIQITAEQLLREAKERDLEILPPPPKQKISDPEELRDYQHR
KRKAFEDNIRKNRLVIGNWLKYAQWEESQKQVQRARSIYERALDVDHRNVTLWLKYTE
MEMRNRQVNHARNLWDRAVTILPRVSQFWYKYTYMEEMLENVAGARQVFERWMEWQ
PDEQAWQTYINFELRYKELDRARQIYERFVMVHPDVKNWIKYAKFEENHGFINGARKVF
ERAVEFFGDEELDERLFIAFAKFEENQKEHDRARVIYKYALDHIPKDRNKELYKAYTIHEK
KYGDRSGIEDVIVNKRKYMYEQEVIENPTNYDAWFDYIRLVENEGNVDDIRDTYERAIAN
VPPSKDKQFWRRYIYLWINYALYEELEAEDAERTRQVYRTCLELIPHKIFTFSKIWLMYAQ
FEVRCKDLKQARKTLGMALGICPRDKLYRGYIDLEIQLREFDRCRILYQKFLEYGPENCIT
WIKFAELETLLGDIDRARAIYEIAVGQPRLDMPELLWKSYIDFEVQQGETEKARQLYERLL
ERTVHVKVWLSYAKFELNAENADNINVDLARRVYERANDSLRSAGEKEARVLLLEAWK
DFETEIGEEEKLEKVMAKMPRRVKKRQKIISESGVEEGWEEVFDYIFPEDEMVRPNLKLL
AAAKNWRKQKEVTQPTETENKQDEEEEGQTPPQRMNEVEDD

>AGQ45625.1 acetyltransferase [*Agrotis ipsilon*]
MNIRCARPSDLMNMQHCNLLCLPENYQMKYYFYHGLSWPQLSYVAEDEKGHIVGYVLA
KMEEDGEDNRHGHITSLAVKRSHRRLGLAQKLMNQASLAMVECFQAKYVSLHVRKSNR
AALNLYTNSLGFKILEIEPKYYADGEDAYSMMRDLSAFVAESKTELPPIENLEIKSESAIISQ
C
>AIN34706.1 fatty alcohol acetyltransferase [*Agrotis segetum*]
MLRRCSKHLQTLYRRQGQTLRFKSSEAPKVFGALSQAAARTTQPRVLTAAHNQVATIHFT
NPLFAEQDVMTPSFPDSVSEGDAKLGDKKVGDAVAVDEVVMEIETDKTALPVMAPGNGII
KEFYVKDGDTVKAGQKLFRLELTEGGPPPKAAAPAPEPPKADAPPPPPPAAAAPPPPPPPA
AAIPTPPPPPPQAPPAAKPAAPISSIPVAAIRHAQSIETATVKVPPTDYSKEIAGTRTEQRVKM
NRMRQRISQRLKEAQNTNAMLTTFNEIDMSHIMAFRKKHLDAFTKKHGVKLGLMSPFVK
AAANALVDQPVVNAVIEDTEIIYRDYVDISVAVATPKGLVVPVVRNVQNMTFADIELTIAGL
AEKAKKGKLTIEEMDGGTFTISNGGVFGSLMGTPIINPPQSAILGMHGIFERPIALNGQVVI
RPMMYIALTYDHRLIDGREAVMFLRKIKEGVEDPATIIAGL
>AIN34712.1 fatty alcohol acetyltransferase [*Agrotis segetum*]
MALVMPFVSVAISILYTPLLLLILCIIFLASIGKSLGVRRLYVNILLKLFEYGRQHIEVAKIKIQ
RTDSSDDEEVPPAPDDDKPPSATIKENGVNGTLNTVIERQEILGPSPELNYKRSTSQERVQN
GHSKPSQGNGENNIEFHLSNCLDLVKAGMESIIEDQVTSVFEAEELRSWNLLTRTNRQYEF
LTWRLTIIWAMGFVVRYMFLLPLRIMIFVIGVWWLVACTACVGTLPDGKTKQRVNYAVSL
MCFNFLSRCISAVITYHDTHYKPRNGICVANHTSPIDALVLMCDNCYSLIGQRHNGFLGILQ
RALARASPHIWFERSEVKDRHAVARRLKEHISVPDNPPILIFPEGTCINNTSVMQFKKGSFE
VGGTIYPVAIKYDPRFGDAFWNSSRYGMLHYLLNMMTSWAIVCDVWYLPAMTRAHDES
AVDFANRVKAVIARRGGLVDLMWDGQLKRMKPKKEWRELQQEEISKRLKGE
>AIN34699.1 fatty alcohol acetyltransferase [*Agrotis segetum*]
MAFAGLKKQINKANQYVTEKMGGAEGTKLDLDFVEMERKTDVTCELVEELQAKTKEFL
QPNPTARAKMAAVKGISKLSGQAKSNTYPQPEGVLGDCMLLYGKKLGEDTVFSNCLIEM
GEALKQMADVKYSLDDNIKQNFLEPLHHLQTKDLKEVMHHRKKLQGRRLDFDCKRRRQ
AKGAHIADDEIRQAEEKFAESLQLAQIGMFNLLDNDVEQVAQLTYFAESLLEYHQQCTEIL
KGLVATLMEKKEEAVNRPKMEFVPKTLADLHIEGIHDLNNGRRYGSTQSLSRPRQHIPPSS
SVGDLSNTDPFTAWEAPPAYRAQARPAQTRPAPGFKPHPAPRNQINGRDPWKASPLPSPVK
SPARTPVAPNKTPCCTALYDFEAENQGELGFKENDVITLINKVDDNWFEGSVHGKTGYFPI
SYVQVTVPLPNM
>AIN34710.1 fatty alcohol acetyltransferase [*Agrotis segetum*]
MSFLMRKCIVNLKNVNRCSSVCVLMQTGKHRSQYSTSNILKRSIVPSDVHLRQRKFHTSQ
IVNKIVAFKLSDIGEGIREVVIKEWFVKVGDKVQQFDNICEVQSDKAAVTITSRYDGVVTK
LYHEVDQTALVGQPLVDIEVQGGADEGTSSAPESIPAAAAKQESVADKSQKVKILTTPSVR
RIAAQFKVDLSSVKATGRNGRVLKEDMLAHLNISSDKSNEIHEPSSISAMAIPLVPAQAKM
EVMLEDRVVPVSGFTKAMVKSMTEAMKIPHFGYSDEYDVTKLVESRESLKKLAEAKGVK
LTYMPIIIKATSLGLEQIPVLNSSLDSTCEHLTYKASHNIGVAMDTPNGLIVPVIKNVQAKTI
LEVARELNTLQEKGSKGQLGLSELTGGTFTLSNIGIVGGTYTKPVILPPQVAIGALGKIQAL
PRFDVEGNLRKAHILTVSFSADHRVIDGVTMARFSNLLKNYLENPYSLLLDL
>AIN34702.1 fatty alcohol acetyltransferase [*Agrotis segetum*]
MWQSSIIIFAVLLVQVYSAPQFITFKEGKLGVNFGGYHAGVGLGGVAGGSNTAGGLFAEA
GTPFGQGAKAGLGGAVNGNSGTAGGLYAAATAGGNVNAAAGLGGAVAGGKSVGGGFST

AQAGGKSATSVLGGESDVSGSSGFSIEAHKSIGVPTTVVKETKVSIVPVEEVKNVQGEAKF
EATNEIAPSANAGAEGNINAYVNVNAKPEIVKEVSTWKGPYYHTSKIPPFDFQDFMSSLFR
SPQGSYSPPMWAPPPQYNYIQQIHAEPTPVVQTIYLRKHKPHRHHVHKAVYVGGYAGVG
GEVAPPVQQTVVYKTVQPIEKRVDVNVDVNAHGGAGAAVSGEHYGPSSGVTYTKQVAV
NSRPSTFFQDIFNIPISTLKAVSGFLSNTAQNTGISVQKSASFNAGGYSGFSGKAGYSGHSG
YYSY
>AIN34708.1 fatty alcohol acetyltransferase [*Agrotis segetum*]
MIGANKLIGKNNIVYQKFIQQRKFTNKNIKDVNYQYLQRSKLPTMHFQKSLPRLPIPELSK
TGDRYLKALRPLLNDNQFEEAEKRTSNFINNEGKVLQEKLISKDKRNKHTSYISDYWFDL
YLRDRAALPINYNPMIVFQNDVRPEYNDQLIRSTNILITAVRFMLSLREQILEPEVYHLNPK
KSDTQLYRTFTRMLPEAISWYGAYLMKVFPLDMSQFVGLFGATRLPRLNKDEIFRDPKSK
HVLVQKQGNFYVFDVLDTDGNLLSPLELLGNLNKIMNDKTPASEHPLGILTTQNRDEWAK
QRDHLEATGNSEVLRKIDSAIFNLILDDDDINDDKRVLLKKYLHSDGTNRWFDKSVSLIVT
RDGVGGVNFEHSWGDGVAVLRFFQDIYAETTKKPFIHPDSKPVDSNISVQKLEFKLDDKS
KHFIDNAKKEYKAWTDSLSIDYILYEGLNKAACKKFKVSPDCIMQLSFQAAHHLLKGNFV
GTYESCSTSAFKHGRTETMRPCTVKTKAFCETLHSNKSSIEELRGKLTECSKLHLELVKDA
AMGQGFDRHMFALMKMAEDNNMPRPEIFDSYEYKFLNKSILSTSTLSSPSVMAGGFGPV
VKEGYGIAYSAFPDKLGAAVASYKAHNNSTQYVEALHKSFLDITKILSG
>AGG55013.1 acyltransferase AGPAT5 [*Heliothis subflexa*]
MWYKIFIFTIVCVFTYILKKLHDTGPNRVKFYFNFFLFYFLSSMLAAVIWPYFLLSPKNVRN
AKIAVRLLKHITKLYDLKWHLRDGKILAEDRGAVIISNHQSSLDILGMFNIWEVVDKLAAI
AKKELFYVWPFGLSAYLAGVVYIDRRNAKGAYKQLKITSEVMVKNKTKIWLFPEGTRNK
DYTKLQPFKKGAFNIAVAAQVPIIPVVFSPYYFINKEKYIFNKGHVIIQCLEPVPTVGLTMED
VPDLIDRVHQKMSVAYQEISKEVFSSLPADYPVTLVG
>AIN34693.1 fatty alcohol acetyltransferase [*Agrotis segetum*]
MLEHLSYAMEIVTKIFSQISALLGIQWAPMDIPMSRRLQTLAAFVWIYLILFGEALSIYLFIQ
LVYSRFWWMGILYGVWFLNDIEICSRGGRASEWVRNWTWWRYLCDYFPIKLVKTVELDP
SKNYMFACFPHGVISLGAFGSFCTNATGFHKLFPGMTCHLITLGGHFLVPFFRDLALALGI
CSSSEQSLLHLLDNKKYEGNCACMIIGGAAEALDAHPKEYKVILSRRKGFIRVAMKSGAA
LVPVFSFGETDLFRPPNNPENSLLRRFQEKVRQYTGISPMFPMGRGLFQCSYGVLPMRAPV
TTVVGAPMEVKRNLEPTNEEINAVHAEFTERLKTLFETEKVKYLQYHEEAKLVIT
>AIN34705.1 fatty alcohol acetyltransferase [*Agrotis segetum*]
MGARNLKVLQVISGWQAVELILTCVFVGIWQIIEISVKRLWKGHRRKIEDTSPVELTIDSSI
GTHCYIKVMGVKYHYVETGPRTGQKVLILKDAPDSGNLWGPNWASVVRRLAETDHHVV
TLDLRGTGGSEGGSRSDLAPPRAVEELSALLKALGVSENRQAVVIGFGVGGMLAWYLVHS
RGPLISKFAVINAPHPNLYWQYPPAPFCHRALQFIQWPHFPERWLAEGEMYDREGSWASSR
ACDWTGALNYVRGAAWWKIKPGLRTSAPALLVGHKDSAGQLVASAQYCTASTLRLVTKP
DPSSKELTGVLLDFLIAKEKLLEEQVPRGLMGRVFGAVADRGRELTARLVLPPMQA
>AIN34704.1 fatty alcohol acetyltransferase [*Agrotis segetum*]
MVFDNNWTGGWFSWTRQSDAMLRNVEKKILSCLKTAYKRFYVDIGSVVGQSDKIWTISL
NDESPKTPLVMLHGMGAGLALWCPNLDSFAATRPVYAIDLLGFGRSSRPKFASDAQKAEA
QWVESVEEWRREVNISQFILLGHSLGGYIATAYAIKYPERVRHLVLADPWGFSERPPNAYE
KAQLPLWVRAIATAVQPLNPLWAVRAAGPAGKWLVSKTRPDISRKYLNFLPDAERVIPEYI
YQCNSQTPSGEAAFHSLMTGFGWAKNPMVRRVDEIDPALPITVLYGSRSWVDNTTGQVL

AEHRGPTNTYVQVINGAGHHVYLDKPELFNKFVLEACARADAHDPRPSLAGASPSAIEAP
PSKLAIEAAPATSATSTESTGKVNISTEAQSS
>AIN34713.1 fatty alcohol acetyltransferase [*Agrotis segetum*]
MAKRLLSSLILNSSGTNLKSSLPVLSKKLHTQQVPTKEIQIPVKFGHLAGKLWGSGDQPPIL
ALHGWQDNAGTWDPLIPMIIKDRPILALDFPGHGFSSWIPPGMLYYQWELPRIILYLKEYF
KMEKVALLAHSMGAIAGMRFATVFPDDVEFYIAIDSLIYDDYDLDAVVDRISKTIRKGLLA
QSRLDKEPPLYTLEDMIKIWHAGTRKSVALESVPHLLKRGANQSKTDPSKYYFSRDSRLK
YSLFNPEDKKFVEALVRRLKCPTLYVKAIDSPYSADAYSIEMREILEQVNEKYEFHFVRGT
HHVHLNNPELVAPLIKNFIQKHNLTI
>AIN34683.1 fatty alcohol acetyltransferase [*Agrotis segetum*]
MAVAINKGIFIVAAKRTPFGRFGGAFKDVYPSDLLAAAAKDALKAGSIAPEVIDTVNIGQV
YGLSGSSDGGLSPRHAALKSGIPEDKPALGISRLCGSGFQAVVNSAQDIITGVAQTSLAGGT
ENMSTVPFVVRNTRFGVNLGVKMPFEDLLTASSLDTSCNNTMPETAENLAEKYGLHRME
VDQYALQSQQRWKAAQDQGAFKAEMTPVTVKVKRQDKVIEVDEHPRPETTTEMLSKLP
VLFRKGGVVTAGNSSGVNDGAGALVLASEESVKQNGFKPLVRLLGWSVVGVDPSIMGIG
PVPAIQNLLKVTGLKLDDIDMVEINEAFSAQTLACAKELGLDQSKLNINGGAIAMGHPVG
ASGARITAHLAHELRRRGLKRGIGSACIGGGQGIALLLETV
>AIN34694.1 fatty alcohol acetyltransferase [*Agrotis segetum*]
MMFGLLLNVLGLIGLSPIPFLSEVIGATEPALKLLISILLGYPLAVIYHKYVKHHKEYRNLYF
VLTGFDMAFYNFGISMYHNAIPAIVIYLSTKFLGPGKNNAIVTFAFNMTYLLAGYVVTESE
DYDITWTMPHCVLTLKLIALSFDLWDGKKMLKGEELSANNKLTALESQPSFLELLGFVYF
PACFLVGPIFSFRRYKDFISDKFPLEREVKVYEAQAVKRLVQGVIYLAAYQIGVTVFSMKY
MLSDEFWDNSVFYRNFYCGLWAHFALYKYISCWLLTEAACIRFGLSYNGSRTENGVSVSQ
WDGCNNIKLLRFEGATRFQHYIDSFNCNTNHFAAEYVYKRLRFLGNRNLSQLITLAFLAL
WHGTQSGYYMTFLNEFLIMVMEKDLESMLLKTEFYHKMWNNSIIKYLLYFILKMYTIVF
MGWSLAPFDVKSFSKWWTVYTSLYFSGFILFVPWSFVYKPLVKKALKASGAHPKAQ
>EFN73032.1 Membrane-bound O-acyltransferase domain-containing protein 2 [*Camponotus floridanus*]
MATTMDHYDGFRTFSWFADFVGLPIDQVNFVLTQFTALILAGLFRSSLSPIAATPAARHVY
GLIIGLALGYFCFGRQAIHLASLPALCYVAMRTQNPRNMQRVVLAIALIYLSCIHFQRQIYD
YGSYTLDITGPLMVITQKVTSLAYSIHDGLTRREEELTPMQRHQAVQKIPTTLEYFSYIFHF
QALMAGPIIFYRDYMDFIYGHHLPGSKSLTIFYDKNSQEKEIVLEPSPTLVVVKKVVASLAC
AIVFITFISDFPIQRVKEDEFLKNTSMLYKMWYLMLTTMLSRFKYYHAWLFADAICNNSGL
GFSGYDERGKPKWDLTSNVDVYKFETSLNLRDSIESWNKGTNLWLRSIMYERAGRNKVL
FTYALSAFWHGFYPGYYLTFASGAFFTVAARSVRRHIRPLFLESQRKKTFYDILTFITTRIV
MAYMTFSFILLEFMPSIKVYLYLYMFPHLLGLIAIILPPRLGLSKKAHKQSAEIDLSETISNG
NAHKTM
>ARD71199.1 acetyltransferase [*Spodoptera exigua*]
MLRSIFVRNQVLNDALKKSIRSNLSRCMSTELSKRKLSHRVLSAQNKRVSAAPQWTIQIRY
YADLPTHSKVNLPALSPTMESGSIINWQKKEGEKLSEGDLLCEIETDKATMGFETPEEGYL
AKILIPAGTKGVPVGQLLCIIVGNEADVAAFKDFKDDSPPGAVKPPVKKTAQAAAAPAASA
PAPAAPAAAPAAAPAAAAAPPPAAPAAAPDSGRVYASPMARRLAEIRNIRLGGQGSGLYGS
LKSGDLSDAPAAAEALAPPPMPAPGATFVDIPLSSMRATIAKRLSAAKQTIPHYQLTATVNV
EKTIEMRKKVNEKLAAEKAEVKVSMNDFIVKAVAAACKRVPTVNSHWMDSFIRQFNNVD

VSVAVATPAGLITPILFNCDSRGIIELSKNMKELAARAREGKLQPQEFQGGTVTVSNLGMY
GITMFNAIINPPQSLILACGGLQELVIPDKNEPQGFRLAKFVTFTASADHRVIDGAVGAQW
MKAFKENMEDPANMIL
>ARD71200.1 acetyltransferase [*Spodoptera exigua*]
MSANVILGCVMALVIILFTISSMARYYIKFTLFIVMALIFATAPVPLMLIKPFDPRNALIPAFF
LRCFARLLGLRWKVRGLENVDNSRGAVVLLNHQSSLDLYALAIIWPLMSRCTVVSKRSLQ
YLVPFGTATWLWGTVFIDRGAKSARDALNKQVDAIKDQKRKLLLFPEGTRHCGDRLLPFR
KGAFHVAMDAGAPIQPVVISKYHYLDGKRHKFGSGEFIVSFLPTIETEGLTKDDIPTLVEKT
QLSMQEEFTKISMETLERRNRLKAN
>ARD71201.1 acetyltransferase [*Spodoptera exigua*]
MSISSVMPRFHIPCCSIWTHYLMPFIYHVIIGFLSWTGGWFSWTRQSDAMLRNIEKQILSCL
KTAYKRFYVDIGSVVGQSDKIWTISLNEESPKTPLVLLHGMGAGLALWCPNLDSFAATRPV
YAIDLLGFGRSSRPKFASDAQKAEAQWVESVEEWRREVNLGQFILLGHSLGGYIATAYAM
KYPERVRHLVLADPWGFAERPPNAYEKAQLPLWVRVIGSALQPLNPLWAVRAAGPAGKW
LVSKTRPDISRKYLNYVPDAERVIPEYIYQCNSQTPSGESAFHTLMTGFGWAKNPMVRRV
NELDPALPITVLYGSRSWVDNSSGQVLVEQRGPTNTYVQVINGAGHHVYLDKPELFNKFV
LEACTRADEHDPRPALKAAPAEPGTETPALPPGGEAPSNTVAITNKASASSDTTAHTS
>ARD71203.1 acetyltransferase [*Spodoptera exigua*]
MAMRIVVLSILFFIVPILCYKPVVLIHGVMTGSASMELIKLRIEEQHPGTIVYNVNRFESWS
SLETMWHQVLEIGMDIANISSKHPEGINLIGYSQGGLIARGIVETFPNVSVSTFISLSSPQAG
QYGAGFLHLVFPGLVKDTVYELFYSRVGQHTSVGNYWNDPYHQSLYESYSVYLPYINNH
LPSAKSADFKKNLLRLKRLVLIGGPDDNVITPWQSSQFGYYNANETIIEMKAQDIYVEDRI
GLRTLDETGRLHVVTVPGINHFNWHMNMSIVDNYLLPYLD
>ARD71204.1 acetyltransferase [*Spodoptera exigua*]
MDLLEKEWYIQAPWGRIAIIAWGNCFDPPVLLCHGSVDSAVSFKPLISKLPKNFYYIGVDL
PGNGKSDRMLPGLMISVTDMAYAINAVAKHFRWKKFTFIGHSFGAFLGQMFNILYPGRLN
KLVNLDPINFFATPPQDFGKWYHSRFTNFYKNYEKFNTPVENGPRVKWTEALQSLRSNRP
SLSEENAIAVLERLSEPAGDGYIRYTYDLRIKCMHFPAFSPEHVKKLFTNHDTPILTVACKN
SLDKKLFRNTAFLLDEAEYPSGNLRFRSVAGGHDVHISNPDRVAVYVSQFLLYGLEGMDN
KAKL
>ARD71205.1 acetyltransferase [*Spodoptera exigua*]
MTYYNYYDGSRIFLFLSTRIGLPLDLVNFLIAQVAALCLARLFRKPLRYASPEFRHSVCLVI
GLTMGYFCFGRQAIHLSVLPMLTYTMLKSVSHKIMGNVILAVSIIYLSCIHLHRQIYHTADY
TLDITGPLMVITQRVTSLAYSLQDSLTVKEKPTSGNTSEANGDLVKIEKIPSPLEYFAFTLAF
QTLMCGPVVFYSDYIKFIEGARIDEFEKSKDATEPSPRRAVFYKVCGSLAAALLYLTLAKK
YPLAVLEELTDPSSEVSRSWSALYLLWYAYLSTLVVRCKYYHAWLLSEAICNNCGMGFNG
YNKDGSPKWDKMSNIDIFGFEFAQNFRVAIASWNKNTNAWLRDVAYERGGAAWRTARVY
ALSAVWHGFHPGYYLTFFAGGLFTIAARKIRYAARPMFLDSAPKKLFYDCVTFLTTRVAMT
YATVPFVLLHLAPSLAFYGKFYYSLHFIALGALLIPEKPKRSKSAQIQEKGSYKSSEETLPIL
ETLDSSHGKLKIT
>ARD71206.1 acetyltransferase [*Spodoptera exigua*]
MALIMSFVSVAISILYTPLLLLILCIIFLASIGKSLGVRRLYVNILLKLFEYGRQHIEVAKIKIQ
RTDSSDEEDLPPVPDDKPPSAIIKENGVNGTKMTVIERQEILGPSPELNYKRSTSQERVQNG
PKTTQGNGESNMEFDLSNCLDLVKAGMESIIEDQVTSVFEAEELRSWNLLTRTNRQYEFLT

WRLTIIWAMGFVVRYMFLLPLRIMIFVIGVWWLIACTACIGTLPDGKTKQRINYAVSVMCF
NFLSRCISAVITYHDAHYKPKNGICVANHTSPIDALVLMCDNCYSLIGQRHNGFLGILQRAL
ARASPHIWFERSEVKDRHAVAKRLKEHISIPDNPPILIFPEGTCINNTSVMQFKKGSFEVGG
TIYPVAIKYDPRFGDAFWNSSRYGMLHYLLNMMTSWAIVCDVWYLPAMTRAADESAVDF
ANRVKAVIARRGGLVDLMWDGQLKRMKPKKEWRELQQEEISKRLKGE
>ARD71207.1 acetyltransferase [*Spodoptera exigua*]
MSFLMRKCIVNLKNFNRCRTVCVLLQTERQLSRYSSNILNRSILLSEVHLRHRKFHTSQILN
KVVAFKLSDIGEGIREVVIKEWFVKVGDKVQQFDNICEVQSDKAAVTITSRYDGVVTKLY
HDVDQTALVGQPLVDIEVQGASDEASSDSNEKPAAASQQEQKADKPQRVKVLTTPSVRRI
AAQFKVDLSTVKATGRNGRVLKEDMLAHLNIDSDGSNKVSDPTSVDAVQIPMTSAQAKV
EVLLEDRVVPVSGFTKAMVKSMTEAMKIPHFGYSDEYDVSKLVESRESLKNIALSRGVKL
TYMPIIIKAASLGLENIPILNSSLDSTCEHLTYKASHNIGVAMDTPNGLVVPVIKNVQNKTIL
EIARELNTLQEKGSKGQLGLSELSGGTFTLSNIGIVGGTYTKPVILPPQVAIGALGKIQVLPR
FDEEGNVRKAHILTVSFSADHRVIDGVTMARFSNHLKNYLENPYTLLLDL
>ARD71208.1 acetyltransferase, partial [*Spodoptera exigua*]
MFIHNYFGFEDMCLTHTHQIGMDMQLYVATLPLMLVIWKYSTLGWSLLALIAMASTVLR
YLAIYWYDISMFVYYGISVQKLLDAARYSYILPTHRATIYLIGVVMAYLMKTKKLNFTLTS
NQTRLLWTFCLVLMTATIATPYKWGLEGYQYEHFPAAMFSALSPILWGVFMSVSHWAIVN
DYAGIGTAFLESRVFKFFNKIAYSVYLTQFPIFFYNVGVQRHAEFYSPLLLLHIPEVFTVTAIS
ILTTVAIEMPFNQVYRIY
>ARD71209.1 acetyltransferase [*Spodoptera exigua*]
MKQSGIILLTVLVVQAYSAPQFITFSEGKLGVNFGGYHAGVGLGGLAGGKGNTAGGLYAE
AGTPFGPAAKAGLGGAVDGSSGTAGGLYAGATAGGNVNAAAGLGGAVAGGKAIGGGYST
AQSGGHTATSVLGGESGASGSAGFSVSAHKSVEVPVTVVKETEISVIPVEEVKTVHKKVY
GEAKYEASNEITPVAKAGVEATANVNVNAQPEFVKEVSNWNRPIYSAPIIPPVFFQSIFSSL
FGSPQRSYPPPMWLPPLSYNFKQVCRVIIIKWYVHPEA
>ARD71210.1 acetyltransferase [*Spodoptera exigua*]
MSSKRPLTKAQKQQYEKKYEKRSLYIPKKYFVIAIIFLLAASSKLYFFKTDCVIPNVDFEQW
WGSYPKTEIDTSIRPFMIEFSDIKVNDLKERLLHRAQFAPPLDSAGFSYGFNSLFLPKVLDF
WQKEYNFEERERFLNKYNHFVTGIQGLDVHYMHVKPDLGVGDDITVLPILLIHGWPGSIR
EFYELIPKLVTPRPNQKFVFEVIAPSIPGFGYSQAPVQQGMGPQEVAVVFYNLMKRLGFTK
YYVQGGNYGAKIGSVMATLFPDTVLGFHTNTPTIMWSPMAIFYTLFGTIWPSFIVEPTLAD
RMYPLSQYLRTIIQETGHFHLQATKPDTVGIALSDSPAGLAAYILEKFSAWTDVDNKQAID
GALLHKFSLTHLLDNVMIYWTTNSITSSMRHYTEYKQLWVLDRIPTDVPTWGIKFKYNLC
FQPDSILRLKYKNYLHSSIVEDGGHFAAMEMPDVLADDIFDAVDTFIRFHEEKKKNEPQPE
PAESKTAETVSAKKSTEPVKKTEPAKKPTEVDYMKAKSVHEFTVKDIHGNEVKLDRYKG
QVLIIVNVASNCGYTNVHYKQLNELYEKYSGKGLRILAFPCNQFAYQEPGSPEEILKFTKA
KQVKFDLFEKVAVNGEDAHPLWNFLKRMQGGTLGDFVKWNFSKFIVDKNGVPVERFGP
NTDPLELVPYLEKLFDQ
>ARD71211.1 acetyltransferase [*Spodoptera exigua*]
MAKRLLCRTILNSNTTTIKSSLPVLSKKLHSQVPTKEIQIPVKFGHIAGKLWGNSNERPILA
LHGWQDNAGTWDPLIPMIIKDRPILALDFPGHGFSSWIPPDMQYYQWELPRIILYLKEYFK
MEKVSILSHSMGAIASMRFASVFPDDVDFYIAVDSLIYDDYDLDAVVSKISKTMKKALIAQ
TRLNDEPPGYTLEDITKIWHLGTRKSVALESVQHLLKRGIKPTKADPNKYYFSRDSRLKYT

LFNPEDKKFVEALVRRLKCPTLYIKAIDSPYSADAYSIEMREILEQNNENYEFHFVPGTHHV
HLNNPELVAPLIKNFIRNHNLSL
>ARD71212.1 acetyltransferase [*Spodoptera exigua*]
MSVAAKGIFIVGAKRTAFGTFGGVFRNTTATELQTAATVAALKEAGVAPEKVDSVVVGQV
MTASQTDGIFIPRHVMLKAGIPQDKPALGVNRLCGSGFQSVVNSAQDILTGAAKISVAGGV
ENMSQAPFAVRNVRFGTALGSTYAFEDTLWAGLTDSYCGLPMGMTAEKLGAQFGITRDEV
DNFALRSQQRWKAAQDAGVFKAEITPVTLTVKRKEVKVEVDEHPRPQTTIEGLKKLPPVF
KKEGLVTAGTASGISDGAGAIVLAGEEAAKGLKPLARLVGWSYVGVDPSIMGVGPVPAIE
NLLKATKMSLNDIDLIEINEAFCAQTLSCAKALKLDMEKLNVNGGATALGHPLGASGSRIT
AHLVHELRRRGLKRGIGSACIGGGQGIALMVETV
>ARD71213.1 acetyltransferase [*Spodoptera exigua*]
MAPSNLSLNEVVIVSAVRTPIGSFRGSLANVTATELGAIVVRAAVERAGIPSSEVKEVFMGN
VCSAGLGQNPARQAAIFGGLEKSTICTTVNKVCASGLKAVTLAVQGLQTGANDVILAGG
MESMSNIPFYIRRGEIPYGGTQLLDGILYDGLTDVYDQIHMGDCAENTAKNLNLSRKQQD
DYAIISYKRSAAAHAAKAFDAEVVPVPVPQKKGGAPVIFAEDEEYKRVDFDKLVKLPTVF
KKENGTVTAGNASALNDGAAAVVMMTAEAAKRLNVKPLARVIGYADGEREPIDFPIAPS
VAIPKLLEKTGVKKEDVAMYEINEAFSVVTLGNQKLLGIDLEKINVHGGAVSLGHPIGMSG
TRIVGHLCHALKKGEIGVATACNGGGGASAIMIEKL
>ARD71214.1 acetyltransferase [*Spodoptera exigua*]
MELQDTYYNKSEYVETASGNKVSRQTVLCGSQNIVLHGKVIVQSDAIIRGDLANVKTGRF
CIISKGSVIRPPFKKFSKGVAFFPLQMGDHVFVGENTVVNAAVVGSYVYIGKNVVIGRRCV
LKDCCMIEDNSVLPAETVVPSFARYSGSPARLITTLPEAMPDLMTEFTKSYYQHFLPTTVQ
>ARD71215.1 acetyltransferase [*Spodoptera exigua*]
MFGLLLTLLGWVGLSPVPFLAGVLGATEPALKLLISILLAYPLAIVYHKHVRQHVEYRNLY
FIATGLDMAYYNFGFSMYHNAIPAIVIYLTTKFLGPGKNNTIITFAFNMTYLVAGYVVTESE
DYDITWTMPHCVLTLKLIALSFDLWDGKKMLKGQELSANNKLTALESSPTFLELIGFVYFP
ACFLVGPMFSFRRYKDYITDKFPLDKEKDVYEAQAIKRLIQGLVYLIAYQVGVTVFSMKY
MMSDEFRETSVFYRHFYCGLWAHFALYKYISCWLLTEASCIRFGLSYNGVETKRYPQVSK
WDGCNNIKLLRFEGATRFQHYIDSFNCNTNYFAAEYVYKRLRFLGNRNLSQLITLAFLAL
WHGTQSGYYMTFFNEFIIMVMEKDVEVMLTKTQFYHKMWDNTILKYLLYIILKTYTIVF
MGWSLAPFDAKSFSKWWSIYHSLYYSGFVLFLPWAFVYKPLLKSGLKSLEKGTNHQQ
>ARD71216.1 acetyltransferase [*Spodoptera exigua*]
MFLLKGSSIITIKMRPTNKLFKAMAAYSSKVSLNDVVIASAVRTPMGSFRGSLASLSASEL
GAVAVKAAVERAGVPKEEIKEVYMGNVCSASMGQAPARQAVIFAGLPKSTICTTVNKVCA
SGMKSIMLATQGLQIGSQDVILAGGMESMSNVPFYMKRGDTPYGGIQLIDGIVFDGLTDV
YNKFHMGNCAENTAKKLNISRQQQDDYAISSYKRSAAAYEAKAFAEELVPVPVPQKRGA
PPVMFAEDEEYKKINFEKFTKLSTVFQKENGTVTAGNASTLNDGAAAMVLMTAEAAQRL
NIKPIARVVGYADGECDPIDFPIAPAVAIPKLLEKTGVKKDDVAMWEINEAFSVVAVANQKL
LELDPAKVNIHGGAVSLGHPIGMSGARIVVHLCHALKKGEKGVASICNGGGGASSIMIEKL
>ARD71217.1 acetyltransferase [*Spodoptera exigua*]
MINLGILKQSTIVHLCFAISYFTSGLILTFIQAILYFGLRPFNKSLYRKINYYLAYSFYSQLVF
MSEWWSNSKLTIYIKKDEYEKYYGKEHGYLIMNHSYEIDWLMGWHFCNTIGVLGNCKA
YAKKSIQYLPPIGWMWKFSEFVFLERSFEKDKETIKHQISELCDYPDPVWLLMTPEGTRYT
KKKHEASLNFAKEKNLPLLKHHLTPRTRGFTTSLQFFRGKIPVIYNIQLAFEKDSKTPPTLTS

LLYGKPVNAHLYIERIPVENVPEDEGEAAKWLHELFVVKDKMQDSFFNTGDFFLESGVER
RESFTVPPPIWSLVNALGWAVVTLTPMLYYLLGLLFSGKLLYFSIACAIFGAFFILLQKSIGM
SKISQGSSYGTEKK
>ARD71218.1 acetyltransferase [*Spodoptera exigua*]
MNIRCARPSDLMNMQHCNLLCLPENYQMKYYFYHGLSWPQLSYVAEDEKGHIVGYVLA
KMEEDGEDNRHGHITSLAVKRSHRRLGLAQKLMNQASLAMVECFKAKYVSLHVRKSNR
AALNLYTNSLGFKILEIEPKYYADGEDAYSMMRDLSAFAAENKTEPQPTENLEIKSESAIIS
QC
>ARD71219.1 acetyltransferase [*Spodoptera exigua*]
MIGANKLICKSNVCQKIIQQRNFTRKNIKDVNYQYLQRSKLPTMHFQKSLPRLPIPELSKTS
ERYLNALRPLLTTKQFEEAQQRTNNFIAKEGKVLQEKLIAKDKRNKHTSYISDYWFDLYL
RDRVPLPINYNPMIVFQNDVRPEYNDQLIRSTNMLISAVRFMLSLREQILEPEVYHMNPKK
SDTPLFRNITRMLPEAISWYGAYLFKVFPLDMSQFVGLFGATRLPRQNKDEIFRDPKSKHV
VVQRRGNFYVFDVLDADGNLLSPQEILGNLSKVMNDNSPISEYPLGVLTTQNRDQWAQQ
RVHLESTGNSEILRKIDSAIFNLVLDDDVINDDKRVLLRKYLHSDGTNRWFDKSFSLIVTGD
GVAGVNFEHSWGDGVAVLRFFQDIYAETTKKPFIHPESKPADSNISVQKLEFKLDDKSKQFI
DNAKIEYNAWCDSLSIDYILYEGLNKAACKKFKVSPDCIMQLSFQAAHHLLKGSFVGTYE
SCSTSAFKHGRTETMRPCTVKTKAFCETLHSNNRSDDDLRSKLTECSKLHLELVKEAAMG
QGFDRHMFALMKMAEDNNMPRPEIFDSYEYKYLNKSILSTSTLSSPSVMAGGFGPVVKE
GFGIAYSAFPDKLGAAVASYKSHNNSSHYVEALHKSFLDITKILSA
>ARD71220.1 acetyltransferase [*Spodoptera exigua*]
MAVVINKGIFIVAAKRTPFGRFGGAFKDIYPSDLLAVAAKDALKAGSVAPEIIDTVNIGQVY
GLSGSSDGGLSPRHAALKAGIPQEKPALGISRLCGSGFQAVVNSAQDIITGAAQTSLAGGT
ENMSTVPFVVRNTRFGVNLGVKVPFEDVLTTSSLDTSCNFTMPQTAENLAEKYGLQRME
VDQFALQSQQRWKAAHDQGVFKAEMAPVTVKVKKQDKVVEVDEHPRPETTTEMLSRLP
VLFRKGGVVTAGNSSGVNDGAGAIVLASEESVKQNGFTPLVRLLAWSAVGVDPSIMGIGP
VPAIQNILSATGLKLDDIDLIEINEAFAAQTLACAKELGLDQSKLNVNGGAIAMGHPVGAS
GARITAHLAHELRRRGLKRGIGSACIGGGQGIALLLETV
>ARD71221.1 acetyltransferase, partial [*Spodoptera exigua*]
MESKTTKMPKVAKVKNKAPAEIQITAEQLLREAKERDLEILPPPPKQKISDPEELRDYQHR
KRKAFEDNIRKNRLVIGNWLKYAQWEESQKQVQRARSIYERALDVDHRNVTLWLKYTE
MEMRNRQVNHARNLWDRAVTILPRVSQFWYKYTYMEEMLENVAGARQVFERWMEWQ
PDEQAWQTYINFELRYKELDRARQIYERFVMVHPDVKNWIKYARFEENHGFINGARKVFE
RAVEFFGDEDLDERLFIAFAKFEENQKEHDRARVIYKYALDHIPKDRNKELYKAYTIHEKK
YGDRSGIEDVIVNKRKYMYEQEVIENPTNYDAWFDYIRLVENEGNVDDIRDTYERAIANV
PPSKDKQFWRRYIYLWINYALYEELEAEDTERTRQVYRTCLELIPHKIFTFSKIWLMYAQFE
VRCKDLKQARKTLGMALGICPRDKLYRGYIDMEIQLREFDRCRILYQKFLEYGPENCITWI
KFAELETLLGDTDRARAIYEIAVGQPRLDMPELLWKSYIDFEVQQGETEKARQLYERLLER
TVHVKVWLSYAKFELNAENPDDINVDLARRVYERANDSLRSAGEKEARVLLLEAWKDFE
TEIGEEEKLEKVLSKMPRRVKKRQKIISESGVEEGWEEVFDYIFPEDEMVRPNLKLLAAAK
QWRKQKEVLQPAESETKTNQEETKEDDNNDDDDDNNSEEEQTPPQPQEQNEKED
>ARD71222.1 acetyltransferase [*Spodoptera exigua*]
MGARSLKVLQVISGWQAVELILTCVFVGIWQIIELSVKRLWKGYRRKVDDNQPVELTVDS
SIGTHCYIKVMGVKYHYVETGPRSGQKVLILKDAPDTGNLWGPNWANVVRRLAETNHH

VVTLDLRGTGGSEGGSRSDLSPPRAVEELSALLKALGVSENRQAVVIGFGIGGMLTWYLV
HTRGSLISKFAVINAPHPNLYWQYPPATFCHRALQFIQWPHFPERWLAEGELNDREGRWTS
SRACDWSGALNYVRGAAWWQVKPGLRTSAPALLVGNKDSAAQLVASAQHCTASTLRLV
SKPEPSSKEVTDVLLDFLIEKEKLIEEVPRGLMGRVFGAVADRGRELTARLVLPPTQA
>ARD71223.1 acetyltransferase [*Spodoptera exigua*]
MASKRINTFKVVFVSLAVVAVGFIIRTPWLPIRRELKASLGYPRDSLLNFTELTAEYGYVSE
EHEVVTEDGYILTMFRIVQARNCHQKKRSPPVLLVHGLLQSSDSFIDSGPNAGLAYLISDA
CYDLWLGNVRGNYYSRGHTRLDPNKDPKYWKFYIDEIGYYDIPAMIDHVLDYTGNDKL
NYIGFSQGSGTFLVMCSERPSYCEKVQLLIGLAPAARQFNTKSKLFRTLTQTFEVLEGPLEN
YGLVEVFSKGAVSQEFVAFFCQLSHFTGKLCEQVLDVFDYVDSSHLGSITNETTRVLFGHF
PAGTSLHNMARYGQSMKSKRFEKFNYGKEKNLVMYGSEEPPRYNLSAVTAPVVCIYGSN
DGLVDTKDVEWLVGKIPNVIESIKVEDPLWNHMDVTYSQYTSDTIFPKINEYLLKYTSA
>ARD71224.1 acetyltransferase [*Spodoptera exigua*]
MIENLSSIVEALSKSFSQISTLLGIQWAPMDIPMSRRLQTFAAFLWIYLILFGEAFAIYLFIRL
VYSKYWWAALLYGAWMLNDIEICNRGGRSSEWVRSWIWWRYLADYFPIKLVKTVDLDP
SKNYMFACFPHGVISLGAFGSFCTNATDFKKLFPGMTCHLITLGGHFLVPLFRDLALALGI
CSSSEQSLLYLLDKKKYEGNCACMIIGGAAEALDAHPKEYKVILNRRKGFIRVAMKSGAA
LVPVFSFGETDIFRPPNNPENSLLRRFQEKVRQLTGISPMFPMGRGVFQYSYGVLPIRAPVT
TVVGAPMEVKRNLEPTNEEIDAVHAEFTERLQTLFETEKKKYLKYYEEARLVIT
>ARD71225.1 acetyltransferase [*Spodoptera exigua*]
MLGIAPVLAVKSLLKRTGLTMNNMDLVELHETFAAATVACIRELDVDDDKLNVNGGAIAI
GHPPAATGARIVTNLTHELRRRGLKRALTAGSIAGGQSIAMIIEAV
>ARD71226.1 acetyltransferase [*Spodoptera exigua*]
MKTLFILLFVIKFISSKPTSIVLWHGMGDTCCVSFSLGGIKVFLENNIPGVYVTSLRIGNSTV
EDFENGYFMNPNYQVEYVCKQLAADPNLKDGFNAIGFSQGSQFLRAVVQRCGHILPPIKN
LISLGGQHQGVYGLPHCGALMHPTCDYIRQVLNYAAYDSWVQDALVQATYWHDPLDEE
TYINKSIFLAEINNELRVNKTYIENLNNLQHFVLVKFDNDTIVQPRETEWFGFYDPGQSKK
VVPFYETRLYVEDRLGLRKMHKDGRLVLISTEGDHLRFSDKWLVETIIKPYLLN
>ARD71227.1 acetyltransferase [*Spodoptera exigua*]
MAFAGLKKQINKANQYVTEKMGGAEGTKLDLDFVEMERKTDVTCELVEELQTKTKEFL
QPNPTARAKMAAVKGISKLSGQAKSNTYPQPEGVLGDCMLLYGKKLGEDTVFSNCLIEM
GEALKQMADVKYSLDDNIKQNFLEPLHHLQTKDLKEVMHHRKKLQGRRLDFDCKRRRQ
AKGAHIADDEIRQAEEKFAESLQLAQIGMFNLLDNDVEQVAQLTFFAESLLEYHQQCTEIL
KGLVSTLMEKKEEAVNRPKMEFVPKTLADLHIEGIHDLNNGRRYGSTQSLSRPRQHIPPSS
SVGDLSTTDPFKAWEAPSPVRTQVRPAPGFKPHPAPRNQFNGRDPWTASPLPSPVKSPART
PVVANKTPCCTALYDFEPENQGELGFKENDVITLINKVDDNWFEGSVHGKTGYFPISYVQ
VTVPLPNM
>AIN34707.1 fatty alcohol acetyltransferase [*Agrotis segetum*]
MRATIAKRLSAAKQTIPHYQLTATVNVEKTMAMRKTVNEKLEAEKAGVKVSMNDFIVKA
VAAACKRVPTVNSHWMDSFIRQFANVDVSVAVATPSGLITPILFNCDSRGIIDLSTNMKELA
AKAREGKLQPNEFMGGTVTVSNLGMYGITMFNAIINPPQSLILACGGLQELVIPDKEDPRG
FRSAKFVTFTASADHRVIDGAVGAQWMKAFKENMEDPANMIL
>AIN34695.1 fatty alcohol acetyltransferase [*Agrotis segetum*]
MGKNPVLFLPTHRSYADFCLMTYLCYHFDIDFPAVAAGMDFYSMAVIGRRMRETCAFYIR

RTLAGDPLYAATLKQYVRTVVGKHAAPIEFFLEGTRSRSNKSMPPKYGMLSMTLVPLFAH
EVSDITIVPVNISYDRVMEHSLFAYEHLGVPKPKESTGGFLKALHSLNDHFGNIYINLGSPL
SVREYLKNDTSHSKETLKPLDIQQLTPEQFKKVQSIADYVISLQQKNTVATISNLLSLVLMQ
SLMKDSPLEFEEVVQEVGWMVQELRNLGATVFENDVRSSVERILVVQKKMMRLDKERK
LRLISGVLTDLSVDVKKKMKGHILQPQTMVAAVPIVQLQLYVNPILHYLVPPAIICLIVHRS
AVTRDNLEVDYHRVRKLLSHEFFHLEREEVNTFNKALDYCMQNGVITYSSELYTLGEDTK
LQYLLKWSVLPALTTLLKCAEVMTEQTNCAHKQALKLVQQRVESERVHPYCLSLEATAN
CLSGLVAAHALVKHKGESDVIYDLVPTTMLECSNLVNSILPSFNVDFERNSVVIDHKELSR
L
>AIN34684.1 fatty alcohol acetyltransferase [*Agrotis segetum*]
MTGSVSMEMIKLRIEEQHPGTIVYNVNRFESWSSLETMWHQVLEIGMDIANISAKHPDGI
NLIGYSQGGLIARGIVETFPNVSVSTFISLSSPQAGQYGAGFLHLVFPGLVKDTAYELFYSR
VGQHTSVGNYWNDPYHQSLYESYSVFLPYINNHILSAKSADFKNNLLRLKRLVLIGGPDD
NVITPWQSSQFGYYDANETIIEMKGQDIYMEDKIGLRTLDESGRLHIVTVPGVNHFSWHM
NISIVDDCLLPFLD
>AIN34711.1 fatty alcohol acetyltransferase [*Agrotis segetum*]
MDLTEHEWYIQAPWGRIAIIAWGDCYDPPVLLVHGSMDSAVSFRPLVSKLPKNFYYIGMD
LPGNGKSDRFLPGLMISVYDMVYSVHAVVKHFRWKTYTLIGHSFGAYLGQFYNLCYPGR
LDKLVNLDPINFFAVPPEEFGRWYHVFFTDYYKNYDKFNTQPENAPKIKWTEALQSIKSSR
PSLTEEQAAAVLERLSMPAGDGYVKYTYDLRMKRVNGPAYSPEHIKQLFTTTKTPILTIAC
QKSLKRKLFRNTDFLLDEAEFPGRNLRFRTVDGTHDVHVSHPERVAAYVGQFLVYGLDGL
DNKAKL
>AIN34703.1 fatty alcohol acetyltransferase [*Agrotis segetum*]
MAMAHNPYINKVSFSAVFGMPWAVIGRSAILYTDSFLYLSGFLNAHNLLTDLEKKGTINLK
DRLIARWFRLFPLFMSLMLFCTYILPDLNNGPQWNLVVEEHSRVCEKNMWKSFLFIHNYF
GFEDMCLTHTHQIGMDMQLYVATLPLMVLIWKYKTLGWSLLALIAVASTALRYLAIYWY
DISMFVYYGISVQKLLDAARYSYILPTHRATIYLIGVAMAYLMKNKKLKFTLSTTQTRLLW
VFCFALMTATIATPYKWGLEGYKYENFGAALFASLTPILWGVFMCVSHWAIANDYAGIGT
KFIESRLFKFFNKIAYSVYLTQFPIFFYNVGVQRNPDYYSPLLLLYIPELLIVTVISILTTVAIE
MPFNQVYRIYFGQSQKKLKEK
>AIN34692.1 fatty alcohol acetyltransferase [*Agrotis segetum*]
MELQDTYYNKSEYVETASGNKVSRQTVLCGSQNIVLHGKVIVQSDAIIRGDLANVKTGRF
CIISKGSVIRPPFKKFSKGVAFFPLQMGDHVFVGENTVVNAAVVGSYVYIGKNVVIGRRCV
LKDCCMIEDNSVLPAETVVPSFARYSGSPARLITTLPEAMPDLMTEFTKSYYQHFLPTTVQ
>AIN34694.1 fatty alcohol acetyltransferase [*Agrotis segetum*]
MMFGLLLNVLGLIGLSPIPFLSEVIGATEPALKLLISILLGYPLAVIYHKYVKHHKEYRNLYF
VLTGFDMAFYNFGISMYHNAIPAIVIYLSTKFLGPGKNNAIVTFAFNMTYLLAGYVVTESE
DYDITWTMPHCVLTLKLIALSFDLWDGKKMLKGEELSANNKLTALESQPSFLELLGFVYF
PACFLVGPIFSFRRYKDFISDKFPLEREVKVYEAQAVKRLVQGVIYLAAYQIGVTVFSMKY
MLSDEFWDNSVFYRNFYCGLWAHFALYKYISCWLLTEAACIRFGLSYNGSRTENGVSVSQ
WDGCNNIKLLRFEGATRFQHYIDSFNCNTNHFAAEYVYKRLRFLGNRNLSQLITLAFLAL
WHGTQSGYYMTFLNEFLIMVMEKDLESMLLKTEFYHKMWNNSIIKYLLYFILKMYTIVF
MGWSLAPFDVKSFSKWWTVYTSLYFSGFILFVPWSFVYKPLVKKALKASGAHPKAQ
>EHJ65205.1 acetyltransferase 1 [*Danaus plexippus*]

MAVALNKGIFIVAAKRTPFGKMGGMLKDMRPADLLAGVAKDAFKAGNVSPAAIDTVNIGI
VNVLSGSPDGGLSPRHAALKAGVPQEKPALGVNRLCGSGFQAVINSAQDIITGSANVSLA
GGTENMSSVPFLVRNVRHGVPLGSNIEFEDTLFRQSLDTYCNHTMPQTAENLADQYNLTR
TEVDEFSFQSQRKWKAAQDSGVFKSELSPVTVRVKKQEVTMEVDEHPRPDTSLEALHKL
PVLFRKGGLVTAGNSSGVNDGAGALILASEEGLKNNNLKPLVRVLGWSCVGVDPSVMGI
GPVPAIQNLLKVANLTLKDIDLVEINEAFAAQTLSCAKALKLDMEKLNVNGGAIAIGHPLA
ASGARITAHLAHELRRRGLKRGIGSACIGGGQGIALLLEVV
>AIN34700.1 fatty alcohol acetyltransferase [*Agrotis segetum*]
MSSKRFNTLNVLLVSLSVVAVAYVIRTPWLPIKRETKASLGYPKDSLMNFTELTGKYGYISE
EHHVITDDGYILTMFRIVKATNCHKQKRSPPVLLMHGLLQSSDSWIDSGPNAGLAYLISDA
CYDLWLGNVRGNYYSRGHVHLNPDKDAAYWKFYIEEIGIYDVPAMIDYVLDYTGFEKLN
YIGFSQGTGTFLVMCSERPGYCDKAQLVIALAPAARNLNTKSMIFRTLTQTFAKIEGALSM
YGVQEVFSKGAFSQEFVAFFCQLSDFTERLCETIIDTFDHADFSHMGSITNETTRVLFGHFP
AGTSVHNMARYGQSTRSTTFKKFDYGKEQNLVVYGSEQPPLYNLSATTVPVLCIYGNND
GLVDTKDVEWLMSKLPNVLESVKVKDPLWNHLDVTYSQYTVGSIFPKINEYLLKYTSA
>AIN34696.1 fatty alcohol acetyltransferase [*Agrotis segetum*]
MTRDEHPQPDVTLEKLSRLQPVSTGGITTAGNITGLNDGAAAMILANGQALRDHNLKPLA
RIVGWSVVGVDPVMMGYAAVPAVETLLKTTGLTIDDMDLVEIHETFAATTVVCARHLGV
DEDKMNVNGGAIAMGHPSGASGARIVSHLTHELRRRGLKRGIASAGIAGGQGIAIIIETV
>AIN34691.1 fatty alcohol acetyltransferase [*Agrotis segetum*]
MKTLFIFLFVIKLISAKPTSIVLWHGMGDTCCVSFSLGGFKLFLEKAIPGVYVDSLQIGNSTI
EDLENGYFLNPNTQVEKVCKYLAEHPKLKDGFNAIGFSQGSQFMRAVVQRCGHTLPTIKN
LISMGGQHQGVYGLPHCGALMHPTCDYIRQLLNYAAYDTWVQHALVQATYWHDPLDEE
TYIHKTIFLPDINNEVFVNKTYIQNLNNLEHFVLVKFDNDTIVQPRETEWFGFYEPGQSKK
MLPMQETRVYKEDRLGLKKMEKEGKLVLISTEGDHLRFSDKWFIENIIKPYLLN
>AIN34699.1 fatty alcohol acetyltransferase [*Agrotis segetum*]
MAFAGLKKQINKANQYVTEKMGGAEGTKLDLDFVEMERKTDVTCELVEELQAKTKEFL
QPNPTARAKMAAVKGISKLSGQAKSNTYPQPEGVLGDCMLLYGKKLGEDTVFSNCLIEM
GEALKQMADVKYSLDDNIKQNFLEPLHHLQTKDLKEVMHHRKKLQGRRLDFDCKRRRQ
AKGAHIADDEIRQAEEKFAESLQLAQIGMFNLLDNDVEQVAQLTYFAESLLEYHQQCTEIL
KGLVATLMEKKEEAVNRPKMEFVPKTLADLHIEGIHDLNNGRRYGSTQSLSRPRQHIPPSS
SVGDLSNTDPFTAWEAPPAYRAQARPAQTRPAPGFKPHPAPRNQINGRDPWKASPLPSPVK
SPARTPVAPNKTPCCTALYDFEAENQGELGFKENDVITLINKVDDNWFEGSVHGKTGYFPI
SYVQVTVPLPNM
>XP_022830278.1 N-alpha-acetyltransferase 60 [*Spodoptera litura*]
MAGFSWYLSEGFQVIIEKSKDAKCSLKDIQLRFLCPDDLEEVRSLCRDWFPIEYPQSWYED
ITSSERFFALAAVHKSEIIGLIVAEIKPYLKLNAEDRGILSRWFASKDTLVAYILSLGVARSFR
RSGVATMLLDVLINHLAGPVPQPPHEHRVKAIFLHVLTTNTEAILFYEHRRFRLHSFLPYYY
SIKGRCKDGFTYVYYVNGGHAPWGLYDYVKYVARAAWRGGGLYPWLWTKLRTALTIAW
HRNSHKT
>XP_021195034.2 N-alpha-acetyltransferase 60 [*Helicoverpa armigera*]
MAGFSWYLSEGFQVIIEKSKDAKCSLKDIQLRFLCPDDLEEVRSLCRDWFPIEYPQSWYED
ITSSERFFALAAVHKSEIIGLIVAEIKPYLKLNAEDRGILSRWFASKDTLVAYILSLGVARSFR
RSGVATMLLDVLINHLAGPVPQPPHEHRVKAIFLHVLTTNTEAILFYEHRRFRLHSFLPYYY

SIKGRCKDGFTYVYYVNGGHAPWGLYDYVKYVARAAWRGGGLYPWLWGKLRTALTIA
WHRRFSSTTTRVGAVRSDGEAI
>XP_047026493.1 N-alpha-acetyltransferase 60 [*Helicoverpa zea*]
MAGFSWYLSEGFQVIIEKSKDAKCSLKDIQLRFLCPDDLEEVRSLCRDWFPIEYPQSWYED
ITSSERFFALAAVHKSEIIGLIVAEIKPYLKLNAEDRGILSRWFASKDTLVAYILSLGVARSFR
RSGVATMLLDVLINHLAGPVPQPPHEHRVKAIFLHVLTTNTEAILFYEHRRFRLHSFLPYYY
SIKGRCKDGFTYVYYVNGGHAPWGLYDYVKYVARAAWRGGGLYPWLWGKLRTALTIA
WHRRFSSSTTRVGAVRSDGEAI
>XP_022817604.1 N-acetyltransferase 6 [*Spodoptera litura*]
MDAEDLQVLRLHENPQYLKPCCELINDEWPRSKTARMMSLQASCNNLPTSLILVNDKKH
LLGHCKLTPIPSIPESCFIETVVISKSMRGKKLGSYLMRQVEEYCKNVLNLKMLHLSTKGQ
ENFYAKLGYEVCPPVSIYGTRILNSEPVDISIKIQNPVPVSNSVKSGPPPPPPPPMPKPESLVQ
SSTVKSKKTFMFKYL
>XP_022821905.1 N-alpha-acetyltransferase 35, NatC auxiliary subunit isoform X1 [*Spodoptera litura*]
MGDNDDYYDGDGRMDSLGATPEVIYNWVDITSDFFKHIQDLQLGELLHDGHLFGLFEAM
SAIEMMDPKMDAGMLCNRGNPKPLNFQQAVAAGKLKIDDLEPPELIGIIDATMACIVSWL
EGHSLAQTVFTNLYLHQPHSIVNKTLKAYCIAVYKLLDCIRDCINKAQVFEEEDFQPMGYG
YRLGSNPQSGNTYDARLEVSEQKCVLMLREQEEELNKKARSCDDEDNLWAALQARIRFT
RMFYRALLMITKRDSQSGADCVALLNGCSEMMKVIIKTSGKGTQPVENSDSPNPMGFEP
MINQRLLPPTFPRYTRIKPRAEALHYFDELVARLRHAWKITSCTNFHTALDFFMEFSRQRA
CILSRSALQLLYLSPSPANTASMAQSAMNGPVGQPRPQHPFVEILRESVKNFVNPPALTPKS
PMVSTPQAREFVENFLARCVRPFAVLLQVCGHNRARQRDKLALLLDEFAALQEEAESVD
AVVSGAAGTGPRACFGTWLLYHVLRVMIAYLLSGLELELYSVHEYHYIFWYLYEFLYGWL
VSALGRAENLSGDSGRRDSRDSRRRKTKKRVRPYAREGLLCNVMQNLCGGYYKALVAFK
LQGKIRQPQSEFDNEAVRYKHRFAPLSVLTPPQVHYHEFCEMTQPLQYENPVILYLGGCKH
FQQARSLLETITTPDQEVQDLLKVAKTNFVVLKLLAGGHKRDSTVPPEFDFSVHRHFPIIKL
V
>XP_022826697.1 N-alpha-acetyltransferase 30-like [*Spodoptera litura*]
MNQLTEENIQSIKCSQKGTNMKNKSKKAKDPENPDEISISNQENLSDSLANTLHINNINRN
LTNGTSDHPNGSPESQKHESENQDDVPIEANEDVNDVNSIQCSDATCKRESIDAPAQIADR
CIFDDALGNLENVSKEEQHPEDEIEIISYESELQMPEIMRLIQKDLSEPYSIYTYRYFIHNWP
KLCFLATHEGKCIGAIVCKLDMHRNVVKRGYIAMLAVDEKYRKRKIGSRLVRKAIQAMIK
DNADEVVLETEITNKPALKLYENLGFVRDKRLFRYYLNGVDALRLKLWLR
>XP_021190649.2 N-alpha-acetyltransferase 30 [*Helicoverpa armigera*]
MNQLTEENVQNMKCSQKGTNIKNKSKKAKDPSENPDEISISNQENLSDTLANTLHINNINS
NLTNGMSDHPNGSPEGPKDKSETREDATSGDVQDVNSVQCSDGMCKRECKDAPAQHTG
RCIYDDALESLDNAISHEEHEPQDEIEIISYESELQMPEIMRLIQKDLSEPYSIYTYRYFIHN
WPKLCFLATHEGKCIGAIVCKLDMHRNVVKRGYIAMLAVDEKYRKRKIGSRLVRKAIQA
MINDNADEVVLETEITNKPALKLYENLGFVRDKRLFRYYLNGVDALRLKLWLR
>XP_022826734.1 N-alpha-acetyltransferase 20 [*Spodoptera litura*]
MTTIRPFTCEDMLKFNNVNLDPLTETYGLSFYTQYLAHWPEYFQVVESPSGEIMGYIMGK
AEGHGENWHGHVTALTVGPEYRRLGLAATLMNILEDVSEKKKAYFVDLFVRVSNKVAIN
MYKNLGYIVYRTVLEYYSGDPDEDAYDMRKACSRDVNKKSVIPLTHPVRPEDVD

>XP_021195271.1 N-alpha-acetyltransferase 20 [*Helicoverpa armigera*]
MTTIRPFTCEDMLKFNNVNLDPLTETYGLSFYTQYLAHWPEYFQVVESPSGEIMGYIMGK
AEGHGDNWHGHVTALTVGPEYRRLGLAATLMNILEDVSEKKKAYFVDLFVRVSNKVAIN
MYKNLGYIVYRTVLEYYSGDPDEDAYDMRKACSRDVNKKSVIPLTHPVRPEDVD
>XP_022830428.1 N-alpha-acetyltransferase 10 [*Spodoptera litura*]
MNIRCARPSDLMNMQHCNLLCLPENYQMKYYFYHGLSWPQLSYVAEDEKGHIVGYVLA
KMEEDGEDNRHGHITSLAVKRSHRRLGLAQKLMNQASLAMVECFKAKYVSLHVRKSNR
AALNLYTNSLGFKILEIEPKYYADGEDAYSMMRDLSAFAAENKTEPQPTENLEIKSESAIIS
QC
>XP_021192849.1 N-alpha-acetyltransferase 10 [*Helicoverpa armigera*]
MNIRCARPSDLMNMQHCNLLCLPENYQMKYYFYHGLSWPQLSYVAEDEKGHIVGYVLA
KMEEDGEDNRHGHITSLAVKRSHRRLGLAQKLMNQASLAMVECFQAKYVSLHVRKSNR
AALNLYTNSLGFKILEIEPKYYADGEDAYSMMRDLSAFAAESKDTQPTENLEIKSESAIISQ
C
>XP_022817999.1 N-alpha-acetyltransferase 40 [*Spodoptera litura*]
MGKKTQANVNKNKEKRNARKQEQRRIADGMSSVNSANKLKDLATLCKELLVYRNNELE
VEMYIQRVTELDKNVLEWAIDLTERNMKHLYETCAWGWNRDRKVEEMTDEGAWYLIAR
EKKGTLLAFSHFRFDMDFGDPVLYCYEVQVEAEGRRRGLGQRVLSVLEKLADATRMRCV
RLTALTHNPSASAFFRACGYSLDETSPGQDEAAHYEILSKLTENQQDGDATQEKCPLSDGM
KAVQVGNPQ
>XP_047034057.1 N-alpha-acetyltransferase 40 [*Helicoverpa zea*]
MGKKTQASATKNKEKRQARKLEQRKIADGMSSVTSANKLKDMATLCKELLVYRNNELE
VEMYIQRVTELDKNVLQWAIDLTERNMKRLYETCAWGWNRDRKVEEMTDEGAWYLIAR
EKNGTLLAFSHFRFDMDFGDPVLYCYEVQVEAEGRRRGLGQRVLIVLEKLANATRMRCV
RLTALTHNPSASAFFRACGYSLDETSPPKDEAAHYEILSKSTDNPDGESSEDKCSLTDGMR
AVQVANTQ
>XP_021193614.2 N-alpha-acetyltransferase 40 [*Helicoverpa armigera*]
MGKKTQASATKNKEKRQARKLEQRKIADGMSSVTSANKLKDMATLCKELLVYRNNELE
VEMYIQRVTELDKNVLQWAIDLTERNMKRLYETCAWGWNRDRKVEEMTDEGAWYLIAR
EKNGTLLAFSHFRFDMDFGDPVLYCYEVQVEAEGRRRGLGQRVLIVLEKLANATRMRCV
RLTALTHNPSASAFFRACGYSLDETSPSKDEAAHYEILSKSTDGPDGESSEDKCSLTDGMR
AVQVANTQ
>XP_022823263.1 N-acetyltransferase 9 [*Spodoptera litura*]
MKLNSNTKIIGRNVVLVPYREYHVPRYHKWMKSEELQKLTASEPLTLEQEYEMQKSWRE
DDDKCTFIILNKTIFEKCNEETGAMVGDTNIFITDKELRVGEIEIMIAEESARGKKLGWEAV
ILMFLYGIQHINLKTFEAKISLSNSISIKMFQKLGFQEKSLSEVFQEITLEKVVNTEWIKWLN
EQAQYEIQTSH
>XP_021200289.1 alpha/beta-tubulin-N-acetyltransferase 9 [*Helicoverpa armigera*]
MKLNSNTKIVGKNLVLVPYREYHVPRYHEWMQSVELQKLTASEPLTLEEEYEMQRSWRE
DEDKCTFIILDKDIYEKSNDETDAMIGDTNIFITDNELAAGEIEIMIAEESARGKKFGWEAVI
LMFLYGIKHINIKLFEAKISLTNTISIKMFNKLGFQEKSVSEVFQEVTLEKKVNDEWIRWLN
EQAQYEIQTC
>XP_047028385.1 alpha/beta-tubulin-N-acetyltransferase 9 [*Helicoverpa zea*]
MKLNSNTKIVGKNLVLVPYREYHVPRYHEWMQSVELQKLTASEPLTLEEEYEMQRSWRE

DEDKCTFIILDKDIYEKSNDDTDAMIGDTNIFITDNELAAGEIEIMIAEESARGKKFGWEAVI
LMFLYGIKHINIKLFEAKISLTNTISIKMFNKLGFQEKSVSEVFQEVTLEKKVNDEWIRWLN
EQAQYEIQTC
>XP_022823402.1 N-alpha-acetyltransferase 15, NatA auxiliary subunit [*Spodoptera litura*]
MPPSNPLPPKENALFKRILRCYEHKQYKNGLKFAKQILSNPKFAEHGETLAMKGLTLNCL
GRKEEAYEYVRRGLRNDLKSPVCWHVYGLLQRSDKKYDEAIKCYRNALKWEKENIQILR
DLSLLQIQMRDLEGYKDTRYQLFMLRPTQRASWIGFAMSYHLLGDYEMAISILDAFRTNQ
MKGTDDYEHSELLLYQNMVLAESGQYERALQHLQKFSSQILDKLSIKETSGEYYLKLKRF
KEAESVYEDLLKRNPENVMYYEKLIEAKQLVDPDEKVAFFEVYKKEFPRAIAPRRLQLTE
ALAQPVFEKLVDEYLRHGLHKGIPPLFVDLRSLYAIQDKAETIEKLILQYLENLSKNGTFGP
DPSEVKQPASALLWAYYYAAQHFDYKKDTDRALQYIDAAIDHTPTLIELFIVKGRIYKHAG
DPVSAYQWLEEAQAMDTADRYVNSKCARYMLRAGHVKRAEDMCAKFTREGVPATENL
NEMQCMWFQTEAAAAYKRLQQWGEALKKAHEVDRHFSEIMEDQFDFHSYCMRKMTLR
SYVGLLRLEDVLRAHPFYFRAAKVAVDVYLRLDQHPLQDAPQTAEPDTENLAPSELKKLR
NKQRKAKRKAEQESALQAQVQVKREQHHKARQQQEQGDPEAPQLDELIPDKLARAEDP
LEQALKFLQPLRTLAADRIDTHLMAFEIYFRKDKPLLMLQSIKRAHRLDAAHHHLHDCLL
RFQGWLDDNLASLNPAVAAVITKEIEPMVRGRSTEEMAEQFVSQPGAARCQAGALSAAR
ALRRLRPPRALHALQLATSLDYPDLSIQGCVDVLDSLRDGDFGPCEKEIEQYIEACRKKFP
YAIAFKPASELSELADDHVADDAPLQPKEIAVNN
>XP_021190458.1 N-alpha-acetyltransferase 15, NatA auxiliary subunit isoform X1 [*Helicoverpa armigera*]
MPPSNPLPPKENALFKRILRCYEHKQYKNGLKFAKQILSNPKFAEHGETLAMKGLTLNCL
GRKDEAYEYVRRGLRNDLKSPVCWHVYGLLQRSDKKYDEAIKCYRNALKWEKENIQIL
RDLSLLQIQMRDLEGYKDTRYQLFMLRPTQRASWIGFAMSYHLLGDYEMANSILDAFRT
NQMKGPYDYEHSELLLYQNMVLAESGQYERALQHLHKFSTQILDKLSIKETSGEYYLKL
KRFKEAEAVYEDLLKRNPENVMYYEKLIEAKQLVTPEEKVAFFDVYKKEYPRAIAPRRLQ
LTEAVAQPVFEQLVDEYLRHGLHKGIPPLFVDLRSLYSDQSKADTIEKLILQYLEHLAKSGT
FGPEESEQKQPASALLWAYYYAAQHFDFKKDTDRALHYIDAAIEHTPTLIELFIVKGRIYKH
AGDPVSAYQWLEEAQAMDTADRYVNSKCARYMLRAGHVKRAEEMCAKFTREGVPATE
NLNEMQCMWFQTEAAAAYKRLHQWGEALKKAHEVDRHFSEIMEDQFDFHSYCMRKMT
LRSYVGLLRLEDVLRAHPFYFRCARVAIAVYLRLYTQPLQDAPQTQEPDTENLAPSELKKL
RNKQRKAKRKAEQESALQAQVQVKREQHHKARQQQEQGDPEAPQLDELVPDKLARAE
DPLEQAIKFLQPLRTLAADRIDTHLMAFEIYYRKDKPLLMLQSIKRAFQLDSSHHHLHDCL
LRFQRWLDDNLAGLNPAVAAVINKEIEPMVRGRSAVQMAEEFIRSAADKTQANALWGAR
ALRRLLPERAHQALKLATALHYPDLSIQGCVDVLDSLREGDFGPCEKEIEQYIEACRSKFP
YAIAFKPASALADLPDNHVADDAPLQPKEIAANN
>XP_047027460.1 N-alpha-acetyltransferase 15, NatA auxiliary subunit [*Helicoverpa zea*]
MPPSNPLPPKENALFKRILRCYEHKQYKNGLKFAKQILSNPKFAEHGETLAMKGLTLNCL
GRKDEAYEYVRRGLRNDLKSPVCWHVYGLLQRSDKKYDEAIKCYRNALKWEKENIQIL
RDLSLLQIQMRDLEGYKDTRYQLFMLRPTQRASWIGFAMSYHLLGDYEMANSILDAFRT
NQMKGPYDYEHSELLLYQNMVLAESGQYERALQHLHKFSTQILDKLSIKETSGEYYLKL
KRFKEAEAVYEDLLKRNPENVMYYEKLIEAKQLITPEEKVAFFDVYKKEYPRAIAPRRLQ
LTEAVAQPVFEQLVDEYLRHGLHKGIPPLFVDLRSLYSDQSKADTIEKLILQYLEHLAKSGT
FGPEESEQKQPASALLWAYYYAAQHFDFKKDTDRALHYIDAAIEHTPTLIELFIVKGRIYKH

AGDPVSAYQWLEEAQAMDTADRYVNSKCARYMLRAGHVKRAEEMCAKFTREGVPATE
NLNEMQCMWFQTEAAAAYKRLHQWGEALKKAHEVDRHFSEIMEDQFDFHSYCMRKMT
LRSYVGLLRLEDVLRAHPFYFRCARVAIAVYLRLYTQPLQDAPQTQEPDTENLAPSELKKL
RNKQRKAKRKAEQESALQAQVQVKREQHHKARQQQEQGDPEAPQLDELVPDKLARAE
DPLEQAIKFLQPLRTLAADRIDTHLMAFEIYYRKDKPLLMLQSIKRAFQLDSSHHHLHDCL
LRFQRWLDDNLAGLNPAVAAVINKEIEPMVRGRSAVQMAEEFIRSAADKTQANALWGAR
ALRRLLPERAHQALKLATALHYPDLSIQGCVDVLDSLREGDFGPCEKEIEQYIEACRSKFP
YAIAFKPASALADLPDNHVADDAPLQPKEIAANN
>XP_022830430.1 N-alpha-acetyltransferase 10 [*Spodoptera litura*]
MNIRCARPSDLMNMQHCNLLCLPENYQMKYYFYHGLSWPQLSYVAEDEKGHIVGYVLA
KMEEDGEDNRHGHITSLAVKRSHRRLGLAQKLMNQASLAMVECFKAKYVSLHVRKSNR
AALNLYTNSLGFKILEIEPKYYADGEDAYSMMRDLSAFAAENKTEPQPTENLEIKSESAIIS
QC
>XP_022826734.1 N-alpha-acetyltransferase 20 [*Spodoptera litura*]
MTTIRPFTCEDMLKFNNVNLDPLTETYGLSFYTQYLAHWPEYFQVVESPSGEIMGYIMGK
AEGHGENWHGHVTALTVGPEYRRLGLAATLMNILEDVSEKKKAYFVDLFVRVSNKVAIN
MYKNLGYIVYRTVLEYYSGDPDEDAYDMRKACSRDVNKKSVIPLTHPVRPEDVD
>XP_022826697.1 N-alpha-acetyltransferase 30-like [*Spodoptera litura*]
MNQLTEENIQSIKCSQKGTNMKNKSKKAKDPENPDEISISNQENLSDSLANTLHINNINRN
LTNGTSDHPNGSPESQKHESENQDDVPIEANEDVNDVNSIQCSDATCKRESIDAPAQIADR
CIFDDALGNLENVSKEEQHPEDEIEIISYESELQMPEIMRLIQKDLSEPYSIYTYRYFIHNWP
KLCFLATHEGKCIGAIVCKLDMHRNVVKRGYIAMLAVDEKYRKRKIGSRLVRKAIQAMIK
DNADEVVLETEITNKPALKLYENLGFVRDKRLFRYYLNGVDALRLKLWLR
>XP_022826548.1 N-alpha-acetyltransferase 38-B, NatC auxiliary subunit [*Spodoptera litura*]
MSNTVSEQLTDAIKNTEDGKAKLRKWLNMNFRIEMTDGRVLIGVFLCTDRDANVILGAC
SEYLKSGDGETEEPRVLGLVMVPGRHIVSIQLDDTTPPQMYCYDE
>XP_022823402.1 N-alpha-acetyltransferase 15, NatA auxiliary subunit [*Spodoptera litura*]
MPPSNPLPPKENALFKRILRCYEHKQYKNGLKFAKQILSNPKFAEHGETLAMKGLTLNCL
GRKEEAYEYVRRGLRNDLKSPVCWHVYGLLQRSDKKYDEAIKCYRNALKWEKENIQILR
DLSLLQIQMRDLEGYKDTRYQLFMLRPTQRASWIGFAMSYHLLGDYEMAISILDAFRTNQ
MKGTDDYEHSELLLYQNMVLAESGQYERALQHLQKFSSQILDKLSIKETSGEYYLKLKRF
KEAESVYEDLLKRNPENVMYYEKLIEAKQLVDPDEKVAFFEVYKKEFPRAIAPRRLQLTE
ALAQPVFEKLVDEYLRHGLHKGIPPLFVDLRSLYAIQDKAETIEKLILQYLENLSKNGTFGP
DPSEVKQPASALLWAYYYAAQHFDYKKDTDRALQYIDAAIDHTPTLIELFIVKGRIYKHAG
DPVSAYQWLEEAQAMDTADRYVNSKCARYMLRAGHVKRAEDMCAKFTREGVPATENL
NEMQCMWFQTEAAAAYKRLQQWGEALKKAHEVDRHFSEIMEDQFDFHSYCMRKMTLR
SYVGLLRLEDVLRAHPFYFRAAKVAVDVYLRLDQHPLQDAPQTAEPDTENLAPSELKKLR
NKQRKAKRKAEQESALQAQVQVKREQHHKARQQQEQGDPEAPQLDELIPDKLARAEDP
LEQALKFLQPLRTLAADRIDTHLMAFEIYFRKDKPLLMLQSIKRAHRLDAAHHHLHDCLL
RFQGWLDDNLASLNPAVAAVITKEIEPMVRGRSTEEMAEQFVSQPGAARCQAGALSAAR
ALRRLRPPRALHALQLATSLDYPDLSIQGCVDVLDSLRDGDFGPCEKEIEQYIEACRKKFP
YAIAFKPASELSELADDHVADDAPLQPKEIAVNN
>XP_022821908.1 N-alpha-acetyltransferase 35, NatC auxiliary subunit isoform X2 [*Spodoptera litura*]

MDSLGATPEVIYNWVDITSDFFKHIQDLQLGELLHDGHLFGLFEAMSAIEMMDPKMDAG
MLCNRGNPKPLNFQQAVAAGKLKIDDLEPPELIGIIDATMACIVSWLEGHSLAQTVFTNLY
LHQPHSIVNKTLKAYCIAVYKLLDCIRDCINKAQVFEEEDFQPMGYGYRLGSNPQSGNTY
DARLEVSEQKCVLMLREQEEELNKKARSCDDEDNLWAALQARIRFTRMFYRALLMITKR
DSQSGADCVALLNGCSEMMKVIIKTSGKGTQPVENSDSPNPMGFEPMINQRLLPPTFPRYT
RIKPRAEALHYFDELVARLRHAWKITSCTNFHTALDFFMEFSRQRACILSRSALQLLYLSPS
PANTASMAQSAMNGPVGQPRPQHPFVEILRESVKNFVNPPALTPKSPMVSTPQAREFVENF
LARCVRPFAVLLQVCGHNRARQRDKLALLLDEFAALQEEAESVDAVVSGAAGTGPRACF
GTWLLYHVLRVMIAYLLSGLELELYSVHEYHYIFWYLYEFLYGWLVSALGRAENLSGDSG
RRDSRDSRRRKTKKRVRPYAREGLLCNVMQNLCGGYYKALVAFKLQGKIRQPQSEFDNE
AVRYKHRFAPLSVLTPPQVHYHEFCEMTQPLQYENPVILYLGGCKHFQQARSLLETITTPD
QEVQDLLKVAKTNFVVLKLLAGGHKRDSTVPPEFDFSVHRHFPIIKLV
>XP_022817999.1 N-alpha-acetyltransferase 40 [*Spodoptera litura*]
MGKKTQANVNKNKEKRNARKQEQRRIADGMSSVNSANKLKDLATLCKELLVYRNNELE
VEMYIQRVTELDKNVLEWAIDLTERNMKHLYETCAWGWNRDRKVEEMTDEGAWYLIAR
EKKGTLLAFSHFRFDMDFGDPVLYCYEVQVEAEGRRRGLGQRVLSVLEKLADATRMRCV
RLTALTHNPSASAFFRACGYSLDETSPGQDEAAHYEILSKLTENQQDGDATQEKCPLSDGM
KAVQVGNPQ
>XP_049691873.1 N-alpha-acetyltransferase 35, NatC auxiliary subunit isoform X3 [*Helicoverpa armigera*]
MDSLGATPEVIYNWVDITSDFFSHIQDLQLGELLHDGHLFGLFEAMSAIEMMDPKMDAG
MLCNRGNPKPLNFQQAVAAGKLKIDDLEPSELIGIIDATMACIVSWLEGHSLAQTVFTNLY
LHQPHSINNKTLKAYCIAVYKLLDCIRDCINKAQVFEEEDFQPMGYGYRLGSNPQSGNTY
DARLEVSEQKCVLMLREQEEELNKKARSSDDEDNLWAALQARIRFTRMFFQALLMITKR
DSQSGADCVALLNGCSEMMKVIIKTSGKGTQPVENSDSPNPMGFEPMINQRLLPPTFPRYT
RIKHRAEALHYFDELVARLRHAWKITSCTNFHTALDFFMEFSRQRACILSRSALQLLYLSPS
PANTAAMAQSAMNGPAGQPRPHHPFVEILRESVKNFVNPPALTPKSPMVSTPQAREFVEN
FLARCVRPFAVLLQVCGHNRARQRDKLALLLDEFAALQEEAEGVDAVVSGAAGTGPRAC
FGTWLLYHVLRVMIAYLLSGLELELYSVHEYHYIFWYLYEFLYGWLVSALGRAENLAGDA
ARRDARDSRKQRKSKKRVRPYAREGLLCNVMQNMCGGYYKALVAFKLQGKIRQPQSEF
DNEAVRYKHRFAPLSVLTPPQVHYHEFCEMTQPLQYENPVILYLGGCKHFQQARSLLETIT
TPDQEVQDLLKVAKTNFVVLKLLAGGHKRDSTVPPEFDFSVHRHFPIIKLV
>XP_049691871.1 N-alpha-acetyltransferase 35, NatC auxiliary subunit isoform X2 [*Helicoverpa armigera*]
MHIGRMDSLGATPEVIYNWVDITSDFFSHIQDLQLGELLHDGHLFGLFEAMSAIEMMDPK
MDAGMLCNRGNPKPLNFQQAVAAGKLKIDDLEPSELIGIIDATMACIVSWLEGHSLAQTV
FTNLYLHQPHSINNKTLKAYCIAVYKLLDCIRDCINKAQVFEEEDFQPMGYGYRLGSNPQS
GNTYDARLEVSEQKCVLMLREQEEELNKKARSSDDEDNLWAALQARIRFTRMFFQALLM
ITKRDSQSGADCVALLNGCSEMMKVIIKTSGKGTQPVENSDSPNPMGFEPMINQRLLPPTF
PRYTRIKHRAEALHYFDELVARLRHAWKITSCTNFHTALDFFMEFSRQRACILSRSALQLL
YLSPSPANTAAMAQSAMNGPAGQPRPHHPFVEILRESVKNFVNPPALTPKSPMVSTPQARE
FVENFLARCVRPFAVLLQVCGHNRARQRDKLALLLDEFAALQEEAEGVDAVVSGAAGTG
PRACFGTWLLYHVLRVMIAYLLSGLELELYSVHEYHYIFWYLYEFLYGWLVSALGRAENL
AGDAARRDARDSRKQRKSKKRVRPYAREGLLCNVMQNMCGGYYKALVAFKLQGKIRQP

QSEFDNEAVRYKHRFAPLSVLTPPQVHYHEFCEMTQPLQYENPVILYLGGCKHFQQARSL
LETITTPDQEVQDLLKVAKTNFVVLKLLAGGHKRDSTVPPEFDFSVHRHFPIIKLV
>XP_021192852.1 N-alpha-acetyltransferase 10 [*Helicoverpa armigera*]
MNIRCARPSDLMNMQHCNLLCLPENYQMKYYFYHGLSWPQLSYVAEDEKGHIVGYVLA
KMEEDGEDNRHGHITSLAVKRSHRRLGLAQKLMNQASLAMVECFQAKYVSLHVRKSNR
AALNLYTNSLGFKILEIEPKYYADGEDAYSMMRDLSAFAAESKDTQPTENLEIKSESAIISQ
C
>XP_021193614.2 N-alpha-acetyltransferase 40 [*Helicoverpa armigera*]
MGKKTQASATKNKEKRQARKLEQRKIADGMSSVTSANKLKDMATLCKELLVYRNNELE
VEMYIQRVTELDKNVLQWAIDLTERNMKRLYETCAWGWNRDRKVEEMTDEGAWYLIAR
EKNGTLLAFSHFRFDMDFGDPVLYCYEVQVEAEGRRRGLGQRVLIVLEKLANATRMRCV
RLTALTHNPSASAFFRACGYSLDETSPSKDEAAHYEILSKSTDGPDGESSEDKCSLTDGMR
AVQVANTQ
>XP_021198318.1 N-alpha-acetyltransferase 80 [*Helicoverpa armigera*]
MKMETQNLQVVRLHENPHYLKACCELINDEWPRSETARMMSLQASCNHLPTSLILIDDM
KRLLGHCKLTAIPSIPESCFIETVVISKSMRGKRLGSYLMRQVEEYCKNILKLKMLHLSTKG
QENFYAKLGYENCAPVSIYGVRSFNSVPTISDKIPNQVPVINNIVEGAPPPPPPPMPKPETM
VNNTLKSTKTFMFKYL
>XP_021195271.1 N-alpha-acetyltransferase 20 [*Helicoverpa armigera*]
MTTIRPFTCEDMLKFNNVNLDPLTETYGLSFYTQYLAHWPEYFQVVESPSGEIMGYIMGK
AEGHGDNWHGHVTALTVGPEYRRLGLAATLMNILEDVSEKKKAYFVDLFVRVSNKVAIN
MYKNLGYIVYRTVLEYYSGDPDEDAYDMRKACSRDVNKKSVIPLTHPVRPEDVD
>XP_021190653.2 N-alpha-acetyltransferase 38-B, NatC auxiliary subunit [*Helicoverpa armigera*]
MSDTTSEQPIDTIKNVEDGKAKLRKWLNMNFRIEMTDGRVLIGVFLCTDRDANVILGACS
EYLKSSDGETEEPRVLGLVMVPGRHIVSIQLDDTTPPQMYCYDE
>XP_021190649.2 N-alpha-acetyltransferase 30 [*Helicoverpa armigera*]
MNQLTEENVQNMKCSQKGTNIKNKSKKAKDPSENPDEISISNQENLSDTLANTLHINNINS
NLTNGMSDHPNGSPEGPKDKSETREDATSGDVQDVNSVQCSDGMCKRECKDAPAQHTG
RCIYDDALESLDNAISHEEHEPQDEIEIISYESELQMPEIMRLIQKDLSEPYSIYTYRYFIHN
WPKLCFLATHEGKCIGAIVCKLDMHRNVVKRGYIAMLAVDEKYRKRKIGSRLVRKAIQA
MINDNADEVVLETEITNKPALKLYENLGFVRDKRLFRYYLNGVDALRLKLWLR
>XP_049697255.1 N-alpha-acetyltransferase 15, NatA auxiliary subunit isoform X2 [*Helicoverpa armigera*]
MPPSNPLPPKENALFKRILRCYEHKQYKNGLKFAKQILSNPKFAEHGETLAMKGLTLNCL
GRKDEAYEYVRRGLRNDLKSPVCWHVYGLLQRSDKKYDEAIKCYRNALKWEKENIQIL
RDLSLLQIQMRDLEGYKDTRYQLFMLRPTQRASWIGFAMSYHLLGDYEMANSILDAFRT
NQMKGPYDYEHSELLLYQNMVLAESGQYERALQHLHKFSTQILDKLSIKETSGEYYLKL
KRFKEAEAVYEDLLKRNPENVMYYEKLIEAKQLVTPEEKVAFFDVYKKEYPRAIAPRRLQ
LTEAVAQPVFEQLVDEYLRHGLHKGIPPLFVDLRSLYSDQSKADTIEKLILQYLEHLAKSGT
FGPEESEQKQPASALLWAYYYAAQHFDFKKDTDRALHYIDAAIEHTPTLIELFIVKGRIYKH
AGDPVSAYQWLEEAQAMDTADRYVNSKCARYMLRAGHVKRAEEMCAKFTREGVPATE
NLNEMQCMWFQTEAAAAYKRLHQWGEALKKAHEVDRHFSEIMEDQFDFHSYCMRKMT
LRSYVGLLRLEDVLRAHPFYFRCARVAIAVYLRLYTQPLQDAPQTQEPDTENLAPSELKKL
RNKQRKAKRKAEQESALQAQVKREQHHKARQQQEQGDPEAPQLDELVPDKLARAEDPL

EQAIKFLQPLRTLAADRIDTHLMAFEIYYRKDKPLLMLQSIKRAFQLDSSHHHLHDCLLRF
QRWLDDNLAGLNPAVAAVINKEIEPMVRGRSAVQMAEEFIRSAADKTQANALWGARALR
RLLPERAHQALKLATALHYPDLSIQGCVDVLDSLREGDFGPCEKEIEQYIEACRSKFPYAIA
FKPASALADLPDNHVADDAPLQPKEIAANN
>XP_021190458.1 N-alpha-acetyltransferase 15, NatA auxiliary subunit isoform X1 [*Helicoverpa armigera*]
MPPSNPLPPKENALFKRILRCYEHKQYKNGLKFAKQILSNPKFAEHGETLAMKGLTLNCL
GRKDEAYEYVRRGLRNDLKSPVCWHVYGLLQRSDKKYDEAIKCYRNALKWEKENIQIL
RDLSLLQIQMRDLEGYKDTRYQLFMLRPTQRASWIGFAMSYHLLGDYEMANSILDAFRT
NQMKGPYDYEHSELLLYQNMVLAESGQYERALQHLHKFSTQILDKLSIKETSGEYYLKL
KRFKEAEAVYEDLLKRNPENVMYYEKLIEAKQLVTPEEKVAFFDVYKKEYPRAIAPRRLQ
LTEAVAQPVFEQLVDEYLRHGLHKGIPPLFVDLRSLYSDQSKADTIEKLILQYLEHLAKSGT
FGPEESEQKQPASALLWAYYYAAQHFDFKKDTDRALHYIDAAIEHTPTLIELFIVKGRIYKH
AGDPVSAYQWLEEAQAMDTADRYVNSKCARYMLRAGHVKRAEEMCAKFTREGVPATE
NLNEMQCMWFQTEAAAAYKRLHQWGEALKKAHEVDRHFSEIMEDQFDFHSYCMRKMT
LRSYVGLLRLEDVLRAHPFYFRCARVAIAVYLRLYTQPLQDAPQTQEPDTENLAPSELKKL
RNKQRKAKRKAEQESALQAQVQVKREQHHKARQQQEQGDPEAPQLDELVPDKLARAE
DPLEQAIKFLQPLRTLAADRIDTHLMAFEIYYRKDKPLLMLQSIKRAFQLDSSHHHLHDCL
LRFQRWLDDNLAGLNPAVAAVINKEIEPMVRGRSAVQMAEEFIRSAADKTQANALWGAR
ALRRLLPERAHQALKLATALHYPDLSIQGCVDVLDSLREGDFGPCEKEIEQYIEACRSKFP
YAIAFKPASALADLPDNHVADDAPLQPKEIAANN

ACBP

>ARD71233.1 acyl-CoA binding protein [*Spodoptera exigua*]
MAEALPEYPDSDFSDDEQSPLDKSFSKASDHVRKLTNVLNNNQLLELYGLYKQGTEGKC
NIPKPGWLDGRGRKKWEAWNSLHNMPQDEAKQKYIALVQKYAPELTDLSNDNESGGKE
AWVAVSSMLKLPEPELVHNELSILDAARENCADRVTELLSKHPELRHERDEDGLSALHWA
ADRNATEALKAALEGGCPVDAADECGQTALHYAATCGHIESTTILLKAGAALLKDEDDCT
PLDLASDDDIRKVLEGAK
>ARD71234.1 acyl-CoA binding protein [*Spodoptera exigua*]
MSLQEQFDKAAGDVKKLKSLPSDSDLLELYALFKQATVGDSDPSKAPGFLDLKGKAKFE
AWTKKKGLSKEDAQKAYIAKVEQLIASIGLQ
>ALJ30272.1 putative acyl-CoA binding protein ACBP1 [*Spodoptera litura*]
MAEALPEYPDSDFSDDEQSPLDKSFSKASDHVRKLTNVLNNNQLLELYGLYKQGTEGKC
NIPKPGWLDGRGRKKWEAWNSLRDMPQDEAKQKYIALVQKYAPELTDLSNDNESGVKE
AWVAVSSMLKSPEPELVHNELSILDAARENCADRVTELLSKHPELRHETDEDGLSALHWA
ADRNATEALKAALEGGCPVDAVDECGQTALHYAATCGHIESTTILLKAGASLLKDEDDCT
PLDLASDDDIRKVLEGAK
>ALJ30273.1 putative acyl-CoA binding protein ACBP2 [*Spodoptera litura*]
MSLQEQFDKAAGDVKKLKSLPSDADLLELYALFKQATVGDSDPSKAPGFLDLKGKAKFE
AWSKKKGLSKEDAQKAYVAKVEQLIASIGLQ
>ABK29477.1 acyl-CoA binding protein [*Helicoverpa armigera*]
MSLDEQFSKVATSVRNWKTTPSNDENLALYSLYKQATIGDVNIAEPSGMVENAKFKAWSG
RKGISQDDAKKQYIELAEKLAPKFA
