# Supplementary material for: Genome-Wide Identification and Expression Profiling of Candidate Sex Pheromone Biosynthesis Genes in the Fall Armyworm (Spodoptera frugiperda)
Source: Insects. 2022 Nov 23;13(12):1078. doi: 10.3390/insects13121078 (PMC9783692; doi:10.3390/insects13121078)
Supplement: Supplementary file 1 [file insects-13-01078-s001.zip › Table S2-Primers used for RT-PCR and qPCR.pdf]

Table S2 Primers used for RT-PCR and qPCR

Primers	Primer sequences	Usage of primers
SfurDES2-ORF-F	ATGGCTCCAGCGCAAAAACACATCC	Amplificaion of ORF
*Sfur*DES2-ORF-R	TCATTCGGCGTGACATTTTTCTGCC	
*Sfur*DES5-ORF-F	ATGGCTCAAACTATACAAACAACAA	
*Sfur*DES5-ORF-R	CTATTCGCCTTTTGTATTTTTTAAA	
*Sfur*FAR2-ORF-F	ATGACGTATAGACGAGTAAGTGAAT	
*Sfur*FAR2-ORF-R	TTAAGTATAATTCTTCATCAAAAAC	
*Sfur*FAR3-ORF-F	ATGGTTGTGTTGACGTCGAAAGAGA	
*Sfur*FAR3-ORF-R	TTATTTTTTCTTTTCCAAAAACTTA	
*Sfur*DES2-104-F	CCAGCGCAAAAACACATCCA	qPCR
*Sfur*DES2-104-R	TCGTACTGTGGAGCTTCGTT	
*Sfur*DES5-100-F	ACCAGCACAAAACATAGCCGT	
*Sfur*DES5-100-R	TCCAAGTTCAGCTGTGCGATA	
*Sfur*FAR2-135-F	TCATCAGCCACAGCATGACAA	
*Sfur*FAR2-135-R	ATGATGATTGTGGGGACGTGT	
*Sfur*FAR3-100-F	ACTGGTGGAACGGGGTTTTT	
*Sfur*FAR3-100-R	CCTTCTTCTCCCTGATCAGCAT	
*Sfur*RPS18-143-F	GACTGGTTCCTCAACAGGCA	
*Sfur*RPS18-143-R	CCCCAGTAGTGACGCATACC	
*Sfur*EF1α-165-F	ACACCTGTGTTGGATTGCCA	
*Sfur*EF1α-165-R	AACACACAGAGGCTTGGAGG	
