# Supplementary material for: Genome-Wide Identification and Expression Profiling of Candidate Sex Pheromone Biosynthesis Genes in the Fall Armyworm (Spodoptera frugiperda)
Source: Insects. 2022 Nov 23;13(12):1078. doi: 10.3390/insects13121078 (PMC9783692; doi:10.3390/insects13121078)
Supplement: Supplementary file 1 [file insects-13-01078-s001.zip › Table S3-Sequences for phylogenetic tree.pdf]

DESs

>*SexiDes2*

MAPAQKHVQMCGDEIQSGLKISPVTYDADKLNEGPQYENNNTVLRNSANDKVNSDADF
DISKYEAIDFKAKFRWPDLTVQILLHLVSIYGLYLTFSNQVKILTLLFALGTIYTSGFGITAGV
HRLWSHRAYRARLPLRIILALLFTVTGQRDIYTWALDHRVHHKYAETVADPHDIRRGFWF
AHVGWLVLTPHPAVEDRRIALRPTCADLIADPVVRLQKQFFIPMFALLNIAIPIWVPWYCW
NETLVNSFVISFVMRFTITLNIAFCVNSFAHLWGNKPYDRFVKSVENSLVSLAALGEGWHN
YHHVFPWDYRTSELGKLNISTGFIDFFAKIGWAYDLKAATTDMISKRAKRCGDGTFGESEE
PYPSSEKCHAE

>*SexiDes3*

MAPNTEKRQVSFPKLEYPIFREAQPKSAQHWLKGKRLQDGAEDLWRIHDSLYDLTDFISS
HPGGTHWISVTKGTDITEAFETHHLKGIAESLLPNYYVRKAIKPRNQPFTFKEDGFYKTLK
LKVMDQMALIPKDVRKKSDFITDSLLLALIILAPLSCWGWTQSFVIGASLTFSTGFVLSSLV
TCAHNYFHRGDNWRMYIFNLAGMSFNDWRVSHSMSHHLHTNTAQDIELSMIEPFLQFIPY
KDKPIWAQMGAFYYPLVYATSLLSIMGHELILSATNHEGKTLSWRNFIPFTIPAWMYLMGG
LPLHSTILLWLVTLVPASFFFVFYGLTAGHHNHRNFFEGDVPRDENIDWGIHQLDAICERID
YAGNHFKSITRFGDHALHHLFPTLDHAELKYLYPVLLEHCEKFDFQFKTNTFYETIINASK
QLIRKRPNNFRDVKATK

>*SexiDes4*

MAHITKTISSKILNKSTHRSLSTAVSQIRIYEVGPRDGLQNEAKFVPTDIKIELINKLAAAGI
KDIESASFVSPKWVKQMSDGVDVMKNIPRVPGVNYPVLVPNLKGYDIAKQCNIEEVAIFP
AGSEAFSQKNLNCSVEEGLKRFKLVADQAVKDGIRVRGYVSCVVGCPYEGPIHPKGIAKIT
EQLFEMGCYEVSLGDTIGVGTAGSVKRLMREVLTVAKPEQLALHFHDTYGQALSNLLAG
LEFGIKTVDSSISGLGGCPYARGASGNLATEDLVYLLYGLGVNTNVDLVKLIEAGRYISNFL
GKPTESKVNRAISDRFKKHNDIVKIASCDI

>*SexiDes5*

MAQTIQTTTILEQKEEKTVTLLVPQAGKRKFEFVYQNLITFAYWHIAGLYGLYLCFTSAKW
ATILFSFILFVIAEIGITAGAHRLWSHKSYKVKLPLEILLMVMNSIAFQNTVIDWVRDHRLH
HKYSDTDADPHNASRGFFYSHIGWLFVRKHPEVKKRGKELDMSDIYNNPVLRFQRKYAV
PFIGAVCFGLPTLIPVYCWGESWTNAWHITMLRYIMNLNATFLVNSAAHIYGKRPYDKKIL
PAQNIGVSIATFGEGFHNYHHVFPWDYRAAELGNNGLNLTTKFIDFFAWIGWAYDLKTVS
KEMIKQRSKRTGDGTNLWGLEDKDTPENLKNIKGE

>*SexiDes7*

MAAMSGAPLLLANATLTSKLQDDDHRYAEPMKKNRDYEWQVVWRNVFAFVYLHAAAL
YGFYLMFTGKVRIWTILFGLLFAIMAGMGVTAGAHRLWAHRSYKARWPLRLFLALMQT
MAFQNHIYEWVRDHRVHHKFTETDADPHNARRGFFFSHIGWLMVRKHKDVFEKGATVD
MSDLEKDPIVMFQKKTYMVLMPLLCFVIPAWIPCYFWGENPWYSWYVASITRYTVALHFT
WLVNSAAHIWGNRPYDKNIGATDNKAVAICAFGEGWHNYHHVFPWDYKAAELGNYSTN
LSTALIDFAAKHGLAYDLKTVSAEMIRQRVNRTGDGSHPWTKDSQEEEHYHPENPVWGW
EDTDMTEEEKQFAEIVHRKTE

>*SexiDes8*

MGARVSRTDFEWVYTEEPHASRRKIILEKYPQIKKLFGYDPNFKWVVTGMVLVQIISLPFV
TQLSWPMMLVVAYCFGGVINHSLMLAIHEIAHNLAFGHNRPLANKLFGFFANLPIGLPVSI
SFKKYHLEHHRYQGNEVIDTDLPTLLEAKLFCTTGGKLLWLFLQPFFYSFRPLVVRPKPPT

PMELINLVIQLFFDAVVIKLWGWKAIGYLLIGALMAMGVHPVAGHFVAEHYMFKKGYET
YSYYGPLNWITFNVGYHNEHHDFPAVPGSKLPEVRRIAPEFYDTLPHHDSWSKVLYDFVM
DPDIGPYARIKRKHHGLDS

>*SexiDes9*

MPPQGQTGGSWVLYETDAVNVDSEAPVIVPPSAEKREWKIVWRNVILMGLLHIGGVYGA
YLFLTTAMWRTSLFAVFLYICSGLGITAGAHRLWAHKSYKARLPLRLLLTLFNTLAFQDAVI
DWARDHRMHHKYSETDADPHNATRGFFFSHVGWLLVRKHPQIKAKGHTIDLSDLKNDPI
LRFQKKYYLILMPLICFILPCYIPTLWGESLWNAYFVCSIFRYVYVLNVTWLVNSAAHLWG
AKPYDKNINPVETKPVSLVVLGEGFHNYHHTFPWDYKTAELGDYSLNLTKLFIDFMAAIG
WAYDLKTVSPDVIQKRVKRTGDGSHAVWGWDDHEVQQEDKKLAAIINPDKTE

>*SexiDes10*

MPPNADWEEVAQQRAIDKNTHVSFPQLKYPSLRDDGLRDPVQWLAGKAMDDGAEGLW
RIHDKLYDLTRFIKRHPGGEEWLELTQGTDITEAFESHHLNPSTEKILTQYYIRDAKTPRNSP
FTFKEDGFYKTLKRAAFEELKKIPKDASRTANNITDGLFVSLLISSAMASWVTNYYAVKF
WYTYASINLAILTVCCHNYIHRKTNWRMYLFNMSMWSYRDFRVSHVLSHHLYTNTLMD
LELSSLEPFLFYTPRKDKPLHAKLGFITEIFFFPFVFFLSFVKRFLSIFLHQGFFKSHYRWHD
AIGLLLPLWMIIASGAPILDVISMWLWINCTGSLIFFSIAVNAAHHHPDAIKDGDQPASETPD
WGMHQVEALLDRKDVNGNVFAVMTLFGDHCLHHMFPTLDHSVLKYMHPLFIDLCEKYQ
ANYRVSTQFKLVLGQIKETMRTEFKTKND

>*SexiDes11*

MAPNISEDVNGVLFESDAATPDLALARPPVQKADNKPKQLVWRNIILFAYLHLAALYGGY
LFLFSAKWQTDIFAYILYVISGLGITAGAHRLWAHKSYKAKWPLKVILIIFNTVAFQDAAM
DWARDHRMHHKYSETDADPHNATRGFFFSHIGWLLVRKHPDLKEKGKGLDMSDLLADP
LLRFQKKYYLVLMPLACFVMPTMIPVYLWGETWTNAFFVAAMFRYAFILNVTWLVNSAA
HKWGDKPYDKSIKPSENMSVAMFALGEGFHNYHHTFPWDYKTAELGNNKLNFATAFINF
FAKIGWAYDMKTVSDDIVKNRVKRTGDGSHHLWGWGDKNQPKEEIEAAIRINPKDD

>*SexiDes12*

MVEVKEAAPVAEEQKLSKSREANWPAVLFFIHIHLLSLYGTYLLLFEVKMMTLLFFILLTS
VALLGMTTGAHRLWAHQAYQASTGLKITLMLFQTLAGIGSIYDWVKYHRFHHAHFATDV
DPYDYNQGFIHSHLLTRLRKLSPHQEKLMQSIDMSDLEKDSVVMFQKRLYWVLYAIIFALL
PLNAPLEYWDDTILSSAFVIGFLRYLVVLHGSWLIESAICVWGLKPGEKSPPDTNAVFILTK
TFWPHYHYLVPYDYKSGEYGTYDSGCSTAFIRVWAALGLATKLRTVETVTIQKALAESAR
TKKDLKACIDAAVNNQKLPDEHYLKRA

>*SlitDes2*

MAPAQKHVQMCGDEIQSGLKISPLTYDADKLNEAPQYENNNTVLRNSANDKVNSEADFD
INKYEAIDFKAKIRWPDLTVQILLHLVSIYGLYLMISNQVKLLTLLFALGTIYTSGFGITAGV
HRLWSHRAYRARLPLRIILALLFTVTGQRDIYTWALDHRVHHKYAETVADPHDIRRGFWF
AHVGWLVLTPHPAVEDRRIALKPTCADLLADPVVRLQKQFFIPLFALLNIAIPIWVPWYCW
NETLINSFVISFVMRFTITLNIAFCVNSFAHLWGNKPYDRFVKSVENSLVSLAALGEGWHN
YHHVFPWDYRTSELGKLNISTGFIDFFAKIGWAYDLKAATTDMISNRAKRCGDGTFGESEE
PYPTSEKCHAE

>*SlitDes3*

MAPNTEKRQVSFPQLEYPIYREAQPKSAQHWLKGKRMQDGAEDLWRIHDSLYDLTDFISS
HPGGSQWIAVNKGTDITEAFETHHLKGIAESLLPNYYVRKATKPRNQPFTFKEDGFYKTLK

LKVMDQIQSIPKDVRKKSDFITDGLLLALIVLAPLSCWGWTQSFIIGASLTLMTSYVLSSVV
TCAHNYFHRGDNWRMYIFNLAGMSFSDWRVSHSMSHHLHTNTAQDIELSMIEPFLQFIPY
KDKPIWAQMGAFYYPLVYATSLLSIMGHELILSATNHEGKTLTWKNLIPFLIPTWMYIMGG
LPLHWTILLWLATMVPASFFFVFYGLTAGHHNHRNFFEGDVPRDENIDWGIHQLDAICERI
DYAGNHFKSITRFGDHALHHLFPTLDHAELKYLYPVLLEHCEKFEFQFKTNTFYETIINAS
KQLIRKRPNNFRDVKATK

>*SlitDes4*

MAHITKTISSKILNKSTHRCLSTAVSQIRIYEVGPRDGLQNEAKFVPTDIKIELINKLAAAGI
KDIESASFVSPKWVKQMSDGVDVMKNIPRVPGVNYPVLVPNLKGYDIAKQCNIEEVAIFP
AGSEAFSQKNLNCSVEEGLKRFKLVADQAVKDGIRVRGYVSCVVGCPYEGPIHPKGIAKIT
EQLFEMGCYEVSLGDTIGVGTAGSVKRLMREVLTVAKPEQLALHFHDTYGQALSNLVAG
LEFGIKTVDSSISGLGGCPYARGASGNLATEDLVYLLYGLGVNTNVDLVKLIEAGRYISNFL
GKPTESKVNRAISDRFKNHSDIVKIASCDI

>*SlitDes5*

MAQTIQTTTILEQKEEKTVTLLVPQAGKRKFEIVYFNLVSFAYWHIAGLYGLYLCFTSAKW
ATILFSFFLFVVAEVGVTAGAHRLWSHKTYKAKLPLQILLMVMNSLAFQNTAIDWVRDHR
LHHKYSDTDADPHNASRGFFYSHIGWLFVRKHPDVKKRGKEIDISDIYNNPVLRFQKKYA
IPFIGAVCFALPTLIPVYGWGETWTNAWHVAMLRYIMNLNVTFLVNSAAHIYGKRPYDKKI
LPSQNIAVSIATFGEGFHNYHHVFPWDYRAAELGNNCLNFTTKFIDFFAWIGWAYDLKTVS
KEMIKQRSKRTGDGTNLWGLEDVDTPEDLKNTKGE

>*SlitDes8*

MGARVSRTDFEWVYTEEPHASRRKIILEKYPQIKKLFGYDPNFKWVVTAMVLVQIISLPFV
TQLSWPLMLVVAYCFGGVINHSLMLAIHEIAHNLAFGHNRPLANKLFGFFANLPIGLPVSIS
FKKYHLEHHRYQGDEVIDTDLPTLIEAKLFCTTGGKLLWLFLQPFFYSFRPLVVRPKAPTP
MELINLVIQLFFDAVIIKLWGWKALGYLLLGAVMAMGVHPVAGHFVAEHYMFKKGYETY
SYYGPLNWITFNVGYHNEHHDFPAVPGSKLPEVRRIAPEFYDTLPHHDSWSKVLYDFVMD
PDIGPYARIKRKHHGLDS

>*SlitDes9*

MPPQGQTGGSWVLYETDAVNVDSEAPVIVPPSAEKREWKIVWRNVILMGLLHIGGVYGA
YLFLTTAMWRTSLFAVFLYICSGLGITAGAHRLWAHKSYKARLPLRLLLTLFNTLAFQDAVI
DWARDHRMHHKYSETDADPHNATRGFFFSHVVWLLVRKHPQIKAKGHTIDLSDLKNDPI
LRFQKKHYLILMPLVCFILPCYIPTLWGESLWNAYFVCSIFRYVYVLNVTWLVNSAAHLW
GAKPYDKNINPVETKPVSLVVLGEGFHNYHHTFPWDYKTAELGDYSLNLTKLFIDFMAAI
GWAYDLKTVSSDVIQKRVKRTGDGSHAVWGWDDHEVHQEDKELAAIINPDKTE

>*SlitDes10*

MPHNTNWEEAAQQRAVDKNTHVSFPQLKYPSLRDEGLRDPVQWLAGKAMDDGAEGLW
RIHDKLYDLTRFIKRHPGGEEWLELTQGTDITEAFESHHLNPSTEKILTQYYIRDAKTPRNSP
FTFKEDGFYKTLKRAAFEELKKIPKDASRTANNITDFLFVSLLISSSLTCWVTNNIAVKFWY
TYASFNLAVLTVACHNYIHRKTNWRMYLFNMSMWSYRDFRVSHVLSHHLYTNTLMDLEL
SSLEPILFYTPRKDKPLHAKLGCITEIFFFPFVFFLSFTKRFLSIFLQQGFFKSHYRWHDAIGL
LLPLWMAITSGAPFLDVISMWLWINCSGSLIFFSIAVNAAHHHPDAIKDGDQPASETPDWG
MHQVEALLDRKDVNGNVFAVMTLFGDHCLHHMFPTLDHSVLKYMHPLFIDLCEKYQAN
YRVSTQFKLVLGQIKETMRTEFKMKND

>*SlitDes11*

MAPNISEDVNGVLFESDAATPDLALARPPVQKADNKPKQLVWRNIILFAYLHLAALYGGY
LFLFSAKWQTDIFAYILYVISGLGITAGAHRLWAHKSYKAKWPLKVILIIFNTVAFQDAAM
DWARDHRMHHKYSETDADPHNATRGFFFSHIGWLLVRKHPDLKEKGKGLDMSDLLADP
VLRFQKKYYLLLMPLACFVMPTVIPVYLWGETWTNAFFVAAMFRYAFILNVTWLVNSAA
HKWGDKPYDKSIKPSENMSVAMFALGEGFHNYHHTFPWDYKTAEFGNNKLNFTTAFINF
FAKIGWAYDMKTVSEDIVKNRVKRTGDGSHHLWGWGDENQPKEEIEAAIRINPKDD
>*SlitDes12*
MLSLYGTYLLLFEVKMMTLLFFMLLTSVALLGMTTGAHRLWAHQTYQASTGLKIMLMLF
QTLAGVGSIYDWVKYHRFHHAHFATDVDPYDYNQGFIHSHLITRLRKLSPHQEKLMQSID
MSDLEKDTVVMFQKKLYWLLYAIIFVLLPLNAPLEYWDDTILCSAFVIGFLRYLVVLHGS
WLIESAISVWGLKPGEKSPPDTNAVFILTKTFWPHYHYLVPYDYKSGEYGTYDGGCSTAFI
RVWAALGLATKLRTVETASIQKALADAARTKKDLKTCIDAAVNNQQLPEEHYLKRA
>*SinfDes1*
GQVAGFGVTAGVHRYWCHRSYKAKLPLQWILMICYSVAGQNTIYEWVRDHRVHHKHSE
TSADPHDANRGFFFSHVGWLMMKKHPDVLKQGVKMDMSDIVNDPLVQFHTRYFLMFKI
SCCWVIPSVIPVLCWNETWLTSIMSQAVLRYLLSLNITWSVNSFAHLWGFKSYDV
>*SinfDes2*
MPPQGQTSGSWVLYETDVVNEDTDAPPPIVPSSAEKREWKIVWRNVILMGSLHIGGVYG
AYLFLTKAMWLTSFFAVFLYICSGLGITAGAHRLWAHKSYKARLPLRILLTIFNTLAFQDAVI
DWARDHRMHHKYSETDADPHNATRGFFFAHVGWLLVRKHPQIKAKGHTIDLSDLKSDPI
LRFQKKYYTILMPLICFAMPMYVPTLWGESLWNAFFVCSIFRYVYVLNVTWLVNSAAHL
WGAKPYDKNINPVEIKPVSLVVLGEGFHNYHHTFPWDYKTAELGNYSLNITKLFIDTMAA
IGWAYDLKTVSTDVIQKRVKRTGDGSHSVWGWDDKEVNQEDKTLAAVINPEKTE
>*SinfDes3*
MAPNIAEDVNGVLFESDAATPDLALATPPVQKADNLPKQYVWRNILLFAYLHLAALYGG
YLFLFSAKWQTDVFAYILYVVSGLGITAGAHRLWAHKSYKAKWPLKVILVIFNTIAFQDAA
MDWARDHRMHHKYSETDADPHNATRGFFFSHIGWLLVKKHPDLKEKGKGLDMSDLRSD
PILRFQKKYYLLLMPLACFIMPTVVPVYFWGETWTNAFFVAAMFRYAFILNVTWLVNSAA
HKWGDKPYDKSIKPSENMSVAMFALGEGFHNYHHTFPWDYKTAELGNNKLNFTTTFINF
FAKLGWAYDMKTVSQDIVKSRVKRTGDGSHNLWGWGDKNQSKEEIAAAIRINPKDD
>*SinfDes4*
MLSQEEPTDTSLVPRAAPRKYQIVYPNLITFGYWHLAGLYGLYLCFTSAKWTTILFSFILCV
IAEIGVTAGAHRLWAHKTYKANLPLQILLMVMNSIAFQNSAIDWVRDHRLHHKYSDTDA
DPHNASRGFFYSHVGWLLVKKHPEVKKRGKELDMSDIYSNPVLRFQKQYAIPFIGAVCFIL
PTVIPVYCWGETWTNAWHITMLRYITNLNVTFLVNSAAHIWGYKPYDENILPAQNIAVSIA
TCGEGFHNYHHVFPWDYRAAELGNNNLNLTTKFIDFFAWLGWAYDLKTVSSDMIKLRAK
RTGDGTNLWGEHNDELKEGKED
>*SinfDes5*
GNRPYDKNIGATDNKTVALCAFGEGWHNYHHVFPWDYKAAELGNYSTNLSTALIDFAAK
HGMAYDLKTVSADMIRQRVNRTGDGTHP
>*SinfDes6*
MAPAQQNIEMCGEEMNAELKLNPHTYKNGKVAQLDNNNTVLRDSASVVTSDSDFDIKKF
EAMEFKAQWRWPDLTVQILLHLVSLYGLYLIFSNQVKLYTILFAFCTIYTSGFGITAGVHRL
WSHRAYRARLPLRILLAILFTITGQRDIYTWALDHRVHHKYSETVADPHDIRRGFWFAHVG

WLVLTPHPAVEDRRIALRPTCADLLADPVVRLQQKLFIPMFAILNIGLPVFLPWYVWNETL
VNSFIISFVLRFTITLNIAFCVNSFAHLWGNKPYDKFVKSVENSLVSLAALGEGWHNYHHV
FPWDYRTSELGKINVSTGFINLFEKIGWAYDLKAATNEMVSKRARRSGDGTFGESEEPYPT
SEHCHSE
>*SlitZ9*
MAPNISEDVNGVLFESDAATPDLALARPPVQKADNKPKQLVWRNIILFAYLHLAALYGGY
LFLFSAKWQTDIFAYILYVISGLGITAGAHRLWAHKSYKAKWPLKVILIIFNTVAFQDAAM
DWARDHRMHHKYSETDADPHNATRGFFFSHIGWLLVRKHPDLKEKGKGLDMSDLLADP
VLRFQKKYYLLLMPLACFVMPTMIPVYLWGETWTNAFFVAAMFRYAFNLNVTWLVNSA
AHKWGDKPYDKSIKPSENMSVAMFALGEGFHNYHHTFPWDYKTAELGNNKLNFTTAFIN
FFAKIGWAYDMKTVSEDIVKNRVKRTGDGSHHLWGWGDENQPKEEIEAATRINPKDD
>*SlitZ9-2*
MPPQGQTGGSWVLYETDAVNVDTEAPVIVPPSAEKREWKIVWRNVILMGLLHIGGVYGA
YLFLTTAMWRTSLFAVFLYICSGLGITAGAHRLWAHKSYKARLPLRILLTLFNTLAFQDAVI
DWARDHRMHHKYSETDADPHNATRGFFFSHVGWLLVRKHPQIKAKGHTIDLSDLKNDPI
LRFQKKHYLILMPLVCFILPCYIPTLWGESLWNAYFVCSIFRYVYVLNVTWLVNSAAHLW
GAKPYDKNINPVETKPVSLVVLGEGFHNYHHTFPWDYKTTELGDYPLNLTKLFIDFMAAI
GWAYDLKTVSSDVIQKRVKRTGDGSHAVWGWDDHEVHQEDKELAAIINPDKTE
>*SlitZ11/E11*
MAQCVQTTTILEQKEEKTVTLLVPQAGKRKFEIVYFNIITFAYWHIAGLYGLYLCFTSTKW
ATVLFSFFLFVVAEVGVTAGSHRLWSHKTYKAKLPLQILLMVMNSLAFQNTVIDWVRDH
RLHHKYSDTDADPHNASRGFFYSHVGWLLVRKHPDVKKRGKEIDISDIYNNPVLRFQKK
YAIPFIGAVCFVLPTLIPVYGWGETWTNAWHVAMLRYIMNLNVTFLVNSAAHIYGKRPYD
KKILPSQNIAVSIATFGEGFHNYHHVFPWDYRAAELGNNSLNFPTKFIDFFAWIGWAYDLK
TVSKEMIKQRSKRTGDGTNLWGLEDVDTPEDLKNTKGE
>*HassGATD*
MAAMSSTSLLLANTMLSSKLQDHDDLRYAEPRKPNRDYEWQVVWRNVLAFVYLHVSAV
YGLYLMLTGKVKSYTILFGALFAIMSGMGVTAGARRLWAHRSYKARWPLRVFLALMQT
MAFQNHIYEWVRDHRVHHKFTETDADPHNAKRGFFFSHIGWLMVRKHKDVFEKGATVD
MSDLEQDPIVMFQKKTYLVVMPILCFVIPAWIPVHFWDENPWTSWYTAAITRYTIALHFT
WLVNSAAHIWGNRPYDKNIGATDNKMVAICAFGEGWHNYHHVFPWDYKAAELGDYST
NLSTALIDFAAKHGYAYDLKTVSADMIRKRVNRTGDGSHPWTKGKVEGDHYHPENPVW
GWEDTDMTEEEKQFAEIVHRKTE
>*HassZ9*
MAPNISEDVNGVLFESDAATPDLALSTPPVQKADNRPKQLVWRNILLFAYLHLAAQYGGY
LFLFSAKWQTDIFAYILYVISGLGITAGAHRLWAHKSYKAKWPLRVILVIFNTVAFQDAAM
DWARDHRMHHKYSETDADPHNATRGFFFSHIGWLLVRKHPDLKEKGKGLDMSDLLADPI
LRFQKKYYLILMPLACFVMPTVIPVYFWGETWTNAFFVAAMFRYAFILNVTWLVNSAAH
KWGDKPYDKSIKPSENLSVAMFALGEGFHNYHHTFPWDYKTAELGNNKLNFTTTFINFFA
KIGWAYDLKTVSDDIVKNRVKRTGDGSHHLWGWGDENQSKEEIDAAIRINPKDD
>*HassZ9-2*
MPPQGQTGGSWVLYETDAVNEDTDTPVIVPPSAEKREWKIVWRNVILMGMLHIGGVYGA
YLFLTTAMWRTCIFAVVLYICSGLGITAGAHRLWAHKSYKARLPLRIMLTLFNTLAFQDAVI
DWARDHRMHHKYSETDADPHNATRGFFFAHVGWLLVRKHPQIKAKGHTIDLSDLKSDPI

LRFQKKHYLFLMPLVCFILPCYIPTLWGESLWNAYFVCSIFRYVYVLNVTWLVNSAAHLW
GAKPYDKNINPVETRPVSLVVLGEGFHNYHHTFPWDYKTAELGDYSLNLTKLFIDTMAAI
GWAYDLKTVSTDVIQKRVKRTGDGSHPVWGWDDHEVHQEDKKLAAIINPEKTE
>*HassZ11*
MAQSYQSTTVLSEEKEPTLTHLVPQASPRKYQIVYPNLITFGYWHIAGLYGLYLCFTSAKW
ATILFSYILFVLAEIGITAGAHRLWAHKTYKAKLPLELLLMVFNSIAFQNSAIDWVRDHRLH
HKYSDTDADPHNASRGFFYSHVGWLLVRKHPEVKKRGKELNMSDIYNNPVLRFQKKYAI
PFIGAVCFALPTMIPVYFWGETWSNAWHITMLRYIMNLNVTFLVNSAAHIWGNKPYDAKI
LPAQNVAVSVATGGEGFHNYHHVYPWDYRAAELGNNSLNLTTKFIDFFAAIGWAYDLKTV
SEDMIKQRIKRTGDGTDLWGHEQKCDKGSGVNDKLS
>*SfruDES1*
MKEKYLEDSPTKVFNNIEKTTCPVIENVDRIPIIKCIATDFSYKHQIVWKNVIGFVLLHVFA
LWGLVLIFTGTALWKTSIWALCVLYIGSEGVTIGAHRCFAHRSFKATPLLRAVMVITQTLAG
QNSVYTWCRDHRLHHRYSDTDADPHNSKRGFFFCHIGWILYKKHPYVKEFGRNIDMSDL
QGDWMVMFQKKYYIILYLVLAILVPVWVPVHYFGETWWNSLFVSYFARYVCQLHGTWC
VNSVAHLYGTRPYDKNLQPVESWFVSFISLGEGWHNYHHAFPWDYKAAELSMHFNLSAS
IIRFLARIGLAYKLKSASTDMVASRILRTGDGSHYKMGTEEARAAVTAWGPLHPLNPTYNT
TLKAPDAILKPGGQPLCY
>*SfruDES2*
MAPAQKHIQMCGEIQSGLKISPLTYDADKLNEAPQYENNNTVLRNSANDKVNSEADFDIT
KYEAIDFKAKLRWPDLTVQVLLHLVSIYGLYLTLSNQVKLLTLLFALGTIYTSGFGITAGVH
RLWSHRAYRARLPLRIILALLFTVTGQRDIYTWALDHRVHHKYAETVADPHDIRRGFWFA
HVGWLVLTPHPAVEDRRIALKPTCADLLADPVVRLQKQFFIPLFALLNIAIPIWVPWYCWN
ETLVNSFVISFVMRFTITLNIAFCVNSFAHLWGNKPYDRFVKSVENSIVSLAALGEGWHNY
HHVFPWDYRTSELGKLNISTGFIDFFAKIGWAYDLKAATTDMISKRAKRCGDGTFGESEEP
YPTAEKCHAE
>*SfruDES3*
MAPNTERRQVSFPQLEYPLFREAQPKSAQHWLKAKRMQDGAEDLWRIHDSLYDLTDFVS
THPGGSHWIAVTKGTDITEAFETHHLKGIAESLLPNYYIRKATKPRNQPFTFKEDGFYKTL
KLKVMDQIQSIPKDARKKSDFITDSLLLALIVLAPLSCWGWTQSFIIGASLTLMTSYVLSSV
VTCAHNYFHRGDNWRMYIFNLAGSSFSDWRVSHSMSHHLHTNTAQDLEISMVEPFLQFIP
YKDKPIWAQMAAFYYPVVYATSLLSIMGHELILSATNHEGKTLTWKNLIPFLIPTWMYIMG
GLPLHWTILLWLVTLVPASFFFVFYGLTAGHHNHRNFFEGDVPRDENIDWGIHQLDAICER
SDYAGNHFKSITRFGDHALHHLFPTLDHAELKYLYPVLLEHCEKFDYQFKTNTFYETVINA
SKQLIRKRPNNFRDVKATK
>*SfruDES4*
MAHITKTISSKILNKSTHRCLSTAVSQIRIYEVGPRDGLQNEAKFVPTDIKIELINKLAAAGI
KDIESASFVSPKWVKQMSDGVEVMKNIPRVPGVNYPVLIPNLKGYEIAKQCNIEEVAIFPA
GSEAFSQKNLNCSVEEGLKRFKLVADQAVKDGIRVRGYVSCVVGCPYEGPIHPKGIAKITE
QLFEMGCYEVSLGDTIGVGTAGSVKRLMREVLTVAKPEQLALHFHDTYGQALSNLVAGL
EFGIKTVDSSISGLGGCPYARGASGNLATEDLVYLLYGLGVNTNVDLVKLIEAGRYISNFLG
KPTESKVNRAISDRFKNHNDIVKIASCDI
>*SfruDES5*
MAQTIQTTTILEQKEEKTITLPLVPQAGKRKFEIIYPNLITFTYWHIAGLYGLYLCFTSAKWA

SILFAFFLFVVAEVGITAGAHRLWSHKTYKAKLPLEILLMVMNSIAFQNSAIDWVRNHRLH
HKYSDTDADPHNASRGFFYSHVGWLLVRKHPDVKKLGKKLDMSDIYNNPVLRFQKKYAI
PFIGAVCFGLPTLIPVYCWGETWTNAWHITMLRYIMNLNVTFLVNSAAHIYGKRPYDKKIL
PAQNIAVSIATFGEGFHNFHHVFPWDYRTAELGNNSLNFTTKFIDFFAWIGWAYDLKTVSN
EMIKQRSKRTGDGTNLWGLEDVDTPDDLKNTKGE
>*SfruDES6*
MGNSKEELGFVSKVREFEKWMGFQTKIKWTVAFIALAYHILAVYWCYHYTLPVKWQTV
LFAYMMFVFSGFGITGGAHRYWAHRTFKASTPLRIIMILGFASAGQNTLRQWVRNHRVHH
KYSDTDSDPHNRERGLFFSHIGWLLMHKNSAVVSKFNEIDMSDIDNDPIVKWHTKYVDIV
NPLACAVIPTLVGILLWGEDWRVAIAWQCFIRMMAVYHSELTVNSLGHTLGYKPYNQSIRP
AENVFIAAITGGEGWHNFHHAFPFDYKTSELPHGIDTTTRLIHLFETFGWVQDKREVSPET
IKKYSLQHCEGIEQIKMAEKEF
>*SfruDES7*
MAAMSGAPLLLANATLTSKLQDDDHRYAEPMKKNRDYEWQVVWRNVFAFVYLHAAAL
YGFYLMFTGKVKIWTILFGLLFAIMAGMGVTAGAHRLWAHRSYKARWPLRLFLALMQT
MAFQNHIYEWVRDHRVHHKFTETDADPHNARRGFFFSHIGWLMVRKHKDVFEKGATVD
MSDLEKDPIVMFQKKTYMVVMPLLCFVVPAWIPCYFWGENAWYSWYVASITRYTVALHF
TWLVNSAAHIWGNRPYDKNIGATDNKAVAICAFGEGWHNYHHVFPWDYKAAELGNYST
NLSTALIDFAAKHGLAYDLKTVSAEMIRQRVNRTGDGSHPWTKDSQEEEHYHPENPVWG
WEDTDMTEEEKQFAEIVHRKTE
>*SfruDES8*
MGARVSRTDFEWVYTEEPHASRRKIILEKYPQIKKLFGYDPNFKWVVTLMVLVQIISLPFV
VQLSWPLMLVVAYCFGGVINHSLMLAIHEIAHNLAFGHNRPLANKLFGFFANLPIGLPVSIS
FKKYHLEHHRYQGDEVIDTDLPTLLEAKLFCTTGGKLLWLFLQPLFYSFRPLIVRPKNPTP
MELINLVIQLFFDAVIIKLWGGKALGYLLLGSVMAMGVHPVAGHFVAEHYMFKKGYETY
SYYGPLNWITFNVGYHNEHHDFPAVPGSKLPEVRRIAPEFYDTLPHHDSWSKVLYDFVMD
PDIGPYARIKRKHHGLDS
>*SfruDES9*
MPPQGQTGGSWVLYETDAVNADTEAPVIVPPSAEKREWKIVWRNVILMGLLHIGGVYGA
YLFLTTAMWRTSLFAVFLYICSGLGITAGAHRLWAHKSYKARLPLRLLLTLFNTLAFQDAVI
DWARDHRMHHKYSETDADPHNATRGFFFSHVGWLLVRKHPQIKAKGHTIDLSDLKNDPI
LRFQKKHYLILMPLVCFILPCYIPTLWGESLWNAYFVCSIFRYVYVLNVTWLVNSAAHLW
GAKPYDKNINPVETKPVSLVVLGEGFHNYHHTFPWDYKTAELGDYSLNLTKLFIDFMAAI
GWAYDLKTVSPDVIQKRVKRTGDGSHAVWGWDDHEVHQEDKKLAAIINPEEKTE
>*SfruDES10*
MKMPPNADWEEVAQQRAVDKNTHVSFPQLKYPSLRDDGLRDPVQWLAGKAMDDGAEG
LWRIHDKLYDLTRFIKRHPGGEEWLELTQGTDITEAFESHHLNPSTEKILTQYYIRDAKTPR
NSPFTFKEDGFYKTLKRAAFEELKKIPKDASRSANNITDCLFVSLLVSSAMACWVTNNYA
MKFWYTYASLNLAVLTVACHNYIHRKTNWRMYLFNMSMWSYRFLSIFLYRGFFKSHYR
WHDAIGLLLPLWMAIASDAPLLDVISMWLWINCTGSLIFFSIAVNAAHHHPDAIKDGDQPA
NETPDWGMHQVEALLDRKDVNGNVFAVMTLFGDHCLHHMFPTLDHSVLKYMHTLFIDL
CEKYQANYRVSTQFKLVLGQIKETMRTEFRVKND
>*SfruDES11*
MWFAILLDCFKLLTFIYCARKCGKMAPNISEDVNGVLFESDAATPDLALARPPVQKADNK

PKQLVWRNIILFAYLHLAAVYGGYLFLFSAKWQTDIFAYILYVISGLGITAGAHRLWAHKSY
KAKWPLKVILIIFNTVAFQDAAMDWARDHRMHHKYSETDADPHNATRGFFFSHIGWLLV
RKHPDLKEKGKGLDMSDLLADPILRFQKKYYLLLMPLACFIMPTVIPVYLWGETWTNAFF
VAAMFRYTFILNVTWLVNSAAHKWGDKPYDKSIKPSENMSVAMFALGEGFHNYHHTFP
WDYKTAELGNNKLNFTTAFINFFAKIGWAYDMKTVSDDIVKNRVKRTGDGSHHLWGWG
DENQPKEEIEAAIRINPKDD
>*SfruDES12*
MYNDLSRSCALSLCCVHRLRLPASNSSKMVEVKEAAPVAGEPKLSKSREANWPAVLFFIHI
HLLSLYGTYLLFFEVKMMTALFFIVLTSVALLGMTTGAHRLWAHQTYQASTGLRITLMLF
QTLAGVGSIYDWVKYHRFHHAHFATDVDPYDYNQGFIHSHFLTRLRKLSPHQEKLMESID
MSDLEKDSVVMFQKRLYWGLYAIIFLLLPLNAPLEYWDDSILASAFVIGFLRYLVVLHGT
WLIESAISVWGLKPGEKSPPDSNTVFILTKTFWPHYHYLVPYDYKSGEYGTYDSGCSTAFI
RVWAALGLATKLRTVETVTIQKALADAARSKKDLKTCIDAAVNHQQLPADHYLKRA
>*SfruDES13*
MAPITFTETEVIEQPIHETDYIQYKLYQPTKDSRDTNGKSNGESCELSPYDQMLSPKDPKFL
APIRKLEKSLGFVTPIRWFNTISISLFHIGAVLWMIRYIFLNKMPIKWQTTAFGYLLGQVAGF
GVTAGAHRYWCHRSYKATKPLQWILILCYSVAGQNTIYEWVRDHRVHHKFSETSADPHD
ANRGFFFSHVGWLMMKKHPDVLKQGVKIDMSDIVNDPLVQFHTRHFWLFKIMCCWVLP
SLVPALCWNDTWEASIMSQVVIRYLLSLNFTWSVNSFAHLWGNKPYDKNIMPVENWGVS
VVAMGEGWHNYHHTFPWDYKAAELGIPMNLTTTLLNYFASIGWAYDLKEASPGLVRAVA
KARGEPAED
>*SfruDES14*
MTIQSNSLGFGSTENGSKQDGKVNSLPTHPKKWDIVWRELIIFTIFHITAVRGLYLFITVAK
WQTCIFAFLLYIATTTSVTVGAHRLWSHKAFQAKLPLKVILLFFFTMAFQYSAVIWARLHR
MHHKYLDTDADPHNAGRGFFFSHIGWMLIKKHPEYRAHKVDISDLMQDPLLRFQHKHY
WEVLFIAFFLVPTYVPTLWGEQSSTAFYTCVCLRYVCGLHSIWLVNSAAHMWGTKPYDK
NIRAVETKIVSMLAAGEGFHNYHHTFPWDYRIAEFGDYSLNLGKLFIDTMAKIGWAYNLK
SVPDELIEKRVKRTGDGTHPVWGWDDPDLPLEDKRMSIVK
>*SfruDES15*
MAPKDSSYIEWAHKTAAEKKTHVSFPQLKYPSLRDGGLRDPIQWLEGKAMDDGAEGLW
RIRNSLYDFSEFLDKHPGGAEWLELAKGTDITEAFECHHLNPVVEKVKDKYYVRDAKTPR
NSPFTFEEDGFYKTLKRSVAEELKKIPASERKRTTLIIDGLVATLLISSALSCWATNYWVVM
VSYLVASLTLAWTTVAAHNYIHMKTNWRMYLFNLSLWSYRDFRVSHALSHHVYPNTLID
LEISGFEPIVTWIPRKKPAFAKFTLLIETIVFPFLFILNFLKRFISNFIRKGFFTRHYRWHDVIG
LLLPVWMYLASGCTFYHAFITWLWINCTGSFIFFTIGSNAAHHHPNIFKDGDQLKEKTVD
WGMHELEAVMDRNDINDNFFKVMTFFGDHALHHLFPTLDHAVLRHLYPVFLKHCEEFRA
NYRLTSQYDFFIAQLKMAVKEDPTVLDESS
>*SfruDES16*
MPPQVDPVPSGVVFESDTQTSDLGLAKDVSVLKNASPRKHEYVWFNIFWFLYLHIASLYG
LYLVFTSAKWQTNVFAFAVHLMCAIGIGAGSHRLWTHRSFKARTPLRIVLMLWQTMGFQD
CIFEWARDHRTHHKYADTDADPHNAERGLFFSHMGWLCCKKSPEVIEGGKRIDLTDLYAD
PVVMFQKKHYMKMMPLLCFVLPTVIPVYFWGETWTNAFFIPTILRYTCGINVVWSVNSFA
HTFGYRPYDKSLNPRENIGVWMICVEGFHNYHHTFPWDYRATELPLYNMLTPTIVFIDLM
AKIGQAYDLKYVSPEIIKQRAHRTGDGTHHLWGWDDPEFTEKLKEKYGAVSHSEENRKL

A
>*SfruDES17*
MTAANMETKSPSSNDLKNPMKDGNVNKEETMEIPKVIGTDYSYKHTIVWKNAIGFLIMHI
LALWGVGLLFAGYMKWQSLVWTLFIAFLSTEGVTIGAHRFYTHKSFKATPFLKAIFLLCQT
IAGQNSMFIWCRDHRLHHRYSDTDGDPHNSKRGFFFCHIGWLMHKKHPYVIELGRKIDM
SDLQADWMVMFQKKYYYHLYFLLAFLIPVWVPVHFFGEPVLHSILVVYFARYVLQLNGT
WLVNSAAHLYGTRPYDAKLQPVESWFVSFISLGEGWHNYHHAFPWDYKAAELTMHFNQ
SASIIRFFERIGLAYDLKTASPELVTNRIIRTGDGSHYKMGTEEARSAVTAWGPVHPLNPTY
NTTLKAPDAILKPEGLPLYHEKDTLNLTVIQKNSRRG
>*AperDES1*
MKIIKFDEILRRFERVFAFDNDIKWGSVFVIFGYHALAVYWCYNYSLPVKWQTVIFAYCTY
VLSGMGITLGVHRYWSHRCFKATKPLQIILFLMFALTGQTSIRIWVRNHRLHHKASDTPGD
PHNATRGLFYSHIGWLMMKKTDQLIEQGKKIDMRDIDSDPLLRWYDRNVFMVNLMAAF
VLPTMVNIWCLGESWRCAVAWQCFVRYLGVLHAQMTINSLAHKFGNRPFNSRIKPAENM
FVSYITFGEGFHNYHHVFPYDYRTTGDYFILDYGKILLKFFEKIGWAYNLRKASPETIASAV
GRLNCAESDDLLKS
>*AperDES2*
MAPNTKKEDELITCEGSIEKLIAPQANQRKHQIVYMNLFTFGYWYIAGLYGLYLFFTSTK
WSTIILNIFLIIASGLGITVGAHRLWTHKCFKAKLPLQIILMIFNTLAYQYTATTWVRYHRM
HHKFADTDADPHNATRGFFFSHIGWLLVKKHPEYLKRRKNIDMSDIYNNPVLYFQQKYAK
PLMATITFILPTVVPMYFWEESLSNSWHTTILRYTSNWNFMFLINSAAHRWGYRPYDKSIQ
PTQNMLATVCTLGEGFHN
>*AperDES3*
MAPNITDANGVLFESDATTPDLGLPIQPIQQADNRPKQFVWRNIILFAYLHIAALYGGYLFL
FHAKWQTDIFAYILYVMSGLGITAGAHRLWAHKSYKAKWPLRLILVIFNTLAFQDSAIDWA
RDHRMHHKYSETDADPHNASRGFFFSHIGWLLVRKHPELKRKGKGLDLNDLYADPILRFQ
KKYYLLLMPIACFILPTVIPVYFWSETWSNAFFVAALFRYTFILNVTWLVNSAAHKWGDK
PYDKSIKPSENISVSVFALGEGFHNYHHTFPWDYKTAELGNNRLNFTTNFINFFAKFGWAY
DLKTVSDEIVQNRVKRTGDGSHHLWGWGDKDHDKAEINAAIRINPKDD
>*AperDES4*
MEEVNQTVETDSSAIKLSKSREANWPSVLFFIHIHLLSLYGLWLLFYEVKWMTLVLLVILA
SIAILGMTTGAHRLWAHGSYKASTGLRITLMLFQTLAGVGSIYDWVLYHRLHHAHFATDD
DPYDYRKGFLYSHMLTRMRKLSPHQEKLKESIDMSDLENDSVIKFQKILYWPLYAIIFLLLP
LNAPLEYWNDSVLSTVFVIGFLRYGIVLHASWLIESAVCLWGLKPGEKSPPDSNSVFIITKT
FWPHYHYLIPYDYKSGEYGTYDSGCSTAFIRVWAALGLATDLRTVESKTIQKALASVAKT
GRPLQTCIDEALKDQSLPDQHYLKRG
>*AperDES5*
MPPQNQQQDSWVVQESDEVNGEADISTLVPHTAEKRKLQIVWRNVILFTFLHIGGVYGAY
LFLFKAMWATSLFAIVLYLCSGLGITAGAHRLWAHKSYKARLPLRVLLTAFNTIAFQDSVL
DWARDHRMHHKYSETDADPHNATRGFFFSHVGWLLVRKHPQIKAKGHTIDLNDLWSDP
VLRFQKKYYLLLMPFACFVLPSYIPTLWGETLWNAFYVCAIFRYVYVLNVTWLVNSAAH
MFGDKPYDRNINPVEIRPVSLVVFGEGFHNYHHTFPWDYKTAELGDYSLNFSKLFIDLMA
KIGWAYDLKTVSSEVIEKRVKRTGDGSHPIWGWDDKDVPIEDRNIATIIKSEKSQ
>*AperDES6*

MAPAQQNVEVCTETVDSQIKMRLKQSDFKCTNGVTSCENNNTVLNDSVSEKLISDTDFDL
MKYEAMEFKPQIRWPDLTVQVSLHLTTIYGLYLILTNQVMILTTLFALLTIYTSGFGITAGV
HRLWSHRAYKANTPLRVILALLFTITGQRDIYTWALDHRVHHKYSETAADPHDIRRGFWF
AHIGWLVLTPHPAVEDRRVALRKTSLDLMADPVVRLQKIFFIPLFALLNIALPVWIPYYFWN
ESLTNSFIVSFVTRFTITLNIAFSVNSFAHLCGNKPYDRFIKSVENGMVSLAALGEGWHNY
HHVFPWDYRASELGRLNISTTFIDTFAKIGWAYDLKAATPAMIISRAQRSGDGTLGEMEEP
EPTSLDPSSSSD

>*OnubZ/E14*

MADIDATNPYDLQDKSEDDKPEDTEGELTIVGTDYSYTHRIIWPIVMVYVVMHIGAITGLL
LVLGGNVKIASIIWAVFYSLVATEGAHMGAHRCFSHRAFKAKPLLKVILLIMQTISGQHSTY
IWCRDHRQHHRYSDTDGDPHNSKRGMFYCHVGWLMTSRHPLCKKLRKTIDMSDLQQDP
LVMFQYRYFRSLFFTFGFLLPVWVPMHFFQESFTNAVFVCFFLRYVYALHVTYFINSLAHK
YGTRPYDKTIQPVETWFVSLLSLGEGWHNYHHAYPWDYKAAEIGMPLNSTASLIRLCASL
GLAYDLKSVDPETLNKRIMNKGDGTYEVKYLQEHVTAIGPLHPLNPSYRGTCPDPEIKLK
VRMKP

>*OnubZ9*

MAPNIKDGADLNGVLFEDDASTPDYALATAPVQKADNYPRKLVWRNIILFAYLHLAAVYG
AYLFLFSAKWQTDIFAYILYVISGLGITAGAHRLWAHKSYKAKWPLRLILIIFNTVSFQDSAL
DWSRDHRMHHKYSETDADPHNATRGFFFSHIGWLLVRKHPELKRKGKGLDLSDLYADPI
LRFQKKYYLLLMPLGCFIMPTVVPVYFWGETWTNAFFVAALFRYTFILNVTWLVNSAAH
KWGHKPYDSSIKPSENLSVSLFALGEGFHNYHHTFPWDYKTAELGNNRLNFTTNFINFFA
KIGWAYDLKTVSDEIIQNRVKRTGDGSHHLWGWGDKDQPKEEVNAAIRINPKDE

>*OnubZ/E11*

MVPYATTADGHPEKDECFEDNEIKSNSLPKLEILYFNVMTFTFLHLSALYGLYLGFTSVKW
ATIGLGIIFYFFAEIGITAGAHRLWSHRSYKAKLPLEILLMVFNSMAFQNTALSWARDHRVH
HKCPDTNGDPHNANRGFFYSHVGWLMTKKSDEVIKQGKLCDVADLYSNPVLRFQKKYA
VPFIGTLCFVLPTLIPMYFWGETLNNAWHFNMFRYVINLNATFCVNSVVHKWGYKPYDK
NICPTQNVLLNLAVLGEAFHNYHHVFPWDYRAAELGNQKMNPTTLFIDFFAWIGWAYDL
KTASKEMIKSRSERTGDGTDLWGHSADKLK

FARs

>*SfurFAR1*

MAVETLTETQMFEPKQGGDITYMDMVEESQPLGDSQIQKLFAGSSVLLTGGTGFLGKLVV
EKLLRSCPDLKKIFLLARPKKNKTITKRLQEQFDDVLYDRLRKECPDFINKISIVEGDVGQL
DLGMCPEDRIKIMNEVEVIFHGAATVRFDEPLKTAVEINVRGTREMFKLARGCSKLKAFV
HISTAYSNCPQNMIGEKFYESPLPGDKLIDLVETMEEKVINNITPGLLGDFPNTYAYTKAVA
ENIVKEYSKGLPVALFRPSIVIGTSKEPVSGWIDNVYGPTGVVVGAAVGLLHVLNCNPKV
KADLVPGDMVVNACIATAWKTAKEYPSNHEDAPPPDLTPPVYNYVSSEQRPLTWEKFMN
YNEVYGFQVPTVQAIYYYLFHLTSSKFLYNLYCFLLHWIPAYIIDGVAVIIGKKPMLRKAYK
KITKFSEVMAYFATREWKFDNTNTQQLFKELCPADKHLFDFDMSALKWNEYFYNYIRGV
RVYLLKDPVETVPQGIKKLHRLKYLHYTFCAILGLLFLRLLWAMLSGVFSF

>*SfruFAR2*

MTYRRVSECDAEIITQYPDPVSYVSVPDFYAGKSIFLTGVTGFLGKVFLEKLLFSCEDIETIY
ILIREKKGKTPLQRIEELFDKPLFSRLKEKDPQCKKKVIPIIGELSEPGLGISKANEETLLEKV
SVVFHAAANVQFDKELKEIINTNVCGTKYVLQLCQRMKNIKAFVHVSTAYCHTDQKVLE

ERVYPPPAELSEVLKFLHQPQHDNKKQIKEFFKEQPNSYTFAKALAETYIAENCGHVPTIII
RPSIISSSLKEPLPGWLDTWNGATGLITASYNGANRVLLGEGSNILDLIPVDFVANLAIVAAT
RCTSPLKVYNCCSSGCNPLTLKQLISHMNHYGCDKNVSIIFTNNKPSMSTFTFLLQTTPSFT
ADMFLRATGKSPKYMKIQSKLTLAQKALSYFTSHSWVMKADNSRKLYASMSIQDRQKFP
CDPTDIDWKQYITIYLEGINKFLMKNYT

>*SfruFAR3*

MVVLTSKEKSNMSVADFYAGKSVFITGGTGFLGKVFIEKLLYSCPDIDKIYMLIREKKGQSI
RERMTKIVADPLFNRLKDKRPGDLDKIVLVPGDITVPGLGISEENETILTDKVSVVIHSAAT
VKFNEPLATAWNINVEGTKMIMALSRRMKRIEVFIHISTAYTNTNRAVIDEVLYPPPADINE
VHQHVKNGVTEEETEKILNGRPNTYTFTKALTEHLVAENQAYMPTIIVRPSIVGAIKDDPIR
GWLSNWYGATGLTVFTAKGLNRVIHGHPNHIVDLIPVDYVANLVIVAGAKTYHSNEVSIY
NCCSSSCNPITMKRLVGLFIDDAVKHKSYVMPLPGWYVYSNYRWLVFLITLIFQVIPAFLG
DIGRRLLGKSPRYYKLQNLVAQTQEAVHFFTSHTWEIKSKRTRELFSSLSLTDQRMFPCDA
NRIGWTDYITDYSSGVRKFLEKKK

>*SfruFAR4*

MAPSMSIAEYYAGKTLFITGATGFMGKVMVEKLLRDCSDVKKMYLLMRPKKGHSSKER
LDELLNFKIFDRLKAENPKLFEKLQVVAGDILSEDLGLSAEDRLLIQEEAQIIFHCAACVRF
DMFLRDAVKMNTMGTKKVLELAEGVKNLEAFVHVSTSYCRCELPLFEEKLYPSKHRPEH
VMHCVNWMDDELLGHLQPKIIEPQPNTYAYTKSLTEDLVSQYEGKFPIVIARPSIVAAAYK
EPLPGWVDNLNGPTGLLVGAGKGVIRTMHCNENYEADVVPVDVTVNACIILGYLTGMER
PKKINFCNITQSQINPITWGQALDMGRVHVQEFPFTVCLWYPGGSAKSSWIAHQFALFFTH
MLPAYFVDLLMFLMGKKTFMIKIQKRINYGLEVLQYYTTKEWHFTNDYFVSLQHRISERD
NEVFYTNMKEMDWSQYIRNYIKGAREYCCKEDPSTLPAARRLQKQLYYLDKAVQIMVGL
LVSYFIYYYFNMLYSVISS

>*SfruFAR5*

MDPALAVEMEALSRQKAMFEATERGDSTVQQFYKDSTVFLTGASGFLGKQLVEKLFRAC
NIRKIFILLRPKKNMTIQERLEEMLQDPVFSLVKKKKPNFAENIVPVKGDVAEIKLGLSDED
WTMITNEVDIIFHVAATTRFDEALRISTMINIRGTREAVLLGKDCQKLKSFVYVSTTYSTAT
QANVDKEVMERFYPCPLPPELMVDMAENIDDERMDAIEANLIKGYPNTYTFTKSIAEEVV
RSRAGGMPTCIIRPAVVISSYREPVPGWADASCAFGASGLILGPATGLIHAIYASNDVKFSLV
PVDYVNNAILVAGWHTATNRPNDVQIYSVSSARNLFHWEPISSKIRDIGKVLPTPLAVWYT
FIVNTSNKPLFFLLTWLLHYIPGYILDACCILLGKPTMFIKLYNRVNRSSLALSYFTTHTWV
FNDTNSDKLFNSLSKTDRLIFNFDTTDINIPEFVTLWCVGLRKYLMKDGIKNTEYAKRKQV
LLKYLHYVVSCLYVYVLFKVACFVCYLILCLFGFV

>*SfruFAR6*

MVPRPAPQTATPPLIPEYFAGREVLITGATGFMGKVLVERLLWTCPDIGRLHLLMRHKKDC
APDKRLALLKQSQVFDVVREQCPQQLDKLCVLPGDVTKTRFGLDQVALHQLNQVSVVFH
SAATLKFDEPLAAAAEQNVRPVLTLMDICDQLANVQVFIHVSTAYSNAELCAVEERVYAA
PLAPRQLLALVDALPPALLQEITPRLIAPKPNTYTFTKAVAESAVSEHAPTAHYACAIFRPSI
VVSSLRHPFPGWIENLNGPSGVVAGAGKGLLRVLRCAARRRADMMPVDICIDTLIAVAWE
TGVDDSREARVYQCASSAHAATWGQFRARMLRLVRAHPFDGALWYPWGVICENTVIQK
ALETVLQTAPLCLAHCVLKACGLKQKPSLWTACKRLQAMNQALQFFATRHWEFGTARVQ
RLADRLHAEDTLRYNLRPETIDWEQHCVDFVKGTRRYLLRERDEDIPAARRRMKLLTVV
HNATLLFAIFIMCRFTVRTAPAILRGIAVLTRLRNKTALAS

>*SfruFAR7*

MSNPTIRDFYKGRNILVTGGTGFMGKVLIEKLLYSIPDIGNIYILMRPKRGKSVDQRIEDMQ
RLKLFERIRTEKPDALKKMKALQGDVLFEKLGLSESDIELLSNEVSVVFHFAATLKLEAPL
KDNVNMNTCGTQRALDIVKKFKKLDVFVHLSTAFCYPDYEVLGERCFAPPIKPENVMKLI
QWLDDKQLALLTPSLLGPHPNCYTFSKRLAESIVEQAYAELPVVIARPSIVCPSLKDPVPG
WVDNLNGPIGLMLGAGKGVIRTMLCDGSLIAQVCPVDIAINGIIAIGMIEGNRKEKPTSLP
VYNVNNGHQKPTTWGEVLQIAKDYGRKFPLSWPLWYPNGDITTNAVLHEYRRIFYHLVP
AYLIDFLLFVLGQKRIMIRIQERISNGLEVLQYFTMRPWNFPCPNYDAIKDKLNPEEQEIYN
TDITEADREVYLMNCVEGGRVYCFKEDPTKVPYNRIYHRFLYFLDWFVKIMFWLFVLSFL
ASWFEPIKALFSYGEPIVKHLPFLGKAVCQVEEL

>*SfruFAR8*

MASETISSAELEYLPDRIADTFSGMKVLITGGTGFMGKVLVEKLLRKCPDIDQILLFVRSK
KGKNPKQRLEEIFSGALFDQLREMRGGVEPLLEKVTLINGDVSEPDLAMSPEDRQMIINQ
VDIIIHAAATIRFDEELKKAVLLNVRGTKLMVELGKACQKLKVFIHISTAYCHLHEKLLEEK
AYPPPADPHQIIQAVEWMDEETIATMTPKLLNKLPNSYAFTKALAEALVVEAMEKANLPA
MVLRPSIVIPIWQEPVPGWTDNINGPTGLLIGAGKGVIRSMYCKSNSYADYLPVDVFINGI
MIVAWNYIKNGDTKCNIINFTSSAEIKVTWSEMIDAGREIIMNRVPLNGVVWYPGGSMKH
SRLYHNICVFFFHWIPAFIIDTLLFCLGYKPVLCRVQRRITKGFEVFEYYTNNQWDFKSDIA
QTLRQKLNAKERRDYKVDAVGLDISKYFEDCIRAARIFILKEYDDTLPAARRHMRIMYWV
DVIVNCLFWGFLLYWLSGWMTTTKAIVPDTASPTVIAMDA

>*SfruFAR9*

MFNLCKMNGVQRSVGRNCVVFNKIVVVKPIRGYSNASSVISKDKYQSVADFYSGKSVFM
TGGTGFLGKVYMEKLLYSCPKLDKLYLLIREKKGVSADKRIADLFENPLFKRLKSSNPDF
MKKVVLVPGDLGLPNLGISPSDEQTLIDKVSVVYHAGATVRFYEPLPVSININFDGTRKML
ELSQRIKNIEAFVYVSTAFAQANAKVLVETAYPSPAKVEDVYKFIEEHGHDKHAVKKFIGN
HFGTYAFAKALSEAYIAKNHGKVPAVIIRPSAVTATKDGPVKGWLDNWFGASGILYYINKG
VIRILHGHGNHTVDFIPVDYVSNLCIISALRAKESNEVQVYNSTSTSDNPVNWDTIFNTMK
EEKTLSGKNKFPYLSMTYVDSKLALQLGTFFLQITPAVLADLWLKIKGKDPKYLKMVSQA
LTIRNAYDYFTTNNFILRSDRTRELHSSLSPEDREEFPCDPTQIRWPEYLKDNMNGIYKYLK
PSKIL

>*SfruFAR10*

MSSATQNASLAQTPVTHEKMEKWVDAQVKGEKVDIDVYGKPTDKMLKELENVRNLSKE
LQDNLHELENSVRIAEVENQAMNPTAQILDFSEDHEFVPDKKDTYYAEEDKLDAKEEEKR
KLTKGKGPKTEIQEFYKDQCVFLTGGTGFLGKVLIEKLIRSCGDINTIYVLARNKKGKDPR
VRLHEMMDDFLFHRAHEENPKGIHKVVPILGDMELPGLGINEEDRKMLASKVTIIINAAA
TVKFDEKLSVSTAINVKGTKEVLKLAKECRNLKAITHVSTAFSNTQVKHIEEKFYEPPMSV
EALEAISEVDEKLVESILPTLLGTRPNTYCFTKAVAEEAVRTYGEGLPICIVRPSIVVSTYEEP
VRGWTDSVYGPTGLVIGIGTGVLRTMYMDQEKVADMVPVDLCVNAILASAWYTARNYK
ENQTSHIPIYNFVSGAQNPLTWGEFIARNRKYGIDKPTTKAVWYYGLNPTNNYYMFLFYN
FFLHYLPALMIDTYCAVTGKRRAMLKLYSKVMKLANILFYFSTQDWKFSDMNVRNMWN
SLSDADRVVFPFSIGEMSWEYMCETFLVGLRVYLIKDDLSTLPEARKKWNKLFYLHQVLK
IITLSLVLYLTYFVLQPIIALVFN

>*SfruFAR11*

MSAPEPGEGPLLPGFYAGRAVLITGGTGFLGKVLIERLLYTCPEVGEVHLLLRDKRGQPPR

VRLNQLKQSQAFDKVRAQCPGQLDKLRVVCGDVAQPRLGLDDAALQQLREVSVVFHSA
ATVKFWESLETALHQNVTSVVALMDLCDQLPKLEALVHVSTAYSNAERRNIEERVYEAPA
QLAGLRAMLDALPPSLLDDLTARYIAPKPNTYVFSKAVAEATIAQRPRKHYATAIVRPSIVV
SSHRHPFPGWIENLAGPSGVVVGCGKGLVHAFNLDLAARADLIPVDITIDTMLAVAWEIAT
DKSGEVRVYNSCSQQNPITWRAFRDRVVRNVRAHPFDHLMYYPFTFGIKNRYVYKTLEL
VLQTVPLHVADYLARLCGVKLQLSLVTVSERLQAMNRVLAFFATREWYFSTRNVHALRR
RLTRADRDIYNLDVCSVDWDEHVRDFVKGTRKFLLKEKDDNIPRARRFVERLRRVHQFV
LLVLSALLYRLLTLLLPRFLFRSAPALAGPH

>*SfruFAR12*

MADPSQVRSFYAGKNIFITGGTGFVGLCLIEKILRCIPDAGKIYLLMRPKKGKEIADRLQEF
PKNPVFEKLLESNSTDIFKKLIPIAGDVGEENLGLSPQDRQTIIDNVNVVIHSAATLDFQESL
RPTVNINLLGTRRVMQLCKDAKNLKVMIHVSSAYVNSYLTEAHEKVYEAPEDPEKVISLV
GTLNDEGLLEVEPKLLKSHPNTYTFTKHLAEHEVVKCADLFPCTIVRPTMIVASWKEPVP
GWTCSKVGPQGFLMGAAKGVVRRLPLAKENVADYIPVDVVVNQLLVAGWEAANSRSGL
TVYHCSSSTCHPFTWSMLDDTVNSMLHKYPLKSAVWYPYLKFVPSLLMFRISAIFVHFFP
ALLLDLMLRMTGGRPILIRLHKNVWNSLNRLERFIFSEWKFHNPNTLELATKLNQTDKEL
FFIDISKLYWVEYFKTLHLGVRRYLNKEKESSLPAARKKDTVLLLFHVIWQLVIMGLVWY
VFACATGLTLAHSAWVAPIIYILFTCL

>*SfruFAR13*

MAPNVLEYYRGKNIFITGGTGFVGKALVEKLLRSCSEVNAIYLLMRQKKGVSTEERLKDL
CSKPVFDMVREKNPSGFKKLRIINGDITEPGLGISEEDVRLLQKECNIIFHSAACVRFDQKL
KDAVNMNTSGTLRMLTLAESMQNLEVFVHLSTAYCRCDLDVLEEKVYAAVHKPRKIMDI
VEWMDNDTLDHLEPKIIESEPNTYSYTKAITEDLVNEYSGKFPIAIARPSIVTAAWKEPIPG
WVDNLNGPTGIVIGSGKGVIRTMHCEPSYKADAISVDVVANACILIAYVTGLDKPKETQV
YNLTLSGVISLTWQEIIKLGEKWVNEYPYTMALWYPGGSIKSYNFTHQIDKFFSHLVPAYL
VDALLFLLGKKTFMINLQKRISHGLNVLQYYTTKEWHFRNNNYKALRTRVSPEDNEEFYT
DASTLNPDEYLKNYVLGTRKFCCHEDPANLPRARKLHRIRYFADRLFKLLFIILVLWTLYS
NSNVFTSSVELLDNSLKSLPLMNQANAEEIPNIAL

>*SfruFAR14*

MASCLSGGHYVPGSKEYVPVAEFYADKSVFVTGGTGFMGKVLVEKLLRSCPKIKKIYLLM
RPKRGQDVASRLTELTQSPLFETLRRERPQELNKIVPIVGDITEPELGISAADQTMLCQKVS
VVFHSAATVKFDEKLKLSVTINMLGTQQLVQLCHRMLNLECHVVDSEYCNPDVTRDEW
KEYGVLLPPAHPEHVVTLVQTLPDDLVDRITPDLVGDRPNTYTFTKALAEDMLIKESGNLP
VSIVRPSIVLSSLREPVKGWVDNWNGPNGIIAAVGKGIFRTMLGTGTKVADLVPVDTVINL
MIVCAWRTHLRRGEGVVVYNCCTGQQNPITWQRFVKTSFKYMRKHPFNEVLWYPGGDI
TSNRLKHGTLSLLQHRAPAAIMDLVSTATGKKPMMVRVQNKLEKAAACLEYFTTRQWAF
ADDNVQALCASLSPDDRRTFDFNVRNIDWDAYIESYVLGIRRFLFKESPDTLPKSRAVLRR
LHIVHILTQVATVFFLWRFLFSRSNALRSIWRRVLELLTRAARLLAIA

>*SfruFAR15*

MGFLEERDLCEVPTIPEFYKGKTIFITGGSGFIGKVLIEKLLYSCSELDRIYLLMRNKKGVK
SEDRLAQLYATPCFDRLKKKKPNVFNSKVFIVSGDVLEPGLGLSQEDRALLVNRVNVIFHV
AASVRFDDTLKYAAQMNLRGTMQVMELAKEVRDLSAVVHVSTSYSNTNRDPIEEVLYPP
LADWRETLAVCETADEHTMRVLTPKYLGELPNTYTFTKQLAEHVVAEYKGQLPIVIVRPSI
VISSVEEPSPGWIENFNGPVGIFVACGKGIMRTIHTKPDIICDFIPVDICAKNIIAGAWIRGTK

PIEPTDDIEIYNCCSGNLNNMLMGDLVEMSKHVSKEVPLDDMLWHTGGTITTNPVYHYIK
VLVQHLLPAFLVDTLLWLIGKKPMLVKVQRRIYIANLALSYYLTQQWTFDNTNFVQLRSV
LKQADMKQFYYDLETTDLVEYFTKCCLGGRRFLLKEKDEDIPKARAHCKRMMILDQAM
QVLFHAVFLWWVCKKIVQLFGIQSNLF
>*SfruFAR16*
MDSKFMSSDKMDEYQKHIEMMNGNDLNVLNLNYESHMNMEHPDKTSEAIADFYEDSIIL
VTGGTGFVGKALLEKLLRSCAGIKTVYVLMRPKRGLTVDQRYKELLKNQVFDRIRAKWP
ERLGKLYPITGDVSAPNLGVSPEQRELLGKVTAVFHSAATVRFTEPLHAATTLNVQGTASL
LKLAEDMPKLKALVHVSTAYSNAPRSHIEERVYPPPYDPDSIVRCTKMLPPETVEVISDTLQ
GEHPNPYTLTKALAESIVYGHTNLPVCIVRPSIVTAALQEPYPGWIDNVYGVTGIIMEISRG
TYRSGYCRERYVVDLVPVDMVVNSCILAAWRQGVKQPGRCPVYNVTSGSINPLQWGQFT
KLCVKWALENPTKYVMWYPNFAFTESRFMNTFWEITCHFLPAFMYDLLLRAQGRKAIM
MKLARRFKMAAATGEYFANHEWQFGIAELTALHDDASYARDSGAFPHWPADFDWDSYI
GAYMFGIRRYILKDTAESLPVARTKLRRLYWVHKLFQAATGYYIFRFLAGRLR
>*SfruFAR17*
MSKANCSNVASFYAGKSVFITGGTGFVGKTLIEKLLYSCNGIDKIYVLVRDKYGKQASERL
ARVTSTPVFDRLKTSKNEELKKIVVISGDITRENLGLQEDVLKTLENEVSVVFHLAATVAF
KLPLKDAMRINVNGTENVIALCRRMKKLQAFVHVSTAFTNSDRKEINEMVYPMPINLEEA
RHVADTYSHDDVIVSAFMGKKPNTYTFSKAFAEQQVMKQCEDLPTAIIRPSIVMSSIKEPC
PGWIDTWNGSTGLFVGMPAGILRVVLGRGSNVTDLVPVDIVTNLIVVAATECQKSKEIKVY
NCCTGTSNPITCDEATTISRKVALKYSLNELPWPFLMFTPSVFLYTLITFLLQIVPAYLIDLW
CILTGRKATQIKLQRRLKKVVDAVKFFLLNEWKFSDDNIRDLLKTMSPVDKEIFNFDVKTI
NWETCIEDYILGARKYLLKSEK
>*SfruFAR18*
MGFLEDRDLSGVPSIPEFYRGKTVFITGASGFMGKVLVEKLLYSCPDLDRIYILLRSKKGV
NPEDRLAKMLSVPLFDRLRKDRPGFASKVFVIAGDVMELGLGISEEDRALIVNRVNIVYH
VAASVRFDDTLEYAIRMNLRGTKEMVELAADIRNLDVFIHVSTSYSNTNRETLEEIIYPPHA
DWRETLEICETMDPHTLKVLTPKFLGELPNTYTFSKQLAENVVAEYKGILPVVIIRPSIVISS
VEEPVSGWVENFNGPVGLMLGCGKGILRSMYSDPDLIADYMPVDIAIKSFIVASWLRGTK
KLSPTDDVPIYNCCSGRLNIITMGDMIEKAKRISWTYPFNDILWVPGGGLTTSRTVYYYVKL
LIQHLLPAMLVDIILWCAGKKPMLFKIQRRIYIASMALIYYITKQWTFDNKNLVLMRSKIKE
EDMKDFYYVMENVDQDEFFVNATLGCRRYLLHEKDEDFPKAKRHYRRMVIVEIIAKIIFY
GALLWWFFNLNYTKKFISEWSH
>*SfruFAR19*
MGFLEDRDLSGVPSIPEFYRGKTVFITGASGFMGKVLVEKLLYSCPDLDRIYILLRNKKGV
KSEDRLAQILAAPLFDRLRKERPGFESQVFVIAGDVMELGLGISEEDRALIVNRVNIVYHV
AASVRFDDPLEYAIRMNLRGTKEMVELAADIRNLNVFVHVSTSYANTNRETLEEIVYPPH
ADWRETLNICETTDSHMLKVLTPKFLGELPNTYTFSKQLAENVVAEYKGILPVVIIRPSIVIS
SVEEPVPGWIENFNGPAALLAACGKGILRSMYTDPDLVADYMPVDISIKSFIVASWLRGTK
ELSPNDDLPIYNCCAGKLNTVTMGQMIAMGRQIYPSVPVNDMLWTPGGDLTKSKTLHYI
KVILLHLLPAIFVDTILWLAGRKPMLVKVQRRIYIANLALQYYITKQWTFDNTNLVLMRTK
IKDEDRKHFYYEMENIDQYKYFVNAVKGGKKYLLKEKDEDMPKAMKHYKRMVVVDIIV
QIVFYSALLWWFLHLNCIQNLISQIYHES
>*SfruFAR20*

MDAAEEVILKALDCQKGTKSVIDRGDSTVQQFYKDSTVFLTGASGFLGKQLVEKLFRTCK
LKKVYVLMRPKKGKSVEERIDEILLDPLYDILRKEKPDFTKRIIPIIGELAEKNLAISEADRRI
IIKEVDIIFHVAATISFQEPLRSAALVNVRGTKEMIVVAKQCRRLRSFVYMSTAYSHATYSR
GGTEIMEEFYTSPMCPELMIELAESLEEERLNRIKDELIKDWPNTYSFTKALAEELLRINSK
ELPVSVIRPAVVVCARREPYAGWIDLSSVFGPSGITLGCMMGVIHSLQSVQGIRIDFVPVDY
VNNTAIVAAAASKGATKIYHVATSKRNHLTWGGLTNILNNVARRTIVSPHAIWYCFVVQSP
YLWLHQLVSFLLHTIPANLADGACVLMKKPPRLKKIYTTVTKMASTIAFYSNHGWIFNDT
NALQLFQSLSKSDRVIYHCDVSDLEWTELIVLWCVGLRKYIVKDGLNTTQKGMKKQQIFR
ITTYVISFVFFAVIFLLFYCLFKLLCNFVIGF

>*SfruFAR21*

MGETEALDPAQELELHFMARQKPMHEATARGNSAIQQFYNGQNVFVTGGTGFLGKQLIE
KMFRATNVSKVFVLLRPKKGKAVELRINELLQDPVFDWVKEHKPKALNKIVPIAGDVAE
MRLGISDKDWKMLAEEINIIFHMAATTRFDETLRVATMINVRGAREALILGKACKQLKSY
VHVSTAYSYACDNLIDTEVLEDFYKSPIDPDTLIKMTETVDETRLNSITPSLIKNWPNTYSF
GKAVAEETVKNMCEGLPLCVVRPAIVICAMKEPSPGWLDMSNVYGASGIVLGPGIGLMHS
FMARDDVHIGLVPVDYVNNAILAAAWETAKRVAAGQTAPKIYSVTASTRNPTRWDNLVNI
NTSEVRTNYPTPAAIGWSFVWQTQYPLVFLIYSWFLHFIPGHVVDGICVLLGKERRFLKIY
KKMYNMCSALSYFTTNNWRFVDDNTAALYNSLSTIDKEIFDFDVCKINWREYMYIWCIG
LRKYIIKDGLKNTVYARRKQFVFKILTCMFMPLYVFALYKVFSFFVLTLFSFLGYFLHALGF

>*SfruFAR22*

MAEVDIMDPAQEYVETMLNRHKKMDDITERGDSEVQKFYEDAVVFVTGGTGFLGKQLV
EKLIRSTKLSKVYVVLRPKKGKGVEERLRALLKEPVFDKLRKLQPEFASKITPVAGDVSEP
NLGISEQEWNIMATEVNIIFHLAATTRFDASIRESTLINIRGAKEALRFGKQCRNLKSYVHV
STAFANACHSRINAQVEEDFYPPPMAPDMMIDLVESVEEERLNNMTPSLIKDWPNTYCFT
KAIAEEAVRAESGKLPICVVKPSIVIGAMKEPHPGWIDVSAVYGVTGAGLGKALGLMHTS
MANSKVFLGLIPVDYVTNATIVAAWETAKQKSTKDENPKVYTVISSTRSPKMWSDLYKL
MTDITLWKYPTPTTMAFSLYFQTRSPFLYWVYSWLFHMIPAYIADGVCSLLGRQRRFVKL
YTKMSKLMSALSYFSLNNWHFADNNTEALYDNLSAADKEIFSFDIDRLNWTEFISSWVIG
VRQFILKDGLVNTRYAIKKLKVLRALTYIGTAIYLFVLYKIVYFSYSMLSFAVGTVF

>*SfruFAR23*

MAVETMDPAQVFMAKITNRFKPVHDLTARGTSSVQQFYSGATVFLTGGSGFLGKQLIEKL
LRATRVSKIYVLLRSKKGKNVQQRLLEMLKDPVYETLREQQPDFVDKIVAVSGDVSELKL
GLSDKDWNTIADEVDIIFHMAATTRFDETLKRATFINVRGTREILDIGKACKKLRSFVYVS
TAYTHASADRVEDAVLEKFYPSPVSPKTMIGMAETIDEERLNDITTSLTKSWPNTYTFTKAI
GESLVESIGKDLPLCVVRPAIVVGTKREPTPGWLDVSCIYGATGLLLAVGLGVMHTSYARP
DNQIDLVPVDYVNNAILVAACETARTKRATSQHNETKIYTVTSSTRNRFTWDKLDYLLTNE
ARYYTTPKAVGYGFVWSTYNRLLFWLYTWLLHLIPAYIIDTIAVVFGHKRRYVKLARKMQ
RLSDVLAYFLTNGWKFEDQNTVTLFDNLSKDDKQIFNFDVTDIDWTEYILIWCLGLRKYV
IKDGLTDTLYACKKQTIFKFATYTLLPLYFYGLFKLMYCAVAFLYVTFLK

>*SfruFAR24*

METETLDPAQAFMAKVSDRFKPVHDLTARGTSSVQQFYSGATVFLTGGSGFLGKQLIEKL
FRATKISKIYVLLRCKKGKSMEQRLVDMLKDPLYDTLREQQPEFANKIVAVSGDVSELKLG
LSDKDWNTIADEVDIVFHLAATTRFDEELKLATFINIRGTREVLALGKACKKLRSFVYVST
AFSHARDEYIAGDVLEKFYESPISPENLIGVAESMDEERLNNITTSLIKGWPNTYTFTKAVA

EELVRTMGKDLPLCVVRPSIVTCSIQEPSPGWLDLSCIYGASGYVYAVGLGVAHTAYGEHS
NKLDFVPVDYVNNAILTAAWETTRTKSSAPGKDEIKIYTVSTATRKPITWRVVQDILANKA
RYYPTPKAVSYTFAWCTNIRLLYWIYAWLLHLIPAYIVDAVMVLFGKERRFGKLAQKLSK
MTMALSHFLSHHWKFDDRNTVALFESLSEDDRKIFNFDVTDIDWSEYILVWCLGLRKYFV
KDGLNGTIYGRKKQTVLKIVTFIMMPLYFYGLFKIVYCVFYYLFVLFNLLFK
>*SfruFAR25*
MAMETETLDPAQALMMKISDRFKPVQDLSARGTSSVQQFYSGATVFLTGGSGFLGKQLIE
KLFRATKISKMYILLRCKKGKSIEQRLQEMLQDSLYDTLREQQSDFAKKIVAVSGDTSELK
LGLSDKDWNTLADEVDLIFHLAATTRFDETLKMATFINIRGTREMLALGKACKKLRSFVY
VSTAFSHARYDRVNMEVLEQFYPSPISPETMIGIAETLDEEKINDITTSLTKKWPNTYTFTK
AIAEELVNTMGKDLPICVVRPAIVICTIQEPAPGWVDKSCIYGASGCLFATGVGVMHVTFA
KRDNLVALIPVDYVNNATIVAAWETAKTKRLAPENNEIKIYTLTSSNRNVIYWDAFEGIMC
GEVRNKYPTPKMVGYGSIKITHNRLLFWIYSWIMHLIPAYTLDAFAAGGKICLYYVRVDKF
GKLAQKLKKLTLALSYFLTHQWKFVDLNTAALHASLSEDDQKIFNFDVTDINWEEYMCT
WCIGLRKYLVKDELKGTLYARKKQTVFKYATFILFPIYLYEKDKGFGVELDVDIHCSPVISP
M
>*SfruFAR26*
MEVDTLDIAERHELALLEKQRPMNAVIDLGNSPVQQFYKGTTIFLTGGSGFLGKQFMEKIF
RSCEIERIYILLRPKKGKTIAQRLTYILKDPLYDTLRKKKPNFANDIVPIEGDVADVRLGLSD
KDWDTLTKEVNVIVHLAATVRFDEHLKKAGLINVRGTREMLELGKQCKNLKIFNHISTAF
AHTTKSRINNEVREQFYPTPVLPDTFIDLVENMEEERLTEITPGLIKGWPNTYTFTKAIAEEL
VRTRAGELPVCIVKPAVVLGSYTEPSPGWIDKQTVMASPVGFLLGCGIGVMHVLYLKRDN
QFVITPVDYANNAILAAGWDSVQNRKAIDSEVPIYIVGTATSNFTWDNLLNTLRTDELVKII
TPKALYYVCAAHTSNPVVFWILTWLFHYIPGYFIDAIVNILGVRPKGLPSLVSIYSKTYKLC
KVYEYFLFNNWKIRDDNLVSMMDRMAPADQAIFGCNVREIDQRGIALSMSVGLRRFIVQ
DGLKDSEYGQKKMRILKYVHVVCVLLYSYLIYSVFRTIYSAFCVIIPV
>*SfruFAR27*
MDLAQEVENKALASYEPVEKLMRQGTSDVQRFYANETVFLTGGSGFLGKQFIEKIFRSCA
IKKMYVLIRPKKGKTIQDRIQFILSDPVFDKLRTVKPNFAEQIVLLEGDVADIRLGISDKEW
EMVAKDTSIIVHMAATIRFDSPIRDAVVTNVRGTREVISLAKDCKHIKSFVHVSTGFTHATA
SRIGKDLREEFHPCPISPSAIIGMVETMEDSRLDNITAELIKDWPNTYTFTKAIAEELVRTTA
ADLPVAIVKPPLVLTSYIEPSPGWADTSIMTGPCGVLIGVGLGLLRVFYLKSDLNLNIAPVD
MVNNAIIAAGWDSVANRPASDGIPIYTVSSTKCGIPWNYFGSLVRSEEFKHLACPKAVWY
CYLIETTNNLLYLFLFWFLHYIPAYVIDGICYVMKIKIKGIPSATKVYEKIYKVSKVFGYFM
TNEWHFKDDNLVAMINRMSETDKTIYNCDVASINYKEFLIRMIVGLRKFIVKDGLIDSEKA
YRKQKRLFYANLVFMTLYIYVWVMIAFYMFKGIKYIVY
>*SfruFAR28*
MAAVSQLISVSDVISENLNKNENNTYCAQCNDSVDENMNNMSDNLTEVQKFYNGKNILI
TGATGFLGKILVEKLLRCCPGVENLYLLVRQKRGKDIYTRMEEIFDDPVFSRLKEEVPKFR
HKVVVVPADCEAAGLGLTLTDRQMLTEKVNIIFHSAATVKFDEHLRAALVTNVMAPLHLL
RLARDLKKLDVLMHISTAYSNSHLPLIEERFYPCEADCEQLHQMVDKLTDDQLNSMLPKI
LGPWPNTYTFTKALAEKELRASAGGMPIGIFRPAIVISTAQEPLKGWLDNMYGPTGVAVGS
ATGILRTMQCDELVSADIVPVDSVVNCLMVAACSVHNAYKRSSPPLEPPIFNYVSSVENRIT
WGDFLSKNMAWIHHYPFSDAVWFISVRLTKSAAVNKVYMFFLHLVPATLVDGLAVCLGR

KPKMLKVYRKIHKFSSVLSYFCTREIKFCNSRTRELWEKTSEADKKLYPFSMSELNWEEYF
RDYLAGIRRYLFKESDDTLPQARIKWKRLYYLHQITRFIFFILAVYALWWILSLMW

>*SfruFAR29*

MGRRLAAISPDVAMVPRPVSPSPAEPLVPRFYAGRSILITGATGFMGKVLVERILATCPDVG
RLHLLMRDKKGHSPQKRLAQLKQSQVFDKVRARNHRQLDKLCVVSGDVSKPQLGMDA
DAIAQLREVSVVFHSAATLKFDEPLPVALDQNVRSVQRLLDICDQLPKMQAFIHVSTAYSN
AELAVVEERVYPAPVPLAQACTLAQTLPGDLLGQINTQYISPKPNTYTFTKALAETVVQEH
GNRGYPVAIFRPSIVISSLRHPFPGWIENLNGPSGVVVAAGKGLLHVFCCRSAARADMLPV
DMAIDTLLCVAWETAIDRPESVRVYNCSTCENPTTWREFEAALRRHLRGHPLDNAFWYPC
GFTVENKLTQKTLEAVLQTAPLHVAEYISKILGIKTKLSLITVSQRLVAMNDVLRFFSVREW
HFVTDNVRKLHARLTPQDAAIYNLDPQTINWDEHYCNFVKGARKYLLQEKDQDIDEAKK
HLRRMYYLHQGVLFFVVTLLCRLALQNQYLRAFIYRTFRMLLTVAGSAYMRIRQS

>*OnubFAR6*

MVSCKDDVTAQIPQFYAGRSIFITGATGFMGKVLVERLLTTCPDVDQLFLLMRTKRDVGPE
KRLQQLKESQVFDNVRQCSPAQLDKLCIVPGDITKPQLGMTGESIAQLRNVSVVFHSAAT
LKFDEALGLAVDQNVRSVVRLMELCDMLPNMQAFVYVSTGYSNAELSVVEERVYPPPAP
LEQVLALTEAMPEDLMASVTRKYIHPKPNTYTFTKAIAEVAVQQHNSSYPRAIFRPTIVVS
ALRTPFPGWVENLNGPTGVIAGVGKGLLHFFSCRRDARADMVPVDIVIDALLAVGWETAI
DRSSEVRVYNCSSYNNPTTWRTFEAALSRYIREHPMDQVFWYPYGSIVENGVAQKCLELL
LQTMPLHLADYCSRIFGIKTKLSFIMVSNRLQAMNTVLQFFSQREWKFITNNMERLRQRL
TPADAAIFNLDVKTINWEELCTNFIKGTKKYIFKEKAENTPEAKKHLQRMKLLPPWCNFL
HFNPASTTHL

>*OnubFAR4*

MAPSMTVPEYYAGKTLFITGSTGFMGKVLIEKLLRSCPDIKKIYLLMRPKKGHGSKERLD
GFMNCRVFDKLKSEHPEQFHKLQVVPGDILMEDLGLSVEDRDTLQRECQVLMHCAACV
RFDMFIRDAVNMNTVGTKRVLDVASGMKQIEVFVHVSTAYCRCEVEVLEERLYPAKHRPK
HVIETVNWMDDELLTHLQPKIIEPQPNTYAYTKSLTEDLVAQHAGKFPIVIARPSIVTAAYK
EPMPGWVDNLNGPTGLLVGAGKGVIRTMHCNENYAADIVPVDMVVNACILLGYTTALEK
PKEVQFCNIAQSGINGFTWGEALALGRIHVMEYPFSICLWYPGGAPSASRLRHQVTLFLTH
TLPAYTVDLLARALQQKPFMVKMQKRIQSGLEVLQYYTTKEWHFRNENLRALRTKVTEE
DDETFYTDLTVIDWNTYIRDYIKGAREFVMKEDPSTLPQARRLNRQLYYLDTFTKIFIYSII
ASSWMKRSISNILKMFWAA

>*OnubFAR2*

MNHSLEASPLIPTEVTRDKMERWVEAQNKGEKTEVDVYGKPSEKQIKELENIRSLSRELQ
DNLHELENSVRIADVENQAMNPTAKILDYSEDHEFVSANQLDNYYAEEDKVDAKEEEKR
RLTKGKAGKTDIQQFYKDQCVFLTGGTGFLGKVLIEKLLRSCTDVDTIYVLIRNKKGKDA
RVRLHDMLDEFLFHRALDENPKGIHKVVPIIGDMELPGLGISDEDRRTITSKVSIIINAAATV
KFDEKLSVSTGINVRGTKEVLRLAKECRNLRAITHVSTAFSNTHVPYIEEKFYDPPMSVEA
LEAVSEINEDLIDAILPTLIGKRPNTYCFTKAVAEEAVRKYGEGLPICIVRPSIVVSTYEEPVR
GWTDSVYGPTGLVVGTGTGVLRTMYMDLNKVADMVPVDLTVNAILASAWHAAKNFKE
NQTSDIPIYNFVSGAQNPIKWGKFIELNRKYGIDKPTTKAVWYYGLNPTNSYFMFLFYNFF
LHYLPALMVDAYCAVTGRRRAMLKLYSKVMKMANILFYFSTRDWRFSDRGVRAMWASL
SEADRAVFPFSMGDMSWDRLCETFLIGLRVYLIKDDLSTLPEARKKWNRLFYLHQLLKVV
TFALVLNLAYLVLRPILSLVFGL

>*OnubFAR5*
MAPLLDSTPGDDAAFKIPAMAYSNDNDNDASLKTDNNNDIDTRNNVELTQIQKFYNGKNI
LITGATGFLGKILVQKLLRCCPGVENLYLLVRQKRGKDIYTRIEEIFDDPVFDRLKEEVPKF
RHKIVVIPADCEAAGLGLTLSDRQTLTEKVNVIFHSAATVKFDEQLRLAFNVNVKASLHVL
RLARDIKGLDVLMHISTAYSNSHLDQVEEKFYPCNMDLETLNQTIEKMTDEEINSQLSKLL
GEYPNTYTMTKALAENELRINSKGIPLAIFRPAVVVSSAKEPVKCWLDNMNGPNGIVVGS
GTGILRTLQCDMQGTADPVPVDMVVNCLMAAAVNVNAAYAQSNPPPEPPIFNYVSSVEN
KITWGEFMTLNMEQVDKYPFSNAVWYISLRLTKSWLLNRFYMFFLHLIPAVLMDGLTVCV
GKKPKMLKVYKKIHKFSDVLSYFCTKDIEFCNRRTQELWQKTSEEDKQLFPFSMAELDW
RDYFQDYYLGIRRYLFKENDDNLPQARIKWKRLYYLHQLVTMLFYFLALYALWSTFSSFL
L
>*OnubFAR1*
MGFLEERNFEDVPEIPEYYKGKTIFITGGSGFMGKALIEKLLYSCSDLDRIYLLLRTKKGVK
AEDRLAELYSTGCFDRLREEKPDIFSSKVFVVAGDVMDPGLGLSEEDRALLVNRVNIIFHV
AASVRFDDPLPFATKLNLGGTKEVVTLAKEVRNLSALVHVSTVYSNTNRDVIEEVMYPPH
ADWKDTLEVCDVIDEHSLRVLTPKYLGDIPNTYTFTKQLAEHVVYEQKGQLPAVIIGPSIVI
SSVEEPVPGWIENFNGPVGMLVACGKGIMRSIYTDPNLISDYMPVDVSIKSIVTAAWIRGT
KKLEPPDDVQIYNCCAGKLNNITMQELVDIGKRLAASLPLNDTLWNIGGSITTSRTIHYIKV
LILHCLPAIFVDAILLLLGKKPLLLKVQRRIYIANLALRYYITKEWTFDNTNFIQLRSRIKEQ
DKKHFYYDIENVDKEEYFRKSCIGGRKYLLKEKDEDLPKAKAHHKRMVILDKTVQCIFY
GYLFWWVVNTHYIKGLTDFLYEIFTM
>*OnubEpgFAR*
MSANTMETDEQFTYNSPIVNFYSGKSVFVTGATGFLGTVLVEKLLFSCKGINNIYILIKQTE
DLTIEARILNYLNSKAFHRVKNTNPELMKKIIPICGNLEDKNLGISDSDMKTLLEEVSIVFH
VAAKLLFKMSLTAAVNINTKPTEQLIAICKKMRRNPIFIYVSSAYSNVNEQIIDEKVYNTGV
PLETIYDTLDTENTRITDIFLDKRPNTYTYSKALAEVVVEKEFDESAAIVRPSIIVSSIREPIP
GWLSGSHGFPRVVGAACKGLLLRWHGDGTVVCDLIPVDHVANLIIAAAWESNERRLMGN
KGVKVYNCCSSLRNPIDVITVVKTCIKYRKYFGTRTMSIFTPRFIMKKNYFIYKLLYFTYHT
IPAAIIDGFFWLTGRTPIMLKTLDKLGKISSVLEYFTHHQFIFLDSNVRGLLRRMEGTDRQT
FNFDVTEIEWEPYLQNFVRGIANNYDYSM
>*OnubZpgFAR*
MSANTMETDEQFTYNSPIVNFYSGKSVFVTGATGFLGTVLVEKLLFSCKGINNIYILIKQTE
DLTIEARILNYLNSKAFHRVKNTNPELMKKIIPICGNLEDKNLGISDSDMKTLLEEVSIVFHL
AAKLLFKMSLAAAVNINTKSTEQLIAICKKMRRNPIFIYVSSAYSNVNKQIIDEKVYSTGVP
LETIYDTLDAKNTRLMDIFLDKRPNTYTYSKALAEVLVENEFDESAAIVRPSIIASSIREPIP
GWLSGSHGFPRVVEAACKGLLLRWHGDGTVAFGIIPVDHVANLIIAAAWESNERRLIGNK
GVKVYNCCSGLRNPIDVSTVMNTCLKYRKYFGTRTMSIITPRFIMKKNYFLYKLLYFTYHT
IPAAIIDGFFWLTGRTPMMLNTLHKLRKLSSVLEYFTLRQFLFLDSNVRGLLRRMEGTDRQ
TFNFDVTEIEWEPFLQNCVRGIANNYD
>*SexiFAR1*
MAVETLTEHQLFEAKQGGDITYKDMVEESQPLGDSQIQKLFAGSSVLLTGGTGFLGKLIVE
KLLRSCPDLKKIFLLARPKKNKTITKRLQEQFDDVLYDRLRKECPDFINKISLVEGDVGQL
DLGMCPEDRIKIMNEVEIIFHGAATVRFDEPLKTAVEINVRGTREMFKLARGCSKLKAFVH
ISTAYSNCPQNMIGEEFYESPLPGDKLIDLVETMEEKVINNITPGLLGDFPNTYAYTKAVAE

NIVKEYSKGLPVALFRPSIVIGTSKEPVSGWIDNVYGPTGVVVGAAVGLLHVLNCNPKVIA
DLVPGDMVVNACIATAWKTAKEYPSNHEDAPPPDLTPPVYNYVSSEQRPLTWEKFMNYN
EVYGFQVPTVQAIYYYLFHLTSSKFLYNLYCFLLHWIPAYIIDGIAVIIGKKPMLRKAYKKIT
KFSEVMAYFATREWKFDNTNTQQLFKELCDADKYMFDFDMSSLEWNEYFYNYIRGVRV
YLLKDPVETVPQGLKKHNRLKYLHYTFCAILGLLFLRLLWAIFSGIFSF
>*SexiFAR2*
MTYRQINEFDAEKFTAAAVPTSYVSVPDFYAGKSIFITGGTGFLGKVFLEKLLYSCKDVET
VYILIREKKGKTPQQRVEDLFNKPIFSRLKQKDSQCMKKVTAIIGDLSEPGLGISKDDEELL
LQKVSVVFHVAANVQFYKEFKEIINTNVGGTKYVLQLCQRIKDIKAFVHISTAYCHTDQK
VLEERIYPPPAELSEVLKFLQQPQHDKKQIKELFKKQPNSYTFAKALAETYIAENCGRVPTII
IRPSIISASLKEPLPGWVDSWNGATGLITASYNGANRVLLGEGGNFLDLIPVDFVANLAIVA
AAKCTSSLKVYNCCSSGCNPLTLKQLVSHMNNVGFDKNVSIIFTNNKASLSTLTFFLQTTP
SFTADMFLRVTGKSPRYMKIQSKLTIARNALNFFTCHSWVMKADNSRRLYASLSLHDRHT
FPCDPTDIDWKKYITIYIEGINQFLMKKRS
>*SexiFAR3*
MVVLTSKEKSNMSVADFYAGKSVFITGGTGFLGKVFIEKLLYSCPDIDKIYMLIREKKGQSI
RERLTKIVDDPLFNRLKEKRPGDLDKIVLIPGDVTVPGLGISDENEAILIDKVSVVIHSAATV
KFNEPLATAWNVNVEGTRMIMALSRRMKRIEIFIHISTAYTNTNRAVVDEVLYPPPADINEV
HQYVKNGITEEETEKILNGRPNTYTFTKALTEHLVAENQAYMPTVIVRPSIVGAIKDDPIRG
WLANWYGATGLSVFTAKGLNRVIYGQSSHVVDLIPVDYVANLVIVAGAKTYRSNEVTIYN
SCSSSCNPITMERLVGLFIDDTVKHNSYVMPLPGWYVYSNYRWLVYLVTIIFQMIPAYLADI
GRRLLGKNPRYYKLQSLVAQTQEAVHFFTSHTWEIKSKRTSELFASLSHTDQRMFPCDAK
KIDWTDYITDYCSGVRQFLEKRK
>*SexiFAR4*
MCHTYCFYYIKKLIMAPSMNIAEYYAGKTLFITGATGFMGKVMVEKLLRDCSDVKKMYL
LMRPKKGHSSKERLDDILSFRIFDRLKAENPKLFEKLQVVAGDILSEDLGLSPEDRLLIQEE
AQIIFHCAACVRFDMFLRDAVKMNTMGTKKVLELAEGVKNLQAFVHVSTSYCRCELPIFE
EKLYPSKHRPEHVMHCVNWMDDELLGHLQPKIIEPQPNTYAYTKSLTEDLVSQYEGKFPIV
IARPSIVAAAYKEPLPGWVDNL
>*SexiFAR5*
MPTPLAVWYTFIINTSNKPLFFLLTWLLHYIPGYILDAGCILLGKPTMFIKLYNRVNRSSLAL
SYFTSRTWVFNDNNSDKLFQSLSKSDKLIFNFDTTDINIPEFVTIWCVGLRKYLMKDGIKN
TEYARKKQ
>*SexiFAR6*
MVPRPAPQFPTPPLIPEYFAGREVLITGATGFMGKVLVERLLWTCPDIGRLHLLMRHKRDV
APDKRLALLKQSQVFDVVRERCPQQLDKLCMVPGDVTKRRFGFDQSALNQLNQVSVVF
HSAATLKFDEPLSVAAEQNVRPVLTLMDICDQLPNMQVLVHVSTAYSNAELAEVEERVYP
APVTPEHLLALVDALPASMLQEITPRLIAPKPNTYTFTKAVAESAVSERAVTARYACAIFRPT
IVVSSLRHPFPGWIENLNGPSGVVAGAGKGLLRVLRCGAQRRADMMPVDICIDTLIAVAW
ETGIDNLREARVYQCASSSHAATWGQFRERMLRLVREHPFDNVLWYPYGVICENTVVQK
VLEAVLQTAPLCVAHCVARACGLKQKPSLWTACKRLQAMNQALQFFATRHWSFRTTRVQ
QLAHRLHPDDQKLYNLRPETIDWEQHCVDFVKGARRYLLRERDDDIHAARRRMRILYLIH
KATLLFAIFTMCRLTIRTAPAILWGVAALTRQRNKSALLS
>*SexiFAR7*

MANPTIRDFYRGRNILVTGGTGFMGKVLIEKVLYSIPDVGNIYILMRPKRGKSVAQRIEDM
QRLRLFERIRSEKPDALKKMKPLQGDVLFENLGLSDSDIEKLSNEVSVVFHFAATLKLEAP
LKDNVNMNTCGTQRALEIAKKFKKLDIFVHLSTAFCYPDYEVLGERCYGPPVRPENVMK
LIQWLDDKQLALLTPSLLGPHPNCYTFSKRLAETIVEQAHDELPVVIARPSIVCPSVAEPMP
GWVDNLNGPVGVMLGAGKGVIRTMLCDGSLIAQVCPVDIAINAIIAIGMIEGNRKEKHTT
LPVYNVNNGHQKPTTWGDVLTIAKDYGRKYPLSWPLWYPNGDITTNAVLHEYRRIFYHL
VPAYLIDFLLFLLGQKRIMIRIQERISQGLEVLQYFTMRPWNFPCPNYDAIKEKLSPEEQVIF
NTDITDADRDEYMKMCVEGGRVYCFKEDPTKIPYNRIYHRFLYVLDWFVKIMFWLFVLS
YLASWFEPIKTLFSLGEPVVKHLPFLGKVVCKTEEL
>*SexiFAR8*
MASETISSAELECLPDRIADTFSGMKVLITGGTGFMGKVLVEKLLRKCPDIEQIYLFVRAK
KGKNPKQRLEEIFSGPLFEKLRDMRGGVEPLLAKMTIVNGDVSEPDLGMSPEDRQMIINQ
VDIIIHAAATIRFDEELKKAVLLNVRGTKLMVELGKACKNLKVFIHISTAYCHLHEKLLEEK
PYPPPADPHQIIQAVEWMDEETIANMTPKLLNKLPNSYAFTKALAEALVVEAMEKANLPA
MVLRPSIVIPIWQEPVPGWTDNINGPTGLLIGAGKGVIRSMYCKSNSYADYLPVDVFINGI
MIVAWNYLKNGDKKCNIINFTSSAEIKVTWSEMIDAGREIIMNRVPLNGVVWYPGGSMKH
SRMYHNICVFFFHWIPAFIIDTLLFCLGYKPVLCRVQRRITKGFEVFEYYTNNQWDFKSDIA
QTLRQKLNAKERRDYKVDAVGLDISKYFEDCIRAARIFILKEYDDTLPAARRHMRIMYWV
DVIVNCLFWGFLLYWLSGWMTTSKAIVPDTATPTVIAMDA
>*SexiFAR9*
MNTNFESTKKLLELSQRMKNIEVFLYISTAFTHTQKKVLVETVYPPPAKVEDIYKFIEENGD
DEQATLKFINGQPNTYTFTKSLSEAYLSKHHGNVPAVIIRPSIVSATNQEPIPGWLDNWYGS
TPFLMNASEGWLRIVRGNYNSGIDFIPVDFVTNLSIVAAAKAKRTNEVQVFHSTTSADNPT
DWSDFKNYFLDEVVRRGKNDLPYPNIVFVESKIALAIGSFLMQTVPAHMLDLWLKITGKE
PRYVKILAQVIRLRDSYEHFSSNDWIMRSDRTRQLHASLSPEDQEKFQCDPTQINWPEYLK
DYCRGVLKYLKPRKMY
>*SexiFAR10*
MNYTAEKSPLSQTPVTREKMDKWVDAQVKGEKLDIDVYGKPTDKMLKELENVRNLSKE
LQDNLHELENSVRIAEVENQAMNPTAQILDFSEDHEFVPDNKDTYYAEEDKLDAKEEEKK
KLTKGKGPKTEIQEFYKDQCVFLTGGTGFLGKVLIEKLIRSCGDINTIYVLARNKKGKDPR
VRLHEMMDEFLFHRAHEENPKGIHKVVPILGDMELPGLGINEEDRKMLASKVTIIINAAAT
VKFDEKLSVSTAINVKGTKEVLKLAKECRNLKAITHVSTAFSNTQVKHIEEKFYEPPMSVE
ALEAISEVDEKLVESILPTLLGSRPNTYCFTKAVAEEAVRTYGEGLPICIVRPSIVVSTYEEPV
RGWTDSVYGPTGLVVGIGTGVLRTMYMDQEKVADMVPVDLCVNAILASAWHTAKNYK
ENQTSHIPIYNFVSGAQNPLTWGEFIERNRKYGIDKPTTKAVWYYGLNPTNNYYLFLFYNF
FLHYLPALMIDTYCAITGKRRAMLKLYSKVMKLANILFYFSTQDWKFSDMNVRNMWNS
LSDADRVIFPFSIGEMSWEYMCETFLVGLRVYLIKDDLSTLPEARKKWNKLFYLHQTLKAI
TLSLVVYLTYFVLQPIIALVFN
>*SexiFAR11*
MASEVNEWYKGRSVLVTGALGLMGKVLIEKLLYSVPDVGCVYALVRSKRGKSPETRIEE
MWKLPLFKRIREEKPHVMKKLIPVTGDIMYDELGISAENLKNIYNEVSIVFHFAASLRLEA
PLKEGLEMNTRGTLRVLEMAKKMKSLVAFIHLSTAFCYPDYERMAEKVFDPPTDPHEVLR
AASWLTEEQLNLLAPSIYQKHPNSYTYSKRLAEALVRESYPNLPAVVVRPSIVTPSYKEPTP
GWVDNLNGPIGLMVGAGKGVIRSMHCYGHYHAEVIPVDIAINSIIVIAYKTGKDTERQPEI

PVYNLTTGDDRNTTWKEVLDIGKATVRKFPFEGPLWYPDGNIRHNKFIHDLCVFFYHIIPA
YFIDFLMFLFRQKRFMVRIQNRISIGLEVLQYFTTREWWFDTNNYKSLVHLLNPVDKETFP
MDTTIIEDEPYIESCMIGGKLYCLKEKLENLPKARLQNHILYILDRLVSLFFYLVLLYWIVSY
FEPARELLSYGGPAVRYLPLVGKAVFKDV

>*SexiFAR12*

MADPSQVRSFYAGKNFFITGGTGFVGLCLIEKILRCIPDSGKIYLLMRPKKGKEIADRLQEF
PKNPVFEKLLESNSTDVFNKLIPIAGDVGEENLGMSPQDRQTIINNVNVVIHSAATLDFQE
NLRPTVNINLLGTRRVMQLCKDAKNLKVMIHVSSAYVNSYLTEAHEKIYEAPEDPEKVISL
VGTLNDEALLEIEPKLLKSHPNTYTFTKHLAEHEVVKCADLFPCTIVRPTMIVASWKEPIPG
WTCSKVGPQGFLMGAAKGVVRRLPLAKENVADYIPVDVVVNQLLVAGWEAANSSSGLT
VYHCSSSTCHPFTWTMLDDTVNGMLHKYPLKSAVWYPHLKFVPSLLMFRISAIFVHFFPA
LLLDLMLRMTGGRPILIRLHKNVWNSLNRLERFIFSEWKFHNPNTLELATKLNQTDKELFF
IDISKLYWVEYFKTLHLGVRRYLNKEKESTLPAARKKDMVLLLFHVMWQLFIMGLVWYI
FACATGLTLAHSAWVAPIIYILFTFL

>*SexiFAR13*

MAPNVKEYYRGKNIFITGGTGFVGKALLEKLLRNCSEVNAIYLLMRQKKGVSAEERLKD
LCSKPVFDMVREKNPSSFKKLMIINGDITEAGLGISEDDVKLLQKECNIIFHSAACVRFDQ
KLKDAVNMNTTGTLRMLTLAESMQNLEVFVHLSTAYCRCDLDVLEEKVYTAVHKPRKIM
DIVEWMDDETLAYLEPKIISSEPNTYSYTKAITEDLVNEYSGKFPIAIARPSIVTAVWKEPIP
GWVDNLNGPTGIVIGSGKGVIRTMHCEPSYKADAISVDVVANACILIAYVTGLDKPKETQ
VYNLTLSGVINLTWQEIIDLGEKWVNEFPYTMALWYPGGSIKSYRITHQIDKFFSHLVPAYL
VDALLFLLGKKTFMINLQKRISHGLNVLQYYTTKEWHFRNNNYKGLRNRVTPEDNEVFY
TDASTLDPDEYLKNYVLGTRKFCCNEDPANLPRARKLHRIRYMADRFFKLLFIFLILWTLY
SNSHVFTSSVELLDNSLKSLPLMNQANAEEISNIAL

>*SlitFAR1*

MAAETLTENQLFEAKQGGDITYMDMIEETQPLGDSQIQKLFAGSAVLLTGGTGFLGKLVV
EKLLRSCPDLKKIFLLARPKKNKTITKRLQEQFDDVLYDRLRKECPDFINKISIVEGDVGQL
DLGMCPEDRIKIMNEVEVIFHGAATVRFDEPLKTAVEINVRGTREMLKLARGCSKLKAFV
HISTAYSNCPQNMIGEKFYESPLPGDKLIDLVETMEEKVINNITPGLLGDFPNTYAYTKAVA
ENIVKEYSKGLPVALFRPSIVIGTSKEPVSGWIDNVYGPTGVVVGAAVGLLHVLNCNPKV
KADLVPGDMVVNACIATAWKTAKEYPSNHEDAPPPDLTPPVYNYVSSEQRPLTWEKFMN
YNEVYGFQVPTVQAIYYYLFHLTSSKFLYNLYCFLLHWIPAYIIDGIAVIIGKKPILRKAYKKI
TKFSEVMAYFATREWKFDNSNTQQLFKELCDADKYLFDFDMSALQWNEYFYNYIRGVRV
YLLKDPVDTVPEGLKKHHRLKFLHYTFCGILGLLFFRLLWAMISGILSF

>*SlitFAR2*

MLWVDFSVSLNLDFKIFATMVPRPVSPSPAEPLIPRFYAGRSILITGATGFMGKVLVERILST
CPEVGRLHLLMRDKKGHSPQKRLAQLKQSQVFDNVRARNHRQLDKLCVVSGDVSKPQL
GMDADAIAQLREVSIVFHSAATLKFDEPLPVAIDQNVRSVERLLDICDKLPNMEAFIHVST
AYSNAELTVVEERVYPAPVPLAQACTLAETLPVDLLGQINTQYISPKPNTYTF

>*SlitFAR3*

MVVLTSKEKSNMSVADFYAGKSVFITGGTGFLGKVFIEKLLYSCPDIDKIYMLIREKKGQSI
RERLTKIVDDPLFNRLKDKRPGDLGKIILIPGDITVPGLGISEENETILIEKVSVVIHSAATVK
FNEPLATAWNVNVEGTRMIMALSRRMKRIEVFIHISTAYTNTNRAVIDEVLYPPPADINDVH
QHVKNGVTEEETEKILNGRPNTYTFTKALTEHLVAENQSYMPTIIVRPSIVGAIKDDPIRGW

LANWYGATGLSVFTAKGLNRVIYGHSNHVVDLIPVDYVANLVIVAGAKTYHSNEVTIYNS
CSSSCNPITMKRLVGLFIDYTVKHKSYVMPLPGWYVYSNYRWLVFLVTLIFQVIPAYLGDI
GRRLLGKNPRYYKLQNLVAQTQEAVHFFTSHTWEIKSKRTSELFSSLSLTDQRMFPCDANR
IDWTDYITDYCSGVRQFLEKIK
>*SlitFAR4*
MAPSVNIAEYYAGKTLFITGATGFMGKVMVEKLLRDCSDVKKMYLLMRPKKGHSSKER
LDELLNFKIFDRLKAENPKLFEKLQVVAGDILLEDLGLSAEDRLLIQEEAQIIFHCAACVRF
DMFLRDAVKMNTMGTKKVLELAEGVKNLEAFVHVSTSYCRCELPLFEEKLYPSKHRPEH
VMHCVNWMDDELLGHLQPKIIEPQPNTYAYTKSLTEDLVSQYEGKFPIVIARPSIVAAAYK
EPLPGWVDNLN
>*SlitFAR5*
MDPALAVELEALSRQKAMFEATERGDSTVQQFYKDSTVFLTGASGFLGKQLVEKLFRACN
IRKIFILLRPKKNMTIQERLEEMLQDPVFNLVKKKKPDFAENIVPVKGDVAETKLGLSDTD
WTMITSEVDVIFHVAATTRFDEALRVSTMINIRGTRETVLLGKDCQKLKSFVYVSTTYSTA
TQANVDKEVMERFYPCPLPPELMIDMAENIDDERMEAIEANLIKGYPNTYTFTKSIAEEVV
RSLAGEMPTCIIRPAVVISSYREPVPGWADASCAFGASGLILGPATGLIHAIYASNDVKFSLV
PVDYVNNAILVAGWHTATEKPNDVQIYSVSSARNLFHWEPISSKIRDIGKVLPTPLAVWYT
FIINTSNKPLFFILTWLLHYIPGYILDAGCILLGKPTMFIKLYNRVNRSSLALSYFTTHTWVF
NDSNTDKLFNSLSKTDRLIFNFDTSDINISEFVTLWCVGLRKYLMKDGIKNTEYARKKQFL
LKYLHYVVSFMYVYVLFKITCLVCYLILCLFG
>*SlitFAR6*
MVPRPAPQSSTPPLIPEYFAGREVFITGATGFMGKVLVERLLWTCPDISRLHLLMRHKKDC
APDKRLALLKQSQVFDVVREQCPQQLDKLCVVPGDVTKRRFGFDQPTLNQLNQVSVVFH
SAATLKFDEPLSVAVEQNVRPVLTLMDICDQLPNMQVFIHVSTAYSNAELSTVEERVYPAP
VSPSHLLALVDALPATMLQEITPRLIAPKPNTYTFTKAVAESAVSERATSAHYACAIFRPSIV
VSSLRHPFPGWIENLNGPSGVIAGAGKGLLRVLRCGAQRRADMMPVDICIDTLIAVAWET
GVDNLREGRVYTCASSCHAATWGQFRARMLRLVREHPFDNVLWYPYGIICENTVIQKVL
ETVLQTAPLCLAHCVWRACGLKQKPSLWTACKRLQAMNHALQFFATRHWSFSTTHVQK
LADRLHGEDKLRYNLRPETIDWEQHCVDFVKGARRYLLRERDDDIHTARRRMKLLTVIH
NATLLFAIFFVCRLTIRTAPAILRGVAVLTRLRNKAIQES
>*SlitFAR7*
MSNPSIRDFYKGRNILVTGGTGFMGKVLIEKMLYSIPDLGNIYILMRPKRGKSVAQRIEDM
QRLRLFERIRTEKPDAFKKMKPLQGDVLFDNLGLSDSDIELLCNEVSVVFHFAATLRLEAP
LKDNVNMNTCGTQRALDIAKKFKKLDIFVHLSTAFCYPDYEVLGEKCFGPPVKPENVMK
LIQWLDDKQLALLTPSLLGPHPNCYTFSKRLAETIVEQAHDELPVVIARPSIVCPSLKEPVP
GWVDNLNGPVGVMLGAGKGVIRTMLCDGSLTAQVCPVDIAINGIIAIGMIEGNKKEKHTS
LPVYNVNNGHQKPTTWGDVLTIAKDYGRKYPLSWPLWYPNGDITTNAVLHEYRRIFYHL
VPAYLIDFLLFLLGQKRIMIRIQERISQGLEVLQYFTMRPWNFPCPNYDAIREKLSPEEQEIY
NTDTTDVDRHEYMKMCVEGGRVYCFKEDPNKIPYNRIYHRFLYVLDWFVKIMFWLFVLS
FIASWCGPIKTVFSFGEPIVKHLPFLGKVVSKVEEL
>*SlitFAR8*
MASETISSAELECLPDRIADTFSGMKVLITGGTGFMGKVLVEKLLRKCPDIDQILLFVRSKK
GKNPKQRLEEICSGVLFEKLREMRGGVEPLLEKVTLINGDVSEPDLAMSPEDRQMIIDQV
DIIIHAAATIRFDEELKKAVLLNVRGTKLMVELGKACKNLKVFIHISTAYCHLHEKLLEEKA

YSPPADPHQIIQAVEWMDEETIATMTPKLLNKLPNSYAFTKALAEALVVEAMEKANLPAM
VLRPSIVIPIWQEPVPGWTDNINGPTGLLIGAGKGVIRSMYCKSNSYADYLPVDVFINGIMI
VAWNYLKNGDKKCNIINFTSSAEIKVTWSEMIDAGREIIMNRVPLNGVVWYPGGSMKHSR
LYHNICVFFFHWIPAFIIDTLLFCLGYKPVLCRVQRRITKGFEVFEYYTNNQWDFKSDIAQT
LRQKLNAKERRDYKVDAVGLDISKYFEDCIRAARVFILKEYDDTLPAARRHMRIMYWVD
VIVNCLFWGFLLYWLSGWMTTSKAIVPDTASPTVIAMDA
>*SlitFAR9*
MHCNENYEADVVPVDVTVNACIILGYLTGMEKPKKINFCNITQSQINPITWGQALDMGRV
HVQEFPFTVCLWYPGGSAKSSWIAHQFALFFTHMLPAYFVDLLMFLMGKKTFMIKIQKRI
NYGLEVLQYYTTKEWHFTNDFFVSLQNRISKRDNEIFYTNMKEMDWSQYIRNYIRGARE
YCCKEDPSTLPAARRLQKQLYYLDKAVQIMVGLLVSYFIYYYFNMLYSMISS
>*SlitFAR10*
MNYAIEKSPLSQTPVTREKMEKWVDAQVKGEKADIDVYGKPTDKMLKELENVRNLSKE
LQDNLHELENSVRIAEVENQAMNPTAQILDFSEDHEFVPDNKDTYYAEEDKVDAKEEEK
KKLTKGKGPKTEIQEFYKDQCVFLTGGTGFLGKVLIEKIIRSCGDINTIYVLARNKKGKDPR
VRLHEMMDEFLFHRAHEENPKGIHKVVPILGDMELPGLGINEEDRKMLASKVTIIINAAAT
VKFDEKLSVSTAINVKGTKEVLKLAKECRNLKAITHVSTAFSNTQVKHIEEKFYEPPMSVE
ALEAISEVDEKLVESILPTLLGTRPNTYCFTKAVAEEAVRTYGEGLPICIVRPSIVVSTYEEPV
RGWTDSVYGPTGLVVGIGTGVLRTMYMDQEKVADMVPVDLCVNAILASAWHTAKNYK
ENQTSHIPIYNYVSGAQNPLTWGEFIERNRRYGIDKPTTKAVWYYGLNPTNNYYLFLFYNF
FLHYLPALMIDTYCAITGKRRAMLKLYSKVMKLANILFYFSTQDWKFSDMNVRNMWNS
LSDADRVVFPFSMGEMSWEYMCETFLVGLRVYLIKDDLSTLPEARKKWNKLYYLHQILKI
ITLSLVLYLTYFVLQPIIALVFN
>*SlitFAR11*
MSTEPGDGPLLPGFYAGRAVLITGGTGFLGKVLIERLLWTCPEVGEVHLLLRDKRGQPPRV
RLNQLKQSQAFDNVRAHCPGQLDKLRVVCGDVAQPRLGLDDAALLQLREVSLVFHSAAT
VKFWESLETALHQNVTSVVALMELCDQLPRLEALVHVSTAYSNAERRHIEERVYEAPAQL
AGLRAMLDALPPSLLDDLTARYIAPKPNTYVFSKAVAEATIAQRPRKHYATAIVRPSIVVSS
HRHPYPGWIENLAGPSGVVVGCGKGLVHAFNLDLAARADLIPVDITIDTMLAVAWEIATD
KSEEVRVYNSCSQQNPITWGTFRDRVVRNARAHPFDQLMYYPFTFGIKNRYVYKALELVL
QTIPLYVADYIARLCGIKLQLSLVTVSERLQAMNRVLAFFATREWYFSTRNVQALRRRLTR
ADQDIYNLDVTSVDWDEHVSNFVKGTRKFLLKEKDDNIPRAKKFVERLRRVHQFVLLML
SVLVYRFLMLLLPRFLFRSSPVLAGLAAPH
>*SlitFAR12*
MADPSQVRSFYAGKNFFITGGTGFVGLCLIEKILRCIPDAGKIYLLMRSKKGKEIADRLQEF
PKNPVFEKLLESNSADIFKKLIPIAGDVGEENLGLSPQDRQTIIDNVNVVIHSAATLDFQESL
RPTVNINLLGTRRVMQLCKDAKNLKVMIHVSSAYVNSYLTEAHEKVYEAPEDPEKVISLV
GTLNDEALLEVEPKLLKSHPNTYTFTKHLAEHEVVKCADLFPCTIVRPTMIVAAWKEPIPG
WTCSKVGPQGFLMGAAKGVVRRLPLAKENVADYIPVDVVVNQLLVAGWEAANSRSGLT
VYHCSSSTCHPFTWTMLDDTVNSMLHKYPLKSAVWYPHLKFVPSLLMFRISAIFVHFFPA
LLLDLMLRMTGGRPILIRLHKNVWNSLNRLERFIFSEWKFHNPNTLELATKLNQTDKELFF
IDISKLYWVEYFKTLHLGVRRYLNKEKESSLPAARKKDMVLLLFHVIWQLFIMGLVWYIF
ACFTGLTLAHSAWIAPIIYILFTFL
>*SlitFAR13*

MAARADMLPVDMAIDTLLAVAWETAVDRPEAVRVYNCSTCENPTTWRDFETALRHRLRV
NPLDNAFWYPSGFTVENKLTQKTLETILQTAPLHIAEYISKILGIKTRLSLITVSQRLIAMNE
VLRFFSVREWHFVTDNVRKLHARLTPQDAAIYNLDPQTINWNEHYCNFIIGARKYLLQEK
DQDINEAKKHLRRMYYLHHGVMFFVVTLLCRLALRNQYLRAFIYRTFRMLLTVAGSAYM
RIRQS

>*SinfFAR1*

RLKEERPNDLNKVIPIAGDINLLDLGISAKDEEILIQKVSVVFHIAATIRFTEELKDAVNVNV
TGTKYVLQLCQRMKNIKAFVHVSTAYSNPEKHILEEKMYPPPAKLTEVQTFLDQSISDSNQ
IKALLKGAPNTYTFTKALAETYVAENYGNIPTVIIRPSIVSASLREPL

>*SinfFAR2*

MVVRATGDAEKPSVAEFYAGKSVFITGGTGFLGKVFIEKLLYSCPDIAKVFMLIREKKGLSI
SERMNQFLDDPLFNRLKDKRSTDLKKIVLIPGDISVPGLGISREIENILLETVSVIIHSAATVK
LNEPLPTAWKINVEGTRSVVALSKRMKRIEVFIHISTAYTNTNRKVVEEILYPPPADINEVYQ
LLEDGITEEDTERLLNGRPNTYTFTKSFSEHVVAENQVRVPTIIVRPSIVGSVKDHPIKGWL
NNWFGATGLSVFTARGMNRVLYGRSSNVVDLIPADYVANLVIAAGAKSGNEKSSELKVY
NCCSSSCNQITLGTVMAKFTEDAVNQPSSDMLLPGWYVFTRYKWMMTVWTILLQVIPAY
LADTYRILTRKNPRYVKLQSLVAQTQTALRFFTFNSWVMKADRVRELFASLSPADKDIFPC
DPISIDWNEYIKDYCCGVRQFLEKRK

>*SinfFAR3*

METVEQISAYESVAGFYAGKSVFVTGFTGFLGKVFLEKLLYSCKDIGDVYVLIRAKKSKDI
KQRLEETFNKPLFARLKEERP

>*HassFAR*

MVVLTSKETKPSVAEFYAGKSVFITGGTGFLGKIFIEKLLYSCPDIGNIYMLIREKKGLSVSE
RIKQFLDDPLFTRLKEKRPADLEKIVLIPGDITAPDLGITSENEKMLIEKVSVIIHSAATVKFN
EPLPTAWKINVEGTRMMLALSRRMKRIEVFIHISTAYTNTNREVVDEILYPAPADIDQVHQY
VKDGISEEETEKILNGRPNTYTFTKALTEHLVAENQAYVPTIIVRPSVVAAIKDEPIKGWLG
NWYGATGLTVFTAKGLNRVIYGHSSYIVDLIPVDYVANLVIAAGAKSSKSTELKVYNCCSS
ACNPITIGKLMSMFAEDAIKQKSYAMPLPGWYVFTKYKWLVLLLTILFQVIPAYITDLYRH
LIGKNPRYIKLQSLVNQTRSSIDFFTSHSWVMKADRVRELFASLSPADKYLFPCDPTDINWT
HYIQDYCWGVRHFLEKKTTNK

>*AperFAR1*

MNNNSHINLETSREVDTDVVSENDEYESVADFYSGKSVFITGATGLLGKVCVEKLLYTCT
NIDKIYILIRDKKGLHIDQRMASFFEQPVFSRIKREKPDVLKKVIPISGDITADNLGLKLEDE
QLLIQTVSVVIHSAATIKFNEPLKIAFNINVEGTRRVLNLAKRMHNIKVFVYVSTAYSNTNQ
KILDEIIYPPSIELDELKHIIAYQHIRGDDEVELKKLLKFHPNTYTFTKSLAENLVAKEHGDL
PIVIIRPSIITASEKEPISGWIDNWGGATAIITFIMNGWNKMYLCDGNIYLDMIPVDYVTNLIL
TSAAKCYRTKSVQVFHSCTSSENPITVNEMLNTLRVYNEKRNIDSKSIILTRSKFLVTCLTLII
HTIPAYVADLWMRACGKKEKYVKLHFQIVAHRDALEYFTTNSWVMRCGGTLSLFNSLNV
CDRQLFPCDPRDIVWTEYMDTYVSGIFKYLLKE

>*AperFAR2*

MNIMSDKINKEEFYQPVAEFYEDKSVFITGATGFLGKVLLERLLWNCPKIDKVYVLVREK
KGVSVDRRIEELLDNVLFSKLKDNRPDDLKKIIPIPGDIAVNNLGMNDADENTLIEKVAIVI
HTAASIKFKNPLKESLKINLEGTNQILNLCHRMKKLEVFVHTSTAYTNTDKKVIEEIVYPPP
ANLSDVYLQLEQNLDEDFTKVLNGQPNTYTFTKALTENLIMDRNRNLTTILIRPSIVTAVAN

EPLPGWIDNWYGASATLSSISKGINRILWGNVDNIVDLIPVDYVANFFLIAIAKGQRSESVV
VYNCCSSFSNPIRYRGLFRAFVDDAIKHRNYIYPFPQIYFMTTNKIIESIVKIFLEILPAFIADC
VGWLFNKPTGFLKLQKRIVAKREVIKFFTTNSWMMQDLGARALYTSLSTSDQNQFPCDV
TQLNWDEYILTYSAGIRKHLENK

>*AperFAR3*

MTSEVNEWYRGRKVLITGSSGFMGKVLMEKLLYSVPDIGCVYALARSKRGKSPESRIEDM
WKLPLFSRIREEKPHVMKKVIPVSGDIMYDNLGINKDQLEQIYNEVSVVFHFAASLRLEAP
LKEGLEMNTRGTLRVLELSKNIKNLAAFVHLSTAFCYPDYERMAEKVHDPPEDPHRVLEA
AGWISDEQFNLLAPSIMKMHPNSYTYSKRLAEALVRESYPELPAVVVRPSIVTPTYKEPIPG
WVDNLNGPIGLMVGAGKGVIRSMHCYGHYHAEVIPVDIAINAIIVTAYKTGKSTERSPEIP
VFNITTGDDRNTTWKQVLDIGKASVRKFPFEGPLWYPDGNIRHSKFIHDLCVFFYHLLPAY
FIDFLLLIFRQRRFMVRIQNRISVGLEVLQYFTTREWWFDTDNFKSLVTILNPIDQALFPMD
LTIIEDEPYIESCMIGGKLYCMKEKLENLPRARLHNNILYLLDRLVTALFYLFLLSWVASYF
EPVRYLLSYGGPVVRLLPFLGKAVFSDD

>*AperFAR4*

MAKDITTHVDFKKKFEDFTDLDMWEEKSLGESQIEKFFSGTTVLLTGGTGFLGKLFVEKL
LRCCPDIHKLYLLTRPKKDKSITKRLQEQFDDVLYDKLRKERPDFIKKIAIIEGDVGAIDLG
MSLKDRNKIVNEVEIIFHGAATVRFDEPLKTAVEINVRGTREICQLARACDKLKSLVHISTA
YSNCVLNEIEERFYESPLPGEKLIDLVETVDETIINNITPGLLGDYPNTYAYTKGVAEDIVQK
YSKGLPVALFRPSIVIGTATEPVSGWIDNVYGPTGVVVGAAVGLLHVLNCNPSAVADLVPG
DMVVNACIATAWKTAKEYPGNHEDAPPPDLPPPVYNFVSSVQQPLTWRNFMKYNETYGL
EVPTVQAVYYYLFHLTPSRFIYSLYCFFLHWIPAYIIDGVAVLIGRKPMLRKAYRKISKFSEVI
GYFATREWKFHNDNVKSLYTELCDADKQMFDFDLSRLDWSDYFESYVRGVRIYLLKDPM
DTIPAGLKKHYKLKYAHYFLCLVLLSLTLRLFWSILCMVIDF

>*AperFAR5*

MSEESQIRAFYSGKSFFITGGTGFVGLCLLEKILRCIPDVGKIYLLMRPKKGKEISQRLEEFA
KHPVFEKLLENKSADIFNKLIPIAGDVGEDNLGINARDRQTLIDNVNVVIHSAATLDFQDN
LRPTVNINLLGTRRVMALCKEIKNLKVMIHVSSAYVNSYLTDVHEKIYDCPEDPEKVISLV
GTLNDAALDEIEPKLLKDHPNTYTFTKHLAEHEVVKCAELFPCTIVRPTMIVASWKEPVPG
WTCSKVGPQGFIMGASKGVVRRIPIAIENIADYIPVDVVVNELLVAGWHAATTKSGITVYH
CSSSTQNPFRWSMLESTVNGNLHKYPLKSAVWYPHLKFVSSLWLFKLSAIFVHFIPAILLD
MVLRLTGGRPILFRLHKNVWSSLGLLERFIFTEWHFHNPKTIELSKELNKTDSELFYINIAS
LNWNEYFTDLLLGVRRYLNKEKDSTLKAARNKDTVLLGFHIVWQLVIIAMFWYIPAWIFG
LTIYQSAWVAPVMYFLYSFL

>*AperFAR6*

MASCLSFGPGSQEYVPVAEFYADKSIFVTGGTGFMGKVLVEKLLRSCPKIKKIYLLMRPKR
GQDVASRLNELTQSPLFETIRKERPQELTKIVPIVGDITEPELGISPADQTMLCQKVSVVFHS
AATVKFDEKLKLSVTINMLGTQQLVQLCHRMLGLEALVHVSTAYCNCERERVEETVYAPP
AHPEHVVTLVQTLPDELVDRITPDLVGDRPNTYTFTKALAEDMLIKESGNLPVAIVRPSIVL
SSVREPVKGWVDNWNGPNGIIAAVGKGIFRTMLGTGTRIADLVPVDTVINLMIVCAWRTH
LRRGDGVVVYNCCTGQQNPITWQQFVRSSFKYMRKHPFTDVVWYPGGDITSNRLKHGA
LCLVQHRAPAVIMDFVSRATGNNKPMMVRVQNKLDKAAACLEYFTTRQWAFADDNVQA
LCSSLSAEDQRTFDFNVRNIDWDAYIESYVLGIRRFLFKESPDTLPKSRALLRRLHIVHILTQ
VAGVILLWRFLFYRSNALRNIWRRVIDMLTRAARLLAIA

>*AperFAR7*
MNHSVEDSPLLDTSLTAEKMEKWIEAQRKGEKIDIDVYGKPTEKQLRELEHVRNLSKELQ
DNLHELENSVRIAEIENQDMNPTAPILDFSEDHEFVSANQLENCYDEEDKVDAREEEKRK
LTKGNAVSTVIQNFYKDQCVFVTGGTGFLGKVLIEKLLRSCVGINTIYVLIRNKKGKDYTT
RLQELLDEFLFHKAHEINPKGIHKVVPINGDMQLPGLGISDTDRKTLTSNVSIIINAAATVK
FDEKLSVSTAINVKGTKEVLKLAKECRNLKAVTHVSTAFSNTHVSYIEEKFYEPPMTVEAV
EAISELDEHLLESILPTLLGKRPNTYCFTKAIAEEAVRIYGEGLPICIVRPSIVISTYEEPMRG
WTDSVYGPTGLVVGIGTGVIRTMYMDLDKVADMVPVDLTVNAILASVWHTARNFKENQ
TSEIPIYNYVSGAQNPIKWRQFIDVNRRNGINNPTTKAVWYYGLNPTNCYYMFMFYNFFL
HYLPALLIDVYCLLSGKRRKTLALYGKVMRMSNILFYFSTQDWKFEDDGVKAMWNTLSE
DDKTVFPFSMSEMSWDYMCETFLVGLRVYLLKDDLSTLPEARMKWNKLFAIHLILKLATL
TFVLYMVYILLRPIFSFVFI
>*AperFAR8*
MESIMTVRDFYKGRNLLITGGTGFMGKVLIEKLLYSIPDIGNIYILMRPKRGKSISQRLEDM
QRLPLFDRLRQERPSAFNKLKPLQGDVLFDDFGLSSLDIDNLSKEVSVLFHFAATLRLEAPL
KDNVNMNTAGTMRTLNVARRLKKLVILIHLSTAFCYPDYEVLGEKLYPPPVKPENVMNLI
EWLDDRQLALLTPSLLGPHPNCYTFSKRLAENVVEQAYNELPVVIARPSIVCPAYTEPLPG
WVDSLNGPVGVMLGAGKGVIRTMLCDGSLYAQVIPVDIAINAIIAIGMIEGLKKDKTETIP
VYNVNIGHQMPTTWGYVLEVAKDYGRQYPLSWPLWYPNGDITTNYTLHEMRRIFYHLLP
AYVIDFILFLVGQKRFMVRIQDRISQGLEVLQYFTIRPWNFPCPNYDGIQTKLNEEEKIIFNT
DITNSNRAEYMKNCVEGGRVYCLKEDPTKVSINRSYHNFLYVLDWICKIMFWLFVFSFLA
SWYEPVSDILKYGEPVVKHLPFLGPAVFKDTN
>*AperFAR9*
FKGKTVFVTGGSGFMGKVLIEKLLYSCPDVEKIYLLLRSKKGIHSEDRLSSIYSSECFNRLR
KEKPDIFQSKVSVVNGDITEIGLGLSAEDRHILTDQVNIIFHVAASVRFNDPMKYAVKMNL
RGTREVVELAKDIKNLMVLVHLSTSYSNTNREVLEEVMYPPHADWRDTLAVTESFDDHT
LDVLTPKYLGELPNTYTFTKQLGEHVMYEQRGRLPIVILRPSIVISSLAEPMPGWIDNFNGP
VGLLVASGKGVMRSIYTDPNLVADYVPVDVAIKAIITSAWSRGTKQLESTDDIPIYNCCSSK
IYRVTLGDIIEMGMELSKEYPLNDVLWHVGGNVTNSKTLHYLKVLLLHLLPAILVDTILWA
LGRKPILIKVQRRIYTANLALHYYITQQWTFDNTNVLKLLESIKDEDKGHFHIDFYNIDVK
QFFRKSFFGARKYLLKE
>*AperFAR10*
SGFVRGRVLILCLFYNVVKNTIYSMSCEGYSAEEVDSMPDRIADTFTGLTVFITGGTGFMG
KVLVEKLLRKCPNIGHIILLVRTKKGKNPRQRLEDIFNGELFEKLRNLRGGIEPLLEKVSIAS
GDVGEPDLALSAADRERITNDVDIIIHAAATIRFDEELKKAVLLNVRGTKLMVELGKQCK
KLKLFIHVSTSYCHLHEKLLEEKPYPPPADPHQVIQAVEWMDTETVAALTPKLLDKLPNSY
AFTKALGEALVVEAMEHMPAMVLRPSIVIPIWQEPIPGWTDNINGPTGLLIGAGKGVIRSM
YCKSNSYADYLPVDVFINGIMIAIWNYIVYGDKESNIINFTSSAEIKVTWSEMIDAGREIIM
NRMPLNGVAWYPGGSMKHSRLYHNICVFFFHWVPAVIVDTLLFCLGYKPVLMRVQRRIN
KGFEVFEYYTNNQWDFKSDIAQTVRRRLNTRERRDYKVDAVGVDISKYFEDCIRAARIYI
LKEYDDTLPAARRHMRVMWWVDLITRIIFWGLILYWISGWFSSFSSFTEGSNETPTVLSMD
A
>*AperFAR11*
MSPTVAEFYAGKTVFITGGTGFVGKVLVEKLLRSCPDVKTICLLMRPKKGYSCEERLHDL

CSSKVFDLVRRDNPGAFNKLKFIDGDITVDGLGISDEDNEMLRSECNIIFHSAACVRFDQK
LKEAVNMNTTGTLRLLTLAETMNKLQAFIHLSTAFCRCELDVLEEKLYKAVHSPRRIMDIV
DWMDNDTLDYLEPKLISSEPNTYSYTKAITEDLVAEYGNKFPLAIARPSIVTPAWKEPFPG
WVDNLNGPVGIFVGGSKGIIRSMLCEGSYKADVISVDIVVNACILIAYVTALDKPKATQVY
NVTLSRAIKISWYQVIKLGEKWVHEYPYTLALWYPGGSLKSSRIMHKISVAVTHYLPAYLI
DGLLYILGKKTFMVKVQKKVSRGLEVLEYYTMKEWNFKNTNFLALRHRVDKSDEKTFFT
DASECNADEYLRNCVLGAREYCLKEDSSTLPRARTLHKIRYVADRIMKFISFCLLFWFLYC
YRDVFLSALELLDCYLKNFTLNPIKAKESCESN
