# Supplementary material for: Genome-Wide Identification and Expression Profiling of Candidate Sex Pheromone Biosynthesis Genes in the Fall Armyworm (Spodoptera frugiperda)
Source: Insects. 2022 Nov 23;13(12):1078. doi: 10.3390/insects13121078 (PMC9783692; doi:10.3390/insects13121078)
Supplement: Supplementary file 1 [file insects-13-01078-s001.zip › Table S4-Primer amplicon characteristics of 4 genes for qRT-PCR.pdf]

Table S4 Primer amplicon characteristics of 4 genes for qRT-PCR.

Gene Name	Efficiently (%)	R^2	Linear regression equation
SfruDES2	106.0	0.9990	y=-3.186x＋19.196
SfruDES5	98.0	0.9999	y=-3.3706x＋16.571
SfruFAR2	102.1	0.9999	y=-3.273x＋20.2
SfruFAR3	108.4	0.9985	y=-3.1348x＋16.327
