# Supplementary material for: Genome-Wide Identification and Expression Profiling of Candidate Sex Pheromone Biosynthesis Genes in the Fall Armyworm (Spodoptera frugiperda)
Source: Insects. 2022 Nov 23;13(12):1078. doi: 10.3390/insects13121078 (PMC9783692; doi:10.3390/insects13121078)
Supplement: Supplementary file 1 [file insects-13-01078-s001.zip › Table S5-Summary of the transcriptome sequencing data of of Spodoptera frugiperda.pdf]

Table S5 Summary of the transcriptome sequencing data of of *Spodoptera frugiperda*

Samples	Total raw reads	Clean reads	Clean bases	≥Q30 (%)	GC (%)	Total Mapped reads	Total Mapped ratio
Pheromone gland-1	44514412	43013248	6.45G	91.21	45.99	33927289	78.88
Pheromone gland-2	47957898	47215578	7.08G	90.33	45.73	37180432	78.75
Pheromone gland-3	45285150	44386310	6.66G	90.79	45.34	34881462	78.59
Abdomen-1	43864586	42119612	6.32G	91.16	44.91	32663382	77.55
Abdomen-2	45225078	43985546	6.60G	91.11	43.09	33920468	77.12
Abdomen-3	47856708	46709310	7.01G	90.96	43.09	36146741	77.39
